# Functional Annotation of Conserved Hypothetical Proteins from *Haemophilus influenzae* Rd KW20

**DOI:** 10.1371/journal.pone.0084263

**Published:** 2013-12-31

**Authors:** Mohd Shahbaaz, Md. ImtaiyazHassan, Faizan Ahmad

**Affiliations:** 1 Department of Computer Science, Jamia Millia Islamia, Jamia Nagar, New Delhi, India; 2 Center for Interdisciplinary Research in Basic Sciences, Jamia Millia Islamia, Jamia Nagar, New Delhi, India; Russian Academy of Sciences, Institute for Biological Instrumentation, Russian Federation

## Abstract

*Haemophilus influenzae* is a Gram negative bacterium that belongs to the family *Pasteurellaceae,* causes bacteremia, pneumonia and acute bacterial meningitis in infants. The emergence of multi-drug resistance *H. influenzae* strain in clinical isolates demands the development of better/new drugs against this pathogen. Our study combines a number of bioinformatics tools for function predictions of previously not assigned proteins in the genome of *H. influenzae*. This genome was extensively analyzed and found 1,657 functional proteins in which function of 429 proteins are unknown, termed as hypothetical proteins (HPs). Amino acid sequences of all 429 HPs were extensively annotated and we successfully assigned the function to 296 HPs with high confidence. We also characterized the function of 124 HPs precisely, but with less confidence. We believed that sequence of a protein can be used as a framework to explain known functional properties. Here we have combined the latest versions of protein family databases, protein motifs, intrinsic features from the amino acid sequence, pathway and genome context methods to assign a precise function to hypothetical proteins for which no experimental information is available. We found these HPs belong to various classes of proteins such as enzymes, transporters, carriers, receptors, signal transducers, binding proteins, virulence and other proteins. The outcome of this work will be helpful for a better understanding of the mechanism of pathogenesis and in finding novel therapeutic targets for *H. influenzae*.

## Introduction

*Haemophilus influenzae* strain Rd KW20 is a Gram-negative bacterium frequently isolated from the lower respiratory tract of patients with chronic bronchitis [1], [2] which is the “fourth-most-common” cause of death in the United States [1]. Due to comparatively small genome size and its phylogenetic closeness to *Escherichia coli*, *H. influenzae* is a very convenient model organism for genomic and proteomic findings [3], [4], [5]. The genome of *H. influenzae* was successfully sequenced [6], and it consists of 1,830,140 base pairs in a single circular chromosome that contains 1740 protein-coding genes, 2 transfer RNA genes, and 18 other RNA genes [6]. Due to successful sequencing of whole genome, *H. influenzae* serve as a model organism for whole-genome annotation, computational analysis and cross-genome comparisons [7]. Furthermore, genome-scale model of metabolic fluxes construction [8], [9], [10] and whole-genome transposon mutagenesis analysis [11], [12] was first implemented in *H. influenzae*. Moreover, in this study it is also used as a test genome to evaluate the performance of various bioinformatics approaches for proteome analysis, with the ultimate aim of determining the *in silico* properties of the protein set expressed by the bacterium under certain conditions.

Genomic analysis of 102 bacterial genomes shows that the respective genomic pool contain 45,110 proteins organized in 7853 orthologous groups with unknown function [13]. Proteins with unknown function may be termed as Hypothetical Proteins (HPs) or putative conserved proteins because these proteins are showing limited correlation to known annotated proteins [14], [15]. The HPs have not been functionally characterized and described at biochemical and physiological level [15]. Nearly half of the proteins in most genomes belong to HPs, and this class of proteins presumably have their own importance to complete genomic and proteomic information [16], [17]. We have been working on structure based rational drug design where we always need a selective target for drug design [18], [19], [20]. A precise annotation of HPs of particular genome leads to the discovery of new structures as well as new functions, and helps in bringing out a list of additional protein pathways and cascades, thus completing our fragmentary knowledge on the mosaic of proteins [17]. Furthermore, novel HPs may also serve as markers and pharmacological targets for drug design, discovery and screen [21], [22].

The use of advanced bioinformatics tools for sequence analysis and comparison is an initial step to identify homologue for only a part of the region shared between proteins, which could lead to a robust function prediction. Most commonly used method for functional prediction of gene products is by identification of related well-characterized homologues using sequence-based search procedures such as BLAST [23]. Multiple sequence alignment of homologues of a family is a suitable method to obtain structurally/functionally important positions and structurally conserved domains. We have considered functional domains as the basis to infer the biological role of HPs. Motif analysis is an obligatory step in the identification and characterization of HPs. Detection of common motifs among proteins in particular with absent or low sequence identities (e.g. less than 30%) may provide important clues for function or classification of HPs into appropriate families [24]. A series of signature databases are publically available, and are used for motif finding including GenomeNet [25] (contains PROSITE [26], PRINTS [27], Pfam [28], ProDom [29], BLOCKS [30]) and InterPro [31] using InterProScan [32]. A potent method for motif searches represents the use of MEME suite [33], a resource for investigating candidate's functional and structural motifs/sites in HPs **(** **Table 1** **)**. Furthermore, study of protein interactions using STRING database [34] is crucial to understand the functional role of individual proteins in a well-organized biological network.

**Table 1:** List of bioinformatics tools and databases used for sequence based function annotation.

S. No.	Software name	URL	Remark
1) **Sequence similarity search**			
**1.**	BLAST: Basic Local Alignment Search Tool	http://www.ncbi.nlm.nih.gov/BLAST/	BLASTp is used for finding similar sequences in protein databases
**2.**	HHpred	ftp://toolkit.genzentrum.lmu.de/pub/HH-suite/	Protein homology detection by HMM-HMM comparison
2) **Physicochemical characterization**			
**3.**	ExPASy – ProtParam tool	http://web.expasy.org/protparam/	Used for computation of various physical and chemical parameters
3) **Sub-cellular localization**			
**4.**	PSORT B	http://www.psort.org/psortb	PSORTb attained an overall precision of 97%
**5.**	PSLpred	http://www.imtech.res.in/raghava/pslpred/	The overall accuracy of PSLpred is 91.2%.
**6.**	CELLO	http://cello.life.nctu.edu.tw	The overall accuracy of CELLO is 91%.
**7.**	SignalP	http://www.cbs.dtu.dk/services/SignalP/	Predict signal peptide cleavage sites
**8.**	SecretomeP	http://www.cbs.dtu.dk/services/SecretomeP/	Predict bacterial non-classical secretion
**9.**	TMHMM	http://www.cbs.dtu.dk/services/TMHMM/.	Predict membrane topology
**10.**	HMMTOP	http://www.enzim.hu/hmmtop/	Predict transmembrane topology
4) **Sequence alignment**			
**11.**	PRALINE (PRofile ALIgNEment)	http://ibivu.cs.vu.nl/programs/pralinewww/	Integrates homology-extended and secondary structure information for multiple sequence alignment
5) **Protein classification**			
**12.**	Pfam	http://pfam.sanger.ac.uk/.	Collection of multiple protein-sequence alignments and HMMs
**13.**	CATH (Class, Architecture, Topology, Homology)	http://www.cathdb.info/	Hierarchical domain classification of PDB structures
**14.**	SUPERFAMILY	http://supfam.cs.bris.ac.uk/SUPERFAMILY	Based on SCOP database
**15.**	SYSTERS	http://systers.molgen.mpg.de	-
**16.**	SVMProt	http://jing.cz3.nus.edu.sg/cgi-bin/svmprot.cgi.	SVM based classification with accuracy of 69.1–99.6%
**17.**	CDART (The Conserved Domain Architecture Retrieval Tool)	http://www.ncbi.nlm.nih. gov/Structure/Lexington/Lexington.cgi.	NCBI Entrez Protein Database search of domain architecture
**18.**	PANTHER (Protein Analysis THrough Evolutionary Relationships)	http://www.pantherdb.org	Classification based on HMM-HMM search
**19.**	ProtoNet	http://www.protonet.cs.huji.ac.il	Based on automatic hierarchical clustering of the protein sequences
**20.**	SMART (Simple Modular Architecture Research Tool)	http://smart.embl.de/	Identification and annotation of protein domains
6) **Motif Discovery**			
**21.**	InterProScan	http://www.ebi.ac.uk/InterProScan/	Searches InterPro for motif discovery
**22.**	MOTIF	http://www.genome.jp/tools/motif/	Japanese GenomeNet service for motif discovery
**23.**	MEME Suite	http://meme.nbcr.net	-
7) **Clustering**			
**24.**	CLUSS	http://prospectus.usherbrooke.ca/cluss/	Clustering on the basis of Substitution Matching Similarity (SMS)
8) **Virulence factor analysis**			
**25.**	VirulentPred	http://bioinfo.icgeb.res.in/virulent/	Accomplish an accuracy of 81.8%
**26.**	VICMpred	http://www.imtech.res.in/raghava/vicmpred/	Attain accuracy of 70.75%.
9) **Protein-protein interaction**			
**27.**	STRING (Search Tool for the Retrieval of Interacting Genes/Proteins)	http://string-db.org	Version –9.05

Here we have used recent bioinformatics tools to assign function to all HPs encoded by *H. influenzae* genome. The Receiver Operating Characteristic (ROC) analysis [35] is used for evaluating the performance of used bioinformatics tools. We also measured the confidence level of the function prediction on the basis of used bioinformatics tools [36]. The function prediction has high confidence level if more than three tools indicate the same functions. While if there is less than three tools then it is less confidently predicted function [36]. So, we have successfully assigned functions to all 296 HPs of *H. influenzae* genome with high confidence. We have performed an extensive sequence analysis of proteins associated with virulence using tools like Virulentpred [37] and VICMpred [38], because *H. influenzae* is the causative agent of infection in respiratory tract.

## Materials and Methods

**Figure 1:**
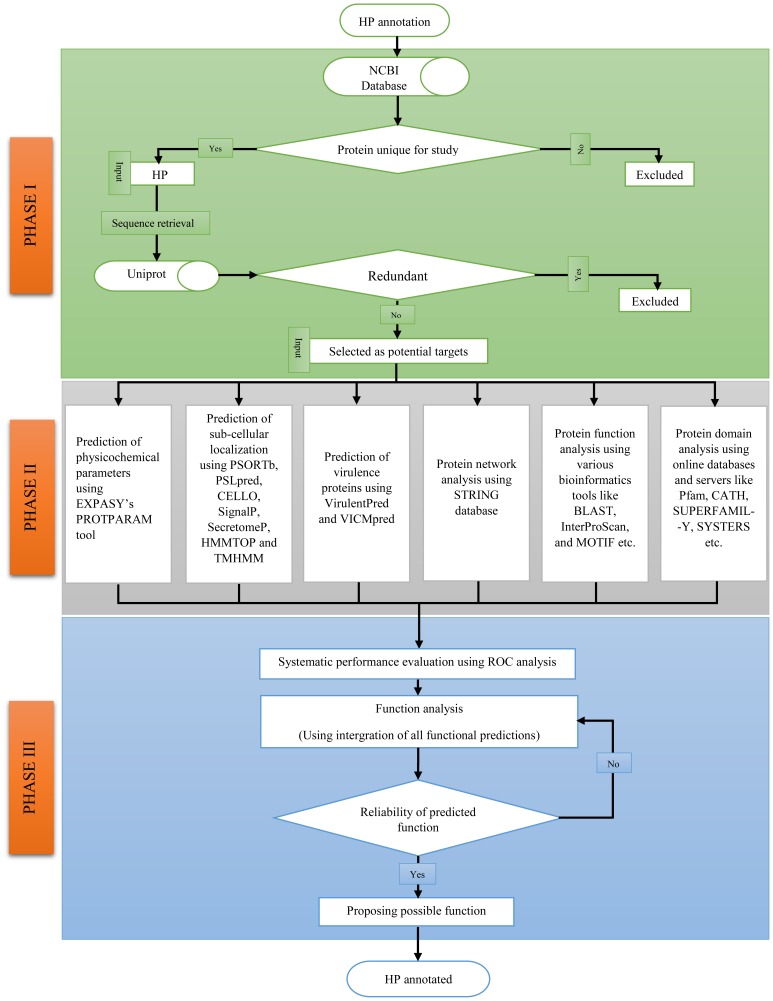
Computational framework used for annotating function of 429 HPs from *H. influenzae.* Methodology is divided into three phases: **PHASE I**. *H. influenzae* HP characterization and sequence retrieval from online databases. **PHASE II**. The extensive analysis of sub-cellular localization, physicochemical parameters, virulence, function and domain present in HPs. **PHASE III**. This phase include assessment of predicted functions using the protein with known function from *H. influenzae* and reliable prediction of possible functions of HPs.

The computational framework used for functional annotation of HPs is given in **Figure 1**, is divided into three phases namely, Phase I, II and III. The Phase I include the characterization and sequence retrieval of HPs by analyzing the genome of *H. influenzae*. The Phase II comprises the automated annotation of various functional parameters using various online servers. In Phase III, the systematic performance evaluation of various bioinformatics tools by using *H. influenzae* protein sequences with known function by performing ROC analysis. The probable functions of the characterized HPs were predicted by the integration of various functional predictions made in PHASE II. In latter phase expert knowledge is used for performing ROC analysis and for confidently annotating the HPs functional properties.

### Sequence retrieval

We have analyzed the genome of *H. influenzae* and found 1,657 proteins present in it (http://www.ncbi.nlm.nih.gov/genome/). The 429 proteins are characterized as HPs and their fasta sequences were retrieved from UniProt (http://www.uniprot.org/) using the primary accession number of all HPs.

### Physicochemical characterization

Expasy's ProtParam server [39] has been used for theoretical measurements of physiochemical properties such as molecular weight, isoelectric point, extinction coefficient [40], instability index [41], aliphatic index [42] and grand average of hydropathicity (GRAVY) [43]. These predicted parameters are listed in **Table S1**.

### Sub-cellular localization

A protein can be characterized as drug or vaccine target by utilizing the knowledge of sub-cellular localization. The proteins localized in cytoplasm can act as possible drug targets, while surface membrane proteins are considered as potent vaccine targets [44]. Databases like UniProt provide valuable information about sub-cellular location of proteins [45]. If experimental information about HP localization is absent, then we have used sub-cellular localization prediction tools like PSORTb [46], PSLpred [47] and CELLO [48], [49]. CELLO (version 2.0) two-level support vector machine based system, which comprises 1444 and 7589 protein sequences as standard datasets for the prediction of bacterial and eukaryotic protein localization, respectively [48], [49]. The PSLpred is used only for predicting sub-cellular localization of Gram negative bacteria. We have used SignalP 4.1 [50] for predicting signal peptide and SecretomeP [51] for identifying protein involvement in non-classical secretory pathway. TMHMM [52] and HMMTOP [53] have been used for predicting the propensity of a protein to be a membrane protein. The sub-cellular localization predictions of 429 HPs are listed in **Table S2**.

### Sequence comparisons

The first step towards predicting the functionality of a protein is generally a sequence similarity search in various available gene and protein databases. We have used BLASTp [23] and HHpred [54] for searching similar sequences with known function. BLAST is a popular bioinformatics tool, most frequently used for calculating sequence similarity by performing local alignments. The BLASTp search against the non-redundant protein sequences (nr) database returns 100 homologs of each HP, and proteins with low query coverage (<50%) or low sequence identity (<20%) are excluded. Proteins showing high sequence identities (>40%) and e-value (<0.005) are referred to as close homologs of HPs and those with low identities (<26%) are considered as remote homologues. The search with the highest value of the respective parameters considered as probable function of the given HP. The BLASTp also used for checking the availability of structural homologs in Protein Data Bank (PDB). Whereas, HHpred utilizes pair wise comparison of profile hidden Markov models (HMMs) for remote protein homology detection by searching various protein databases like PDB [55], [56], SCOP [57], CATH [58], etc. is also used for detection of structural homologs. We have used BLASTp for determining the sequence identity between two proteins sequences and PRALINE [59] for multiple sequences comparison **(Table S3)**.

### Function prediction

We have used various tools for precise functional assignments to all 429 HPs from *H. influenzae* are described in **Table 1**. The functional domain of a protein is predicted by using various publically available databases such as Pfam, SUPERFAMILY [60], CATH, PANTHER [61], SYSTERS [62], SVMProt [63], CDART [64], SMART [65], and ProtoNet [66] **(Table S4)**. The database SYSTERS was used for clustering proteins on the basis of their functions. We used BLASTp for searching SYSTERS database and the output is obtained in the form of clusters of functionally related proteins. The clusters with e-value (<0.005) are considered as a proper classification of HP. SVMProt was used for the SVM based classification of proteins into 54 functional families from its primary sequences. The significance level of classification is measured in the form of R-value and P-value (%), classification with R-value (>2.0) and P-value (>60%) are considered as significant. CDART and SMART were used for similarity search based on domain architecture and profiles rather than by direct sequence similarity. The Simple modular architecture research tool (SMART) search for similar domain in Swiss-Prot [67], SP-TrEMBL [68] and stable Ensembl [69] proteomes in normal mode. The search with e-value (<0.005) was considered as a significant match for the given HP.

Similarly, PANTHER is a comprehensively organized database of protein families, trees and subfamilies, used to develop evolutionary relationships to infer the functions of HPs. The HMM- based search is performed on PANTHER database for functional annotation of HPs and important hits with e-value greater than 1e-3 are reported in the output. ProtoNet (Version 6.0) tree provided an automatic hierarchical clustering of the protein sequences. The “Classify your protein” option in ProtoNet is used for assignment of a biological function to HPs.

Protein sequence motifs are signatures of protein families and can often be used as tools for the prediction of protein function, particularly in enzymes, in which motifs are associated with catalytic functions. We used InterProScan which combines different protein signature recognition methods from the InterPro consortium which is the integration of several large databases, including PANTHER, Pfam, SMART, ProSite and SUPERFAMILY etc. for motif discovery. The output generated by InterProScan is presented in the form of the checksum of the protein sequence which is supposed to be unique, e-value of the match which should be less than 0.005 and status of the match in the form of true (T) or unknown (?), indicative of reliability of the generated result. The MOTIF and MEME suite have been used to perform motif- sequence database searching and assignment of function. The MOTIF tool generates a very large set of output and to identify the probable function of the HP we check whether the SCOP database predicted fold in HP is also present in the MOTIF generated functional annotations. While in motif discovery using MEME suite we first cluster the protein sequences of HPs into clusters using CLUSS [70], [71] online server and then submit the clustered sequences in the MEME suite server. MEME suite server identified three motif sites in the clustered HPs by default. The MAST [33] module of MEME suite then perform database searching for assigning function to the discovered motifs in the HPs.

### Virulence factors analysis

Virulence factors (VFs) are described as potent targets for developing drugs because it is essential for the severity of infection [72]. For identifying these VFs we have used VICMpred and Virulentpred. Both are SVM based method to predict bacterial VFs from protein sequences with an accuracy of 70.75% and 81.8%, respectively. Both methods use five-fold cross-validation technique for the evaluation of various prediction strategies.

### Functional protein association networks

The function and activity of a protein are often modulated by other proteins with which it interacts. Therefore, understanding of protein-protein interactions serve as valuable information for predicting the function of a protein. We have used STRING (version–9.05) [34] to predict protein interactions partners of HPs. The interactions include direct (physical) and indirect (functional) associations, experimental or co-expression. STRING quantitatively integrates interaction data from these sources for a large number of organisms, and transfers information between these organisms wherever applicable.

### Performance assessment

The statistical estimation of diagnostic accuracy is considered as an important step towards the validation of the predicted outcome of the adopted pipeline [73]. There are various available conventional methods for comparing the accuracy of various predicted models but ROC analysis is an extensively used method for analyzing and comparing the diagnostic accuracy [74], provides the most comprehensive explanation of diagnostic accuracy available till date [74]. We used six levels at which diagnostic efficacy can be evaluated. The two binary numerals “0” or “1” used to classify the prediction as true positive (“1”) or true negative (“0”). The integers (2, 3, 4 and 5) are used as confidence rating for each case. The ROC analysis is carried out for sequences of 100 proteins with known function from *H. influenzae*. We used the above explained *in silico* pipeline for the function prediction these known proteins using various online bioinformatics tools. We further classified the predicted function of proteins using already known function (Table S5 and S6). The classification results are submitted to “ROC Analysis: Web-based Calculator for ROC Curves” [75] in format 1 form as required by the software. This online software automatically calculates the ROC using the submitted data and generates the result in the form of accuracy, sensitivity, specificity and the ROC area. These generated parameters are utilized for validating the predicted functions of HPs. The average accuracy of used pipeline is 96.25% (Table S7) and indicates that outcomes of functional annotation of HPs are reliable that can be further utilized for other experimental research.

## Results and Discussion

### Sequence analysis

**Figure 2:**
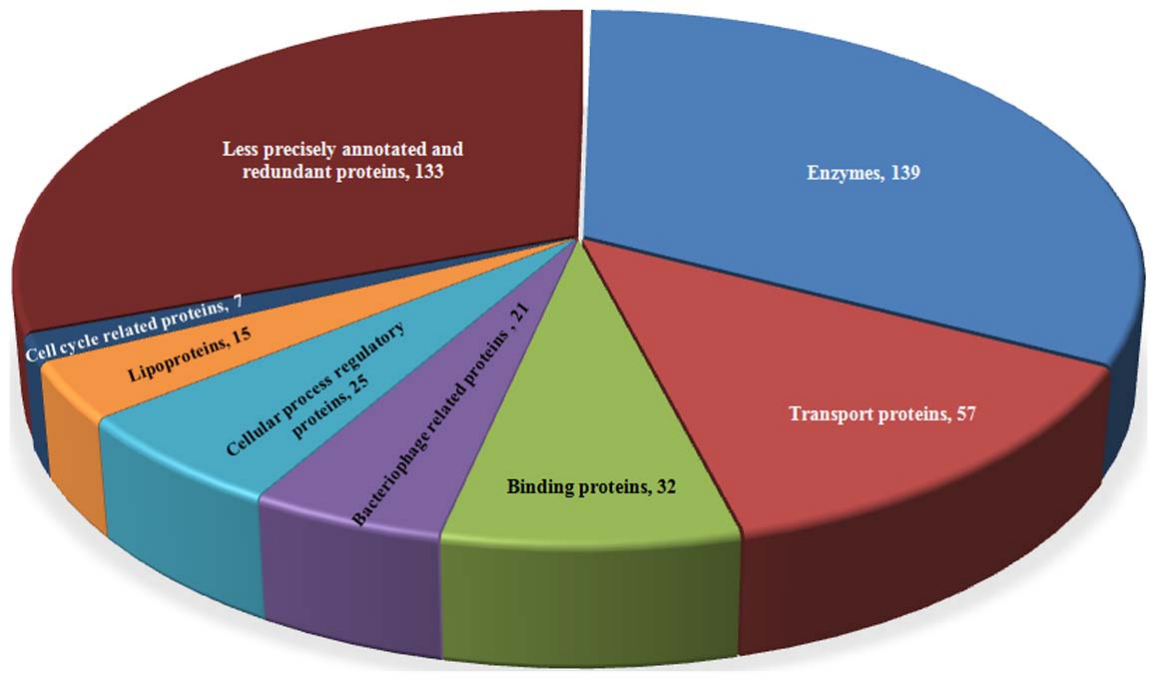
Classification of 429 HPs into various groups by utilizing the functional annotation result of various bioinformatics tools. The chart shows that there are 41% are enzymes, 20% proteins involve in transportation, 12% binding proteins, 7% bacteriophage related proteins and rest are proteins involved in cellular processes like transcription, translation, replication etc., among 429 HPs from *H. influenzae*.

We have extensively analyzed sequences of 429 HPs using BLAST, Pfam, PANTHER, CATH, CDART, and SVMProt. Tools like InterProScan, MOTIF, and MEME suite were used for discovering functional motifs in the HPs. We have successfully assigned a proposed function to each of 429 HPs present in *H. influenzae* **(Table S3 and Table S4**) and discovered motif in 420 HPs using MEME suite using 208 predicted clusters of CLUSS [70], [71] online software tool (**Table S8**), among which 296 HPs are characterized with high confidence and are listed in **Table 2** **,** and less confident annotated proteins are listed in **Table S9**. All sequence analyses were compiled. It was observed that in HPs present in *H. influenzae,* there are 139 enzymes, 57 transporters, 32 binding proteins, 21 bacteriophage related proteins, 15 lipoproteins and the rest are involved in various cellular process like transcription, translation, replication, etc. **(** **Figure 2** **)**. These analyses suggest a possible role of HPs in the development and pathogenesis of the organism, and identified groups are described here separately.

**Table 2:** List of annotated HPs from *H. influenzae.*

S. NO.	PROTEIN NAME	GENE ID	UNIPROT ID	Protein Function
1.	HP HI0020	950917	Q57048	Sodium/sulphate symporter
2.	HP HI0034	950928	P44471	Protein Iojap ribosomal silencing factor RsfS
3.	HP HI0035	950933	P44472	K+ uptake protein TrkA
4.	HP HI0044	950935	P44477	Bax inhibitor-1 like protein
5.	HP HI0051	950946	P44484	TRAP-type transporter system, small permease component
6.	HP HI0052	950947	P71336	TRAP type C4 dicarboxylate transport system, periplasmic component
7	HP HI0056	950954	P43932	Integral membrane protein TerC
8.	HP HI0065	950963	P44492	P-loop containing nucleoside triphosphate hydrolases
9.	HP HI0077	950975	P43935	Ferritin- like protein
10.	HP HI0080	950976	P43936	PemK-like family protein
11.	HP HI0081	950980	P44500	TatD related DNase
12.	HP HI0082	950979	P43937	Acyl-CoA dehydrogenase
13.	HP HI0090	950992	P44506	Alanine racemase
14.	HP HI0091	950989	P44507	Glycerate kinase
15.	HP HI0092	950987	Q57493	Gluconate transporter
16.	HP HI0093	950994	P44509	Putative sugar diacid recognition
17.	HP HI0094	950995	P43939	GntP family permease
18.	HP HI0095	950997	Q57060	Methyltransferase type II
19.	HP HI0103	951002	P44515	Arsenate reductase (ArsC protein)
20.	HP HI0105	951007	Q57354	NIF3-like protein (metal-binding protein)
21.	HP HI0112	951016	P71339	Transposase
22.	HP HI0118	951021	Q57097	Ubiquitin activating enzyme
23.	HP HI0125	951038	P44530	xanthine/uracil/vitamin C permease
24.	HP HI0134	951034	P43952	sugar transporter (AsmA-like C-terminal domain protein)
25.	HP HI0143	951052	P44540	HTH-type transcriptional regulator
26.	HP HI0146	951056	P44542	sialic acid transporter, TRAP-type C4-dicarboxylate transport system, periplasmic component
27.	HP HI0147	951057	P44543	C4-dicarboxylate ABC transporter permease
28.	HP HI0149	951059	P43953	protein-S-isoprenylcysteinemethyltransferase
29.	HP HI0150	951060	P44545	Band 7 protein/HflC protease
30.	HP HI0152	951063	P43954	4′-phosphopantetheinyl transferase
31.	HP HI0175	951085	P44552	multi-copper polyphenol oxidoreductase laccase
32.	HP HI0177	951089	P44553	Tetratricopeptide repeat like
33.	HP HI0178	951088	P43961	Prokaryotic membrane protein lipid attachment site profile
34.	HP HI0217	951128	P43965	transposase IS200-family protein
35.	HP HI0220.2	951123	O86222	Uracil-DNA glycosylase
36.	HP HI0223	951139	P44579	DMT superfamily drug/metabolite transporter RarD
37.	HP HI0228	951145	P43966	glycosyltransferase family 8
38.	HP HI0242	949384	P44593	SulfurtransferaseTusA family
39.	HP HI0243	949380	P43971	Hemerythrin HHE cation binding domain protein
40.	HP HI0246	949373	P43972	Prokaryotic membrane lipoprotein lipid attachment site profile
41.	HP HI0257	949379	P71346	S30EA ribosomal protein/Sigma 54 modulation protein
42.	HP HI0270	950625	P44606	tRNA-dihydrouridine synthase C
43.	HP HI0275	949970	P43975	Sulphatases EC 3.1.6.
44.	HP HI0277	949404	P44609	SEC-C motif domain-containing protein
45.	HP HI0315	949441	P44634	DNA-binding regulatory protein, YebC
46.	HP HI0318	949431	P43984	isoprenylcysteine carboxyl methyltransferase family protein
47.	HP HI0325	950706	P44640	sodium:protonantiporter
48.	HP HI0326	949439	P43987	primosomal replication protein N
49.	HP HI0329	949459	P44641	Lysine 2,3-aminomutase
50.	HP HI0352	949950	P24324	CMP-neu5Ac-lipooligosaccharide alpha 2–3 sialyltransferase
51.	HP HI0367	949469	Q57065	transcriptional regulator with an N-terminal xre-type HTH domain
52.	HP HI0370	949833	P43989	TPR-like (Tetratricopeptide repeat)
53.	HP HI0371	949472	P44668	Fe-S cluster related protein IscX
54.	HP HI0374	950642	P44670	histidyl-tRNA synthetase
55.	HP HI0376	950630	P44672	iron-binding protein IscA
56.	HP HI0379	949480	P44675	Rrf2 family transcriptional regulator
57.	HP HI0380	949482	P44676	tRNA/rRNAmethyltransferase
58.	HP HI0386	950554	P44679	acyl-CoA thioesterase
59.	HP HI0388	950019	P43990	O-Sialoglycoproteinendopeptidase
60.	HP HI0391	949488	P43992	Rhamnogalacturonanacetylesterase -like domain family protein
61.	HP HI0395	949524	P43994	RnfH family Ubiquitin
62.	HP HI0396	950708	P44683	RmlC-like cupins
63.	HP HI0398	949499	P44684	ADP-ribose pyrophosphatase
64.	HP HI0407	949507	P44691	ABC transporter involved in vitamin B12 uptake, BtuC family protein
65.	HP HI0409	949412	P44693	Endopeptidases (Peptidase, M23/M37 family)
66.	HP HI0414	949402	Q57392	Porin, opacity type
67.	HP HI0420	949520	P43995	Ribbon-helix-helix superfamily protein
68.	HP HI0423	949527	P44702	tRNA (adenine-N6)-methyltransferase
69.	HP HI0441	949523	P31777	S-adenosyl-L-methionine-dependent methyltransferases
70.	HP HI0442	950773	P44711	YbaB/EbfC DNA-binding protein
71.	HP HI0449	949746	P43997	Prokaryotic membrane lipoprotein lipid attachment site profile
72.	HP HI0452	949660	P44717	cystathionine-beta-synthase CBS domain protein
73.	HP HI0454	949545	P44718	TatD type deoxyribonuclease
74.	HP HI0457	950653	P44720	aminodeoxychorismate lyase
75.	HP HI0466	949552	P44000	Aminomethyltransferase folate-binding domain family protein
76.	HP HI0467	949553	P44726	YICC alpha Helix stress-induced protein
77.	HP HI0487	950695	P44003	PTS-regulatory domain, PRD
78.	HP HI0489	949626	P44005	SNARE associated Golgi protein
79.	HP HI0493	949783	O05023	Transposase/integrase
80.	HP HI0500	949635	P44733	DNA recombination protein RmuC
81.	HP HI0510	949577	P44740	tRNA (adenine(37)-N6)-methyltransferase
82.	HP HI0520	949583	P44743	Radical SAM protein
83.	HP HI0521	950665	P44744	glycine radical enzyme, YjjI family
84.	HP HI0526	949589	P44012	Ribonuclease T2
85.	HP HI0552	949603	P44013	Glucose-6-phosphate 1-dehydrogenase
86.	HP HI0554	949606	P44014	Transposase IS200-like
87.	HP HI0561	950224	P44016	oligopeptide transporter, OPT family
88.	HP HI0562	949610	P44754	S4 RNA-binding domain
89.	HP HI0573	949619	P44759	DNA-binding domain/SlyX like
90.	HP HI0575	950683	P44761	YheO DNA-binding (transcription regulator)
91.	HP HI0577	949622	P44017	SulfurtransferaseTusD -like domain family protein
92.	HP HI0585	949628	P44018	C4-dicarboxylate anaerobic carrier
93.	HP HI0586	950596	P44019	C4-dicarboxylate anaerobic carrier
94.	HP HI0594	949632	P44023	C4-dicarboxylate anaerobic carrier
95.	HP HI0597	950123	P44771	Cof protein like hydrolase
96.	HP HI0617	950684	P44782	23S rRNA/tRNApseudouridine synthase A
97.	HP HI0627	950813	P44025	Succinate dehydrogenase assembly factor 2, -like domain family
98.	HP HI0633	950781	P44026	Voltage gated chloride channel
99.	HP HI0638	950538	P44796	High frequency lysogenization protein HflD
100.	HP HI0650	949696	P44028	Prokaryotic membrane lipoprotein lipid attachment site profile protein
101.	HP HI0656	950161	P44807	tRNAthreonylcarbamoyladenosine biosynthesis protein RimN
102.	HP HI0656.1	949423	P46494	Topoisomerase DNA binding C4 zinc finger
103.	HP HI0660	950644	P44031	Phage derived protein Gp49-like
104.	HP HI0665	949704	P44033	HipA-like N-terminal domain
105.	HP HI0666	949708	P44034	HipA-like N-terminal
106.	HP HI0666.1	949707	O86228	HTH-type transcriptional regulator
107.	HP HI0668	949710	P44812	cell division protein ZapB
108.	HP HI0677	950735	P44036	N-acetyl transferase, NAT family
109.	HP HI0687	949720	P71356	Multidrug resistance efflux transporter EmrE family
110	HP HI0694	950211	P44827	ribosomal large subunit pseudouridine synthase E
111.	HP HI0698	950204	P44038	bacterial surface antigen protein
112.	HP HI0700	949725	P44831	Regulator of ribonuclease activity B
113.	HP HI0704	949730	P44040	outer membrane antigenic lipoprotein B
114.	HP HI0710	950711	P71357	bifunctional antitoxin/transcriptional repressor RelB
115.	HP HI0711	949734	P44041	Plasmid stabilisation system protein RelE/ParE
116	HP HI0719	949739	P44839	Endoribonuclease L-PSP
117.	HP HI0722	949742	P44842	Translation elongation factor EFG, V domain
118.	HP HI0725	949753	P44043	coproporphyrinogen III oxidase
119.	HP HI0744	949771	P44854	rhodanese-related sulfurtransferase
120.	HP HI0755	949515	P44863	Polysaccharide deacetylase
121.	HP HI0756	950697	P44864	peptidase M23 family protein
122.	HP HI0760	949979	P44048	Fe(2+)-trafficking protein
123.	HP HI0762	949781	P44050	Calcineurin-like phosphoesterase
124.	HP HI0767	949786	P44869	16S rRNA m(2)G966 methyltransferase
125.	HP HI0804	950170	P44053	cAMP-dependent protein kinase regulatory subunit -like domain ½ family
126.	HP HI0806	949820	P44054	Sulfite exporter TauE/SafE family protein
127.	HP HI0827	949716	P44886	acyl-CoA thioester hydrolase
128.	HP HI0841	949855	P44898	Sulphatases EC 3.1.6.
129.	HP HI0842	949857	P44058	N-isopropylammelide isopropyl amidohydrolase
130.	HP HI0852	949865	P44903	Drug resistance transporter EmrB/QacA
131.	HP HI0857	950666	P44062	BolA family transcriptional regulator
132.	HP HI0858	949870	P44905	5-formyltetrahydrofolate cyclo-ligase
133.	HP HI0866	950756	P44063	lipopolysaccharide biosynthesis protein WzzE
134	HP HI0868	949464	Q57022	glycosyl transferase family A protein
135.	HP HI0869	949879	P44064	Glycosyltransferase
136.	HP HI0874	949882	P44067	O-antigen ligase WaaL
137.	HP HI0878	949421	P71360	multidrug resistance efflux transporter EmrE
138.	HP HI0902	949698	P44070	Sulfite exporter TauE/SafE
139	HP HI0906	949908	P44931	Cytidinedeaminase
140.	HP HI0912	950836	P44074	SAM dependent methyltransferase
141.	HP HI0918	949920	P44936	Peptidase M50 (metalloendopeptidase)
142.	HP HI0920	950624	P44938	Undecaprenyl pyrophosphate synthetase
143.	HP HI0925	950812	P44075	type I restriction enzyme M protein
144.	HP HI0926	949651	P44076	glutaredoxin-like protein (electron transport)
145.	HP HI0929	949927	P44940	Bifunctionalglutathionylspermidine synthetase/amidase
146.	HP HI0930	949932	P44077	Prokaryotic membrane lipoprotein lipid attachment site profile
147.	HP HI0933	949936	P44941	FAD/NAD(P)-binding oxidoreductase
148.	HP HI0938	949906	P44079	Type II secretory pathway, pseudopilin
149	HP HI0948	949840	Q57120	Antidote-toxin recognition MazE
150.	HP HI0960	950757	P44084	Prokaryotic membrane lipoprotein lipid attachment site profile
151.	HP HI0966	950444	P44085	Prokaryotic membrane lipoprotein lipid attachment site profile
152.	HP HI0973	949511	Q57133	transferrin-binding protein
153.	HP HI0976	949977	Q57147	EamA-like transporter family protein
154.	HP HI0976.1	949978	O86230	Multidrug resistance efflux transporter EmrE
155.	HP HI0979	949982	P44965	tRNA-dihydrouridine synthase
156.	HP HI0983	949986	P43907	Prokaryotic membrane lipoprotein lipid attachment site profile
157.	HP HI0984	949993	P43908	Peroxide stress response protein YAAA
158.	HP HI1005	949997	P44974	Sulphatases EC 3.1.6.
159.	HP HI1008	950002	Q57134	competence protein ComE
160.	HP HI1011	950004	P44093	D-Tagatose-1,6-bisphosphate aldolase
161.	HP HI1013	950733	Q57151	hydroxypyruvate isomerase
162.	HP HI1014	950006	P44094	Nucleoside-diphosphate-sugar epimerase
163.	HP HI1016	949991	P44095	cyclase family protein
164.	HP HI1028	949528	P44992	TRAP dicarboxylate transporter subunit DctP
165.	HP HI1029	949652	P44993	C4-dicarboxylate ABC transporter permease
166.	HP HI1030	950014	P44994	C4-dicarboxylate ABC transporter permease
167.	HP HI1037	950020	P44098	glutamine amidotransferase
168.	HP HI1038	950021	P44099	AAA+ superfamily ATPase
169.	HP HI1048	949536	P44103	transglutaminase family protein
170.	HP HI1053	950030	Q57498	Carboxymuconolactone decarboxylase
171.	HP HI1054	950034	P44104	Type III restriction-modification system restriction enzyme
172.	HP HI1058	949400	P44106	type III restriction/modification enzyme methylation subunit
173.	HP HI1064	950040	P71367	Sulphatases EC 3.1.6.
174.	HP HI1082	949428	P45026	BolA family transcriptional regulator
175.	HP HI1099	950069	P44112	Prokaryotic membrane lipoprotein lipid attachment site
176.	HP HI1146	950109	P45071	P-loop containing ATPase protein
177.	HP HI1152	950115	P45077	TldD/PmbA, Putative modulator of DNA gyrase
178.	HP HI1161	950121	P45083	Thioesterase
179.	HP HI1162	950122	P44116	Restriction endonuclease type II-like
180.	HP HI1163	950119	Q57252	FAD-linked oxidoreductase
181.	HP HI1165	949810	P45085	Glutaredoxin (electron carrier)
182.	HP HI1173	950125	P44119	Zinc metal-binding SPRT metallopeptidase
183.	HP HI1189	950138	P45097	Methyltransferase (radical SAM protein)
184.	HP HI1191	950043	P44124	7-cyano-7-deazaguanine synthase(QueC)
185.	HP HI1192	950139	P44125	Prokaryotic membrane lipoprotein lipid attachment site profile
186.	HP HI1198	950741	P45103	Sua5/YciO/YrdC/YwlC family protein (Double stranded RNA binding)
187.	HP HI1199	950150	P45104	ribosomal large subunit pseudouridine synthase B
188.	HP HI1202	950140	P44126	Smr protein/MutS2
189.	HP HI1208	950157	P71373	Amidophosphoribosyltransferase (Epimerase)
190.	HP HI1246	950184	P44135	Sulphatases EC 3.1.6.
191.	HP HI1248	950186	P44136	Nickel/cobalt transporter(ABC-type transport system)
192.	HP HI1250	950243	P44138	plasmid maintenance system killer protein (Toxin-antitoxin system)
193.	HP HI1253	950692	P44139	invasion protein expression up-regulator SirB
194.	HP HI1254	950259	P44140	tRNA(Met) cytidineacetyltransferase
195.	HP HI1265	950187	P44144	YcaO protein (Involved in beta-methylthiolation of ribosomal protein S12)
196.	HP HI1273	950164	P44150	S-adenosyl-L-methionine-dependent methyltransferases
197.	HP HI1282	950221	P45138	ribosome maturation protein RimP
198.	HP HI1292	949593	P44154	Zn-ribbon-containing protein (DNA binding protein)
199.	HP HI1293	950226	P44156	SufE protein probably involved in Fe-S center assembly
200.	HP HI1297	950233	P45145	LrgA like protein (Export murein hydrolases)
201.	HP HI1298	950227	P45146	murein hydrolase regulator LrgB
202.	HP HI1307	950239	Q57320	Lysine-type exporter protein (LYSE/YGGA)
203.	HP HI1309	950234	P45154	2Fe-2S ferredoxin-type domain (elctron carrier)
204.	HP HI1315	950581	P71375	Sodium/solute symporter
205.	HP HI1317	950209	P44160	Aldose 1-epimerase
206.	HP HI1323	950258	P44161	MacrodomainTer protein, MatP
207.	HP HI1327	950255	P44163	Prokaryotic membrane lipoprotein lipid attachment site profile
208.	HP HI1333	949671	P71376	RNA-binding, CRM domain
209.	HP HI1338	950260	P44164	phosphohistidine phosphatase SixA
210.	HP HI1339	950818	P71378	Late embryogenesis abundant protein
211.	HP HI1340	950814	P44165	Outer membrane efflux porinTdeA
212.	HP HI1343	949643	P71379	cysteine desulfurase, catalytic subunit CsdA
213.	HP HI1349	950182	P45173	DNA-binding ferritin-like protein
214.	HP HI1351	950443	P44167	tRNAmo(5)U34 methyltransferase, SAM-dependent
215.	HP HI1361	950286	P45180	Glycosyl transferase, family 35
216.	HP HI1369	950892	P45182	TonB-dependent receptor
217.	HP HI1376	950804	P44170	Multidrug resistance efflux transporter EmrE
218.	HP HI1388.1	950703	O86237	Tautomerase/MIF
219.	HP HI1394	950304	P44172	RNA binding domain (ASCH)
220.	HP HI1395	950305	P44173	zeta toxin family protein
221.	HP HI1400	950717	P44176	Polymerase and histidinol phosphatase like
222.	HP HI1413	949414	P44185	Prokaryotic membrane lipoprotein lipid attachment site profile
223.	HP HI1415	950713	P44187	Lysozyme-like superfamily protein
224.	HP HI1416	950758	P44188	Phage holin, lambda family
225.	HP HI1418	950323	P44189	BRO family, N-terminal domain
226.	HP HI1419	949900	P44190	Phage derived protein Gp49-like
227.	HP HI1420	950760	P44191	Helix-turn-helix protein
228.	HP HI1422	949966	P44193	antA/AntBantirepressor family protein
229.	HP HI1434	949657	P45202	Cys-tRNAPro/Cys-tRNACysdeacylaseybaK
23.0	HP HI1435	950339	P44197	tRNApseudouridine synthase C
231.	HP HI1436	950784	Q57152	RNA pseudouridine synthase C
232.	HP HI1454	950340	P44202	Cytochrome C biogenesis protein transmembrane region
233.	HP HI1462	950787	P45217	Outer membrane efflux porinTdeA
234.	HP HI1469	949595	P44205	molybdenum ABC transporter substrate-binding protein
235.	HP HI1475	950353	Q57380	molybdate ABC transporter, permease
236.	HP HI1479	950355	P44208	Transposase
237.	HP HI1493	950360	P44218	N-acetylmuramoyl-L-alanine amidase
238.	HP HI1497	950363	P44221	Zinc finger, DksA/TraR C4-type
239.	HP HI1498.1	950365	O86242	Ribonuclease R winged-helix domain protein
240.	HP HI1499	950366	P44223	Mu-like phage gp27
241.	HP HI1500	950367	P44224	Mu-like prophageFluMu protein gp28
242.	HP HI1501	950368	P44225	Mu-like prophageFluMu protein gp29
243.	HP HI1502	950369	P44226	F protein, phage head morphogenesis, SPP1 gp7 family domain protein
244.	HP HI1505	950373	P44227	Mu-like prophageFluMu major head subunit
245.	HP HI1508	950376	P44230	Mu-like prophage protein GP36
246.	HP HI1509	950377	P44231	Mu-like prophageFluMu protein gp37
247.	HP HI1510	950834	P44232	Mu-like prophageFluMu protein gp38
248.	HP HI1512	950378	P44234	Mu-like prophageFluMu tail tube protein
249	HP HI1513	950379	P44235	Mu-like prophageFluMu protein gp41
250.	HP HI1518	950383	P44238	Mu-like prophageFluMu protein gp45
251.	HP HI1519	950384	P44239	Mu-like prophageFluMu protein gp46
252.	HP HI1520	950385	P44240	Mu-like prophageFluMu protein gp47
253.	HP HI1521	950386	P44241	Mu-like prophageFluMu protein gp48
254.	HP HI1522	950387	P44242	Mu-like prophageFluMu defective tail fiber protein
255.	HP HI1522.1	950388	P71390	Mu-like prophage protein Com
256.	HP HI1523	949672	P44243	D12 class N6 adenine-specific DNA methyltransferase
257.	HP HI1534	950396	P44246	tRNA 5-methylaminomethyl-2-thiouridine biosynthesis bifunctional protein MnmC
258.	HP HI1536	950398	P44247	TRNA U-34 5-methylaminomethyl-2-thiouridine biosynthesis protein MnmC, C-terminal
259.	HP HI1542	950405	P45244	NAD(P)H nitroreductase
26.	HP HI1555	949639	P44252	Outer membrane-specific lipoprotein ABC transporter, permease component LolE
261.	HP HI1558	950418	P45252	Tetratricopeptide repeat (TPR) like
262.	HP HI1559	950419	P45253	N5-glutamine S-adenosyl-L-methionine-dependent methyltransferase
263.	HP HI1560	950420	P44253	RDD domain-containing protein
264.	HP HI1562	950422	P44254	TPR repeat, Sel1 subfamily protein (key negative regulator of the Notch pathway)
265.	HP HI1564	950424	P44256	DNA polymerase IV
266.	HP HI1571.1	950429	Q4QKT3	bacteriophage replication protein A
267.	HP HI1581	950440	P44262	Glyoxalase/Bleomycin resistance protein/Dihydroxybiphenyldioxygenase
268.	HP HI1598	950454	P45267	adenylatecyclase
269.	HP HI1600	950455	P44268	Xylose isomerase-like, TIM barrel domain
270.	HP HI1602	950457	P44270	TQO small subunit DoxD family protein (subunit of the terminal quinol oxidase)
271.	HP HI1605	950458	P44272	SH3 domain-containing protein
272.	HP HI1625	950478	P44277	Sel1 repeat domain
273.	HP HI1627	950462	P71394	Endoribonuclease L-PSP
274.	HP HI1629	950844	P45280	SNARE associated Golgi protein
275.	HP HI1632	950850	Q57525	Aspartokinase
276.	HP HI1637	950851	P44280	P-loop containing nucleoside triphosphate hydrolases
277.	HP HI1650	950489	P44281	DEAD/DEAH box helicase/type I restriction endonuclease subunit R
278.	HP HI1651	950855	P44282	Signal transduction histidine kinase
279.	HP HI1654	950491	P45298	S-adenosylmethionine-dependent methytransferase
280.	HP HI1656	950807	P45300	Restriction endonuclease type II-like
281.	HP HI1657	950796	P52606	Sedoheptulose 7-phosphate isomerase
282.	HP HI1658	950803	P45301	Transport-associated and nodulation domain, bacteria (BON domain) (ion transport)
283.	HP HI1663	950497	Q57544	Metallo-beta-lactamase
284.	HP HI1664	950504	P45305	TatD-related deoxyribonuclease
285.	HP HI1665	950493	P44283	Hedgehog signalling/DD-peptidase zinc-binding domain/Peptidase_M15_2
286.	HP HI1666	950486	P44284	Hedgehog signalling/DD-peptidase zinc-binding domain/Peptidase_M15_2
287.	HP HI1667	950498	P44285	L, D-transpeptidase
288.	HP HI1671	950860	P44287	Paraquat-inducible protein A/Multihaem cytochrome (electron transport)
289.	HP HI1672	950502	P44288	Mammalian cell entry (MCE) related protein
290.	HP HI1680	950508	P44289	MFS general substrate transporter superfamily
291.	HP HI1709	950526	P44293	Viral OB-fold, YgiW
292.	HP HI1718	950877	P44296	trimericautotransporteradhesin
293.	HP HI1720	950873	Q57066	Transposase
294.	HP HI1728	950517	O05087	Mn2+ and Fe2+ transporter of the NRAMP family
295.	HP HI1730	950540	P44298	allophanate hydrolase subunit 2
296.	HP HI1731	950880	P44299	allophanate hydrolase subunit 1

### Enzymes

Enzymes produced by bacteria are key player for the survival of organism in their host because they provide nutrient for growth and responsible for pathogenesis of organism, for enzymes modify the local environment for favorable growth inside the host and metabolism of compounds inside the host [76]. We characterized 139 enzymes. Knowledge of these enzymes is important for understanding the host-pathogen interaction as well.

We identified 14 oxidoreductase enzymes, which are critically important for bacterial virulence and pathogenesis. It is well understood that the disulfide bonds are important for the stability and/or structural rigidity of many extracellular proteins, including bacterial virulence factors. Bond formation is catalyzed by thiol-disulfide oxidoreductases (TDORs). Oxidoreductases like SdbA is required for disulfide bond formation in *S. gordonii*, which is required for autolytic activity [77]. Protein P45154 contain 2Fe-2S ferredoxin-type domain. Many bacteria produce protein antibiotics known as bacteriocins to kill competing strains of the same or closely related bacterial species. We identified protein P44743 as a radical SAM (S-adenosylmethionine) protein, it is understood that radical SAM proteins play a significant role in pathogenesis of an organism and is also validated that the inhibition of these enzymes is effective in preventing the lethal diseases [78].

Similarly, we identified 39 transferase enzymes which are required for the efficient spore germination and full virulence of bacteria like *Bacillus anthracis.* Transferase enzymes are essential for biosynthesis of lipoprotein, and bacterial lipoproteins play an important role in virulence of bacteria [79]. Proteins Q57022, P44064 and P45180 are glycosyl transferase, and on mutation it affects extracellular polysaccharide (EPS) and lipopolysaccharide (LPS) biosynthesis, cell motility, and reduces the development of disease symptoms [80], [81]. We have characterized protein P44256 as DNA polymerase IV and it is observed that virulent strains contain increased level of activity of DNA polymerase than non-virulent strains, indicating its role in virulence [82].

The protein Q57544 is found to be a β-lactamase. The enzyme responsible for generation of resistance against β-Lactam antibiotics like penicillin, cephalosporins, etc. [83]. We annotated 56 hydrolase enzymes having an established role in virulence of bacteria, e.g. Kdo hydrolase is the main cause of virulence in *Francisella tularensis*, which is classified as a bioterrorism agent [84]. Similarly, nudix hydrolase encoded by nudA gene in *Bacillus anthracis* is important for the complete virulence [85].

There are 8 lyase enzymes. These are important for the virulence of pathogen in host [76]. The P44717 protein is a cystathionine β-lyase, an enzyme which forms the cystathionine intermediate in cysteine biosynthesis, may be considered as the target for pyridiamine anti-microbial agents [86]. Similarly, isocitrate lyase is an enzyme of glyoxylate cycle, which catalyzes the cleavage of isocitrate to succinate and glyoxylate together with malate synthase. This enzyme bypasses two decarboxylation steps of TCA cycle. It is found to up-regulate glyoxylate cycle during pathogenesis, and therefore, this pathway is used by bacteria, fungi, etc., for survival in their hosts [87].

The isomerase enzyme catalyze changes within one molecule by structural rearrangement [88] and isomerases like peptidylprolyl cis/trans isomerases (PPIases) involved in protein folding. These isomerases are considered as surface-exposed proteins which are important for virulence and resistance to NaCl [88]. We identified 13 isomerases and 5 ligases in a group of 139 enzymes. Ligase enzymes are also part of virulence in the hosts. It is found that E3 ligase activity associated with the C-terminal region of XopL, a type III effectors, which specifically interacts with plant E2 ubiquitin conjugating enzyme that induce plant cell death and subvert plant immunity [89]. There are also 4 HPs with kinase activity, which play a significant role in growth, differentiation, metabolism and apoptosis in response to external and internal stimuli [90]. Thus, such enzymes are important for the survival of pathogen and may serve as a target for drug design and discovery [91].

### Transport

Transport process plays a pivotal role in cellular metabolism, e.g., for the uptake of nutrients or the excretion of metabolic waste products, etc. We successfully predicted 50 transporters, 3 carriers, 3 receptors and 1 signal transduction proteins among HPs. It is recently identified that these proteins may be involved in virulence and essential for intracellular survival of pathogens [92]. The protein P44691 was predicted to be a member of ABC 3 transporter family, presumably involved in virulence because they are associated with the uptake of metal ions, such as iron, zinc, and manganese [93]. This protein also helps in the attachment of pathogenic bacteria to the mucosal surfaces of host cells, which is a critical step in bacterial pathogenesis, thereby present as a putative drug target [93].

We found protein P44005 and P45280 as SNARE associated Golgi protein. The soluble N-ethylmaleimide-sensitive factor attachment protein receptors (SNARE) proteins play an essential role in the compartment fusion in eukaryotic cells [94]. They share a conserved motif, known as SNARE motif, and have been classified as glutamine containing SNAREs (Q-SNAREs) and arginine containing SNAREs (R-SNAREs) on the basis of favorably conserved residue at the center of this motif [95]. These proteins are central regulators of membrane fusion, so they are potential targets for intracellular organisms, which frequently rely on destabilizing the host intracellular traffic. This finding helps us to conclude that by mimicking SNAREs some inclusion proteins can control intracellular trafficking.

Bacteriocins proteins contain an N-terminal domain with an extensive resemblance to a [2Fe-2S] plant ferredoxin and a C-terminal colicin M-like catalytic domain and to gain entry into vulnerable cells. These proteins parasitize an existing iron uptake pathway by using a ferredoxin-containing receptor binding domain [96]. Protein Q57133 is a transferrin-binding protein. Transferrins are a group of non-haem iron-binding glycoproteins, widely distributed in the physiological fluids and cells of vertebrates. These proteins are involved in iron transport within the circulatory system of the vertebrates. Transferrins is important for bacterial virulence but their role in virulence is still not fully understood [97]. The membrane transferrin receptor-mediated endocytosis is a major route of cellular iron uptake and the efficient cellular uptake of transferrin pathway has shown potential in the delivery of anticancer drugs, proteins, and therapeutic genes into primarily proliferating malignant cells over expressed transferrin receptors [98], [99].

### Binding Proteins

32 HPs are annotated as binding proteins in which 15 are DNA binding, 5 RNA binding, 9 metal binding and 3 ATP/coenzyme binding proteins. We have identified a tetratricopeptide repeat (TPR), a structural motif involved in the assembly of various multi-protein complexes in many HPs. TPR-containing proteins often play important roles in cell processes, and involved in virulence-associated functions [100].

HPs function as DNA-binding proteins also contribute to the virulence. The winged-helix-turn-helix (wHTH) motif in sarZ proteins in *Staphylococcus aureus* contributes to virulence by binding to *cvf* gene that encodes for alpha hemolysin [101]. In complex regulatory system of group A *Streptococcus* (GAS), there is the streptococcal regulator of virulence (Srv) which is the member of the CRP/FNR family of transcriptional regulators, and members of this family possess a characteristic C-terminal helix-turn-helix motif (HTH) that facilitates binding to DNA targets. Point mutation in this motif alters protein-DNA interaction [102], indicate that DNA binding motifs are regulatory factors of the virulence of bacteria. The RNA binding proteins are also contributing to the survival of the organism and control the virulence factors of the pathogens [103].

### Lipoprotein

Lipoproteins identified in bacteria are formed by lipid modification of proteins that facilitate the anchoring of hydrophilic proteins to hydrophobic surfaces through hydrophobic interactions of the attached acyl groups to the cell wall phospholipids. This process has a considerable significance in many cellular and virulence phenomena. We found 15 lipoproteins from the group of HPs because they play crucial roles in adhesion to host cells, variation of inflammatory processes and translocation process of virulence factors into host cells. It is also discovered that lipoproteins may function as vaccines. The knowledge of these facts may be utilized for the generation of novel countermeasures to bacterial diseases [104].

### Other Proteins

Structural motifs like helix-turn-helix are conserved in various organisms. A detection of these common patterns in a sequence refers that such proteins are mainly involved in the regulation of transcription. The transcription regulators like HilC and HilD also showed DNA binding activities and contributes to the virulence of *Salmonella enterica*, where these are involved in the invasion to the host cells [105]. We found 18 transcriptional regulatory, 3 translation regulatory, 1 replication regulatory, 3 cell cycle regulatory enzyme/protein. The regulatory protein RfaH is found in *E. coli* and enhances the expression of different factors that are supposed to play a role in the bacterial virulence. Furthermore, inactivation of rfaH decreases the virulence of uropathogenic *E. coli* strain [106]. Similarly, the RNA-binding protein Hfq has emerged as an important regulatory factor in varieties of physiological processes, including stress resistance and virulence in various Gram-negative bacteria such as *E. coli*. Hfq modulates the stability or translation of mRNAs and interacts with numerous small regulatory RNAs [107]. The cell cycle and related protein P44063, is involved in lipopolysaccharide biosynthesis and are important in understanding the virulence of *H. influenzae,* as proteins involved in this particular biosynthesis are considered as primary virulence factors [108].

### Virulent proteins

We use the consensus of VICMpred and VirulentPred for predicting the virulence factors among the 429 HPs and found 40 HPs that give positive virulence score in both servers, and can be used as potent drug targets for drug design. These are listed in **Table 3**. In this group of virulent proteins we observed that protein P43936 is a PemK superfamily toxin of the ChpB-ChpS toxin-antitoxin system protein involved in plasmid maintenance [109]. We have also identified 30 bacteriophage related proteins among HPs. It is known that SuMu protein 1a, a bacteriophage related protein, has shown homology to IgA metalloproteinase and IgA1 protease which are described as virulence factors in non-typeable *H. influenzae* [110]. So, SuMu proteins are considered as highly virulent proteins.

**Table 3:** List of HPs with virulence factors in *H. influenzae.*

S No.	UNIPROT ID	Virulent proteins	
		Virulentpred	VICMpred
**1.**	**P71336**	Yes	Yes
**2.**	**P43936**	Yes	Yes
3.	P44553	Yes	Metabolism molecule
**4.**	**P44609**	Yes	Yes
**5.**	**P44670**	Yes	Yes
**6.**	**P44675**	Yes	Cellular process
**7.**	**P43990**	Yes	Cellular process
**8.**	**P44693**	Yes	Cellular process
**9.**	**Q57144**	Yes	Cellular process
**10.**	**P44733**	Yes	Cellular process
**11.**	**P44740**	Yes	Yes
**12.**	**P44023**	Yes	Yes
13.	Q57523	Yes	Yes
**14.**	**P44038**	Yes	Cellular process
**15.**	**P44041**	Yes	Information and storage
**16.**	**P44863**	Yes	Yes
**17.**	**P44054**	Yes	Yes
**18.**	**P44063**	Yes	Cellular process
**19.**	**Q57120**	Yes	Cellular process
**20.**	**Q57133**	Yes	Yes
**21.**	**P43907**	Yes	Cellular process
**22.**	**P44972**	Yes	Cellular process
**23.**	**P45074**	Yes	Cellular process
**24.**	**P45077**	Yes	Cellular process
**25.**	**P71373**	Yes	Yes
**26.**	**P44132**	Yes	Metabolism molecule
**27.**	**P44138**	Yes	Cellular process
**28.**	**P44140**	Yes	Yes
**29.**	**P44165**	Yes	Yes
**30.**	**P45182**	Yes	Yes
**31.**	**P44169**	Yes	Yes
**32.**	**P44183**	Yes	Yes
**33.**	**P56507**	Yes	Yes
**34.**	**P45217**	Yes	Yes
**35.**	**P44242**	Yes	Cellular process
**36.**	**P44246**	Yes	Yes
**37.**	**P44288**	Yes	Metabolism molecule
**38.**	**P44293**	Yes	Yes
**39.**	**P44296**	Yes	Metabolism molecule
**40.**	**P44298**	Yes	Yes

### Conclusions

Using an innovative *in silico* approach we have analyzed all 429 HPs from *H. influenzae.* Using the ROC analysis and confidence level measurements of the predicted results, we precisely predict the function of 296 HPs with confidence and successfully characterized them. We did not find enough evidences for functional prediction of 124 proteins, and hence these sequences require further analysis. The sub-cellular localization and physicochemical parameters prediction are useful in distinguishing the HPs with transporter activity from the rest of the protein. The protein-protein interaction also helps to find out the involvement of such proteins in various metabolic pathways. Further, we are able to detect the 40 virulence proteins essential for the survival of pathogen, particularly protein Q57523 showing highest virulence score in VICMpred which is known to be the most virulent HP among the listed virulence proteins. Our results could facilitate in developing drugs/vaccines, specifically targeting the pathogen's system without causing any allergic or side effect to the host. This *in silico* approach for functional annotation of HPs can be further utilized in drug discovery for characterizing putative drug targets for other clinically important pathogens.

## Supporting Information

Table S1 List of predicted physicochemical parameters by Expasy's ProtParam tool of 429 HP from *H. influenzae.*
(DOCX)

Table S2 List of predicted sub-cellular localization of 429 HPs from *H. influenzae.*
(DOCX)

Table S3 List of annotated functions of 429 HPs from *H. influenzae* using BLASTp, STRING, SMART, INTERPROSCAN and MOTIF.
(DOCX)

Table S4 List of functionally annotated domains of 429 HPs from *H. influenzae* by CATH, SUPERFAMILY, PANTHER, Pfam, SYSTERS, CDART SVMProt and ProtoNet.
(DOCX)

Table S5 List of annotated functions of 100 proteins with known function from *H. influenzae* using BLASTp, SMART, INTERPROSCAN and MOTIF for ROC analysis.
(DOCX)

Table S6 List of functionally annotated domains of 100 proteins with known function from *H. influenzae* by CATH, SUPERFAMILY, PANTHER, Pfam, SYSTERS, CDART SVMProt and ProtoNet for ROC analysis.
(DOCX)

Table S7 List of accuracy, sensitivity, specificity and ROC area of various bioinformatics tools used for predicting function of HPs from *H. influenzae* obtained after ROC analysis.
(DOCX)

Table S8 List of clusters formed by CLUSS online tool and predicted motif sequence site and sequence by MEME Suite in 429 HPs from *H. influenzae.*
(DOCX)

Table S9 List of annotated HPs at low confidence from *H. influenzae.*
(DOCX)
