# Correction: Functional Annotation of Conserved Hypothetical Proteins from *Haemophilus influenzae* Rd KW20

**DOI:** 10.1371/annotation/23d005b8-fe53-4b14-a31c-915be3e839b5

**Published:** 2014-01-13

**Authors:** Mohd Shahbaaz, Md. Imtaiyaz Hassan, Faizan Ahmad

In the online version of the article, a space was erroneously omitted from the second author's name. The correct name is Md. Imtaiyaz Hassan. The correct citation is:

Shahbaaz M, Hassan MI, Ahmad F (2013) Functional Annotation of Conserved Hypothetical Proteins from Haemophilus influenzae Rd KW20. PLoS ONE 8(12): e84263. doi:10.1371/journal.pone.0084263

The name and citation are correct on the PDF.

