# Supplementary material for: Functional Annotation of Conserved Hypothetical Proteins from Haemophilus influenzae Rd KW20
Source: PLoS One. 2013 Dec 31;8(12):e84263. doi: 10.1371/journal.pone.0084263 (PMC3877243; doi:10.1371/journal.pone.0084263)
Supplement: Table S1 — List of predicted physicochemical parameters by Expasy's ProtParam tool of 429 HP from H. influenzae. (DOCX) [file pone.0084263.s001.docx]

| **S.NO**  Table S1: List of predicted physicochemical parameters by Expasy’s ProtParam tool of 429 HP from *H. influenzae* | **UNIPROT ID** | **Molecular weight, M_w_**  **(Da)** | **Theoretical PI** | **Extinction coefficient**  **(M^-1^ cm^-1^)** | **Instability Index** | | **Aliphatic index** | **Grand average of hydropathicity**  **(GRAVY)** |
| --- | --- | --- | --- | --- | --- | --- | --- | --- |
|  |  |  |  |  | **computed** | **classification** |  |  |
| 1 | **Q57048** | 51353.58 | 9.40 | 104975 | 25.11 | stable | 125.43 | 0.858 |
| 2 | **P44465** | 10506.23 | 6.28 | 7450 | 29.24 | stable | 95.33 | -0.291 |
| 3 | **P44471** | 11386.06 | 4.74 | 12615 | 25.17 | stable | 88.92 | -0.063 |
| 4 | **P44472** | 59013.77 | 7.72 | 53985 | 26.33 | stable | 129.33 | 0.664 |
| 5 | **P43929** | 30174.91 | 4.37 | 33015 | 37.67 | stable | 88.41 | -0.338 |
| 6 | **P43931** | 40436.22 | 8.57 | 60055 | 41.69 | unstable | 111.89 | -0.005 |
| 7 | **P44477** | 24245.91 | 7.82 | 14900 | 33.38 | stable | 125.05 | 1.010 |
| 8 | **P44478** | 17704.43 | 9.36 | 19480 | 15.41 | stable | 95.82 | -0.133 |
| 9 | **P44484** | 18580.29 | 6.55 | 15025 | 32.34 | stable | 122.97 | 0.901 |
| 10 | **P71336** | 35164.49 | 8.77 | 55350 | 37.53 | stable | 96.04 | -0.364 |
| 11 | **P43932** | 26230.70 | 6.30 | 20970 | 35.59 | stable | 145.23 | 0.900 |
| 12 | **P44492** | 17969.54 | 4.74 | 18910 | 41.42 | stable | 96.96 | -0.164 |
| 13 | **P43935** | 33032.89 | 5.79 | 52035 | 37.65 | stable | 98.99 | -0.218 |
| 14 | **P43936** | 16292.12 | 9.64 | 14565 | 54.83 | stable | 111.05 | -0.201 |
| 15 | **P44500** | 29789.19 | 6.14 | 21680 | 37.33 | stable | 94.06 | -0.215 |
| 16 | **P43937** | 7500.77 | 6.69 | 13075 | 42.19 | unstable | 89.89 | 0.382 |
| 17 | **P43938** | 6009.06 | 9.45 | 6990 | 42.46 | unstable | 74.23 | -0.746 |
| 18 | **P44506** | 26766.58 | 5.99 | 17085 | 50.23 | unstable | 88.14 | -0.447 |
| 19 | **P44507** | 39758.74 | 4.90 | 6335 | 34.53 | stable | 102.99 | 0.147 |
| 20 | **Q57493** | 42781.26 | 9.28 | 7450 | 30.00 | stable | 133.94 | 0.977 |
| 21 | **P44509** | 42250.89 | 7.75 | 68485 | 38.68 | stable | 108.89 | -0.156 |
| 22 | **P43939** | 11735.14 | 9.30 | 8605 | 32.28 | stable | 121.42 | 0.482 |
| 23 | **Q57060** | 28050.51 | 8.65 | 14105 | 31.35 | stable | 85.54 | -0.278 |
| 24 | **P43940** | 21909.73 | 4.33 | 23840 | 35.85 | stable | 96.96 | -0.196 |
| 25 | **P44515** | 13059.94 | 6.82 | 20970 | 29.54 | stable | 97.54 | -0.378 |
| 26 | **Q57354** | 27813.76 | 5.64 | 30160 | 23.44 | stable | 96.81 | -0.291 |
| 27 | **P44520** | 32821.16 | 8.83 | 23295 | 20.40 | stable | 120.51 | 0.707 |
| 28 | **P43943** | 9076.20 | 7.97 | 9970 | 30.31 | stable | 85.40 | -0.557 |
| 29 | **P71339** | 11900.02 | 10.49 | 15930 | 16.41 | stable | 97.45 | -0.237 |
| 30 | **Q57097** | 28572.02 | 8.85 | 16305 | 36.75 | stable | 94.90 | -0.020 |
| 31 | **P43947** | 19159.09 | 8.85 | 39670 | 35.34 | stable | 135.30 | 0.961 |
| 32 | **P44530** | 45298.40 | 7.65 | 29575 | 30.54 | stable | 133.40 | 1.148 |
| 33 | **P43952** | 44575.23 | 8.67 | 19620 | 36.31 | stable | 102.30 | -0.131 |
| 34 | **P44540** | 31465.19 | 6.54 | 9065 | 41.19 | unstable | 104.65 | -0.061 |
| 35 | **P44542** | 34164.85 | 6.37 | 23840 | 29.12 | stable | 83.71 | -0.258 |
| 36 | **P44543** | 67592.58 | 9.18 | 66600 | 31.99 | stable | 128.39 | 1.010 |
| 37 | **O86220** | 7600.42 | 9.43 | 4470 | 32.87 | stable | 151.91 | 1.404 |
| 38 | **P43953** | 5306.20 | 9.60 | 27960 | 57.26 | unstable | 136.19 | 0.344 |
| 39 | **P44545** | 32901.71 | 9.55 | 17420 | 30.26 | stable | 90.92 | -0.219 |
| 40 | **P43954** | 27807.63 | 5.68 | 42650 | 48.86 | unstable | 92.13 | -0.367 |
| 41 | **P43790** | 19743.54 | 4.42 | 16180 | 44.00 | unstable | 99.08 | -0.217 |
| 42 | **P43960** | 9494.01 | 6.70 | 3230 | 41.63 | unstable | 84.07 | -0.090 |
| 43 | **P44552** | 27137.81 | 5.96 | 34170 | 23.38 | stable | 83.48 | -0.184 |
| 44 | **P44553** | 27498.11 | 8.40 | 27850 | 30.06 | stable | 80.92 | -0.268 |
| 45 | **P43961** | 16572.79 | 9.28 | 27515 | 34.72 | stable | 91.44 | -0.406 |
| 46 | **P46490** | 27608.50 | 9.73 | 29575 | 37.58 | stable | 119.02 | 0.939 |
| 47 | **P43963** | 26912.79 | 8.65 | 7450 | 32.43 | stable | 97.59 | -0.264 |
| 48 | **P43965** | 21403.20 | 7.72 | 44475 | 42.82 | unstable | 72.56 | -0.813 |
| 49 | **P44577** | 23338.46 | 7.74 | 35535 | 32.76 | stable | 103.05 | 0.441 |
| 50 | **O86222** | 25083.88 | 6.91 | 45380 | 49.90 | unstable | 90.65 | -0.275 |
| 51 | **P44579** | 34181.17 | 9.18 | 93640 | 36.46 | stable | 135.20 | 0.898 |
| 52 | **P44583** | 17670.33 | 5.08 | 13660 | 25.61 | stable | 91.10 | -0.323 |
| 53 | **P43966** | 14298.50 | 5.87 | 21095 | 45.17 | unstable | 110.08 | 0.155 |
| 54 | **P43968** | 5499.45 | 6.11 | 1490 | 12.33 | stable | 113.54 | 0.021 |
| 55 | **P44588** | 8153.60 | 10.36 | 4470 | 5.64 | stable | 60.72 | -1.083 |
| 56 | **P44593** | 8361.84 | 5.34 | 4470 | 29.59 | stable | 121.64 | 0.075 |
| 57 | **P43971** | 20408.09 | 4.98 | 20065 | 61.60 | unstable | 87.91 | -0.592 |
| 58 | **P43972** | 14000.81 | 5.36 | 10095 | 32.54 | stable | 87.57 | -0.049 |
| 59 | **P71346** | 12059.65 | 6.29 | 6990 | 45.09 | unstable | 86.64 | -0.667 |
| 60 | **P44606** | 35057.08 | 6.61 | 48400 | 36.64 | stable | 92.45 | -0.359 |
| 61 | **P43975** | 62256.74 | 5.87 | 80010 | 36.58 | Stable | 91.69 | 0.118 |
| 62 | **P44609** | 18372.14 | 6.93 | 24450 | 57.03 | unstable | 75.09 | -0.278 |
| 63 | **P43980** | 20920.86 | 5.26 | 21555 | 26.32 | stable | 80.75 | -0.154 |
| 64 | **P43982** | 8344.56 | 9.07 | 1615 | 39.09 | stable | 97.58 | -0.231 |
| 65 | **P44634** | 26383.47 | 4.57 | 18700 | 26.26 | stable | 84.15 | -0.327 |
| 66 | **P43984** | 19724.58 | 9.57 | 20525 | 47.95 | unstable | 131.10 | 0.642 |
| 67 | **P44640** | 47439.74 | 9.02 | 30035 | 37.85 | stable | 137.42 | 0.998 |
| 68 | **P43987** | 10449.92 | 9.40 | 4470 | 33.06 | stable | 89.66 | -0.636 |
| 69 | **P44641** | 38675.82 | 6.19 | 30160 | 47.67 | unstable | 105.92 | -0.179 |
| 70 | **P44646** | 38163.02 | 9.35 | 29575 | 34.40 | stable | 143.65 | 0.979 |
| 71 | **P44649** | 13385.06 | 4.84 | 28085 | 25.57 | stable | 82.02 | -0.534 |
| 72 | **P24324** | 27278.55 | 8.70 | 42415 | 48.39 | unstable | 97.92 | -0.343 |
| 73 | **Q57065** | 34011.30 | 5.24 | 44920 | 53.40 | unstable | 88.45 | -0.431 |
| 74 | **P43989** | 22664.42 | 5.02 | 27960 | 40.11 | unstable | 84.80 | -0.432 |
| 75 | **P44668** | 7711.61 | 4.20 | 17990 | 32.02 | stable | 88.44 | -0.728 |
| 76 | **P44670** | 26195.41 | 5.12 | 60390 | 38.70 | stable | 77.85 | -0.423 |
| 77 | **P44672** | 11722.34 | 5.09 | 7575 | 17.64 | stable | 94.58 | -0.111 |
| 78 | **P44675** | 16613.08 | 7.75 | 10095 | 49.57 | unstable | 107.20 | -0.183 |
| 79 | **P44676** | 27241.38 | 8.25 | 16640 | 43.87 | unstable | 101.58 | -0.210 |
| 80 | **P44679** | 15597.20 | 6.90 | 9065 | 26.02 | stable | 96.03 | 0.036 |
| 81 | **P43990** | 26083.68 | 5.06 | 25565 | 29.51 | stable | 97.63 | -0.062 |
| 82 | **P43992** | 21053.18 | 9.08 | 26360 | 35.46 | stable | 79.29 | -0.491 |
| 83 | **P43994** | 11584.30 | 7.93 | 5960 | 48.84 | unstable | 94.80 | -0.436 |
| 84 | **P44683** | 46419.24 | 4.37 | 82765 | 50.64 | unstable | 89.28 | -0.365 |
| 85 | **P44684** | 24580.00 | 5.52 | 40575 | 57.04 | unstable | 93.92 | -0.312 |
| 86 | **P44686** | 10342.60 | 4.45 | 1865 | 28.10 | stable | 79.05 | -0.096 |
| 87 | **P44691** | 28465.51 | 8.60 | 32555 | 31.08 | stable | 150.92 | 1.232 |
| 88 | **P44693** | 53255.26 | 9.11 | 48820 | 32.87 | stable | 86.82 | -0.553 |
| 89 | **Q57392** | 7917.90 | 9.22 | 11460 | 28.67 | stable | 71.00 | -0.473 |
| 90 | **P43995** | 11054.71 | 6.57 | 5960 | 22.22 | stable | 101.52 | -0.254 |
| 91 | **P44702** | 26562.53 | 6.43 | 31315 | 36.26 | stable | 87.93 | -0.268 |
| 92 | **P44709** | 22721.06 | 4.73 | 28210 | 37.40 | stable | 108.47 | -0.214 |
| 93 | **P31777** | 32217.09 | 8.37 | 49850 | 37.78 | stable | 91.92 | -0.427 |
| 94 | **P44711** | 11968.90 | 4.99 | 0 | 58.88 | unstable | 74.40 | -0.373 |
| 95 | **P43997** | 17325.35 | 5.70 | 33015 | 26.07 | stable | 80.06 | -0.292 |
| 96 | **P43998** | 7047.02 | 4.25 | 2980 | 40.66 | unstable | 105.08 | -0.062 |
| 97 | **P44717** | 48707.68 | 4.71 | 38975 | 35.33 | stable | 115.46 | 0.328 |
| 98 | **P43999** | 20416.35 | 7.85 | 38390 | 19.26 | stable | 80.57 | -0.156 |
| 99 | **P44718** | 29418.66 | 5.31 | 18255 | 34.14 | stable | 92.31 | -0.194 |
| 100 | **P44720** | 39620.66 | 8.59 | 51340 | 35.68 | stable | 93.89 | -0.444 |
| 101 | **Q57144** | 34845.90 | 9.16 | 53080 | 51.09 | unstable | 77.77 | -0.640 |
| 102 | **P44000** | 31549.10 | 5.01 | 32555 | 39.56 | stable | 98.57 | -0.175 |
| 103 | **P44726** | 33269.98 | 5.02 | 26470 | 42.09 | unstable | 99.93 | -0.504 |
| 104 | **P44003** | 14737.36 | 8.66 | 15595 | 59.90 | unstable | 112.32 | -0.022 |
| 105 | **P44005** | 17491.92 | 9.39 | 33585 | 29.94 | stable | 124.90 | 0.959 |
| 106 | **O05023** | 13997.21 | 10.15 | 13075 | 32.62 | stable | 60.34 | -0.844 |
| 107 | **P44733** | 51256.78 | 5.62 | 29005 | 41.04 | unstable | 89.31 | -0.596 |
| 108 | **P44010** | 20813.71 | 9.05 | 33920 | 39.72 | stable | 120.33 | 0.372 |
| 109 | **P44740** | 27214.22 | 8.77 | 27055 | 47.44 | stable | 88.45 | -0.441 |
| 110 | **P44743** | 29496.32 | 8.42 | 12295 | 37.81 | stable | 107.86 | 0.020 |
| 111 | **P44744** | 58159.12 | 5.33 | 53470 | 32.03 | stable | 87.90 | -0.306 |
| 112 | **Q57256** | 23520.47 | 9.92 | 33710 | 23.77 | stable | 139.95 | 1.007 |
| 113 | **P44012** | 28805.59 | 8.96 | 55265 | 48.83 | unstable | 69.60 | -0.636 |
| 114 | **P44013** | 24938.27 | 5.68 | 56295 | 48.99 | unstable | 78.26 | -0.467 |
| 115 | **P44014** | 21604.85 | 8.93 | 38640 | 58.62 | unstable | 86.61 | -0.503 |
| 116 | **Q57409** | 9646.10 | 5.55 | 20065 | 66.43 | unstable | 92.53 | 0.015 |
| 117 | **O86226** | 12826.20 | 4.93 | 26930 | 37.41 | stable | 132.35 | 1.036 |
| 118 | **P44016** | 66616.07 | 6.40 | 80120 | 25.77 | stable | 126.21 | 0.892 |
| 119 | **P44754** | 15504.91 | 10.03 | 20970 | 54.25 | unstable | 73.05 | -1.045 |
| 120 | **P44759** | 8722.90 | 4.63 | 2980 | 84.17 | unstable | 82.88 | -0.738 |
| 121 | **P44761** | 24850.30 | 5.70 | 9190 | 38.92 | stable | 104.52 | -0.160 |
| 122 | **P44017** | 10901.68 | 4.73 | 16055 | 57.14 | unstable | 111.89 | 0.038 |
| 123 | **P44018** | 28990.26 | 5.94 | 21430 | 33.40 | stable | 118.57 | 0.783 |
| 124 | **P44019** | 15225.54 | 9.51 | 4595 | 16.84 | stable | 127.93 | 1.101 |
| 125 | **P44023** | 55486.78 | 5.86 | 64205 | 31.10 | stable | 115.05 | 0.726 |
| 126 | **P44771** | 30522.81 | 5.02 | 24660 | 28.45 | stable | 88.49 | -0.254 |
| 127 | **P44782** | 25138.01 | 8.84 | 23170 | 40.52 | unstable | 84.16 | -0.414 |
| 128 | **P44025** | 10430.35 | 7.85 | 15470 | 40.00 | stable | 104.35 | -0.169 |
| 129 | **P44026** | 10921.90 | 6.03 | 12615 | 64.81 | unstable | 101.53 | 0.471 |
| 130 | **P44027** | 10966.41 | 4.19 | 4845 | 46.01 | unstable | 76.25 | -0.168 |
| 131 | **P44796** | 23234.98 | 9.62 | 26930 | 53.41 | unstable | 110.44 | -0.138 |
| 132 | **P44028** | 7763.27 | 4.23 | 16960 | 26.03 | stable | 136.57 | 0.876 |
| 133 | **P44807** | 20487.33 | 6.31 | 8730 | 39.56 | stable | 83.66 | -0.341 |
| 134 | **P46494** | 20304.46 | 8.44 | 9940 | 76.05 | unstable | 61.35 | -0.553 |
| 135 | **P44031** | 14321.67 | 9.58 | 12950 | 34.97 | stable | 96.64 | -0.733 |
| 136 | **P44033** | 39832.46 | 6.03 | 23965 | 46.89 | unstable | 86.73 | -0.505 |
| 137 | **P44034** | 12453.12 | 5.65 | 12950 | 43.78 | unstable | 80.00 | -0.397 |
| 138 | **O86228** | 11464.32 | 8.82 | 2980 | 30.94 | stable | 107.06 | -0.108 |
| 139 | **P44812** | 8626.66 | 4.74 | 5500 | 76.21 | unstable | 101.53 | -0.996 |
| 140 | **P44036** | 17192.71 | 5.45 | 18450 | 30.86 | stable | 92.95 | -0.196 |
| 141 | **P71356** | 33886.93 | 9.42 | 15150 | 27.55 | stable | 134.38 | 0.920 |
| 142 | **P44037** | 11730.25 | 5.86 | 7575 | 28.22 | stable | 93.79 | -0.564 |
| 143 | **P44827** | 27463.95 | 10.12 | 19480 | 25.12 | stable | 91.38 | -0.488 |
| 144 | **Q57523** | 141222.83 | 6.34 | 100060 | 33.83 | stable | 99.68 | -0.228 |
| 145 | **P44038** | 63401.42 | 9.47 | 121700 | 31.58 | stable | 79.64 | -0.480 |
| 146 | **P44831** | 17591.85 | 3.91 | 14440 | 40.99 | unstable | 78.50 | -0.767 |
| 147 | **P44040** | 6302.40 | 4.94 | 0 | 27.88 | stable | 94.26 | 0.262 |
| 148 | **P71357** | 11015.37 | 4.73 | 5960 | 46.75 | unstable | 84.69 | -0.300 |
| 149 | **P44041** | 11907.69 | 6.89 | 14565 | 51.88 | unstable | 101.27 | -0.382 |
| 150 | **P44839** | 14024.13 | 5.35 | 2980 | 31.39 | stable | 102.08 | 0.048 |
| 151 | **P44842** | 22710.00 | 5.60 | 21555 | 43.06 | unstable | 100.39 | -0.111 |
| 152 | **P44844** | 21537.78 | 8.86 | 3105 | 42.00 | unstable | 80.75 | -1.008 |
| 153 | **P44043** | 17054.31 | 9.41 | 11460 | 38.85 | stable | 120.27 | -0.003 |
| 154 | **P44045** | 5491.13 | 4.36 | Not visible | 49.67 | unstable | 88.00 | -0.298 |
| 155 | **P44047** | 11897.60 | 4.78 | 24325 | 20.85 | stable | 98.53 | 0.197 |
| 156 | **P44854** | 16537.19 | 8.89 | 13980 | 29.63 | stable | 107.36 | 0.184 |
| 157 | **P44863** | 27845.23 | 9.37 | 12950 | 47.94 | unstable | 98.68 | -0.013 |
| 158 | **P44864** | 45983.70 | 9.93 | 25900 | 43.21 | unstable | 90.22 | -0.635 |
| 159 | **P44048** | 10582.25 | 6.11 | 15470 | 30.72 | stable | 75.78 | -0.556 |
| 160 | **P44050** | 26079.67 | 6.25 | 39545 | 43.57 | unstable | 82.83 | -0.404 |
| 161 | **P44869** | 22001.33 | 8.70 | 28085 | 32.16 | stable | 90.93 | -0.412 |
| 162 | **P44052** | 23814.25 | 5.09 | 29340 | 45.49 | unstable | 100.85 | -0.291 |
| 163 | **P44053** | 15008.18 | 6.41 | 13535 | 27.35 | stable | 80.93 | -0.319 |
| 164 | **P44054** | 29137.30 | 9.32 | 17795 | 35.38 | stable | 132.05 | 1.082 |
| 165 | **P44882** | 20290.44 | 3.96 | 20065 | 39.67 | stable | 96.48 | -0.223 |
| 166 | **P44056** | 27745.10 | 9.82 | 30940 | 30.60 | stable | 128.24 | 0.794 |
| 167 | **P44886** | 16763.42 | 8.37 | 14230 | 28.06 | stable | 92.99 | 0.009 |
| 168 | **P44897** | 7683.85 | 6.70 | 1490 | 20.34 | stable | 105.83 | 0.151 |
| 169 | **P44898** | 67737.57 | 8.80 | 111270 | 32.47 | stable | 89.21 | -0.208 |
| 170 | **P44058** | 28207.49 | 6.18 | 37150 | 32.96 | stable | 82.36 | -0.350 |
| 171 | **P44059** | 4965.88 | 4.64 | 5625 | 32.42 | stable | 130.00 | 0.498 |
| 172 | **P44900** | 10281.93 | 5.16 | 11460 | 34.36 | stable | 98.64 | -0.534 |
| 173 | **P31811** | 12805.10 | 6.60 | 14440 | 28.94 | stable | 43.15 | -0.954 |
| 174 | **P44903** | 50002.52 | 9.45 | 86650 | 40.39 | unstable | 125.85 | 0.822 |
| 175 | **P44904** | 12247.74 | 9.03 | 1615 | 38.66 | stable | 130.61 | 0.458 |
| 176 | **P44062** | 11422.38 | 5.69 | Not visible | 58.19 | unstable | 135.40 | -0.074 |
| 177 | **P44905** | 21821.18 | 9.21 | 30940 | 50.85 | unstable | 95.40 | -0.361 |
| 178 | **P44908** | 26888.75 | 8.89 | 36440 | 32.92 | stable | 111.62 | 0.773 |
| 179 | **P44063** | 23656.16 | 7.72 | 17880 | 44.17 | unstable | 102.22 | -0.246 |
| 180 | **Q57022** | 28915.59 | 8.83 | 26610 | 42.90 | unstable | 101.00 | -0.099 |
| 181 | **P44064** | 20857.83 | 5.36 | 10430 | 33.92 | stable | 103.89 | -0.136 |
| 182 | **P44065** | 4289.96 | 9.99 | 4470 | 55.44 | unstable | 51.47 | -1.088 |
| 183 | **P44067** | 45281.22 | 9.71 | 39100 | 38.34 | stable | 124.94 | 0.620 |
| 184 | **P71360** | 33892.71 | 9.09 | 45755 | 35.70 | stable | 127.84 | 0.861 |
| 185 | **P44068** | 29072.18 | 8.62 | 52870 | 47.64 | unstable | 61.39 | -0.807 |
| 186 | **P44069** | 14753.63 | 8.73 | 10430 | 25.46 | stable | 117.99 | 0.913 |
| 187 | **P44070** | 27542.37 | 9.79 | 18700 | 30.06 | stable | 132.23 | 1.127 |
| 188 | **P44931** | 19359.24 | 6.91 | 13075 | 43.59 | unstable | 90.87 | -0.240 |
| 189 | **P44072** | 12029.88 | 8.03 | 1490 | 45.44 | unstable | 91.92 | -0.280 |
| 190 | **P44073** | 11175.85 | 9.81 | 6990 | 42.71 | unstable | 101.50 | -0.115 |
| 191 | **P44074** | 29739.02 | 8.60 | 28545 | 44.59 | unstable | 80.67 | -0.669 |
| 192 | **P44936** | 49070.30 | 7.69 | 59025 | 35.79 | stable | 107.18 | 0.195 |
| 193 | **P44938** | 27343.28 | 8.98 | 36565 | 46.23 | unstable | 89.04 | -0.380 |
| 194 | **P44075** | 14588.62 | 5.88 | 15930 | 29.00 | stable | 86.20 | -0.712 |
| 195 | **P44076** | 9564.05 | 4.43 | 4470 | 36.02 | stable | 108.84 | 0.119 |
| 196 | **P44940** | 45792.35 | 4.99 | 100395 | 38.98 | stable | 72.24 | -0.489 |
| 197 | **P44077** | 21841.18 | 8.77 | 10095 | 45.91 | unstable | 66.36 | -0.700 |
| 198 | **P44078** | 18798.46 | 6.04 | 29910 | 27.23 | stable | 81.12 | -0.324 |
| 199 | **P44941** | 44476.02 | 6.21 | 63745 | 34.71 | stable | 94.06 | -0.045 |
| 200 | **P44079** | 19835.07 | 9.10 | 25815 | 42.81 | unstable | 95.82 | -0.085 |
| 201 | **P44080** | 26787.17 | 9.24 | 25815 | 43.12 | unstable | 97.94 | -0.199 |
| 202 | **P44081** | 25484.32 | 8.95 | 31065 | 33.01 | stable | 94.54 | -0.189 |
| 203 | **P44082** | 9035.36 | 9.51 | 8605 | 28.80 | stable | 101.39 | 0.130 |
| 204 | **Q57120** | 8880.98 | 4.68 | 5500 | 54.23 | unstable | 81.04 | -0.368 |
| 205 | **P44954** | 9716.13 | 4.50 | 12615 | 54.82 | unstable | 111.57 | -0.372 |
| 206 | **P44084** | 11741.29 | 9.16 | 22920 | 46.30 | unstable | 96.64 | -0.329 |
| 207 | **P44085** | 21442.49 | 8.32 | 24535 | 31.89 | stable | 78.40 | -0.405 |
| 208 | **P44086** | 4089.51 | 8.25 | 1490 | 24.97 | stable | 40.29 | -0.944 |
| 209 | **Q57133** | 30603.82 | 6.90 | 21555 | 26.41 | stable | 72.87 | -0.474 |
| 210 | **P46455** | 9817.63 | 6.52 | 28085 | 30.03 | stable | 121.65 | 0.398 |
| 211 | **Q57147** | 15013.26 | 10.20 | 40450 | 27.54 | stable | 124.06 | 0.775 |
| 212 | **O86230** | 17972.41 | 8.66 | 32095 | 22.90 | stable | 144.00 | 1.022 |
| 213 | **P44965** | 36764.51 | 9.03 | 35785 | 34.89 | stable | 97.30 | -0.145 |
| 214 | **P43907** | 22749.02 | 9.30 | 30035 | 36.41 | stable | 85.80 | -0.614 |
| 215 | **P43908** | 29254.35 | 5.61 | 33015 | 36.93 | stable | 88.88 | -0.293 |
| 216 | **P44972** | 9564.42 | 9.70 | 8730 | 38.39 | stable | 96.40 | -0.053 |
| 217 | **P44974** | 60430.51 | 5.91 | 103265 | 35.59 | stable | 89.79 | -0.035 |
| 218 | **Q57134** | 9832.48 | 7.86 | 1490 | 16.78 | stable | 94.11 | -0.034 |
| 219 | **P44093** | 44786.95 | 5.13 | 37610 | 25.82 | stable | 87.63 | -0.025 |
| 220 | **Q57151** | 29348.26 | 5.15 | 40465 | 20.34 | stable | 82.40 | -0.390 |
| 221 | **P44094** | 35145.31 | 5.30 | 25690 | 41.05 | unstable | 100.89 | -0.022 |
| 222 | **P44095** | 15926.22 | 5.27 | 21805 | 32.43 | stable | 81.06 | -0.024 |
| 223 | **P44992** | 34133.40 | 9.21 | 21430 | 28.44 | stable | 94.54 | -0.269 |
| 224 | **P44993** | 45467.64 | 8.31 | 31190 | 36.92 | stable | 141.11 | 1.196 |
| 225 | **P44994** | 18069.80 | 8.54 | 17085 | 34.35 | stable | 140.50 | 1.056 |
| 226 | **P44097** | 20200.58 | 9.79 | 25440 | 32.32 | stable | 148.62 | 1.046 |
| 227 | **P44098** | 33181.93 | 7.68 | 32805 | 27.47 | stable | 79.32 | -0.231 |
| 228 | **P44099** | 46102.82 | 6.05 | 50770 | 36.36 | stable | 104.27 | -0.215 |
| 229 | **P44103** | 39280.44 | 6.72 | 60070 | 21.79 | stable | 75.61 | -0.424 |
| 230 | **Q57498** | 11862.48 | 6.28 | 13075 | 20.56 | stable | 87.61 | 0.058 |
| 231 | **P44104** | 53471.73 | 6.19 | 47900 | 40.24 | unstable | 76.59 | -0.752 |
| 232 | **P44106** | 19381.67 | 6.08 | 9970 | 63.87 | unstable | 91.29 | -0.365 |
| 233 | **P44107** | 8285.30 | 5.77 | 8480 | 18.86 | stable | 68.29 | -1.144 |
| 234 | **P71367** | 55401.14 | 9.01 | 66615 | 35.15 | stable | 92.72 | -0.026 |
| 235 | **P45019** | 13449.94 | 9.21 | 27960 | 22.66 | stable | 124.44 | 0.884 |
| 236 | **P44110** | 15479.12 | 9.60 | 12490 | 26.94 | stable | 174.80 | 1.413 |
| 237 | **P45026** | 9769.21 | 6.06 | 11460 | 45.36 | unstable | 98.71 | -0.229 |
| 238 | **P44111** | 5027.87 | 9.25 | 2980 | 33.80 | stable | 92.95 | -0.548 |
| 239 | **P44112** | 11601.40 | 9.65 | 10555 | 54.36 | unstable | 86.76 | -0.307 |
| 240 | **P45071** | 32564.35 | 7.22 | 29005 | 38.84 | stable | 104.07 | -0.269 |
| 241 | **P45074** | 16275.48 | 9.47 | 2980 | 28.69 | stable | 95.12 | -0.273 |
| 242 | **P45075** | 23819.09 | 7.70 | 31400 | 40.42 | unstable | 89.04 | -0.452 |
| 243 | **P45076** | 21034.13 | 5.89 | 16960 | 53.21 | unstable | 98.65 | -0.878 |
| 244 | **P45077** | 48497.65 | 5.05 | 41495 | 35.47 | stable | 93.68 | -0.132 |
| 245 | **P45083** | 15051.39 | 6.40 | 16750 | 43.16 | unstable | 108.04 | 0.123 |
| 246 | **P44116** | 18541.50 | 8.97 | 27055 | 56.06 | unstable | 98.09 | -0.388 |
| 247 | **Q57252** | 116743.37 | 8.27 | 103930 | 37.18 | stable | 92.01 | -0.310 |
| 248 | **P45085** | 11940.95 | 5.25 | 17085 | 59.40 | unstable | 96.73 | -0.016 |
| 249 | **P44117** | 13866.81 | 9.33 | 7450 | 41.07 | unstable | 79.56 | -1.182 |
| 250 | **P44119** | 18683.81 | 9.19 | 14690 | 34.45 | stable | 83.08 | -0.517 |
| 251 | **P45097** | 24337.16 | 6.83 | 30285 | 52.96 | unstable | 86.40 | -0.281 |
| 252 | **P44124** | 25659.45 | 6.59 | 20775 | 34.47 | stable | 87.32 | -0.290 |
| 253 | **P44125** | 3284.51 | 8.98 | 12490 | 27.07 | stable | 89.36 | -0.136 |
| 254 | **P45103** | 23092.49 | 5.87 | 10555 | 50.61 | unstable | 96.09 | -0.229 |
| 255 | **P45104** | 40636.68 | 10.24 | 34045 | 42.13 | unstable | 78.63 | -0.750 |
| 256 | **P44126** | 19393.19 | 5.77 | 17085 | 40.55 | unstable | 91.14 | -0.566 |
| 257 | **P44127** | 17270.37 | 9.24 | 34950 | 43.92 | unstable | 97.69 | 0.147 |
| 258 | **P71373** | 33371.58 | 9.29 | 36690 | 41.58 | unstable | 96.28 | -0.183 |
| 259 | **P44129** | 10915.22 | 9.90 | 9970 | 27.72 | stable | 152.78 | 0.931 |
| 260 | **P44131** | 15218.60 | 5.85 | 2980 | 26.50 | stable | 106.90 | -0.085 |
| 261 | **P44132** | 41355.06 | 6.76 | 60515 | 40.52 | unstable | 94.90 | -0.250 |
| 262 | **P45122** | 24227.78 | 6.58 | 26025 | 27.05 | stable | 123.68 | 0.738 |
| 263 | **P44133** | 29650.89 | 8.61 | 39670 | 41.23 | unstable | 142.97 | 1.109 |
| 264 | **P44134** | 24637.21 | 9.25 | 32890 | 45.62 | unstable | 70.97 | -0.544 |
| 265 | **P44135** | 72751.73 | 8.61 | 103710 | 39.45 | stable | 89.85 | -0.038 |
| 266 | **P44136** | 35669.31 | 10.01 | 56630 | 23.11 | stable | 116.96 | 0.484 |
| 267 | **P44137** | 21484.27 | 6.08 | 26485 | 36.79 | stable | 83.35 | -0.360 |
| 268 | **P44138** | 12248.89 | 7.83 | 14900 | 35.80 | stable | 84.95 | -0.805 |
| 269 | **P44139** | 14575.95 | 10.15 | 26470 | 21.70 | stable | 145.12 | 1.227 |
| 270 | **P44140** | 75161.88 | 6.15 | 79800 | 47.70 | unstable | 91.37 | -0.291 |
| 271 | **Same as 270** | - | - | - | - | - | - | - |
| 272 | **Same as 270** | - | - | - | - | - | - | - |
| 273 | **P44144** | 66529.21 | 4.54 | 107970 | 36.73 | stable | 85.45 | -0.329 |
| 274 | **P44145** | 15106.79 | 9.46 | 11460 | 28.68 | stable | 113.59 | 0.156 |
| 275 | **No result** | - | - | - | - | - | - | - |
| 276 | **P44148** | 4562.33 | 8.12 | 2980 | 30.62 | stable | 84.74 | -0.297 |
| 277 | **P44150** | 30510.64 | 5.27 | 49640 | 32.95 | stable | 87.01 | -0.318 |
| 278 | **P45138** | 17266.85 | 4.55 | 14105 | 47.02 | unstable | 103.84 | -0.152 |
| 279 | **P44154** | 29818.42 | 4.91 | 49890 | 51.19 | unstable | 80.04 | -0.450 |
| 280 | **P44156** | 14642.87 | 6.25 | 18115 | 25.42 | stable | 87.54 | -0.360 |
| 281 | **P45145** | 15958.53 | 10.17 | 21430 | 43.82 | unstable | 154.43 | 0.883 |
| 282 | **P45146** | 24885.98 | 9.39 | 30035 | 35.93 | stable | 137.58 | 1.002 |
| 283 | **Q57320** | 23636.28 | 9.28 | 27515 | 34.85 | stable | 122.95 | 0.820 |
| 284 | **P45154** | 9588.08 | 6.27 | 4845 | 48.78 | unstable | 92.68 | -0.389 |
| 285 | **P44158** | 29371.14 | 9.26 | 42650 | 31.95 | stable | 81.78 | -0.247 |
| 286 | **P71375** | 11403.60 | 7.74 | 24075 | 14.53 | stable | 127.05 | 0.893 |
| 287 | **P44160** | 30802.28 | 6.75 | 43805 | 48.40 | unstable | 83.06 | -0.344 |
| 288 | **P44161** | 17996.80 | 9.70 | 32430 | 52.04 | unstable | 74.59 | -1.053 |
| 289 | **P44162** | 29169.01 | 9.06 | 58120 | 28.81 | stable | 95.83 | -0.290 |
| 290 | **P44163** | 4276.10 | 10.06 | 0 | 24.85 | stable | 104.00 | 1.122 |
| 291 | **P71376** | 10923.81 | 9.63 | 1490 | 29.48 | stable | 115.25 | -0.128 |
| 292 | **P44164** | 18700.55 | 6.04 | 26930 | 41.81 | unstable | 107.56 | -0.035 |
| 293 | **P71378** | 13778.74 | 5.41 | 0 | 22.36 | stable | 74.96 | -0.502 |
| 294 | **P44165** | 49789.84 | 6.55 | 74050 | 34.38 | stable | 86.08 | -0.448 |
| 295 | **P71379** | 26720.06 | 9.68 | 41480 | 30.48 | stable | 108.19 | 0.041 |
| 296 | **P45173** | 18167.54 | 5.20 | 23045 | 39.43 | stable | 89.69 | -0.295 |
| 297 | **P44167** | 36854.39 | 6.13 | 61670 | 30.17 | stable | 93.49 | -0.295 |
| 298 | **P44168** | 18162.70 | 5.96 | 38430 | 29.92 | stable | 72.21 | -0.647 |
| 299 | **P45180** | 94441.36 | 5.64 | 137420 | 38.71 | stable | 88.53 | -0.352 |
| 300 | **P45182** | 96410.83 | 8.81 | 147155 | 39.60 | stable | 78.43 | -0.550 |
| 301 | **P44169** | 35503.47 | 5.52 | 39880 | 36.49 | stable | 95.56 | -0.534 |
| 302 | **P44170** | 31916.37 | 9.45 | 28670 | 34.12 | stable | 133.75 | 1.037 |
| 303 | **P44171** | 17004.33 | 7.80 | 28420 | 37.90 | stable | 92.23 | -0.309 |
| 304 | **O86237** | 14961.39 | 6.42 | 16055 | 38.53 | stable | 92.97 | -0.307 |
| 305 | **P44172** | 14517.45 | 4.82 | 11585 | 39.40 | stable | 90.33 | -0.402 |
| 306 | **P44173** | 15866.42 | 10.00 | 12950 | 24.33 | stable | 91.29 | -0.226 |
| 307 | **P44175** | 23238.34 | 4.83 | 33920 | 38.99 | stable | 74.90 | -0.388 |
| 308 | **P44176** | 30116.40 | 6.21 | 49180 | 27.12 | stable | 88.72 | -0.213 |
| 309 | **P44177** | 7841.06 | 4.61 | 5500 | 59.14 | unstable | 108.97 | 0.004 |
| 310 | **P44180** | 40122.21 | 5.11 | 18575 | 30.37 | stable | 77.32 | -0.634 |
| 311 | **P44181** | 13325.34 | 6.08 | 19480 | 41.93 | unstable | 112.46 | -0.081 |
| 312 | **P44183** | 48921.03 | 4.62 | 56045 | 41.36 | unstable | 98.42 | -0.299 |
| 313 | **P45197** | 20511.96 | 9.02 | 18575 | 39.10 | stable | 80.06 | -0.484 |
| 314 | **P44185** | 10360.15 | 9.16 | 11710 | 28.78 | stable | 99.57 | -0.032 |
| 315 | **P44186** | 10342.81 | 5.29 | 4470 | 52.34 | unstable | 109.23 | -0.237 |
| 316 | **P44187** | 22895.25 | 9.18 | 42650 | 22.58 | stable | 76.60 | -0.494 |
| 317 | **P44188** | 10458.27 | 7.80 | 25105 | 40.03 | unstable | 93.47 | 0.223 |
| 318 | **P44189** | 23567.17 | 9.11 | 37025 | 44.95 | unstable | 72.29 | -0.475 |
| 319 | **P44190** | 11169.05 | 9.76 | 8480 | 15.45 | stable | 107.37 | -0.288 |
| 320 | **P44191** | 10889.38 | 4.72 | 2980 | 62.13 | unstable | 98.66 | -0.202 |
| 321 | **P44193** | 22701.06 | 7.06 | 30035 | 36.90 | stable | 86.28 | -0.613 |
| 322 | **P44194** | 10657.30 | 9.57 | 11460 | 42.96 | unstable | 93.40 | -0.366 |
| 323 | **P44196** | 28967.15 | 8.33 | 42525 | 27.45 | stable | 85.97 | -0.346 |
| 324 | **P45202** | 17154.82 | 8.56 | 4470 | 21.41 | stable | 94.49 | -0.235 |
| 325 | **P56507** | 5902.69 | 5.52 | Not visible | 48.11 | unstable | 88.27 | -0.700 |
| 326 | **P44197** | 27776.53 | 6.67 | 31525 | 29.61 | stable | 88.91 | -0.381 |
| 327 | **Q57152** | 12274.26 | 4.26 | 17990 | 70.91 | unstable | 91.23 | -0.279 |
| 328 | **P44198** | 10085.79 | 8.58 | 6085 | 36.22 | stable | 99.66 | -0.116 |
| 329 | **P44201** | 21241.26 | 5.53 | 26595 | 33.16 | stable | 127.49 | 0.903 |
| 330 | **P44202** | 22789.50 | 9.62 | 10095 | 17.78 | stable | 136.85 | 1.055 |
| 331 | **P44203** | 18751.55 | 8.85 | 17420 | 36.59 | stable | 87.08 | -0.077 |
| 332 | **P45217** | 50289.49 | 8.93 | 78270 | 30.50 | stable | 89.47 | -0.276 |
| 333 | **Same as 293** | - | - | - | - | - | - | - |
| 334 | **Same as 292** | - | - | - | - | - | - | - |
| 335 | **P44205** | 13064.08 | 8.64 | 4595 | 47.95 | unstable | 101.83 | 0.026 |
| 336 | **Q57380** | 13271.84 | 9.25 | 21095 | 37.86 | stable | 109.08 | 0.687 |
| 337 | **P44208** | 10467.19 | 9.63 | 18450 | 41.58 | unstable | 71.78 | -0.477 |
| 338 | **P44209** | 17735.52 | 6.65 | 22460 | 53.06 | unstable | 108.78 | -0.003 |
| 339 | **P44210** | 11985.18 | 9.17 | 15595 | 26.12 | stable | 93.71 | -0.107 |
| 340 | **P44212** | 8833.83 | 7.91 | 15470 | 61.49 | unstable | 32.33 | -1.400 |
| 341 | **P44213** | 6587.75 | 9.40 | 1490 | -3.29 | stable | 77.02 | -0.388 |
| 342 | **P44214** | 21636.84 | 6.62 | 27515 | 25.03 | stable | 84.35 | -0.396 |
| 343 | **P44215** | 20782.00 | 8.92 | 14690 | 42.28 | unstable | 96.52 | -0.398 |
| 344 | **P44217** | 17028.98 | 5.00 | 19035 | 27.08 | stable | 79.58 | -0.415 |
| 345 | **P44218** | 10506.30 | 11.53 | 22000 | 36.56 | stable | 85.11 | -0.261 |
| 346 | **P44219** | 9463.64 | 5.29 | 9970 | 29.65 | stable | 66.98 | -0.193 |
| 347 | **P44220** | 9773.23 | 9.69 | 5960 | 63.51 | unstable | 89.40 | -0.748 |
| 348 | **P44221** | 8405.30 | 4.29 | 5750 | 56.24 | unstable | 84.67 | -0.547 |
| 349 | **P44222** | 15625.14 | 8.95 | 19605 | 36.72 | stable | 101.80 | -0.080 |
| 350 | **O86242** | 11648.23 | 6.08 | 15595 | 61.08 | unstable | 96.47 | -0.423 |
| 351 | **P44223** | 21016.07 | 5.74 | 6990 | 36.37 | stable | 93.54 | -0.419 |
| 352 | **P44224** | 57190.96 | 5.28 | 89435 | 34.64 | stable | 82.13 | -0.323 |
| 353 | **P44225** | 58369.18 | 4.99 | 68995 | 44.68 | Unstable | 87.54 | -0.371 |
| 354 | **P44226** | 46494.63 | 8.55 | 82975 | 30.40 | stable | 83.91 | -0.533 |
| 355 | **P44227** | 33811.20 | 5.38 | 49515 | 30.03 | stable | 82.37 | -0.288 |
| 356 | **P44228** | 13841.45 | 5.04 | 1490 | 38.15 | stable | 75.55 | -0.512 |
| 357 | **Same as 356** | - | - | - | - | - | - | - |
| 358 | **P44230** | 15872.88 | 4.77 | 21555 | 32.28 | stable | 89.29 | -0.344 |
| 359 | **P44231** | 21816.67 | 5.47 | 34045 | 34.65 | stable | 83.87 | -0.334 |
| 360 | **P44232** | 7161.15 | 6.56 | 6990 | 25.05 | stable | 74.29 | -0.851 |
| 361 | **P44234** | 12910.60 | 4.74 | 14105 | 27.67 | stable | 71.86 | -0.345 |
| 362 | **P44235** | 13977.98 | 4.72 | 8480 | 34.09 | stable | 93.78 | -0.206 |
| 363 | **P44238** | 19774.28 | 5.95 | 6085 | 50.54 | unstable | 95.27 | -0.232 |
| 364 | **P44239** | 14964.85 | 4.16 | 35980 | 42.41 | unstable | 109.78 | -0.012 |
| 365 | **P44240** | 38451.87 | 5.49 | 41620 | 33.72 | stable | 97.04 | 0.071 |
| 366 | **P44241** | 19819.71 | 6.31 | 24785 | 49.39 | unstable | 85.24 | -0.443 |
| 367 | **P44242** | 67677.80 | 6.00 | 74760 | 28.62 | stable | 77.67 | -0.281 |
| 368 | **P71390** | 4449.48 | 9.77 | 1740 | 21.17 | stable | 94.87 | 0.195 |
| 369 | **P44243** | 34485.55 | 8.15 | 47580 | 38.88 | stable | 87.64 | -0.397 |
| 370 | **P44246** | 75691.18 | 6.51 | 106060 | 36.69 | stable | 86.09 | -0.278 |
| 371 | **Same as 370** | - | - | - | - | - | - | - |
| 372 | **P44247** | 12778.70 | 9.57 | 15470 | 37.14 | stable | 99.30 | -0.167 |
| 373 | **P45244** | 20399.69 | 6.11 | 21095 | 20.47 | stable | 92.34 | -0.102 |
| 374 | **P44251** | 25200.61 | 5.90 | 59150 | 39.00 | stable | 85.81 | -0.288 |
| 375 | **P44252** | 43578.88 | 7.81 | 31860 | 23.07 | stable | 114.07 | 0.295 |
| 376 | **P45252** | 31231.72 | 5.50 | 43445 | 46.07 | unstable | 87.00 | -0.363 |
| 377 | **P45253** | 33054.66 | 5.06 | 41940 | 38.97 | stable | 105.58 | -0.270 |
| 378 | **P44253** | 17581.75 | 9.69 | 29450 | 40.02 | unstable | 106.28 | 0.322 |
| 379 | **P44254** | 6034.92 | 8.95 | 3105 | 37.94 | stable | 61.92 | -1.310 |
| 380 | **P44255** | 20604.12 | 4.93 | 33015 | 37.38 | stable | 75.73 | -0.544 |
| 381 | **P44256** | 5836.87 | 8.28 | 5500 | 77.49 | unstable | 101.40 | -0.324 |
| 382 | **P44260** | 9048.37 | 4.53 | 15470 | 42.74 | unstable | 108.61 | -0.089 |
| 383 | **Q4QKT3** | 10670.58 | 9.60 | 10095 | 47.92 | unstable | 97.42 | -0.417 |
| 384 | **P44262** | 22252.21 | 8.43 | 25565 | 41.66 | unstable | 108.69 | -0.210 |
| 385 | **Same as 384** | - | - | - | - | - | - | - |
| 386 | **P45267** | 41315.29 | 5.15 | 37360 | 51.18 | unstable | 88.66 | -0.407 |
| 387 | **P44267** | 27533.17 | 4.59 | 46535 | 33.25 | stable | 92. 65 | -0.238 |
| 388 | **P44268** | 34801.13 | 5.00 | 34965 | 43.56 | unstable | 90.59 | -0.283 |
| 389 | **P44269** | 6934.74 | 8.93 | 500 | -2.98 | stable | 44.53 | -0.486 |
| 390 | **P44270** | 17217.34 | 7.75 | 40575 | 26.03 | stable | 116.23 | 0.517 |
| 391 | **P44272** | 20602.45 | 9.75 | 30940 | 46.01 | unstable | 96.60 | -0.502 |
| 392 | **P44275** | 14975.08 | 7.86 | 13410 | 20.55 | stable | 99.44 | 0.063 |
| 393 | **P44277** | 19037.09 | 7.77 | 32430 | 39.82 | stable | 82.24 | -0.269 |
| 394 | **P44278** | 27573.41 | 9.62 | 60850 | 22.76 | stable | 127.44 | 0.934 |
| 395 | **P71394** | 13000.93 | 5.25 | 20970 | 38.26 | stable | 108.53 | -0.056 |
| 396 | **P45279** | 15314.27 | 6.31 | 18450 | 17.56 | stable | 82.31 | -0.610 |
| 397 | **P45280** | 24550.37 | 9.49 | 41495 | 33.81 | stable | 122.22 | 0.590 |
| 398 | **P44279** | 19030.93 | 9.57 | 14900 | 39.62 | stable | 97.47 | -0.299 |
| 399 | **Q57525** | 12950.88 | 7.83 | 4595 | 32.08 | stable | 101.49 | 0.191 |
| 400 | **P44280** | 54250.89 | 8.96 | 53985 | 37.25 | stable | 90.04 | -0.364 |
| 401 | **P45290** | 36720.77 | 8.93 | 41495 | 26.36 | stable | 129.56 | 0.929 |
| 402 | **P44281** | 4284.69 | 8.12 | 9970 | 61.22 | unstable | 48.89 | -1.481 |
| 403 | **P44282** | 10747.51 | 8.74 | 1490 | 41.86 | unstable | 97.50 | -0.477 |
| 404 | **No result** | - | - | - | - | - | - | - |
| 405 | **P45298** | 31463.20 | 5.57 | 21680 | 27.43 | stable | 102.83 | -0.157 |
| 406 | **P45300** | 13811.75 | 6.09 | 23615 | 34.95 | stable | 84.54 | -0.339 |
| 407 | **P52606** | 21025.25 | 4.88 | 6210 | 44.63 | unstable | 117.11 | 0.306 |
| 408 | **P45301** | 17233.56 | 8.56 | 9970 | 9.62 | stable | 103.52 | 0.088 |
| 409 | **Q57544** | 23882.11 | 5.12 | 19480 | 38.52 | stable | 97.55 | -0.253 |
| 410 | **P45305** | 29334.72 | 6.73 | 39420 | 41.70 | unstable | 103.39 | -0.340 |
| 411 | **P44283** | 4363.02 | 9.82 | 8480 | 23.18 | stable | 77.75 | -0.235 |
| 412 | **P44284** | 14493.89 | 10.63 | 11460 | 53.86 | unstable | 85.28 | -0.420 |
| 413 | **P44285** | 55582.31 | 8.21 | 82865 | 41.46 | unstable | 96.54 | -0.322 |
| 414 | **P44287** | 47373.88 | 8.93 | 85215 | 45.46 | unstable | 117.21 | 0.691 |
| 415 | **P44288** | 96677.92 | 6.61 | 84230 | 33.86 | stable | 98.31 | -0.172 |
| 416 | **P44289** | 82373.92 | 8.81 | 86680 | 36.54 | stable | 110.56 | 0.213 |
| 417 | **P44290** | 21245.78 | 8.95 | 13980 | 26.40 | stable | 86.06 | -0.119 |
| 418 | **P44292** | 27612.21 | 8.45 | 43890 | 34.96 | stable | 108.34 | 0.143 |
| 419 | **P45332** | 39962.81 | 9.30 | 57410 | 24.90 | stable | 110.84 | 0.380 |
| 420 | **P45333** | 41336.83 | 9.12 | 40005 | 34.74 | stable | 121.94 | 0.405 |
| 421 | **P44293** | 11245.58 | 5.11 | 12490 | 24.89 | stable | 87.19 | -0.201 |
| 422 | **P44294** | 4838.59 | 5.06 | 2980 | 39.24 | stable | 108.26 | 0.257 |
| 423 | **P44296** | 27434.54 | 6.00 | 7450 | 18.28 | stable | 81.19 | -0.417 |
| 424 | **Q57066** | 21747.39 | 10.01 | 17085 | 45.08 | unstable | 82.50 | -0.589 |
| 425 | **P44297** | 13487.04 | 4.34 | 13075 | 49.03 | unstable | 88.38 | -0.122 |
| 426 | **O05087** | 41961.94 | 9.57 | 35410 | 26.14 | stable | 128.72 | 1.008 |
| 427 | **P44298** | 34561.90 | 8.90 | 38640 | 47.19 | unstable | 95.08 | -0.153 |
| 428 | **P44299** | 23641.07 | 5.34 | 25440 | 53.06 | unstable | 92.86 | -0.056 |
| 429 | **P44300** | 8880.67 | 9.22 | 11460 | 37.56 | stable | 140.52 | 1.100 |
