# Supplementary material for: Functional Annotation of Conserved Hypothetical Proteins from Haemophilus influenzae Rd KW20
Source: PLoS One. 2013 Dec 31;8(12):e84263. doi: 10.1371/journal.pone.0084263 (PMC3877243; doi:10.1371/journal.pone.0084263)
Supplement: Table S2 — List of predicted sub-cellular localization of 429 HPs from H. influenzae. (DOCX) [file pone.0084263.s002.docx]

| **S.No**  Table S2: List of predicted sub-cellular localization of 429 HPs from *H. influenzae* | **UNIPROT ID** | **Sub-cellular localization** | | | **Signal Peptide** | **Secretory Protein****(SecretomeP)** | **Tran membrane helices prediction** | |
| --- | --- | --- | --- | --- | --- | --- | --- | --- |
|  |  | **PSORT B** | **PSLpred** | **CELLO** |  |  | **HMMTOP** | **TMHMM** |
| 1 | **Q57048** | Cytoplasmic membrane | Inner membrane protein | Inner membrane | No | No | 13 TM helices | 13 TM helices |
| 2 | **P44465** | Unknown | Cytoplasmic protein | Cytoplasmic | No | No | Not present | Not present |
| 3 | **P44471** | Cytoplasmic | Cytoplasmic protein | Cytoplasmic | No | No | Not present | Not present |
| 4 | **P44472** | Cytoplasmic membrane | Inner membrane protein | Inner membrane | No | No | 11 TM helices | 11TM helices |
| 5 | **P43929** | Cytoplasmic | Cytoplasmic protein | Cytoplasmic | No | No | Not present | Not present |
| 6 | **P43931** | Cytoplasmic membrane | Cytoplasmic protein | Cytoplasmic | No | No | 4 TM helices | 3 TM helices |
| 7 | **P44477** | Cytoplasmic membrane | Inner membrane protein | Inner membrane | No | No | 7 TM helices | 7TM helices |
| 8 | **P44478** | Unknown | Periplasmic protein | Periplasmic protein | Yes | Yes | Not present | Not present |
| 9 | **P44484** | Cytoplasmic membrane | Inner membrane protein | Inner membrane | No | No | 4 TM helices | 4 TM helices |
| 10 | **P71336** | Periplasmic | Outer membrane protein | Cytoplasmic | Yes | No | Not present | Not present |
| 11 | **P43932** | Cytoplasmic membrane | Inner membrane protein | Inner membrane | No | No | 7 TM helices | 7 TM helices |
| 12 | **P44492** | Cytoplasmic | Cytoplasmic protein | Cytoplasmic | No | No | Not present | Not present |
| 13 | **P43935** | Cytoplasmic | Cytoplasmic protein | Cytoplasmic | No | No | Not present | Not present |
| 14 | **P43936** | Unknown | Cytoplasmic protein | Cytoplasmic | No | No | Not present | Not present |
| 15 | **P44500** | Cytoplasmic | Cytoplasmic protein | Cytoplasmic | No | No | Not present | Not present |
| 16 | **P43937** | Unknown | Inner membrane protein | Extracellular | No | No | Not present | Not present |
| 17 | **P43938** | Unknown | Periplasmic protein | Cytoplasmic | No | Yes | Not present | Not present |
| 18 | **P44506** | Cytoplasmic | Inner membrane protein | Cytoplasmic | No | No | Not present | Not present |
| 19 | **P44507** | Cytoplasmic | Cytoplasmic protein | Cytoplasmic | No | No | Not present | Not present |
| 20 | **Q57493** | Cytoplasmic membrane | Inner membrane protein | Inner membrane | No | No | 11 TM helices | 11 TM helices |
| 21 | **P44509** | Cytoplasmic | Cytoplasmic protein | Cytoplasmic | No | No | Not present | Not present |
| 22 | **P43939** | Unknown | Cytoplasmic protein | Cytoplasmic | No | No | 1 TMhelices | 1 TM helices |
| 23 | **Q57060** | cytoplasmic | Cytoplasmic protein | Cytoplasmic | No | No | Not present | Not present |
| 24 | **P43940** | Unknown | Extracellular protein | Extracellular protein | No | No | Not present | Not present |
| 25 | **P44515** | Unknown | Cytoplasmic protein | Cytoplasmic | No | No | Not present | Not present |
| 26 | **Q57354** | Cytoplasmic | Cytoplasmic protein | Cytoplasmic | No | No | Not present | Not present |
| 27 | **P44520** | Cytoplasmic membrane | Inner-membrane protein | Inner membrane | No | No | 5 TM helices | 5 TM helices |
| 28 | **P43943** | Unknown | Periplasmic protein | Cytoplasmic | No | No | Not present | Not present |
| 29 | **P71339** | Unknown | Cytoplasmic protein | Cytoplasmic | No | No | Not present | Not present |
| 30 | **Q57097** | Cytoplasmic | Cytoplasmic protein | Cytoplasmic | No | No | 1 TM helices | 1 TM helices |
| 31 | **P43947** | Cytoplasmic membrane | Inner membrane protein | Inner membrane | No | No | 3 TM helices | 5 TM helices |
| 32 | **P44530** | Cytoplasmic membrane | Inner membrane protein | Inner membrane | No | No | 13 TM helices | 13 TM helices |
| 33 | **P43952** | Cytoplasmic | Periplasmic protein | Outer membrane | No | No | 2 TMhelices | 1 TM helices |
| 34 | **P44540** | Cytoplasmic | Cytoplasmic protein | Outer membrane | No | No | Not present | Not present |
| 35 | **P44542** | Periplasmic | Periplasmic protein | Periplasmic | Yes | Yes | Not present | 1 TM helices |
| 36 | **P44543** | Cytoplasmic membrane | Inner membrane protein | Inner membrane | No | No | 15 TMhelices | 17 TM helices |
| 37 | **O86220** | Cytoplasmic membrane | Extracellular protein | Inner membrane | No | No | 2 TMhelices | 2 TM helices |
| 38 | **P43953** | Cytoplasmic membrane | Inner membrane protein | Inner membrane | No | No | 1 TMhelices | 1 TM helices |
| 39 | **P44545** | Cytoplasmic | Cytoplasmic protein | Cytoplasmic | No | No | 1 TMhelices | 1 TM helices |
| 40 | **P43954** | Cytoplasmic | Cytoplasmic protein | Cytoplasmic | Signal peptide | No | Not present | Not present |
| 41 | **P43790** | Cytoplasmic | Cytoplasmic protein | Cytoplasmic | No | No | Not present | Not present |
| 42 | **P43960** | Cytoplasmic membrane | Inner membrane protein | Periplasmic | No | No | 1 TMhelices | 1 TM helices |
| 43 | **P44552** | Unknown | Cytoplasmic protein | Periplasmic | No | Yes | Not present | Not present |
| 44 | **P44553** | Outer membrane | Periplasmic protein | Periplasmic | Yes | Yes | No TMhelices | Not present |
| 45 | **P43961** | Unknown | Inner membrane protein | Periplasmic | Yes | Yes | Not present | Not present |
| 46 | **P46490** | Cytoplasmic membrane | Inner membrane protein | Inner membrane | No | No | 10 TM helices | 8 TM helices |
| 47 | **P43963** | Unknown | Inner membrane protein | Periplasmic | No | No | Not present | Not present |
| 48 | **P43965** | Cytoplasmic | Cytoplasmic protein | Cytoplasmic | No | No | Not present | Not present |
| 49 | **P44577** | Cytoplasmic membrane | Inner membrane protein | Inner membrane | No | No | 2 TM helices | 4 TM helices |
| 50 | **O86222** | Cytoplasmic membrane | Outer membrane protein | Inner membrane | No | No | 2 TMhelices | Not present |
| 51 | **P44579** | Cytoplasmic membrane | Inner membrane protein | Inner membrane | No | No | 10 TM helices | 8 TM helices |
| 52 | **P44583** | Cytoplasmic | Extracellular protein | Cytoplasmic | No | No | Not present | Not present |
| 53 | **P43966** | Cytoplasmic membrane | Inner membrane protein | Cytoplasmic | No | No | Not present | Not present |
| 54 | **P43968** | Unknown | Cytoplasmic protein | Cytoplasmic | No | No | Not present | Not present |
| 55 | **P44588** | Unknown | Cytoplasmic protein | Cytoplasmic | No | Yes | Not present | Not present |
| 56 | **P44593** | Unknown | Cytoplasmic protein | Cytoplasmic | No | No | Not present | Not present |
| 57 | **P43971** | Cytoplasmic | Cytoplasmic protein | Cytoplasmic | No | No | Not present | Not present |
| 58 | **P43972** | Unknown | Periplasmic protein | Periplasmic | Yes | Yes | Not present | Not present |
| 59 | **P71346** | Cytoplasmic | Periplasmic protein | Cytoplasmic | No | No | Not present | Not present |
| 60 | **P44606** | Cytoplasmic | Cytoplasmic protein | Cytoplasmic | No | No | Not present | Not present |
| 61 | **P43975** | Cytoplasmic membrane | Inner membrane protein | Inner membrane | No | No | 6 TM helices | 5 TM helices |
| 62 | **P44609** | Unknown | Cytoplasmic protein | Cytoplasmic | No | No | No TM | Not present |
| 63 | **P43980** | Cytoplasmic membrane | Cytoplasmic membrane | Cytoplasmic | No | No | Not present | Not present |
| 64 | **P43982** | Cytoplasmic | Cytoplasmic protein | Cytoplasmic | Yes | Yes | 1 TMhelices | Not present |
| 65 | **P44634** | Cytoplasmic membrane | Inner membrane protein | Inner membrane | No | No | No TM helices | Not present |
| 66 | **P43984** | Cytoplasmic membrane | Inner membrane protein | Inner membrane | No | No | 5 TM helices | 4 TM helices |
| 67 | **P44640** | Unknown | Cytoplasmic protein | Cytoplasmic | No | No | 12 TMhelices | 13 TM helices |
| 68 | **P43987** | Cytoplasmic | Cytoplasmic protein | Cytoplasmic | No | No | Not present | Not present |
| 69 | **P44641** | Cytoplasmic | Cytoplasmic protein | Cytoplasmic | No | No | Not present | Not present |
| 70 | **P44646** | Cytoplasmic membrane | Inner membrane protein | Inner membrane | No | No | 7 TM helices | 7 TM helices |
| 71 | **P44649** | Cytoplasmic | Cytoplasmic protein | Cytoplasmic | No | No | Not present | Not present |
| 72 | **P24324** | Unknown | Outer membrane protein | Cytoplasmic | No | No | No TMhelices | Not present |
| 73 | **Q57065** | Unknown | Inner membrane protein | Outer membrane | No | Yes | No TMhelices | 1 TM helices |
| 74 | **P43989** | Unknown | Inner membrane protein | Periplasmic | No | Yes | Not present | 1 TM helices |
| 75 | **P44668** | Unknown | Cytoplasmic protein | Cytoplasmic | No | No | Not present | Not present |
| 76 | **P44670** | Unknown | Outer membrane protein | Extracellular | No | No | Not present | Not present |
| 77 | **P44672** | Cytoplasmic | Cytoplasmic protein | Cytoplasmic | No | No | Not present | Not present |
| 78 | **P44675** | Cytoplasmic | Cytoplasmic protein | Cytoplasmic | No | No | Not present | Not present |
| 79 | **P44676** | Cytoplasmic | Cytoplasmic protein | Cytoplasmic | No | No | Not present | Not present |
| 80 | **P44679** | Unknown | Cytoplasmic protein | Cytoplasmic | No | No | Not present | Not present |
| 81 | **P43990** | Cytoplasmic | Extracellular protein | Cytoplasmic | No | No | Not present | Not present |
| 82 | **P43992** | Unknown | Periplasmic protein | Periplasmic | No | Yes | Not present | Not present |
| 83 | **P43994** | Unknown | Extracellular protein | Cytoplasmic | No | No | Not present | Not present |
| 84 | **P44683** | Cytoplasmic | Cytoplasmic protein | Cytoplasmic | No | No | Not present | Not present |
| 85 | **P44684** | Cytoplasmic | Cytoplasmic protein | Cytoplasmic | No | No | Not present | Not present |
| 86 | **P44686** | Unknown | Cytoplasmic protein | Cytoplasmic | No | No | Not present | Not present |
| 87 | **P44691** | Cytoplasmic membrane | Inner membrane protein | Inner membrane | No | No | 7 TMhelices | 7 TM helices |
| 88 | **P44693** | Cytoplasmic | Outer membrane protein | Outer membrane | No | Yes | 1 TMhelices | 1 TM helices |
| 89 | **Q57392** | Outer membrane | Outer membrane | Cytoplasmic | No | No | Not present | Not present |
| 90 | **P43995** | Unknown | Cytoplasmic protein | Cytoplasmic | No | Yes | Not present | Not present |
| 91 | **P44702** | Cytoplasmic | Cytoplasmic protein | Cytoplasmic | No | No | Not present | Not present |
| 92 | **P44709** | Cytoplasmic | Cytoplasmic protein | Cytoplasmic | No | No | Not present | Not present |
| 93 | **P31777** | Cytoplasmic | Cytoplasmic protein | Cytoplasmic | No | No | Not present | Not present |
| 94 | **P44711** | Unknown | Cytoplasmic | Cytoplasmic | No | No | Not present | Not present |
| 95 | **P43997** | Unknown | Inner membrane protein | Periplasmic | Yes | Yes | Not present | 1 TM helices |
| 96 | **P43998** | Unknown | Cytoplasmic protein | Cytoplasmic | No | No | Not present | Not present |
| 97 | **P44717** | Cytoplasmic membrane | Inner membrane protein | Inner membrane | Yes | No | 4 TM helices | 4 TM helices |
| 98 | **P43999** | Unknown | Outer membrane protein | Cytoplasmic | No | No | 2 TM helices | 2 TM helices |
| 99 | **P44718** | Cytoplasmic | Cytoplasmic protein | Cytoplasmic | No | No | Not present | Not present |
| 100 | **P44720** | Cytoplasmic | Cytoplasmic protein | Periplasmic | No | Yes | 1 TMhelices | 1 TM helices |
| 101 | **Q57144** | Cytoplasmic | Inner membrane protein | Cytoplasmic | No | No | Not present | Not present |
| 102 | **P44000** | Cytoplasmic | Cytoplasmic protein | Cytoplasmic | No | No | Not present | Not present |
| 103 | **P44726** | Cytoplasmic | Cytoplasmic protein | Cytoplasmic | No | No | Not present | Not present |
| 104 | **P44003** | Unknown | Inner membrane protein | Cytoplasmic | No | No | Not present | Not present |
| 105 | **P44005** | Cytoplasmic membrane | Inner membrane protein | Inner membrane | No | No | 4 TMhelices | 4 TM helices |
| 106 | **O05023** | Unknown | Periplasmic protein | Cytoplasmic | No | Yes | Not present | Not present |
| 107 | **P44733** | Cytoplasmic | Cytoplasmic protein | Cytoplasmic | No | No | Not present | Not present |
| 108 | **P44010** | Cytoplasmic membrane | Inner membrane protein | Inner membrane | No | No | 4 TMhelices | 4 TM helices |
| 109 | **P44740** | Cytoplasmic | Periplasmic protein | Cytoplasmic | No | No | Not present | Not present |
| 110 | **P44743** | Cytoplasmic | Cytoplasmic protein | Cytoplasmic | No | No | Not present | Not present |
| 111 | **P44744** | Cytoplasmic | Cytoplasmic protein | Cytoplasmic | No | No | Not present | Not present |
| 112 | **Q57256** | Cytoplasmic membrane | Inner membrane protein | Inner membrane | No | No | 5 TMhelices | 5 TM helices |
| 113 | **P44012** | Periplasmic | Periplasmic protein | Periplasmic | No | Yes | 1 TMhelices | 1 TM helices |
| 114 | **P44013** | Unknown | Cytoplasmic protein | Cytoplasmic | No | No | Not present | Not present |
| 115 | **P44014** | Cytoplasmic | Cytoplasmic protein | Cytoplasmic | No | No | Not present | Not present |
| 116 | **Q57409** | Unknown | Inner membrane protein | Cytoplasmic | No | No | Not present | 1 TM helices |
| 117 | **O86226** | Cytoplasmic membrane | Inner membrane protein | Inner membrane | No | No | 3 TMhelices | 3 TM helices |
| 118 | **P44016** | Cytoplasmic membrane | Inner membrane protein | Inner membrane | No | No | 15 TMhelices | 16 TM helices |
| 119 | **P44754** | Cytoplasmic | Cytoplasmic protein | Cytoplasmic | No | No | Not present | Not present |
| 120 | **P44759** | Unknown | Cytoplasmic protein | Cytoplasmic | No | No | Not present | Not present |
| 121 | **P44761** | Cytoplasmic | Cytoplasmic protein | Cytoplasmic | No | No | Not present | Not present |
| 122 | **P44017** | Unknown | Cytoplasmic protein | Cytoplasmic | No | No | Not present | Not present |
| 123 | **P44018** | Cytoplasmic membrane | Inner membrane protein | Inner membrane | No | No | 7 TMhelices | 7 TM helices |
| 124 | **P44019** | Cytoplasmic membrane | Inner membrane protein | Inner membrane | No | No | 5 TMhelices | 4 TM helices |
| 125 | **P44023** | Cytoplasmic membrane | Inner membrane protein | Inner membrane | No | No | 13TMhelices | 12 TM helices |
| 126 | **P44771** | Cytoplasmic | Cytoplasmic protein | Cytoplasmic | No | No | Not present | Not present |
| 127 | **P44782** | Cytoplasmic | Cytoplasmic protein | Cytoplasmic | No | No | Not present | Not present |
| 128 | **P44025** | Cytoplasmic | Cytoplasmic protein | Cytoplasmic | No | No | Not present | Not present |
| 129 | **P44026** | Cytoplasmic membrane | Inner membrane protein | Inner membrane | No | No | 2 TMhelices | 2 TM helices |
| 130 | **P44027** | Unknown | Cytoplasmic protein | Cytoplasmic | No | No | Not present | Not present |
| 131 | **P44796** | Unknown | Cytoplasmic protein | Inner membrane | No | No | Not present | Not present |
| 132 | **P44028** | Cytoplasmic membrane | Inner membrane protein | Cytoplasmic | No | No | 2 TMhelices | 1 TM helices |
| 133 | **P44807** | Cytoplasmic | Cytoplasmic protein | Cytoplasmic | No | No | Not present | Not present |
| 134 | **P46494** | Unknown | Cytoplasmic protein | Periplasmic | No | Yes | Not present | Not present |
| 135 | **P44031** | Unknown | Cytoplasmic protein | Cytoplasmic | No | No | Not present | Not present |
| 136 | **P44033** | Cytoplasmic | Cytoplasmic protein | Cytoplasmic | No | No | Not present | Not present |
| 137 | **P44034** | Unknown | Outer membrane protein | Cytoplasmic | No | No | Not present | Not present |
| 138 | **O86228** | Unknown | Extracellular protein | Cytoplasmic | No | No | Not present | Not present |
| 139 | **P44812** | Cytoplasmic | Cytoplasmic protein | Cytoplasmic | No | No | Not present | Not present |
| 140 | **P44036** | Cytoplasmic | Cytoplasmic protein | Cytoplasmic | No | No | Not present | Not present |
| 141 | **P71356** | Cytoplasmic membrane | Inner membrane protein | Inner membrane | No | No | 9TMhelices | 10 TM helices |
| 142 | **P44037** | Unknown | Extracellular protein | Cytoplasmic | No | No | Not present | Not present |
| 143 | **P44827** | Cytoplasmic | Periplasmic protein | Cytoplasmic | No | No | Not present | Not present |
| 144 | **Q57523** | Outer membrane | Extracellular protein | Outer membrane | No | Yes | 1TMhelices | 1 TM helices |
| 145 | **P44038** | Outer membrane | Outer membrane protein | Outer membrane | Yes | Yes | Not present | Not present |
| 146 | **P44831** | Cytoplasmic | Cytoplasmic protein | Cytoplasmic | No | No | Not present | Not present |
| 147 | **P44040** | Unknown | Periplasmic protein | Periplasmic | No | Yes | Not present | Not present |
| 148 | **P71357** | Unknown | Inner membrane protein | Cytoplasmic | No | No | Not present | Not present |
| 149 | **P44041** | Unknown | Cytoplasmic protein | Cytoplasmic | No | No | Not present | Not present |
| 150 | **P44839** | Cytoplasmic | Cytoplasmic protein | Cytoplasmic | No | No | Not present | Not present |
| 151 | **P44842** | Cytoplasmic | Cytoplasmic protein | Cytoplasmic | No | No | 1TMhelices | Not present |
| 152 | **P44844** | Cytoplasmic | Cytoplasmic protein | Cytoplasmic | No | No | Not present | Not present |
| 153 | **P44043** | Cytoplasmic | Cytoplasmic protein | Cytoplasmic | No | No | 1 TMhelices | 1 TM helices |
| 154 | **P44045** | Unknown | Inner membrane protein | Cytoplasmic | No | Yes | Not present | Not present |
| 155 | **P44047** | Unknown | Cytoplasmic protein | Cytoplasmic | No | No | Not present | Not present |
| 156 | **P44854** | Cytoplasmic membrane | Inner membrane protein | Inner membrane | No | No | 3TMhelices | 1 TM helices |
| 157 | **P44863** | Cytoplasmic membrane | Cytoplasmic protein | Inner membrane | Yes | No | 1 TMhelices | Not present |
| 158 | **P44864** | Outer membrane | Extracellular protein | Outer membrane | Yes | No | 2 TMhelices | Not present |
| 159 | **P44048** | Cytoplasmic | Cytoplasmic protein | Cytoplasmic | No | No | Not present | Not present |
| 160 | **P44050** | Cytoplasmic | Cytoplasmic protein | Cytoplasmic | No | No | Not present | Not present |
| 161 | **P44869** | Cytoplasmic | Cytoplasmic protein | Cytoplasmic | No | Yes | Not present | Not present |
| 162 | **P44052** | Cytoplasmic | Cytoplasmic protein | Cytoplasmic | No | No | Not present | Not present |
| 163 | **P44053** | Unknown | Cytoplasmic protein | Cytoplasmic | No | No | Not present | Not present |
| 164 | **P44054** | Cytoplasmic membrane | Inner membrane protein | Inner membrane | No | No | 9TMhelices | 8 TM helices |
| 165 | **P44882** | Cytoplasmic | Cytoplasmic protein | Cytoplasmic | No | No | Not present | Not present |
| 166 | **P44056** | Cytoplasmic membrane | Inner membrane protein | Inner membrane | No | No | 7TMhelices | 6 TM helices |
| 167 | **P44886** | Unknown | Cytoplasmic protein | Cytoplasmic | No | No | Not present | Not present |
| 168 | **P44897** | Unknown | Periplasmic protein | Cytoplasmic | No | Yes | Not present | Not present |
| 169 | **P44898** | Cytoplasmic membrane | Inner membrane protein | Inner membrane | No | No | 5 TMhelices | 5 TM helices |
| 170 | **P44058** | Cytoplasmic | Cytoplasmic protein | Cytoplasmic | No | No | Not present | Not present |
| 171 | **P44059** | Unknown | Inner membrane protein | Cytoplasmic | No | No | Not present | Not present |
| 172 | **P44900** | Unknown | Cytoplasmic protein | Cytoplasmic | No | No | Not present | Not present |
| 173 | **P31811** | Unknown | Cytoplasmic protein | Cytoplasmic | No | Yes | Not present | Not present |
| 174 | **P44903** | Cytoplasmic | Inner membrane protein | Inner membrane | No | No | 14 TMhelices | 14 TM helices |
| 175 | **P44904** | Cytoplasmic | Cytoplasmic protein | Cytoplasmic | No | No | 1 TMhelices | Not present |
| 176 | **P44062** | Cytoplasmic | Inner membrane protein | Cytoplasmic | No | No | Not present | Not present |
| 177 | **P44905** | Cytoplasmic membrane | Inner membrane protein | Cytoplasmic | No | No | Not present | Not present |
| 178 | **P44908** | Cytoplasmic membrane | Inner membrane protein | Inner membrane | No | No | 6 TMhelices | 6 TM helices |
| 179 | **P44063** | Cytoplasmic membrane | Inner membrane protein | Outer membrane | No | No | 1 TMhelices | 1 TM helices |
| 180 | **Q57022** | Cytoplasmic membrane | Cytoplasmic protein | Cytoplasmic | No | No | 1 TMhelices | Not present |
| 181 | **P44064** | Cytoplasmic | Cytoplasmic protein | Cytoplasmic | No | No | Not present | Not present |
| 182 | **P44065** | Unknown | Periplasmic protein | Cytoplasmic | No | No | Not present | Not present |
| 183 | **P44067** | Cytoplasmic membrane | Inner membrane protein | Inner membrane | No | No | 11TMhelices | 11 TM helices |
| 184 | **P71360** | Cytoplasmic membrane | Inner membrane protein | Inner membrane | No | No | 10 TMhelices | 10 TM helices |
| 185 | **P44068** | Cytoplasmic | Cytoplasmic protein | Cytoplasmic | No | No | Not present | Not present |
| 186 | **P44069** | Cytoplasmic membrane | Inner membrane protein | Inner membrane | No | No | 4TMhelices | 4 TM helices |
| 187 | **P44070** | Cytoplasmic membrane | Inner membrane protein | Inner membrane | No | No | 8 TMhelices | 7 TM helices |
| 188 | **P44931** | Cytoplasmic | Cytoplasmic protein | Cytoplasmic | No | No | Not present | Not present |
| 189 | **P44072** | Unknown | Cytoplasmic protein | Cytoplasmic | No | No | Not present | Not present |
| 190 | **P44073** | Unknown | Inner membrane protein | Cytoplasmic | Yes | Yes | Not present | Not present |
| 191 | **P44074** | Cytoplasmic | Cytoplasmic protein | Cytoplasmic | No | No | Not present | Not present |
| 192 | **P44936** | Cytoplasmic membrane | Inner membrane protein | Inner membrane | No | No | 4 TMhelices | 4 TM helices |
| 193 | **P44938** | Cytoplasmic | Cytoplasmic protein | Cytoplasmic | No | No | Not present | Not present |
| 194 | **P44075** | Cytoplasmic | Cytoplasmic protein | Cytoplasmic | No | No | Not present | Not present |
| 195 | **P44076** | Unknown | Cytoplasmic protein | Cytoplasmic | No | No | Not present | Not present |
| 196 | **P44940** | Cytoplasmic | Cytoplasmic protein | Cytoplasmic | No | No | Not present | Not present |
| 197 | **P44077** | Extracellular | Outer membrane protein | Extracellular | No | Yes | Not present | Not present |
| 198 | **P44078** | Unknown | Cytoplasmic protein | Cytoplasmic | No | No | Not present | Not present |
| 199 | **P44941** | Unknown | Cytoplasmic protein | Outer membrane | No | No | 4TMhelices | Not present |
| 200 | **P44079** | Cytoplasmic membrane | Inner membrane protein | Cytoplasmic | No | No | Not present | 1 TM helices |
| 201 | **P44080** | Unknown | Cytoplasmic protein | Outer membrane | No | No | 1 TMhelices | 1 TM helices |
| 202 | **P44081** | Cytoplasmic membrane | Cytoplasmic protein | Outer membrane | No | No | 1 TMhelices | 1 TM helices |
| 203 | **P44082** | Cytoplasmic membrane | Inner membrane protein | Inner membrane | No | No | 1 TMhelices | 1 TM helices |
| 204 | **Q57120** | Unknown | Periplasmicprotein | Cytoplasmic | No | No | Not present | Not present |
| 205 | **P44954** | Unknown | Cytoplasmic protein | Cytoplasmic | No | No | Not present | Not present |
| 206 | **P44084** | Unknown | Periplasmic protein | Extracellular | Yes | Yes | Not present | Not present |
| 207 | **P44085** | Unknown | Periplasmic protein | Periplasmic | Yes | Yes | Not present | Not present |
| 208 | **P44086** | Unknown | Periplasmic protein | Cytoplasmic | No | Yes | Not present | Not present |
| 209 | **Q57133** | Outer membrane | Extracellular protein | Extracellular | Yes | Yes | Not present | Not present |
| 210 | **P46455** | Unknown | Inner membrane protein | Cytoplasmic | No | No | 2 TMhelices | 2 TM helices |
| 211 | **Q57147** | Cytoplasmic membrane | Inner membrane protein | Inner membrane | No | No | 3TMhelices | 4 TM helices |
| 212 | **O86230** | Cytoplasmic membrane | Inner membrane protein | Inner membrane | No | No | 5 TMhelices | 5 TM helices |
| 213 | **P44965** | Cytoplasmic | Cytoplasmic protein | Cytoplasmic | No | No | Not present | Not present |
| 214 | **P43907** | Cytoplasmic | Inner membrane protein | Periplasmic | No | No | Not present | Not present |
| 215 | **P43908** | Unknown | Cytoplasmic protein | Cytoplasmic | No | No | Not present | Not present |
| 216 | **P44972** | Unknown | Inner membrane protein | Cytoplasmic | No | No | Not present | Not present |
| 217 | **P44974** | Cytoplasmic membrane | Inner membrane protein | Inner membrane | No | No | 5TMhelices | 5 TM helices |
| 218 | **Q57134** | Cytoplasmic membrane | Inner membrane protein | Cytoplasmic | Yes | Yes | Not present | Not present |
| 219 | **P44093** | Unknown | Cytoplasmic protein | Periplasmic | No | No | Not present | Not present |
| 220 | **Q57151** | Cytoplasmic | Cytoplasmic protein | Cytoplasmic | No | No | Not present | Not present |
| 221 | **P44094** | Cytoplasmic | Cytoplasmic protein | Cytoplasmic | No | No | Not present | Not present |
| 222 | **P44095** | Unknown | Cytoplasmic protein | Cytoplasmic | No | No | Not present | Not present |
| 223 | **P44992** | Periplasmic | Periplasmic protein | Cytoplasmic | Yes | No | Not present | Not present |
| 224 | **P44993** | Cytoplasmic membrane | Inner membrane protein | Inner membrane | No | No | 11 TMhelices | 11 TM helices |
| 225 | **P44994** | Cytoplasmic membrane | Inner membrane protein | Inner membrane | No | No | 4 TMhelices | 4 TM helices |
| 226 | **P44097** | Cytoplasmic membrane | Inner membrane protein | Inner membrane | No | No | 4 TMhelices | 4 TM helices |
| 227 | **P44098** | Cytoplasmic | Cytoplasmic protein | Cytoplasmic | No | No | Not present | Not present |
| 228 | **P44099** | Cytoplasmic | Cytoplasmic protein | Cytoplasmic | No | No | Not present | Not present |
| 229 | **P44103** | Unknown | Periplasmic protein | Periplasmic | Yes | Yes | Not present | Not present |
| 230 | **Q57498** | Unknown | Periplasmic protein | Cytoplasmic | No | No | Not present | Not present |
| 231 | **P44104** | Cytoplasmic | Cytoplasmic protein | Cytoplasmic | No | No | Not present | Not present |
| 232 | **P44106** | Unknown | Periplasmic protein | Extracellular | No | Yes | Not present | Not present |
| 233 | **P44107** | Unknown | Extracellular protein | Cytoplasmic | No | No | Not present | Not present |
| 234 | **P71367** | Cytoplasmic membrane | Inner membrane protein | Outer membrane | No | No | 6TMhelices | 5 TM helices |
| 235 | **P45019** | Cytoplasmic membrane | Inner membrane protein | Inner membrane | Yes | No | 3TMhelices | 4 TM helices |
| 236 | **P44110** | Cytoplasmic membrane | Inner membrane protein | Inner membrane | No | No | 5 TMhelices | 4 TM helices |
| 237 | **P45026** | Unknown | Cytoplasmic protein | Cytoplasmic | No | No | Not present | Not present |
| 238 | **P44111** | Unknown | Extracellular protein | Cytoplasmic | No | Yes | Not present | Not present |
| 239 | **P44112** | Unknown | Cytoplasmic protein | Periplasmic | Yes | Yes | Not present | Not present |
| 240 | **P45071** | Cytoplasmic | Cytoplasmic protein | Cytoplasmic | No | No | Not present | Not present |
| 241 | **P45074** | Unknown | Periplasmic protein | Periplasmic | Yes | Yes | Not present | Not present |
| 242 | **P45075** | Unknown | Periplasmic protein | Outer membrane | No | Yes | Not present | 1 TM helices |
| 243 | **P45076** | Cytoplasmic | Cytoplasmic protein | Cytoplasmic | No | No | Not present | Not present |
| 244 | **P45077** | Cytoplasmic | Cytoplasmic protein | Outer membrane | No | No | Not present | Not present |
| 245 | **P45083** | Cytoplasmic | Cytoplasmic protein | Cytoplasmic | No | No | Not present | Not present |
| 246 | **P44116** | Cytoplasmic | Cytoplasmic protein | Cytoplasmic | No | No | Not present | Not present |
| 247 | **Q57252** | Cytoplasmic | Cytoplasmic protein | Cytoplasmic | No | No | Not present | Not present |
| 248 | **P45085** | Unknown | Cytoplasmic protein | Cytoplasmic | No | No | Not present | Not present |
| 249 | **P44117** | Cytoplasmic | Cytoplasmic protein | Cytoplasmic | No | No | Not present | Not present |
| 250 | **P44119** | Cytoplasmic | Cytoplasmic protein | Cytoplasmic | No | No | Not present | Not present |
| 251 | **P45097** | Cytoplasmic | Cytoplasmic protein | Cytoplasmic | No | No | Not present | Not present |
| 252 | **P44124** | Cytoplasmic | Cytoplasmic protein | Cytoplasmic | No | No | Not present | Not present |
| 253 | **P44125** | Unknown | Cytoplasmic protein | Cytoplasmic | Yes | Yes | Not present | Not present |
| 254 | **P45103** | Cytoplasmic | Cytoplasmic protein | Cytoplasmic | No | No | Not present | Not present |
| 255 | **P45104** | Cytoplasmic | Cytoplasmic protein | Cytoplasmic | No | Yes | Not present | Not present |
| 256 | **P44126** | Cytoplasmic | Cytoplasmic protein | Cytoplasmic | No | No | Not present | Not present |
| 257 | **P44127** | Cytoplasmic membrane | Inner membrane protein | Inner membrane | No | No | 2 TMhelices | 2 TM helices |
| 258 | **P71373** | Cytoplasmic | Cytoplasmic protein | Cytoplasmic | No | No | Not present | Not present |
| 259 | **P44129** | Cytoplasmic membrane | Extracellular protein | Inner membrane | No | No | 2 TMhelices | 2 TM helices |
| 260 | **P44131** | Cytoplasmic membrane | Periplasmic protein | Cytoplasmic | No | No | Not present | Not present |
| 261 | **P44132** | Cytoplasmic membrane | Inner membrane protein | Extracellular | Yes | Yes | Not present | 1 TM helices |
| 262 | **P45122** | Cytoplasmic membrane | Inner membrane protein | Inner membrane | No | No | 7 TMhelices | 5 TM helices |
| 263 | **P44133** | Cytoplasmic membrane | Inner membrane protein | Inner membrane | No | No | 8 TMhelices | 9 TM helices |
| 264 | **P44134** | Unknown | Periplasmic protein | Periplasmic | No | Yes | Not present | Not present |
| 265 | **P44135** | Cytoplasmic membrane | Cytoplasmic protein | Inner membrane | No | No | 5 TMhelices | 5 TM helices |
| 266 | **P44136** | Cytoplasmic membrane | Inner membrane protein | Inner membrane | No | No | 8TMhelices | 7 TM helices |
| 267 | **P44137** | Unknown | Periplasmic protein | Periplasmic | Yes | Yes | Not present | Not present |
| 268 | **P44138** | Unknown | Outer membrane protein | Cytoplasmic | No | No | Not present | Not present |
| 269 | **P44139** | Cytoplasmic membrane | Inner membrane protein | Inner membrane | No | No | 4TMhelices | 4 TM helices |
| 270 | **P44140** | Cytoplasmic | Cytoplasmic protein | Cytoplasmic | No | No | Not present | Not present |
| 273 | **P44144** | Cytoplasmic | Cytoplasmic protein | Cytoplasmic | No | No | Not present | Not present |
| 274 | **P44145** | Unknown | Inner membrane protein | Cytoplasmic | No | No | 1 TMhelices | 1 TM helices |
| 276 | **P44148** | Unknown | Cytoplasmic protein | Cytoplasmic | No | No | Not present | Not present |
| 277 | **P44150** | Cytoplasmic | Cytoplasmic protein | Cytoplasmic | No | No | Not present | Not present |
| 278 | **P45138** | Cytoplasmic | Cytoplasmic protein | Cytoplasmic | No | No | Not present | Not present |
| 279 | **P44154** | Unknown | Inner membrane protein | Cytoplasmic | No | No | Not present | Not present |
| 280 | **P44156** | Cytoplasmic | Cytoplasmic protein | Cytoplasmic | No | No | Not present | Not present |
| 281 | **P45145** | Cytoplasmic membrane | Inner membrane protein | Inner membrane | No | No | 3TMhelices | 4 TM helices |
| 282 | **P45146** | Cytoplasmic membrane | Inner membrane protein | Inner membrane | No | No | 7 TMhelices | 6 TM helices |
| 283 | **Q57320** | Cytoplasmic membrane | Inner membrane protein | Inner membrane | No | No | 6 TMhelices | 6 TM helices |
| 284 | **P45154** | Cytoplasmic | Cytoplasmic protein | Cytoplasmic | No | No | Not present | Not present |
| 285 | **P44158** | Unknown | Cytoplasmic protein | Periplasmic | No | No | Not present | Not present |
| 286 | **P71375** | Cytoplasmic membrane | Inner membrane protein | Inner membrane | No | No | 3 TMhelices | 3 TMhelices |
| 287 | **P44160** | Cytoplasmic | Cytoplasmic protein | Cytoplasmic | No | Yes | Not present | Not present |
| 288 | **P44161** | Cytoplasmic | Cytoplasmic protein | Cytoplasmic | No | No | Not present | Not present |
| 289 | **P44162** | Cytoplasmic | Cytoplasmic protein | Cytoplasmic | No | No | 1 TMhelices | Not present |
| 290 | **P44163** | Cytoplasmic membrane | Extracellular protein | Extracellular | No | No | 1 TMhelices | 1 TM helices |
| 291 | **P71376** | Cytoplasmic | Cytoplasmic protein | Cytoplasmic | No | No | Not present | Not present |
| 292 | **P44164** | Cytoplasmic | Cytoplasmic protein | Cytoplasmic | No | No | Not present | Not present |
| 293 | **P71378** | Unknown | Outer membrane protein | Periplasmic | Yes | Yes | 1 TMhelices | Not present |
| 294 | **P44165** | Cytoplasmic | Outer membrane protein | Outer membrane | No | Yes | Not present | Not present |
| 295 | **P71379** | Cytoplasmic | Cytoplasmic protein | Cytoplasmic | No | No | Not present | Not present |
| 296 | **P45173** | Cytoplasmic | Cytoplasmic protein | Cytoplasmic | No | No | Not present | Not present |
| 297 | **P44167** | Cytoplasmic | Cytoplasmic protein | Cytoplasmic | No | No | Not present | Not present |
| 298 | **P44168** | Cytoplasmic | Cytoplasmic protein | Cytoplasmic | No | No | Not present | Not present |
| 299 | **P45180** | Cytoplasmic | Cytoplasmic protein | Cytoplasmic | No | No | Not present | Not present |
| 300 | **P45182** | Outer membrane | Extracellular protein | Outer membrane | No | Yes | 2TMhelices | 1 TM helices |
| 301 | **P44169** | Cytoplasmic | Extracellular protein | Cytoplasmic | No | No | Not present | Not present |
| 302 | **P44170** | Cytoplasmic membrane | Inner membrane protein | Inner membrane | No | No | 10TMhelices | 10 TM helices |
| 303 | **P44171** | Unknown | Inner membrane protein | Outer membrane | No | No | Not present | Not present |
| 304 | **O86237** | Cytoplasmic | Cytoplasmic protein | Cytoplasmic | No | No | Not present | Not present |
| 305 | **P44172** | Cytoplasmic | Cytoplasmic protein | Cytoplasmic | No | No | Not present | Not present |
| 306 | **P44173** | Unknown | Cytoplasmic protein | Cytoplasmic | No | No | Not present | Not present |
| 307 | **P44175** | Unknown | Cytoplasmic protein | Periplasmic | Yes | No | Not present | Not present |
| 308 | **P44176** | Cytoplasmic | Cytoplasmic protein | Cytoplasmic | No | No | Not present | Not present |
| 309 | **P44177** | Unknown | Cytoplasmic protein | Cytoplasmic | No | No | Not present | Not present |
| 310 | **P44180** | Cytoplasmic | Periplasmic protein | Periplasmic | No | Yes | 1 TMhelices | Not present |
| 311 | **P44181** | Unknown | Cytoplasmic protein | Cytoplasmic | No | No | Not present | Not present |
| 312 | **P44183** | Unknown | Cytoplasmic protein | Cytoplasmic | No | No | Not present | Not present |
| 313 | **P45197** | Cytoplasmic | Cytoplasmic protein | Cytoplasmic | No | No | Not present | Not present |
| 314 | **P44185** | Unknown | Periplasmic protein | Periplasmic | Yes | Yes | 1 TMhelices | Not present |
| 315 | **P44186** | Unknown | Cytoplasmic protein | Cytoplasmic | No | No | 1 TMhelices | 1 TM helices |
| 316 | **P44187** | Cytoplasmic | Cytoplasmic protein | Cytoplasmic | No | No | Not present | Not present |
| 317 | **P44188** | Cytoplasmic membrane | Inner membrane protein | Inner membrane | No | No | 3TMhelices | 2 TM helices |
| 318 | **P44189** | Unknown | Cytoplasmic protein | Cytoplasmic | No | No | Not present | Not present |
| 319 | **P44190** | Unknown | Cytoplasmic protein | Cytoplasmic | No | No | Not present | Not present |
| 320 | **P44191** | Unknown | Cytoplasmic protein | Cytoplasmic | No | No | Not present | Not present |
| 321 | **P44193** | Cytoplasmic | Cytoplasmic protein | Cytoplasmic | No | No | Not present | Not present |
| 322 | **P44194** | Unknown | Cytoplasmic protein | Cytoplasmic | No | Yes | Not present | Not present |
| 323 | **P44196** | Unknown | Periplasmic protein | Periplasmic | Yes | Yes | Not present | Not present |
| 324 | **P45202** | Cytoplasmic | Periplasmic protein | Periplasmic | No | Yes | Not present | Not present |
| 325 | **P56507** | Unknown | Extracellular protein | Cytoplasmic | No | No | Not present | Not present |
| 326 | **P44197** | Cytoplasmic | Cytoplasmic protein | Cytoplasmic | No | No | Not present | Not present |
| 327 | **Q57152** | Unknown | Periplasmic protein | Cytoplasmic | No | No | Not present | Not present |
| 328 | **P44198** | Cytoplasmic | Cytoplasmic protein | Cytoplasmic | No | No | Not present | Not present |
| 329 | **P44201** | Cytoplasmic membrane | Inner membrane protein | Inner membrane | No | No | 5 TMhelices | 5 TM helices |
| 330 | **P44202** | Cytoplasmic membrane | Inner membrane protein | Inner membrane | No | No | 6 TMhelices | 6 TM helices |
| 331 | **P44203** | Unknown | Inner membrane protein | Outer membrane | No | No | 2 TMhelices | Not present |
| 332 | **P45217** | Outer membrane | Outer membrane protein | Outer membrane | Yes | Yes | Not present | Not present |
| 335 | **P44205** | Unknown | Periplasmic protein | Cytoplasmic | No | No | Not present | Not present |
| 336 | **Q57380** | Cytoplasmic membrane | Inner membrane protein | Inner membrane | No | No | 1TMhelices | 2 TM helices |
| 337 | **P44208** | Unknown | Periplasmic protein | Periplasmic | No | Yes | Not present | Not present |
| 338 | **P44209** | Unknown | Cytoplasmic protein | Cytoplasmic | No | No | Not present | Not present |
| 339 | **P44210** | Unknown | Cytoplasmic protein | Cytoplasmic | No | No | Not present | Not present |
| 340 | **P44212** | Unknown | Cytoplasmic protein | Cytoplasmic | No | No | Not present | Not present |
| 341 | **P44213** | Unknown | Periplasmic protein | Cytoplasmic | No | No | Not present | Not present |
| 342 | **P44214** | Cytoplasmic | Cytoplasmic protein | Cytoplasmic | No | No | Not present | Not present |
| 343 | **P44215** | Cytoplasmic | Cytoplasmic protein | Cytoplasmic | No | No | Not present | Not present |
| 344 | **P44217** | Unknown | Periplasmic protein | Periplasmic | Yes | Yes | Not present | Not present |
| 345 | **P44218** | Unknown | Periplasmicprotein | Periplasmic | No | Yes | Not present | Not present |
| 346 | **P44219** | Cytoplasmic membrane | Inner membrane protein | Cytoplasmic | No | Yes | 1 TMhelices | 1 TM helices |
| 347 | **P44220** | Unknown | Inner membrane protein | Periplasmic | No | No | 1 TMhelices | 1 TM helices |
| 348 | **P44221** | Unknown | Cytoplasmic protein | Cytoplasmic | No | No | Not present | Not present |
| 349 | **P44222** | Unknown | Cytoplasmic protein | Cytoplasmic | No | No | 1 TMhelices | 1 TM helices |
| 350 | **O86242** | Unknown | Cytoplasmic protein | Cytoplasmic | No | No | Not present | Not present |
| 351 | **P44223** | Unknown | Cytoplasmic protein | Cytoplasmic | No | No | Not present | Not present |
| 352 | **P44224** | Cytoplasmic | Cytoplasmic protein | Cytoplasmic | No | No | Not present | Not present |
| 353 | **P44225** | Periplasmic | Cytoplasmic protein | Cytoplasmic | No | No | Not present | Not present |
| 354 | **P44226** | Cytoplasmic | Outer membrane protein | Cytoplasmic | No | No | Not present | Not present |
| 355 | **P44227** | Cytoplasmic | Periplasmic protein | Periplasmic | No | Yes | Not present | Not present |
| 356 | **P44228** | Unknown | Extracellular protein | Extracellular | No | Yes | Not present | Not present |
| 357 | **Same as 356** | - | - | - | - | - | - | - |
| 358 | **P44230** | Unknown | Cytoplasmic protein | Cytoplasmic | No | No | Not present | Not present |
| 359 | **P44231** | Unknown | Inner membrane protein | Periplasmic | No | Yes | Not present | Not present |
| 360 | **P44232** | Unknown | Extracellular protein | Cytoplasmic | No | Yes | Not present | Not present |
| 361 | **P44234** | Unknown | Cytoplasmic protein | Cytoplasmic | No | Yes | Not present | Not present |
| 362 | **P44235** | Unknown | Cytoplasmic protein | Cytoplasmic | No | No | Not present | Not present |
| 363 | **P44238** | Cytoplasmic | Cytoplasmic protein | Cytoplasmic | No | No | Not present | Not present |
| 364 | **P44239** | Unknown | Periplasmic protein | Cytoplasmic | No | No | Not present | Not present |
| 365 | **P44240** | Unknown | Cytoplasmic protein | Cytoplasmic | No | No | Not present | Not present |
| 366 | **P44241** | Cytoplasmic | Cytoplasmic protein | Cytoplasmic | No | No | Not present | Not present |
| 367 | **P44242** | Unknown | Cytoplasmic protein | Outer membrane | No | Yes | Not present | Not present |
| 368 | **P71390** | Unknown | Cytoplasmic protein | Cytoplasmic | No | No | Not present | Not present |
| 369 | **P44243** | Cytoplasmic | Cytoplasmic protein | Cytoplasmic | No | No | Not present | Not present |
| 370 | **P44246** | Cytoplasmic | Cytoplasmic protein | Cytoplasmic | No | No | 2TMhelices | Not present |
| 371 | **Same as 370** | - | - | - | - | - | - | - |
| 372 | **P44247** | Cytoplasmic membrane | Periplasmic protein | Inner membrane | No | No | Not present | Not present |
| 373 | **P45244** | Cytoplasmic | Cytoplasmic protein | Cytoplasmic | No | No | Not present | Not present |
| 374 | **P44251** | Cytoplasmic | Cytoplasmic protein | Cytoplasmic | No | No | Not present | Not present |
| 375 | **P44252** | Cytoplasmic membrane | Inner membrane protein | Inner membrane | No | No | 5 TMhelices | 4 TM helices |
| 376 | **P45252** | Cytoplasmic | Cytoplasmic protein | Cytoplasmic | No | No | Not present | Not present |
| 377 | **P45253** | Cytoplasmic | Cytoplasmic protein | Cytoplasmic | No | No | Not present | Not present |
| 378 | **P44253** | Cytoplasmic membrane | Inner membrane protein | Inner membrane | No | No | 2 TMhelices | 2 TM helices |
| 379 | **P44254** | Unknown | Extracellular protein | Cytoplasmic | No | Yes | Not present | Not present |
| 380 | **P44255** | Unknown | Cytoplasmic protein | Cytoplasmic | No | No | Not present | Not present |
| 381 | **P44256** | Unknown | Cytoplasmic protein | Cytoplasmic | No | No | Not present | Not present |
| 382 | **P44260** | Unknown | Inner membrane protein | Cytoplasmic | No | No | Not present | Not present |
| 383 | **Q4QKT3** | Unknown | Cytoplasmic protein | Cytoplasmic | No | No | Not present | Not present |
| 384 | **P44262** | Cytoplasmic | Cytoplasmic protein | Cytoplasmic | No | No | Not present | Not present |
| 385 | **Same as 384** |  |  |  |  |  |  |  |
| 386 | **P45267** | Cytoplasmic | Cytoplasmic protein | Cytoplasmic | No | No | Not present | Not present |
| 387 | **P44267** | Unknown | Cytoplasmic protein | Cytoplasmic | No | No | Not present | Not present |
| 388 | **P44268** | Cytoplasmic | Cytoplasmic protein | Cytoplasmic | No | No | Not present | Not present |
| 389 | **P44269** | Periplasmic | Periplasmic protein | Periplasmic | Yes | Yes | 1 TMhelices | Not present |
| 390 | **P44270** | Cytoplasmic membrane | Inner membrane protein | Inner membrane | No | No | 3 TMhelices | 2 TM helices |
| 391 | **P44272** | Cytoplasmic membrane | Extracellular protein | Outer membrane | Yes | No | 1TMhelices | 1 TM helices |
| 392 | **P44275** | Unknown | Cytoplasmic protein | Cytoplasmic | Yes | No | 2 TMhelices | 2 TMhelices |
| 393 | **P44277** | Unknown | Inner membrane protein | Periplasmic | Yes | No | Not present | Not present |
| 394 | **P44278** | Cytoplasmic membrane | Inner membrane protein | Inner membrane | No | No | 9TMhelices | 8 TM helices |
| 395 | **P71394** | Cytoplasmic | Inner membrane protein | Cytoplasmic | No | No | Not present | Not present |
| 396 | **P45279** | Cytoplasmic membrane | Inner membrane protein | Periplasmic | No | Yes | 1 TMhelices | 1 TM helices |
| 397 | **P45280** | Cytoplasmic membrane | Inner membrane protein | Inner membrane | No | No | 6TMhelices | 4 TM helices |
| 398 | **P44279** | Cytoplasmic | Cytoplasmic protein | Cytoplasmic | No | No | 1 TMhelices | 1 TMhelices |
| 399 | **Q57525** | Unknown | Cytoplasmic protein | Cytoplasmic | No | No | Not present | Not present |
| 400 | **P44280** | Cytoplasmic | Inner membrane protein | Cytoplasmic | No | No | Not present | Not present |
| 401 | **P45290** | Cytoplasmic membrane | Inner membrane protein | Inner membrane | No | No | 11TMhelices | 10 TM helices |
| 402 | **P44281** | Unknown | Extracellular protein | Cytoplasmic | No | Yes | Not present | Not present |
| 403 | **P44282** | Unknown | Cytoplasmic protein | Cytoplasmic | No | No | Not present | Not present |
| 405 | **P45298** | Cytoplasmic | Cytoplasmic protein | Cytoplasmic | No | No | Not present | Not present |
| 406 | **P45300** | Unknown | Cytoplasmic protein | Cytoplasmic | No | No | Not present | Not present |
| 407 | **P52606** | Cytoplasmic | Cytoplasmic protein | Cytoplasmic | No | No | Not present | Not present |
| 408 | **P45301** | Periplasmic | Periplasmic protein | Periplasmic | No | Yes | 1 TMhelices | 1 TM helices |
| 409 | **Q57544** | Cytoplasmic | Cytoplasmic protein | Cytoplasmic | No | No | Not present | Not present |
| 410 | **P45305** | Cytoplasmic | Cytoplasmic protein | Cytoplasmic | No | No | Not present | Not present |
| 411 | **P44283** | Unknown | Periplasmic protein | Cytoplasmic | No | Yes | Not present | Not present |
| 412 | **P44284** | Unknown | Inner membrane protein | Periplasmic | No | Yes | 1 TMhelices | 1 TM helices |
| 413 | **P44285** | Unknown | Inner membrane protein | Outer membrane | Yes | Yes | 1 TMhelices | 1 TM helices |
| 414 | **P44287** | Cytoplasmic membrane | Inner membrane protein | Inner membrane | No | No | 8 TMhelices | 8 TM helices |
| 415 | **P44288** | Unknown | Cytoplasmic protein | Outer membrane | No | No | 1TMhelices | 1 TM helices |
| 416 | **P44289** | Cytoplasmic membrane | Inner membrane protein | Inner membrane | No | No | 10 TMhelices | 9 TM helices |
| 417 | **P44290** | Unknown | Extracellular protein | Extracellular | Yes | Yes | Not present | 1 TM helices |
| 418 | **P44292** | Cytoplasmic membrane | Inner membrane protein | Inner membrane | No | No | 2 TMhelices | 2 TM helices |
| 419 | **P45332** | Cytoplasmic membrane | Inner membrane protein | Inner membrane | No | No | 6 TMhelices | 6 TM helices |
| 420 | **P45333** | Cytoplasmic membrane | Inner membrane protein | Inner membrane | No | No | 6 TMhelices | 6 TM helices |
| 421 | **P44293** | Unknown | Cytoplasmic protein | Periplasmic | Yes | Yes | Not present | Not present |
| 422 | **P44294** | Unknown | Cytoplasmic protein | Cytoplasmic | No | No | Not present | Not present |
| 423 | **P44296** | Unknown | Outer membrane protein | Extracellular | No | Yes | Not present | Not present |
| 424 | **Q57066** | Unknown | Inner membrane protein | Periplasmic | No | Yes | Not present | Not present |
| 425 | **P44297** | Unknown | Cytoplasmic protein | Cytoplasmic | No | No | Not present | Not present |
| 426 | **O05087** | Cytoplasmic membrane | Inner membrane protein | Inner membrane | No | No | 10 TMhelices | 11 TM helices |
| 427 | **P44298** | Cytoplasmic | Cytoplasmic protein | Inner membrane | No | No | Not present | Not present |
| 428 | **P44299** | Cytoplasmic | Periplasmic protein | Inner membrane | No | No | 2 TMhelices | Not present |
| 429 | **P44300** | Cytoplasmic membrane | Inner membrane protein | Inner membrane | No | No | 2 TMhelices | 2 TM helices |
