# Supplementary material for: Functional Annotation of Conserved Hypothetical Proteins from Haemophilus influenzae Rd KW20
Source: PLoS One. 2013 Dec 31;8(12):e84263. doi: 10.1371/journal.pone.0084263 (PMC3877243; doi:10.1371/journal.pone.0084263)
Supplement: Table S3 — List of annotated functions of 429 HPs from H. influenzae using BLASTp, STRING, SMART, INTERPROSCAN and MOTIF. (DOCX) [file pone.0084263.s003.docx]

| **S.NO**  Table S3: List of annotated function of 429 HPsfrom *H. influenzae*using BLASTp, STRING, SMART, INTERPROSCAN and MOTIF | **UNIPROT ID** | **Major BLAST hit** | **Predicted functional partner**  **(STRING)** | **SMART** | **INTERPROSCAN** | **MOTIF** |
| --- | --- | --- | --- | --- | --- | --- |
|  |  |  |  |  | **MOTIF FOUND** | |
|  | **Q57048** | oxoglutarate/malate translocator | CitG protein | Na_sulph_symp | Sodium/sulphate symporter | Sodium/sulfate symporter  (BLOCKS) |
|  | **P44465** | No result | lipoyltransferase | Metallo-hydrolase/oxidoreductase | YbeD-like domain | DUF493 |
|  | **P44471** | Iojap family protein | rRNA large subunit methyltransferase | Ribosomal silencing factor RsfS /  Oligomerisation domain | Protein Iojap ribosomal silencing factor RsfS | Iojap-related protein  (BLOCKS) |
|  | **P44472** | K+ uptake protein TrkA | ABC transporter ATP-binding protein | TrkA-C domain  (ligand-gated K+ channels) | Transport protein Yide | Permease YBJL predicted uptake potassium TRKA-C  (PRODOM) |
|  | **P43929** | TIGR 1619 family protein | exonuclease III | ARM repeat  (Armadillo repeats) | Domain of unknown function DUF1260 | Transglycosylase lytic cytosolic transferase (PRODOM) |
|  | **P43931** | Membrane protein | transcriptional regulatory protein | L-aspartase-like | Uncharacterised protein family UPF0283 | Fumarate lyase superfamily signature (PRINTS) |
|  | **P44477** | Bax inhibitor-1 like protein | pseudouridine synthase-like protein | Bax Inhibitor-1  (BI-1) | Bax inhibitor 1 | Bax inhibitor 1  (BLOCKS) |
|  | **P44478** | YtfJ transcriptional regulator protein | alkylphosphonate uptake protein | Ubiquitin-like | Conserved hypothetical protein CHP01626,Ytfj | YTFJ transcriptional exportedregulatorperiplasmic predicted precursorsignal Y0655 similar  (PRODOM) |
|  | **P44484** | TRAP-type transporter system, small permeas component | integral membrane protein transporter | Tripartite ATP-independent periplasmic transporters, DctQ component | Tripartite ATP-dependent periplasmic transporter, DctQ component | Tripartite ATP-independent periplasmic transporter  (BLOCKS) |
|  | **P71336** | TRAP type C4 dicarboxylate transport system, periplasmic component | integral membrane protein transporter | Bacterial extracellular solute-binding protein | TRAP-dicarboxylate transporter | TRAP dicarboxylate transporter- DctP subunit  (BLOCKS) |
|  | **P43932** | Integral membrane protein TerC | carboxy terminal protease  (protect bacteria from stress) | Integral membrane protein TerC | Integral membrane protein TerC | Integral membrane protein TerC  (BLOCKS) |
|  | **P44492** | ATP binding protein  (ATPase) | N-acetylmuramoyl-L-alanine amidase | Uncharacterised P-loop hydrolase | tRNA threonylcarbamoyl adenosine modification protein YjeE | Hydrolase kinase UPF0079  ATP binding  (PRODOM) |
|  | **P43935** | Anaerobic ribonucleoside triphosphate reductase | acyl-CoA thioesterase II | Ferritin- like | Ferritin- like | Flavin-containing monooxygenase (FMO) 4 signature (BLOCKS) |
|  | **P43936** | PemK-like family protein | peptidyl-prolyl cis-trans isomerase B | PemK-like protein | mRNA interfease  PemK-like protein | Soybean trypsin inhibitor (Kunitz-type) signature (PRINTS) |
|  | **P44500** | TatD related DNase | Sec-independent protein translocase protein TatC | TatD related DNase | TatD family deoxyribonuclease | TatD-related deoxyribonuclease  (BLOCKS) |
|  | **P43937** | isovaleryl-CoA-dehydrogenase | HP HI0083 | Acyl-CoA dehydrogenase NM domain-like | Acyl-COA hydrogenase/oxidase | Acyl-CoA dehydrogenase (BLOCKS) |
|  | **P43938** | pentatricopeptide repeat-containing protein | HP HI0082 | beta-lactamase/transpeptidase-like | No result | Group II E-class P450 signature (PRINTS) |
|  | **P44506** | putative pyridoxal phosphate-dependent enzyme, YBL036C type | Holliday junction resolvase-like protein | Alanine racemase | Alanine racemase | Proline synthetase  (PRODOM) |
|  | **P44507** | F-box protein SKP2B | glycerate dehydrgenase | Glycerate kinase | Glycerate kinase | Glycerate kinase  (PRODOM) |
|  | **Q57493** | NF-X1 type zinc finger family protein | hydroxyethylthiazole kinase | GntP family permease / citrate transporter | Gluconate transporter | Gluconate transporter  (BLOCKS) |
|  | **P44509** | CCR4-NOT transcription complex subunit 1 domain protein | L-lactate permease | Putative sugar diacid recognition | Putative sugar diacid recognition | Putative sugar diacid recognition  (BLOCKS) |
|  | **P43939** | H(+)-ATPase 7 | intracellular septation protein A | GntP family permease | Gluconate transporter | Epimerase nucleoside-diphosphate sugar  (PRODOM) |
|  | **Q57060** | Methyltransferase type 11 family protein | putative DNA binding / iron metalloprotein/AP endonuclease | Methyltransferase | Methyltransferase type II | Methyltransferase  (PRODOM) |
|  | **P43940** | Methyltransferase | lipopolysaccharide biosynthesis protein | Formate dehydrogenase/DMSO reductase, domains 1-3 | No result | Nitrate reductase, gamma subunit (BLOCKS) |
|  | **P44515** | Arsenate reductase (ArsC protein) | succinyl- diaminopimelate desuccin ylase | Arsenate reductase | Arsc_related Transcriptional regulator Spx/MgsR | Arsenate reductase and related  (BLOCKS) |
|  | **Q57354** | NIF3-like protein (metal-binding protein) | lamB/YcsF family protein | NGG1 interacting factor 3 | Ngg1p interact ting factor 3 , NIF 3 | Ngg1- interacting factor  (PRODOM) |
|  | **P44520** | Inner membrane protein | Dihydrofolate reductase | Protein of unknown function (DUF1212) | Domain of unknown function DUF1212 | Phosphopantetheine attachment site  (PROSITE PATTERN) |
|  | **P43943** | heat-shock protein | seryl-tRNA synthetase | Urease beta subunit | Unintegrated | Mitochondrial ATP synthase B chain  (BLOCKS) |
|  | **P71339** | Transposase | hemin receptor | Transposase protein | Homeodomain like | Putative transposase, YhgA-like  (BLOCKS) |
|  | **Q57097** | sulfur acceptor protein CsdL  (high-affinity zinc transporter periplasmic component) | murein transgycosylase A | Ubiquitin activating enzyme | UBA/THIF-type NAD/FAD binding protein | U UBA/THIF-type NAD/FAD binding fold  (BLOCKS) |
|  | **P43947** | Arabinose efflux permease | UDP-2,3-diacylglucosamine hydrolase | Acyl-CoA N-acyltransferases (Nat) | Protein of unknown function DUF2301 | Bacterial lipid A biosynthesis acyltransferase  (BLOCKS) |
|  | **P44530** | xanthine/uracil/vitamin C permease | GMP synthtase | Xanthin/uracil/vit C permease | Xanthin/uracil/vit C permease | Xanthin/uracilfamily permease  (PRODOM) |
|  | **P43952** | sugar transporter (AsmA-like C-terminal domain protein) | sugar efflux transporter | Concanavalin A-like lectins/glucanases | AsmA-like C-terminal region | Glycoside hydrolase, family 34 (BLOCKS) |
|  | **P44540** | N-acetylmannosamine kinase  (HTH-type transcriptional regulator) | N-acetylmannoseamine-6-phosphate 2 epimerase | Helix turn helix | Helix turn helix RpiR | Helix turn helix motif protein RpiR  (BLOCKS) |
|  | **P44542** | sialic acid transporter, TRAP-type C4-dicarboxylate transport system, periplasmic component | part of TRAP transport system | Bacterial extracellular solute binding protein | TRAP dicarboxylate transporter | TRAP dicarboxylate transporter ,DctP subunit  (BLOCKS) |
|  | **P44543** | C4-dicarboxylate ABC transporter permease | part of TRAP transport sysytem | Tripartite ATP-independent priplasmic transporter | TRAP trnspoter permease protein | TRAP C4-dicarboxylate transport system permease D  (BLOCKS) |
|  | **O86220** | protein-S-isoprenylcysteine methyltransferase | N-acetylmannoseamine -6-phosphate 2-epimerase | N-terminal domain of alpha and beta subunits of F1 ATP synthase | No result | Fungal ATP synthase 8 like (BLOCKS) |
|  | **P43953** | protein-S-isoprenylcysteine methyltransferase | N-acetylmannoseamine -6-phosphate 2-epimerase | Elongation factor TFIIS domain 2 | Phospholipid methytransferase | Isoprenylcysteine carboxy methyltransferase  (BLOCKS) |
|  | **P44545** | protease modulator complex HflKC subunit HflC | HflK HI0151 , could encode or regulate protease | Prohibitin homolog  (Band 7 protein) | Band 7 protein/  HflC protease like | HFLC protease  (PRODOM) |
|  | **P43954** | 4'-phosphopantetheinyl transferase | dephospho-CoA kinase | 4’’-phosphopantetheinyl transferase | 4’’-phosphopantetheinyl transferase | 4’’-phosphopantetheinyl transferase  (PRODOM) |
|  | **P43790** | putative metal/nucleic acid-binding protein | 50S ribosomal protein L32 | Second domain of Mu2 adaptin subunit (ap50) of ap2 adaptor | DUF 177 protein of unknown function | Protein of unknown function DUF177 (BLOCKS) |
|  | **P43960** | Na(+)-translocating NADH-quinone reductase subunit E | tRNA-specific 2-thiouridylase MnmA | Protein of unknown function (DUF539) | DUF539 protein of unknown function | Transmembrane four family signature (BLOCKS) |
|  | **P44552** | polyphenol oxidase | 23S rRNA pseudouridine synthase D | multi-copper polyphenol oxidoreductase laccase | multi-copper polyphenol oxidoreductase laccase | Copper containing nitrite reductase signature (BLOCKS) |
|  | **P44553** | Lipoprotein | 23S rRNA pseudouridine synthase D | Tetratricopeptide repeat like | Tetratricopeptide repeat like helical | TPR repeat  (PRODOM) |
|  | **P43961** | Adhesin protein E (PE) | pyruvate formate lyase activating enzyme 1 | RNA methyl transferase | Periplasmic protein , HI0178 | Prokaryotic membrane protein lipid attachment side profile  (PROSITE PROFILE) |
|  | **P46490** | inner membrane protein YfcA | penicillin – insensitive murein endopeptidase | Sulfite exporter TauE/SafE | Transmembrane protein Tau like | YFCA similar protein permease (PRODOM) |
|  | **P43963** | Shikimate kinase | NAD nucleotidase | Aldolase | No result | Pneumovirus nucleocapsid protein (BLOCKS) |
|  | **P43965** | transposase IS200-family protein | type 1 restriction modification specificity protein | Transposases | Transposase IS200 like | YAFM protein like  transposase activity  (PRODOM) |
|  | **P44577** | Vascular endothelial growth factor receptor 3 like  (Uniprot) | cytochrome c type biogenesis protein CcmE | NAD(P)-binding Rossmann-fold domains | Protein of unknown function DUF417 | 6-phosphogluconate dehydrogenase, NAD binding domain (BLOCKS) |
|  | **O86222** | uracil-DNA glycosylase | putative RNA methyltransferase | Uracil DNA glycosylase | Uracil DNA glycosylase like | Uracil DNA glycosylase  (PRODOM) |
|  | **P44579** | DMT superfamily drug/metabolite transporter RarD | lipopolysaccharide biosynthesis protein | EamA like transporter | RarD protein | Neutral zinc metallopeptidases, zinc-binding region signature  (PROSITE) |
|  | **P44583** | Putative sugar isomerase | putative HTH-type transcriptional regulator | bacterial YhcH protein like  (indicates a role in sialic acid catabolism) | Clavaminate synthase-like | bacterial YhcH protein like (PRODOM) |
|  | **P43966** | glycosyltransferase family 8 | HP HI0227 | Nucleotide-diphospho-sugar transferases | Unintegrated | Glycosyl transferase, family 8 (BLOCKS) |
|  | **P43968** | dithiobiotin synthetase | 1-acyl-sn-glycerol-3-phosphate acyltransferase | No result | Unintegrated | Core binding factor, beta subunit  (BLOCKS) |
|  | **P44588** | Alternative ribosome-rescue factor A | arsenate reductase | FAD-linked reductases, C-terminal domain | Protein of unknown function , DUF331 | Ribonucleotide reductase (BLOCKS) |
|  | **P44593** | SirA family protein | queuine tRNA-ribosyltransferase | SirA-like (TusA)protein | Tus- A like domain | Protein of unknown function UPF0033  (BLOCKS) |
|  | **P43971** | Hemerythrin HHE cation binding domain protein | queuine tRNA-ribosyltransferase | Hemerythrin HHE cation binding domain | Hemerythrin /HHE cation binding motif | Hemerythrin (BLOCKS) |
|  | **P43972** | excinuclease ABC subunit A | monofunctional biosynthetic peptidoglycan transglycosylase | WD40 repeat-like | Uncharacterized conserved protein UCP020772/  Prokar_lipoprotein | Prokaryotic membrane lipoprotein lipid attachment site profile  (PROSITE) |
|  | **P71346** | ribosome-associated inhibitor A | lic-1 operon protein | Sigma 54 modulation protein / S30EA ribosomal protein | S30EA ribosomal protein/ Sigma 54 modulation protein | Sigma 54 modulation protein/ribosomal protein S30E  (BLOCKS) |
|  | **P44606** | tRNA-dihydrouridine synthase C | Dna-J like membrane chaperone protein | Dihydrouridine synthase (Dus) | tRNA-Dihydrouridine synthase | tRNA-Dihydrouridine synthase  (PRODOM) |
|  | **P43975** | pe-tn-6--lipooligosaccharide phosphorylethanolamine transferase | D,D-heptose 1,7-bisphosphate phosphatase | Sulphatases EC 3.1.6. | Sulphatase | Integral transmembrane  (PRODOM) |
|  | **P44609** | SEC-C motif domain-containing protein | ribonuclease BN | SEC-C motif | SEC-C motif | SEC-C motif  (BLOCKS) |
|  | **P43980** | transcriptional regulator | Holliday junction resolvase-like protein | Cysteine proteinases | Protein of unknown function DUF179 | YQGE transcriptional DUF179 F14M2.10 regulator  (PRODOM) |
|  | **P43982** | Holliday junction DNA helicase RuvB | site-specific tyrosine recombinase XerD | Cyclin-like | Uncharacterizedprotein family HI0310 | HI0310 PRECURSOR SIGNAL  (PRODOM) |
|  | **P44634** | DNA-binding regulatory protein, YebC/PmpR family/  dATP pyrophosphohydrolase | Holliday junction resolvase | transcriptional regulatory protein YebC | Transcriptional regulator TACO1 like | Jun-like transcription factor  (BLOCKS) |
|  | **P43984** | isoprenylcysteine carboxyl methyltransferase family protein | high-affinity zinc transporter periplasmic component | Isoprenylcysteine carboxyl methyltransferase (ICMT) | Isoprenylcysteine carboxyl methyltransferase | Isoprenylcysteine carboxyl methyltransferase  (BLOCKS) |
|  | **P44640** | sodium:proton antiporter | primosomal replication protein N | Na+/H+ antiporter | Na+/H+ antiporter NhaC like | Na+/H+ antiporter NhaC  (BLOCKS) |
|  | **P43987** | primosomal replication protein N | HP HI0325 | Primosomal replication protein priB and priC | Primosomal replication protein priB / priC | Mitochondrial protein of unknown function DUF1082 (BLOCKS) |
|  | **P44641** | lysine 2,3-aminomutase | elongation factor P | Radical SAM , L-lysine 2,3-aminomutase (SCOP) | Lysine 2,3-aminomutase like | L-lysine 2,3-aminomutase  (PRODOM) |
|  | **P44646** | PerM family permease | nitrogen regulatory protein P-II | Domain of unknown function DUF20 | Protein of unknown function UPF0118 | PERMEASE SO2920  (PRODOM) |
|  | **P44649** | YggL | tRNA (guanine-N(7)-)-methyltransferase | Acyl-CoA dehydrogenase NM domain-like | Protein with unknown function (DUF469) | Haemagglutinin HA1/HA2 chain signature  (BLOCKS) |
|  | **P24324** | CMP-neu5Ac-lipooligosaccharide alpha 2-3 sialyltransferase | UDP-glucose 4-epimerase | Alpha-2,3-sialyltransferase (CST-I) | Alpha-2,3-sialyltransferase (CST-I) | Alpha-2,3-sialyltransferase (PRODOM) |
|  | **Q57065** | transcriptional regulator with an N-terminal xre-type HTH domain | 4-hydroxy-3-methylbut-2-en-1-yl diphosphate synthase | Helix-turn-helix XRE-family like proteins | Domain of unknown function (DUF4115) | Helix-turn-helix (PRODOM) |
|  | **P43989** | membrane protein | histidyl-tRNA synthetase | TPR-like (Tetratricopeptide repeat) | Tetratricopeptide-like helical | Paired-like homeodomain protein  (BLOCKS) |
|  | **P44668** | Fe-S cluster related protein IscX | Ferredoxin | Protein IscX (Iron binding protein) | ISC system FeS cluster assembly , IscX | Cytochrome b559, alpha subunit  (BLOCKS) |
|  | **P44670** | histidyl-tRNA synthetase | chaperone protein HscA | beta-lactamase/transpeptidase-like | Protein of unknown function DUF2625 | Propetide, peptidase C25 (BLOCKS) |
|  | **P44672** | iron-binding protein IscA | scaffold protein | Iron-binding protein IscA | FeS cluster biogenesis | Hypothetical hesB/yadR/yfhF family signature  (PROSITE) |
|  | **P44675** | Rrf2 family transcriptional regulator | cysteine desulfurase | Transcriptional regulator | Rrf2-type HTH domain like | Rrf2-type HTH domain signature  (PROSITE) |
|  | **P44676** | tRNA/rRNA methyltransferase | cysteinyl-tRNA synthetase | SpoU rRNA Methylase | tRNA/rRNA methyltransferase (SpoU) | tRNA/rRNA methyltransferase (SpoU)  (BLOCKS) |
|  | **P44679** | acyl-CoA thioesterase | colicin transport protein | Thioesterase | Thioesterase | 4-hydroxybenzoyl-CoA thioesterase  (BLOCKS) |
|  | **P43990** | putative M22 peptidase-like protein YeaZ | putative DNA-binding/iron metalloprotein/AP endonuclease | Glycoprotease | O-Sialoglycoprotein endopeptidase | O-Sialoglycoprotein endopeptidase  (PRODOM) |
|  | **P43992** | Acyltransferase | O-antigen acetylase | SGNH hydrolase | SGNH hydrolase-type esterase domain | lipopolysaccharide acyltransferase  (PRODOM) |
|  | **P43994** | protein YfjF | electron transport complex protein RnfB | FMN-linked oxidoreductases | RnfH protein | NADH:flavin oxidoreductase/NADH oxidase (BLOCKS) |
|  | **P44683** | cupin family protein | exodeoxyribonuclease VII large subunit | RmlC-like cupins | JmjC domain | Cupin 4  (BLOCKS) |
|  | **P44684** | ADP-ribose pyrophosphatase | cyclic 3',5'-adenosine monophosphate phosphodiesterase | NUDIX hydrolases | Nudix hydrolase domain profile | Nudix hydrolase domain profile  (PROSITE PROFILE) |
|  | **P44686** | DNA mismatch repair protein | hemoglobin-binding protein | Head and neck region of the ectodomain of NDV fusion glycoprotein | Conserved hypothetical protein CHP00743 | T-cell surface glycoprotein CD4 signature (BLOCKS) |
|  | **P44691** | high-affinity zinc uptake system membrane protein ZnuB | ABC transporter ATP-binding protein | ABC 3 transporter family | ABC 3 transporter | ABC transporter, family 3  (BLOCKS) |
|  | **P44693** | Peptidase M23 | high-affinity zinc transporter periplasmic component | Opacity-associated protein A LysM-like domain / endopeptidases | Opacity-associated protein A | Opacity-associated protein A, N-terminal  (BLOCKS) |
|  | **Q57392** | opacity outermembrane protein | ribonuclease E | Opacity family porin protein | Porin, opacity type | Porin, opacity type  (BLOCKS) |
|  | **P43995** | No result | DNA topoisomerase IV subunit A | Protein of unknown function (DUF1778) | Ribbon-helix- helix | ATP-dependent helicase, DEAH-box  (BLOCKS) |
|  | **P44702** | tRNA (adenine-N6)-methyltransferase | thymidylate kinase | O-methyltransferase | N-6 Adenine-specific DNA methylases | N-6 Adenine-specific DNA methylases signature  (PROSITE PATTERN) |
|  | **P44709** | D-fructose-6-phosphate amidotransferase | NADH pyrophosphatase | NAD(P)-binding Rossmann-fold domains | Protein of unknown function (DUF416) | Malate dehydrogenase (PRODOM) |
|  | **P31777** | DNA (exogenous) processing protein | competence protein F | S-adenosyl-L-methionine-dependent methyltransferases | S-adenosyl-L-methionine-dependent methyltransferases superfamily | N-6 Adenine-specific DNA methylases signature  (PROSITE PATTERN) |
|  | **P44711** | DNA-binding protein | recombination protein RecR | Ribosome recycling factor, RRF | Nucleoid-associated protein YbaB | Nucleoid-associated protein YbaB  (PRODOM) |
|  | **P43997** | DNA polymerase III subunit delta' | antigen | Galactose-binding domain-like | Prokar_lipoprotein | Prokaryotic membrane lipoprotein lipid attachment site profile  (PROSITE PROFILE) |
|  | **P43998** | RNA polymerase sigma factor | virulence-associated protein D | Soluble quinoprotein glucose dehydrogenase | Unintegrated | Cytochrome bd ubiquinol oxidase, subunit I  (BLOCKS) |
|  | **P44717** | cystathionine-beta-synthase CBS domain protein | thymidylate kinase | cystathionine-beta-synthase | Cystathionine beta-synthase, core | CBS domain profile  (PROSITE PROFILE) |
|  | **P43999** | twin-arginine translocation protein TatA/E | hemoglobin-binding protein | Concanavalin A-like lectins/glucanases superfamily | Bacteriophage phiNM3, A0EWY4 | Glycosyl hydrolase family 30 signature (BLOCKS) |
|  | **P44718** | DNAse | thymidylate kinase | TatD related DNase | TatD type deoxyribonuclease | TatD deoxyribonuclease family signature 1  (PROSITE PATTERN) |
|  | **P44720** | aminodeoxychorismate lyase | DNA polymerase III subunit delta | ARM repeat | Zinc finger C2H2-type/integrase DNA-binding domain | Aminodeoxychorismate lyase  (PRODOM) |
|  | **Q57144** | coproporphyrinogen III oxidase | colicin V production protein | Aldolase | Protein of unknown function DUF535 | VIRK virulence protein  (PRODOM) |
|  | **P44000** | D-3-phosphoglycerate dehydrogenase | aminopeptidase P | Aminomethyltransferase folate-binding domain | Glycine cleavage T-protein, N-terminal | Glycine cleavage T protein (aminomethyl transferase)  (BLOCKS) |
|  | **P44726** | K+ -transporting ATPase | DNA-directed RNA polymerase subunit omega | YicC-like , N-terminal region | YicC-like , N-terminal region | YICC Helix alpha stress-induced uncharacterized protein  (PRODOM) |
|  | **P44003** | S-ribosylhomocysteinase | putative phosphatase/phosphohexomutase | PTS-regulatory domain, PRD | PTS-regulatory domain, PRD | PRD domain profile  (PROSITE PROFILE) |
|  | **P44005** | glucose-inhibited division protein B | putative phosphatase/phosphohexomutase | SNARE associated Golgi protein | SNARE associated Golgi protein | Type III secretion system inner membrane P protein (BLOCKS) |
|  | **O05023** | Transposase/integrase | type III restriction-modification system methyltransferase-like protein | Integrase core domain | Ribonuclease H-like domain | Hox9 activation region  (BLOCKS) |
|  | **P44733** | DNA recombination protein RmuC | excinuclease ABC subunit B | RmuC DNA recombination protein | RmuC DNA recombination proteins | RmuC DNA recombination proteins  (PRODOM) |
|  | **P44010** | Membrane protein | transport protein | Uncharacterized protein family, UPF0114 | Uncharacterized protein family, UPF0114 | Uncharacterized protein family, UPF0114 |
|  | **P44740** | 1,4-dihydroxy-2-naphthoate octaprenyltransferase | prolyl-tRNA synthetase | tRNA (adenine(37)-N6)-methyltransferase | S-adenosylmethionine-dependent methyltransferase | TsaA-like domain signature  (PROSITE PATTERN) |
|  | **P44743** | pyruvate-formate lyase-activating enzyme | anaerobic ribonucleoside triphosphate reductase | Radical SAM protein | Radical SAM protein | Radical activating enzymes signature  (PROSITE PATTERN) |
|  | **P44744** | glycine radical enzyme, YjjI family | pyruvate formate lyase-activating enzyme 1 | PFL-like glycyl radical enzyme | PFL-like glycyl radical enzyme | YJJI glycine radical enzyme  (PRODOM) |
|  | **Q57256** | Membrane protein | carbamate kinase | Uncharacterized BCR, YitT  DUF161 | Protein of unknown function DUF161 | GGY family of carbohydrate kinases signature 2  (PROSITE PATTERN) |
|  | **P44012** | ribonuclease HI | ferredoxin | Ribonuclease T2 | Ribonuclease T2 like | Ribonuclease T2 family histidine active site 1  (PROSITE PATTERN) |
|  | **P44013** | 6-phosphogluconate dehydrogenase | diadenosine tetraphosphatase | Glucose-6-phosphate 1-dehydrogenase | Glucose-6-phosphate 1-dehydrogenase like | Glycoside hydrolase, family 5 (BLOCKS) |
|  | **P44014** | glucose-6-phosphate 1-dehydrogenase | type I restriction enzyme | FAD/NAD(P)-binding domain | Transposase IS200-like | Transposase IS30  (BLOCKS) |
|  | **Q57409** | integral membrane protein/LysR family transcriptional regulator | 6-phosphogluconolactonase | Phosphoenolpyruvate/pyruvate domain | No result | Phosphoinositide 3-kinase, ras-binding domain (BLOCKS) |
|  | **O86226** | Membrane protein | sulfite synthesis pathway protein | Clathrin heavy-chain terminal domain | No result | Porin, opacity type  (BLOCKS) |
|  | **P44016** | oligopeptide transporter, OPT family | extragenic suppressor | OPT oligopeptide transporter protein | OPT oligopeptide transporter protein | OPT oligopeptide transporter protein  (BLOCKS) |
|  | **P44754** | ribosome-associated heat shock protein Hsp15 | HSP33-like chaperonin | Heat shock protein 15 | RNA-binding S4 domain | S4 RNA-binding domain profile  (PROSITE PROFILE) |
|  | **P44759** | slyX like protein (Lysin protein) | FKBP-type peptidyl-prolyl cis-trans isomerase | Putative DNA-binding domain/ SlyX like | SlyX | DM DNA-binding (BLOCKS) |
|  | **P44761** | DNA-binding transcriptional regulator | sulfur transfer complex subunit TusD | YheO-like PAS domain | YheO-like | YheO-like  (BLOCKS) |
|  | **P44017** | sulfur relay protein TusB/DsrH | sulfur transfer complex subunit TusD | DsrH like protein | Sulphur relay, TusB/DsrH | DsrE-like protein  (BLOCKS) |
|  | **P44018** | C4-dicarboxylate ABC transporter | Hydrolase | C4-dicarboxylate anaerobic carrier | C4-dicarboxylate anaerobic carrier | C4-dicarboxylate anaerobic carrier  (BLOCKS) |
|  | **P44019** | C4-dicarboxylate ABC transporter | Hydrolase | C4-dicarboxylate anaerobic carrier | No result | C4-dicarboxylate anaerobic carrier  (BLOCKS) |
|  | **P44023** | C4-dicarboxylate anaerobic carrier DcuC | carbamate kinase | C4-dicarboxylate anaerobic carrier | C4-dicarboxylate anaerobic carrier | C4-dicarboxylate anaerobic carrier  (BLOCKS) |
|  | **P44771** | hydrolase (HAD superfamily) | camphor resistance protein CrcB | Haloacid dehalogenase-like hydrolase | Cof protein like hydrolase | Cof protein  (BLOCKS) |
|  | **P44782** | 23S rRNA/tRNA pseudouridine synthase A | ATP-dependent helicase HepA | RNA pseudouridylate synthase | Pseudouridine synthase, RsuA/RluB/C/D/E/F | Pseudouridine synthase, Rlu  (BLOCKS) |
|  | **P44025** | TPR repeat protein | single-stranded-DNA-specific exonuclease | Flavinator of succinate dehydrogenase | Flavinator of succinate dehydrogenase | NAD-dependent epimerase/dehydratase  (BLOCKS) |
|  | **P44026** | chloride transporter, ClC family | tRNA-dihydrouridine synthase A | Voltage gated chloride channel | Chloride channel, voltage gated | Chloride channel signature  (BLOCKS) |
|  | **P44027** | tRNA-dihydrouridine synthase A | tryptophanyl-tRNA synthetase | Acyl-CoA dehydrogenase NM domain-like | Conserved hypothetical protein CHP00743 | NADH dehydrogenase (ubiquinone), chloroplast chain  (BLOCKS) |
|  | **P44796** | lysogenization regulator | adenylosuccinate lyase | High frequency lysogenization protein HflD | High frequency lysogenization protein HflD | Protein of unknown function DUF489  (BLOCKS) |
|  | **P44028** | aspartate-semialdehyde dehydrogenase/ periplasmic lipoprotein | 2,3-deoxy-D-manno-octulosonic-acid transferase | Aquaporin-like | Protein of unknown function DUF1375 | Prokaryotic membrane lipoprotein lipid attachment site profile  (PROSITE PROFILE) |
|  | **P44807** | tRNA(ANN) t(6)A37 threonylcarbamoyladenosine modification protein | DNA processing chain A | tRNA threonylcarbamoyladenosine biosynthesis protein RimN | tRNA threonylcarbamoyladenosine biosynthesis protein RimN (YrdC) | Sua5/YciO/YrdC/YwlC protein family  (BLOCKS) |
|  | **P46494** | DNA topoisomerase | DNA topoisomerase III | Topoisomerase DNA binding C4 zinc finger | DNA Topoisomerase ,type IA ,zn finger | Topoisomerase I, zinc-ribbon-like  (BLOCKS) |
|  | **P44031** | Phage-related protein | putative HTH-type transcriptional regulator | Phage derived protein Gp49-like | Addiction module killer protein | Phage NinH  (BLOCKS) |
|  | **P44033** | HipA like kinase | intracellular septation protein A | HipA-like N-terminal domain | HipA-like C-terminal domain | HipA-like, C-terminal  (BLOCKS) |
|  | **P44034** | Flavodoxin | glycerol-3-phosphate acyltransferase | (Trans)glycosidases | HipA-like N-terminal | Tubulin-beta mRNA autoregulation signal  (PROSITE PATTERN) |
|  | **O86228** | HTH-type transcriptional regulator | fructose 1,6-bisphosphatase II | Helix-turn-helix XRE-family like proteins | Helix-turn-helix | Cro/C1-type HTH domain profile  (PROSITE PROFILE) |
|  | **P44812** | cell division protein ZapB | cell division protein FtsZ | Cell division protein ZapB | Cell division protein ZapB | Tumour necrosis factor alpha (cachectin) signature  (BLOCKS) |
|  | **P44036** | acyl-CoA N-acyltransferase | site-specific tyrosine recombinase XerC | Acyl-CoA N-acyltransferases (Nat) | GNAT domain | Gcn5-related N-acetyltransferase (GNAT) domain profile  (PROSITE PROFILE) |
|  | **P71356** | triose-phosphate Transporter family protein | sn-glycerol-3-phosphate dehydrogenase subunit A | EamA-like transporter | Drug/metabolite transporter | Drug/metabolite transporter  (PRODOM) |
|  | **P44037** | sn-glycerol-3-phosphate dehydrogenase subunit A | glycerophosphodiester phosphodiesterase | C-terminal domain of transcriptional repressors | No result | DM DNA-binding domain (BLOCKS) |
|  | **P44827** | ribosomal large subunit pseudouridine synthase E | lipoprotein E | RNA pseudouridylate synthase | Pseudouridine synthase, RsuA/RluB/E/F | Pseudouridine synthase, Rsu  (BLOCKS) |
|  | **Q57523** | tubulin binding protein | Exopolyphosphatase | Lipocalins superfamily | Family of unknown function (DUF490) | Gramicidin channels (PRODOM) |
|  | **P44038** | bacterial surface antigen protein | Exopolyphosphatase | Surface antigen | Bacterial surface antigen (D15) | Bacterial surface antigen (D15)  (BLOCKS) |
|  | **P44831** | Regulator of ribonuclease activity B | monofunctional biosynthetic peptidoglycan transglycosylase | Regulator of ribonuclease activity B | Regulator of ribonuclease activity B | E2 early regulatory protein, C-terminal  (BLOCKS) |
|  | **P44040** | outer membrane antigenic lipoprotein B | stationary phase survival protein SurE | ALDH-like | Prokar_lipoprotein | Prokaryotic membrane lipoprotein lipid attachment site profile  (PROSITE PROFILE) |
|  | **P71357** | bifunctional antitoxin/transcriptional repressor RelB | virulence-associated protein A | RelB antitoxin | RelB antitoxin/Antitoxin DinJ | RelB antitoxin  (BLOCKS) |
|  | **P44041** | Toxin RelE | virulence-associated protein D | Plasmid stabilisation system protein  RelE/ParE | Toxin-antitoxin system, YafQ-like toxin | Conserved hypothetical protein 53  (BLOCKS) |
|  | **P44839** | Endoribonuclease L-PSP | DNA-directed RNA polymerase subunit omega | Endoribonuclease L-PSP | Enamine/imine deaminase YjgF-like | Endoribonuclease L-PSP (BLOCKS) |
|  | **P44842** | Elongation factor | Trk system potassium uptake protein TrkH | DNA-glycosylase | Translation elongation factor EFG, V domain | Ribosomal protein L34e, C-terminal  (BLOCKS) |
|  | **P44844** | Aminotransferase | replication initiation regulator SeqA | PLP-dependent transferases | Der GTPase activator | Ubiquitin thiolesterase, family 2  (BLOCKS) |
|  | **P44043** | coproporphyrinogen III oxidase | transcriptional repressor protein MetJ | Cytochrome c oxidase subunit I-like | Protein of unknown function (DUF2489) | Coproporphyrinogen Oxidase III  (PRODOM) |
|  | **P44045** | cell division protein FtsQ | truncated suppressor of FtsI protein | Fibre shaft of virus attachment proteins | No result | D-arabinono-1,4-lactone oxidase (BLOCKS) |
|  | **P44047** | DNA polymerase III subunit alpha | bifunctional 2',3'-cyclic nucleotide 2'-phosphodiesterase/3'-nucleotidase periplasmic precursor protein | N-terminal nucleophile aminohydrolases (Ntn hydrolases) | No result | Glycoside hydrolase, family 34 (BLOCKS) |
|  | **P44854** | rhodanese-related sulfurtransferase | preprotein translocase subunit SecB | Rhodanese/Cell cycle control phosphatase | Rhodanese-like domain | Rhodanese-like (BLOCKS) |
|  | **P44863** | polysaccharide deacetylase | putative DNA-binding/iron metalloprotein/AP endonuclease | Divergent polysaccharide deacetylase | Polysaccharide deacetylase | Haloacid dehalogenase/epoxide hydrolase family sig  (BLOCKS) |
|  | **P44864** | peptidase M23 family | cell division protein FtsX | zinc metallopeptidases | Peptidase M23 | Peptidase M23/M37  (BLOCKS) |
|  | **P44048** | Fe(2+)-trafficking protein | A/G-specific adenine glycosylase | Bacterial Fe(2+) trafficking | Fe(II) trafficking protein YggX | Fe(II) trafficking protein  yggx protein  (PRODOM) |
|  | **P44050** | diadenosine tetraphosphatase and murein transglycosylase C | nicotinamide-nucleotide adenylyltransferase | Calcineurin-like phosphoesterase | Calcineurin-like phosphoesterase | Phosphoesterase  (PRODOM) |
|  | **P44869** | 16S rRNA m(2)G966 methyltransferase | putative RNA methyltransferase | Ribosomal RNA small subunit methyltransferase D | RNA methyltransferase, RsmD | N-6 Adenine-specific DNA methylases signature  (PROSITE PATTERN) |
|  | **P44052** | 30S ribosomal protein S17 | 50S ribosomal protein L14 | DBL homology domain (DH-domain) | No result | Bindin precursor signature  (BLOCKS) |
|  | **P44053** | cAMP-binding protein-catabolite gene activator and regulatory subunit of cAMP-dependent protein kinase | No predicted functional partner | Cyclic nucleotide-monophosphate binding domain | Cyclic nucleotide-binding domain | cAMP/cGMP binding motif profile  (PROSITE PROFILE) |
|  | **P44054** | sulfite exporter TauE/SafE family protein | thioredoxin | Sulfite exporter TauE/SafE | Transmembrane protein TauE like | C.elegans Srg family integral membrane protein sig (BLOCKS) |
|  | **P44882** | Chain A, Structure Of Ygfb From Haemophilus Influenzae | aminopeptidase P | Polypeptide chain release factor 2 (RF2) | Uncharacterised protein family (UPF0149) | Uncharacterised protein family (UPF0149)  (BLOCKS) |
|  | **P44056** | beta-methylgalactoside transporter inner membrane component | intracellular septation protein A | Transmembrane region | No result | AmiS/UreI family transporter  (BLOCKS) |
|  | **P44886** | acyl-CoA thioester hydrolase | intracellular septation protein A | Thioesterase/thiol ester dehydrase-isomerase | Thioesterase | Acyl-COA thioester  (PRODOM) |
|  | **P44897** | Chain A, Solution Nmr Structure Of Hi0947 From Haemophilus Influenzae | nucleoid-associated protein NdpA | ATPase domain of HSP90 chaperone/DNA topoisomerase II/histidine kinase | Uncharacterised protein family UPF0352 | Vacuolar ATP synthase subunit S1 (BLOCKS) |
|  | **P44898** | hydrolase of alkaline phosphatase superfamily | nucleoid-associated protein NdpA | Sulphatases EC 3.1.6. | Sulphatase | Sulfatase  (BLOCKS/PRODOM) |
|  | **P44058** | Cytosine deaminase (Hydrolase) | 16S rRNA methyltransferase GidB | N-isopropylammelide isopropyl amidohydrolase | Metallo-dependent hydrolases | N-isopropylammelide isopropyl amidohydrolase  (PRODOM) |
|  | **P44059** | Cytosine deaminase | 16S rRNA methyltransferase GidB | Metallo-dependent hydrolases | Unintegrated | Glycoside hydrolase, family 8  (BLOCKS) |
|  | **P44900** | YihD protein | periplasmic oxidoreductase | Chelatase | Protein of unknown function (DUF1040) | Chaperonin clpA/B (PRODOM) |
|  | **P31811** | YifE like protein | dsDNA-mimic protein | Protein of unknown functionDUF413 | Protein of unknown functionDUF413 | transcriptional regulator with pssR  (PRODOM) |
|  | **P44903** | multidrug or homocysteine efflux system | multidrug resistance protein A | Major Facilitator transporter | Drug resistance transporter EmrB/QacA | Phosphopantetheine attachment site  (PROSITE PATTERN) |
|  | **P44904** | tRNA (uracil-5-)-methyltransferase | DNA polymerase I | AD(P)-binding Rossmann-fold domains | Unintegrated | Homoserine dehydrogenase  (BLOCKS) |
|  | **P44062** | cell division protein ZapA | cell division protein FtsZ | Cell division protein ZapA | Cell division protein ZapA-like | Cell divisionFtsK/SpoIIIE protein  (BLOCKS) |
|  | **P44905** | 5-formyltetrahydrofolate cyclo-ligase | FolD bifunctional protein | 5-formyltetrahydrofolate cyclo-ligase | 5-formyltetrahydrofolate cyclo-ligase | 5-formyltetrahydrofolate cyclo-ligase  (BLOCKS) |
|  | **P44908** | preQ0 transporter | PP-loop superfamily protein | alpha-ketoacid dehydrogenase kinase, N-terminal domain | Integral membrane protein | YHHQ transmembrane transporter protein  (PRODOM) |
|  | **P44063** | lipopolysaccharide biosynthesis protein WzzE | lipopolysaccharide biosynthesis protein | Lipopolysaccharide biosynthesis protein WzzE | Unintegrated | Lipopolysaccharide biosynthesis  (BLOCKS) |
|  | **Q57022** | glycosyl transferase family A | undecaprenyl-phosphate galactosephosphotransferase | Glycosyl transferase | Glycosyl transferase | Glycosyl transferase  (BLOCKS) |
|  | **P44064** | Glycosyltransferase | lipopolysaccharide biosynthesis protein | Glycosyl transferase | Glycosyl transferase | Glycosyl transferase  (BLOCKS) |
|  | **P44065** | Glycosyltransferase | lipopolysaccharide biosynthesis protein | No result | Unintegrated | Core binding factor  (BLOCKS) |
|  | **P44067** | O-antigen ligase WaaL | lipopolysaccharide biosynthesis protein | O-Antigen ligase | O-Antigen ligase related | O-antigen polymerase (PRODOM) |
|  | **P71360** | putative multidrug resistance efflux transporter EmrE | GTPase ObgE | Small multidrug resistant protein | Drug/metabolite transporter | Small multidrug resistant protein  (BLOCKS) |
|  | **P44068** | No result | S-adenosylmethionine : tRNA ribosyltransferase isomerase | PLP-dependent transferases | Protein of unknown function DUF208 | O-methyltransferase , family 3 |
|  | **P44069** | bifunctional phosphoribosylaminoimidazolecarboxamide formyltransferase/IMP cyclohydrolase | thiol:disulfide interchange protein precursor | DoxX like | Uncharacterized protein YphA | Ribosomal protein S6e signature  (PROSITE PATTERN) |
|  | **P44070** | Sulfite exporter TauE/SafE | prolipoprotein diacylglyceryl traneferase | Sulfite exporter TauE | Sulfite exporter TauE like | Keratin, high-sulphur matrix protein (BLOCKS) |
|  | **P44931** | tRNA-specific adenosine deaminase | Thymidylate synthase | Cytidine and doxycytidylate zinc binding region | Cytidine deaminase | Cytidine and doxycytidylate zinc binding region  (BLOCKS) |
|  | **P44072** | No result | uridylate kinase | Prokaryotic type KH domain (KH-domain type II) | Protein of unknown function DUF721 | Transcription factor CBF/NF-Y/archeal histone like  (BLOCKS) |
|  | **P44073** | No result | preprotein translocase subunit SecA | RuBisCo,Cterminal domain | Protein of unknown function DUF2547 | Glycoside hydrolase, family 34 (BLOCKS) |
|  | **P44074** | SAM dependent methyltransferase | Glutathione-regulated potassium efflux system protein | S-adenosylmethionine dependent methytransferase | Methytransferase type 11 | Methytransferase type 11  (BLOCKS) |
|  | **P44936** | zinc metallopeptidase RseP | protective surface antigen | PDZ Domain like | Peptidase M50 , putative membrane associated zinc metallopeptidase | Peptidase M50  (BLOCKS) |
|  | **P44938** | Undecaprenyl pyrophosphate synthetase | CDP-diglyceride synthetase | Undecaprenyl diphosphate synthase | Undecaprenyl diphosphate synthase-like | Undecaprenyl pyrophosphate synthase signature  (PROSITE PATTERNS) |
|  | **P44075** | type I restriction enzyme M protein | glutaredoxin-related protein | SIS domain  (Sugar ISomerase) | Domain of unknown function DUF559 | Inositol-3-phosphate synthase  (Isomerase)  (BLOCKS) |
|  | **P44076** | glutaredoxin-like protein | glycyl-tRNA synthetase subunit alpha | No result | Glutaredoxin related protein | Glutaredoxin  (BLOCKS) |
|  | **P44940** | glutathionylspermidine synthase | F0F1 ATP synthase subunit A | Bifunctional glutathionylspermidine synthetase/amidase | Glutathionylspermidine synthase, pre-ATP-grasp-like domain | Glutathionylspermidine synthase |
|  | **P44077** | Lipoprotein | phosphopyruvate hydratase | Protein kinase-like (PK-like) | Prokar_lipoprotein | Prokaryotic membrane lipoprotein lipid attachment site profile  (PROSITE PROFILE) |
|  | **P44078** | glycyl-tRNA synthetase subunit alpha | S-ribosylhomocysteinase | N-terminal nucleophile aminohydrolases (Ntn hydrolases) | Uncharacterised conserved protein UCP007050, HI0931 | Glycoside hydrolase  (BLOCKS) |
|  | **P44941** | FAD/NAD(P)-binding oxidoreductase | cytochrome C-type biogenesis | Pyridine nucleotide-disulphide oxidoreductase | FAD/NAD(P)-binding domain | Flavoprotein dehydrogenases/ oxidoreductase  (PRODOM) |
|  | **P44079** | Type II secretory pathway, pseudopilin | exodeoxyribonuclease V gamma chain | Metallo-dependent phosphatases | Pili subunits | Bacterial type II secretion system protein I/J  (BLOCKS) |
|  | **P44080** | Type II secretory pathway, pseudopilin | extragenic suppressor | Class II aaRS and biotin synthetases | Prepilin peptidase-dependent protein B | Leucyl-tRNA synthetase signature (BLOCKS) |
|  | **P44081** | Putative type II secretory pathway, pseudopilin | exodeoxyribonuclease V gamma chain | alpha/beta-Hydrolases | Protein of unknown function DUF2572 | Pneumovirus attachment glycoprotein G  (BLOCKS) |
|  | **P44082** | Putative type II secretory pathway, pseudopilin | exodeoxyribonuclease V gamma chain | p53-like transcription factors | Uncharacterised protein family HI0941 | Bacterial type II secretion system protein I/J  (BLOCKS) |
|  | **Q57120** | Antitoxin | L-2,4-diaminobutyrate decarboxylase | Antidote-toxin recognition MazE | AbrB-like domain | SpoVT/AbrB-like domain  (BLOCKS) |
|  | **P44954** | phosphopyruvate hydratase | cAMP-regulatory protein | NAD(P)-linked oxidoreductase | YehU-like | YehU-like  (PRODOM) |
|  | **P44084** | periplasmic lipoprotein | beta-hexosaminidase | Protein of unknown function (DUF1425) | Prokar_lipoprotein | Prokaryotic membrane lipoprotein lipid attachment site profile  (PROSITE PROFILE) |
|  | **P44085** | bifunctional riboflavin kinase/FMN adenylyltransferase | naphthoate synthase | No result | Prokar_lipoprotein | Prokaryotic membrane lipoprotein lipid attachment site profile  (PROSITE PROFILE) |
|  | **P44086** | No result | No predicted association | No result | No result | Lantibiotic regulatory protein signature  (BLOCKS) |
|  | **Q57133** | transferrin-binding protein | acetyl-CoA carboxylase biotin carboxylase subunit | Transferrin binding protein-like solute binding protein | Solute-binding protein, transferrin-binding protein-like | Transferrin binding protein  (BLOCKS) |
|  | **P46455** | sodium/panthothenate symporter | ribosomal protein L11 methyltransferase | Methyl-coenzyme M reductase alpha and beta chain C-terminal domain | Protein of unknown function (DUF997) | Protein of unknown function DUF1673, Methanosarcin (BLOCKS) |
|  | **Q57147** | EamA-like transporter family protein | cell filamentation protein | EamA-like transporter | Drug/metabolite transporter EamA | CDR ABC transporter  (BLOCKS) |
|  | **O86230** | sodium/panthothenate symporter | cell filamentation protein | EamA-like transporter | Drug/metabolite transporter EamA | Sulphate transporter  (BLOCKS) |
|  | **P44965** | tRNA-dihydrouridine synthase | DNA-binding protein Fis | tRNA-dihydrouridine synthase | tRNA-dihydrouridine synthase | tRNA-dihydrouridine synthase  (PRODOM) |
|  | **P43907** | 6-phosphofructokinase | transcriptional repressor | Class II aaRS and biotin synthetases | Prokar_lipoprotein | Prokaryotic membrane lipoprotein lipid attachment site profile  (PROSITE PROFILE) |
|  | **P43908** | YaaA-like protein | DNA processing chain A | alpha-catenin/vinculin-like | Peroxide stress response protein YAAA | Peroxide stress response protein YAAA  (PRODOM) |
|  | **P44972** | ribonuclease P | putative inner membrane protein translocase component YidC | Ribonuclease H-like | Haemolytic domain | YIDD alpha-hemolysin  (PRODOM) |
|  | **P44974** | phosphoethanolamine transferase | lysyl-tRNA synthetase | Sulphatases EC 3.1.6. are enzymes that hydrolyze various sulphate esters | Sulfatase | Transmembrane hydrolase protein  (PRODOM) |
|  | **Q57134** | competence protein ComE | protein transport protein | Helix-hairpin-helix DNA-binding motif | Competence protein ComEA, helix-hairpin-helix domain | Helix-hairpin-helix motif, class 2  (BLOCKS) |
|  | **P44093** | 4-hydroxy-3-methylbut-2-enyl diphosphate reductase | 3-hydroxyisobutyrate dehydrogenase | Aminoacid dehydrogenase-like, N-terminal domain | D-Tagatose-1,6-bisphosphate aldolase | Glycosyl hydrolases family 1 active site  (PROSITE PATTERN) |
|  | **Q57151** | hydroxypyruvate isomerase | 3-hydroxyisobutyrate dehydrogenase | Xylose isomerase-like TIM barrel | Xylose isomerase-like, TIM barrel domain | Hydroxypyruvate isomerase  (PRODOM) |
|  | **P44094** | NAD-dependent epimerase/dehydratase | 3-hydroxyisobutyrate dehydrogenase | Nucleoside-diphosphate-sugar epimerase | NAD-dependent epimerase/dehydratase | Nucleoside-diphosphate-sugar epimerase  (PRODOM) |
|  | **P44095** | cyclase family protein | gluconate permease | Putative cyclase | Putative cyclase | Glucose transporter type 3 (GLUT3) signature  (BLOCKS) |
|  | **P44992** | TRAP dicarboxylate transporter subunit DctP | 2,3-diketo-L-gulonate reductase | Sialic acid-binding periplasmic protein SiaP | TRAP dicarboxylate transporter | TRAP dicarboxylate transporter- DctP subunit  (BLOCKS) |
|  | **P44993** | C4-dicarboxylate ABC transporter permease | 2,3-diketo-L-gulonate reductase | DctM-like transporters (TRAP system permease) | TRAP transporter permease protein | TRAP C4-dicarboxylate transport system permease D  (BLOCKS) |
|  | **P44994** | C4-dicarboxylate ABC transporter permease | 2,3-diketo-L-gulonate reductase | Tripartite ATP-independent periplasmic transporters, DctQ component | Tripartite ATP-independent periplasmic transporters, DctQ component | Tripartite ATP-independent periplasmic transporters, (BLOCKS) |
|  | **P44097** | YGGT family protein | magnesium/nickel/cobalt transporter CorA | Carbamoyl phosphate synthetase, large subunit connection domain | YGGT like protein | Cytochrome c oxidase, subunit I  (BLOCKS) |
|  | **P44098** | glutamine amidotransferase | transcriptional regulator NrdR | Glutamine amidotransferases class-II | Glutamine amidotransferases typr-2 domain | Glutamine amidotransferase, class-II  (BLOCKS) |
|  | **P44099** | AAA+ superfamily ATPase | N-acetylmuramoyl-L-alanine amidase | AAA ATPase | P-loop containing nucleoside triphosphate hydrolases | Prokaryotic ATPase  (BLOCKS) |
|  | **P44103** | transglutaminase family protein | mercuric ion transport protein | Transglutaminase/protease-like homologues | Transglutaminase -like | Bacterial transglutaminase-like, N-terminal  (BLOCKS) |
|  | **Q57498** | alkylhydroperoxidase AhpD core | AraC family transcriptional regulator | Carboxymuconolactone decarboxylase | Carboxymuconolactone decarboxylase | Carboxymuconolactone decarboxylase  (BLOCKS) |
|  | **P44104** | Type III restriction-modification system restriction enzyme | type III restriction-modification system endonuclease-like protein | Positive stranded ssRNA viruses | No result | Restriction type iii enzyme lipoprotein endonuclease plasmid system: methyltransferase membrane  (PRODOM) |
|  | **P44106** | type III restriction/modification enzyme methylation subunit | type III restriction-modification system methyltransferase-like protein | Type III restriction/modification enzyme methylation subunit | Type III restriction/modification enzyme methylation subunit | Type III restriction/modification enzyme methylation subunit  (PRODOM) |
|  | **P44107** | PE--lipooligosaccharide phosphorylethanolamine transferase | UDP-3-O-[3-hydroxymyristoyl] N-acetylglucosamine deacetylase | Upper collar protein gp10 (connector protein) | Uncharacterised domain HI1063, transferase-like | D12 class N6 adenine-specific DNA methyltransferase  (BLOCKS) |
|  | **P71367** | PE--lipooligosaccharide phosphorylethanolamine transferase | uridylate kinase | Sulphatases EC 3.1.6., enzymes that hydrolyze various sulphate esters. | Sulphatase | Transmembrane hydrolase protein  (PRODOM) |
|  | **P45019** | CTP synthetase | putative nucleotide-binding protein | Dehydroquinate synthase-like | Protein of unknown function DUF423 | Protein of unknown function DUF423  (BLOCKS) |
|  | **P44110** | membrane protein | transcriptional regulator NrdR | Protein of unknown function (DUF441) | Protein of unknown function (DUF441) | Cytochrome c biogenesis protein, transmembrane region  (BLOCKS) |
|  | **P45026** | BolA family transcriptional regulator (DNA binding) | UDP-N-acetylglucosamine 1-carboxyvinyltransferase | BolA-like protein | BolA-like protein | BolA-like protein  (BLOCKS) |
|  | **P44111** | No result | HP HI1099 | Ribokinase-like | No result | Salmonella type III secretion SopE effector protein like  (BLOCKS) |
|  | **P44112** | Lipoprotein | 50S ribosomal protein L20 | Prokaryotic membrane lipoprotein lipid attachment site | Prokar_lipoprotein | Prokaryotic membrane lipoprotein lipid attachment site profile  (PROSITE PROFILE) |
|  | **P45071** | glmZ(sRNA)-inactivating NTPase | nitrogen regulatory IIA protein | P-loop ATPase protein | P-loop containing ATPase protein | P-loop ATPase protein family  (BLOCKS) |
|  | **P45074** | sugar ABC transporter substrate-binding protein | Phosphatase | OstA-like protein | Lipopolysaccharide assembly, LptA | OstA-like protein  (BLOCKS) |
|  | **P45075** | sugar transporter | Phosphatase | N-terminal nucleophile aminohydrolases (Ntn hydrolases) | Lipopolysaccharide assembly, LptC-related | Exported transmembrane YRBK like protein  (PRODOM) |
|  | **P45076** | ribosome-associated, YjgA family protein | anaerobic ribonucleotide reductase-activating protein | Aldehyde ferredoxin oxidoreductase, C-terminal domains | Ribosome-associated, YjgA | Microtubule atp-binding  YjgA  (PRODOM) |
|  | **P45077** | peptidase PmbA | hypoxanthine-guanine phosphoribosyltransferase | Putative modulator of DNA gyrase | TldD/PmbA , Putative modulator of DNA gyrase | Modulator of DNA gyrase |
|  | **P45083** | acyl-CoA esterase | Ferrochelatase | Thioesterase | Thioesterase | Phenylacetic acid degradation related  (PRODOM) |
|  | **P44116** | DNA (cytosine-5-)-methyltransferase | HP HI1163 | Bacterial enterotoxins | Restriction endonuclease type II-like | Protein of unknown function DUF559  (BLOCKS) |
|  | **Q57252** | FAD-linked oxidoreductase | Ferrochelatase | Octameric flavoenzyme vanillyl-alcohol oxidase | Oxidoreductase | FAD linked oxidase, C-terminal  (BLOCKS) |
|  | **P45085** | Glutaredoxin | Thioredoxin | Glutaredoxin-4 | Glutaredoxin | Glutaredoxin  (BLOCKS) |
|  | **P44117** | Hypothetical protein | fructose 1,6-bisphosphatase II | NagB/RpiA/CoA transferase-like | Protein of unknown function DUF496 | Protein of unknown function DUF496  (BLOCKS) |
|  | **P44119** | metallopeptidase, SprT family | putative Na+/alanine symporter | SprT homologues. | SprT homologues. | Zinc metal-binding SPRT metallopeptidase  (PRODOM) |
|  | **P45097** | Methyltransferase (radical SAM protein) | PP-loop superfamily ATPase | Radical SAM | 7-carboxy-7-deazaguanine synthase-like | Radical-activating enzyme  (BLOCKS) |
|  | **P44124** | 7-cyano-7-deazaguanine synthase | organic radical activating protein | 7-cyano-7-deazaguanine synthase  (QueC) | Queuosine biosynthesis protein QueC | PP-loop/ NAD+ synthase  (BLOCKS) |
|  | **P44125** | Lipoprotein | glutaminyl-tRNA synthetase | No result | Prokar_lipoprotein | Prokaryotic membrane lipoprotein lipid attachment site profile  (PROSITE PROFILE) |
|  | **P45103** | Sua5/YciO/YrdC/YwlC family protein | dihydroxy-acid dehydratase | YrdC/RibB like | YrdC like domain | SUA5/yciO/yrdC, N-terminal  (BLOCKS) |
|  | **P45104** | ribosomal large subunit pseudouridine synthase B | 23S rRNA pseudouridine synthase D | Ribosomal small subunit pseudouridine synthase | Pseudouridine synthase, RsuA/RluB/E/F | Pseudouridine synthase, Rsu  (BLOCKS) |
|  | **P44126** | Smr protein/MutS2 | thioredoxin reductase | Small MutS-related domain | Smr protein/MutS2 C-terminal | Smr protein/MutS2 C-terminal  (BLOCKS) |
|  | **P44127** | Membrane protein | transcriptional repressor protein MetJ | Agglutinin | Uncharacterised protein family UPF0208 | Transmembrane YFBV protein  (PRODOM) |
|  | **P71373** | Amidophosphoribosyltransferase (Epimerase) | arginine repressor | NAD dependent epimerase/dehydratase | NAD dependent epimerase/dehydratase | NAD-dependent epimerase/dehydratase  (BLOCKS) |
|  | **P44129** | 30S ribosomal protein S1 | tetratricopeptide repeat protein | Light-harvesting complex subunits | Protein of unknown function DUF1049 | C4-dicarboxylate transporter/malic acid transport (BLOCKS) |
|  | **P44131** | gamma-glutamyl phosphate reductase | molecular chaperone DnaK | Carbonic anhydrase | Protein of unknown function DUF945 | EAG potassium channel signature  (BLOCKS) |
|  | **P44132** | gamma-glutamyl phosphate reductase | monofunctional biosynthetic peptidoglycan transglycosylase | PLP-dependent transferases | Protein of unknown function DUF945 | YDGA exported gtp-binding transmembrane  Protein  (PRODOM) |
|  | **P45122** | bicyclomycin/multidrug efflux system protein | gamma-glutamyl phosphate reductase | (Trans)glycosidases | Uncharacterised domain UPF0126 | Uncharacterised domain UPF0126 |
|  | **P44133** | gamma-glutamyl phosphate reductase | Lipoprotein | Actin-like ATPase domain | No result | Transmembrane hi1241 |
|  | **P44134** | polysaccharide biosynthesis protein | 16S pseudouridylate 516 synthase | Starch-binding domain-like | Protein of unknown function DUF1919 | HI1244 homolog uncharacterized  Protein  (PRODOM) |
|  | **P44135** | phosphoglycerol transferase-like protein | diacylglycerol kinase | Sulphatases EC 3.1.6. are enzymes that hydrolyze various sulphate esters | Sulphatase | Sulphatase  (BLOCKS) |
|  | **P44136** | nickel/cobalt transporter  (ABC-type transport system) | thiol-disulfide interchange protein | High-affinity nickel-transport protein | Nickel/cobalt transporter, high-affinity | High-affinity nickel-transport protein  (BLOCKS) |
|  | **P44137** | ABC-type transport system, periplasmic component | disulfide interchange protein | N-terminal nucleophile aminohydrolases (Ntn hydrolases) | Protein of unknown function DUF1007 | Cleaved adhesion  (BLOCKS) |
|  | **P44138** | plasmid maintenance system killer protein | virulence-associated protein A | Plasmid maintenance system killer protein | Plasmid maintenance system killer | Influenza matrix protein (M1)  (BLOCKS) |
|  | **P44139** | invasion protein expression up-regulator SirB | putative ABC transporter ATP-binding protein | Invasion gene expression up-regulator, SirB | Invasion gene expression up-regulator, SirB | Invasion gene expression up-regulator, SirB  (BLOCKS) |
|  | **P44140** | tRNA(Met) cytidine acetyltransferase | No predicted association | Acyl-CoA N-acyltransferases (Nat) | tRNA(Met) cytidine acetyltransferase TmcA | Gcn5-related N-acetyltransferase (GNAT) domain profile  (PROSITE PROFILE) |
|  | **Same as 270** | - | - | - | - | - |
|  | **Same as 270** | - | - | - | - | - |
|  | **P44144** | YcaO protein | recombination protein | YcaO-like protein | YcaO-like | YcaO-like  (PRODOM) |
|  | **P44145** | chemotaxis protein methyltransferase | N-acetylmuramoyl-L-alanine amidase | Transmembrane | No result | Transmembrane Glycosyl transferase, family 8  (BLOCKS) |
|  | **No result** | - | - | - | - | - |
|  | **P44148** | ABC transporter substrate-binding protein | HP HI1268 | No result | No result | KCNQ2 voltage-gated potassium channel signature  (BLOCKS) |
|  | **P44150** | type 11 methyltransferase | ABC transporter ATP-binding protein | cobalt-precorrin-6Y C(15)-methyltransferase | S-adenosyl-L-methionine-dependent methyltransferases | Ribosomal RNA adenine dimethylase/  Methytransferase  (PRODOM) |
|  | **P45138** | ribosome maturation protein RimP | transcription elongation factor NusA | Ribosome maturation factor RimP | Ribosome maturation factor RimP | Domain of unknown function DUF150  (BLOCKS) |
|  | **P44154** | Zn-ribbon-containing protein (DNA binding protein) | putative selenocysteine lyase | "Helical backbone" metal receptor | Predicted Zinc-ribbon-containing protein | Predicted Zinc-ribbon-containing protein  (PRODOM) |
|  | **P44156** | SufE protein probably involved in Fe-S center assembly | putative selenocysteine lyase | Fe-S metabolism associated SufE | Fe-S metabolism associated domain , SufE | Fe-S metabolism associated SufE  (PRODOM) |
|  | **P45145** | Membrane protein | Exonuclease I | LrgA like protein  (Export murein hydrolases) | LrgA family protein | LrgA family protein  (BLOCKS) |
|  | **P45146** | murein hydrolase regulator LrgB | excinuclease ABC subunit B | LrgB-like protein | LrgB-like protein | LrgB-like protein  (BLOCKS) |
|  | **Q57320** | threonine efflux system | phosphatidylglycerophosphatase A | LysE type translocator | Lysine-type exporter protein (LYSE/YGGA) | Lysine exporter protein (LYSE/YGGA)  (BLOCKS) |
|  | **P45154** | (2Fe-2S)-binding protein | ribonucleotide-diphosphate reductase subunit beta | Ferredoxin-1 | 2Fe-2S ferredoxin-type domain | 2Fe-2S binding ferredoxin-like  (BLOCKS) |
|  | **P44158** | cytochrome C oxidase | peptide deformylase | (Trans)glycosidases | No result | Glycoside hydrolase, family 10  (BLOCKS) |
|  | **P71375** | AraC family transcriptional regulator | sulfatase-like protein | Sodium:solute symporter | Sodium/solute symporter | Na+/solute symporter  (BLOCKS) |
|  | **P44160** | glucose-6-phosphate 1-epimerase | glucose-6-phosphate isomerase | Aldose 1-epimerase | Galactose mutarotase-like | Aldose 1-epimerase  (BLOCKS) |
|  | **P44161** | Ter macrodomain organizer matS-binding protein | condesin subunit F | Macrodomain Ter protein, MatP | Macrodomain Ter protein, MatP | Kinetoplastid membrane protein 11  (BLOCKS) |
|  | **P44162** | 30S ribosomal protein S15 | diacylglycerol kinase | No result | Protein kinase-like domain | Calcium/calmodulin dependent protein kinase II  (BLOCKS) |
|  | **P44163** | No result | 30S ribosomal protein S15 | Transmembrane | Prokar_lipoprotein | Prokaryotic membrane lipoprotein lipid attachment site profile  (PROSITE PROFILE) |
|  | **P71376** | 23S rRNA methyltransferase J (RNA-binding protein) | ribosomal RNA large subunit methyltransferase J | RNA-binding protein | RNA-binding, CRM domain | CRM domain profile  (PROSITE PROFILE) |
|  | **P44164** | phosphohistidine phosphatase SixA | glmM1 HI1463,MrsA | Phosphohistidine phosphatase SixA | Phosphohistidine phosphatase SixA | Carbamoyl-phosphate synthase (BLOCKS) |
|  | **P71378** | molecular chaperone | HP HI1338 | Late embryogenesis abundant protein | Late embryogenesis abundant protein | Late embryogenesis abundant protein  (BLOCKS) |
|  | **P44165** | Outer membrane efflux porin TdeA | hemoglobin-binding protein | NADPH-dependent 7-cyano-7-deazaguanine reductase | Outer membrane efflux protein | Cyclohydrolase yqcd i i-like similar enzyme  (PRODOM) |
|  | **P71379** | cysteine desulfurase, catalytic subunit CsdA | hemoglobin-binding protein | Cysteine desulfurase | Aminotransferase, class V/Cysteine desulfurase | Aminotransferase, class V  (BLOCKS) |
|  | **P45173** | DNA-binding ferritin-like protein | DNA-binding transcriptional regulator OxyR | Ferritin-like protein | DNA-binding ferritin like protein Dps | DNA-binding protein Dps  (BLOCKS) |
|  | **P44167** | tRNA mo(5)U34 methyltransferase, SAM-dependent | S-adenosyl-L-methionine-dependent methyltransferase | Protein arginine N-methyltransferase 3 | tRNA (mo5U34)-methyltransferase | Methyltransferase type 11  (BLOCKS) |
|  | **P44168** | flagellin N-methylase family protein | ribonuclease D | Eukaryotic type KH-domain (KH-domain type I) | Uncharacterised protein family UPF0153 | Protein of unknown function UPF0153 |
|  | **P45180** | glycogen phosphorylase | glycogen branching enzyme | Glycogen phosphorylase, liver form | Glycosyl transferase, family 35 | Glycosyl transferase, family 35  (BLOCKS) |
|  | **P45182** | TonB-dependent receptor | putative zinc protease | TonB-dependent Receptor Plug Domain | TonB-dependent receptor, beta-barrel | TonB-dependent receptor  (PRODOM) |
|  | **P44169** | putative DNA binding protein | Exonuclease I | Frataxin/Nqo15-like | No result | Transporter  (PRODOM) |
|  | **P44170** | cell division protein MukB | Exonuclease I | Concanavalin A-like lectins/glucanases | Multidrug resistance efflux transporter EmrE | Small multidrug resistance protein  (BLOCKS) |
|  | **P44171** | Glycosyltransferase | anthranilate synthase component I | AraD/HMP-PK domain-like | No result | Transcription factor Tfb2  (BLOCKS) |
|  | **O86237** | anthranilate phosphoribosyltransferase | anthranilate synthase component II | Tautomerase/MIF | Tautomerase/MIF | 4-oxalocrotonate tautomerase  (BLOCKS) |
|  | **P44172** | ASCH domain protein | valyl-tRNA synthetase | RNA binding domain  (ASCH) | ASCH domain | Uncharacterised conserved protein UCP029143  (BLOCKS) |
|  | **P44173** | zeta toxin family protein | glucosamine--fructose-6-phosphate aminotransferase | Zeta toxin | Zeta toxin | ATPase AAA-2  (BLOCKS) |
|  | **P44175** | dihydroorotate dehydrogenase 2 | fumarate hydratase | aspartate decarboxylase, ADC like | No result | Bacterial protein of unknown function DUF903  (BLOCKS) |
|  | **P44176** | S-adenosylmethionine tRNA ribosyltransferase | dihydroorotate dehydrogenase 2 | DNA polymerase alpha chain like domain | Polymerase and histidinol phosphatase like | Bacterial DNA polymerase III, alpha subunit  (BLOCKS) |
|  | **P44177** | tail assembly protein | tail fibre protein/phosphomannomutase | No result | Uncharacterised protein family HI1402 | KCNQ2 voltage-gated potassium channel signature  (BLOCKS) |
|  | **P44180** | gp27 putative head protein (Bacteriophage protein) | plasmid RP4 TraN-like protein | ARM repeat  (Armadillo repeat) | Uncharacterised conserved protein UCP029215 | Calcium/calmodulin dependent protein kinase II  (PRODOM) |
|  | **P44181** | No result | Phosphorylase | Concanavalin A-like lectins/glucanases | Protein of unknown function DUF2513 | CRP bacterial regulatory protein HTH signature  (PRINTS) |
|  | **P44183** | HI1409 family phage-associated protein | plasmid RP4 TraN-like protein | TolB, N-terminal domain | Phage-associated protein HI1409 | BACTERIOPHAGE SIMILAR protein with MLR8004 similarities  (NADH dehydrogenasesubunit D)  (PRODOM) |
|  | **P45197** | lytic transglycosylase | terminase small subunit | Phage regulatory protein Rha | Bacteriophage regulatory protein, Rha family | Phage NinH  (BLOCKS) |
|  | **P44185** | putative lytic protein Rz | diaminopimelate decarboxylase | Prokaryotic type I DNA topoisomerase | Prokar_lipoprotein | Prokaryotic membrane lipoprotein lipid attachment site profile  (PROSITE PROFILE)) |
|  | **P44186** | putative lytic protein Rz | terminase small subunit | Tryptophan synthase beta subunit-like PLP-dependent enzymes | Protein of unknown function DUF2570 | Imidazoleglycerol-phosphate dehydratase (BLOCKS) |
|  | **P44187** | Putative phage lysozyme | terminase small subunit | O-Glycosyl hydrolases (EC 3.2.1.) | Lysozyme-like domain | Glycoside hydrolase, family 19  (BLOCKS) |
|  | **P44188** | phage holin, lambda family | fumarate/nitrate reduction transcriptional regulator | Phage holin like | Bacteriophage lambda, GpS, holin | Holin, phage lambda  (BLOCKS) |
|  | **P44189** | prophage antirepressor protein | tail fibre protein/phosphomannomutase | BRO family, N-terminal domain | BRO N-terminal domain | Antirepressor protein  (PRODOM) |
|  | **P44190** | addiction module killer protein | mercuric ion scavenger protein | Phage derived protein Gp49-like | Addiction module killer protein | Alcohol dehydrogenase related protein signature  (BLOCKS) |
|  | **P44191** | putative transcriptional regulator | mercuric ion scavenger protein | Helix-turn-helix protein | Helix-turn-helix protein /Addiction module antidote protein, HI1420 | Putative helix-turn-helix protein, YlxM/p13-like  (BLOCKS) |
|  | **P44193** | antA/AntB antirepressor family protein | tail fibre protein/phosphomannomutase | AntA/AntB antirepressor | AntA/AntB antirepressor | Phage anti repressor protein  (PRODOM) |
|  | **P44194** | helicase domain protein | HI1546,ImpA | "Winged helix" DNA-binding domain | No result | DnaB-like helicase, N-terminal  (BLOCKS) |
|  | **P44196** | ABC-type transport system protein, periplasmic component | ABC transporter ATP-binding protein | Galactose-binding domain-like | Protein of unknown function DUF4198 | Ribosomal protein L19e (BLOCKS) |
|  | **P45202** | Cys-tRNAPro/Cys-tRNACys deacylase ybaK | leucyl-tRNA synthetase | Cys-tRNA(Pro)/Cys-tRNA(Cys) deacylase YbaK  (Hydrolase) | Prolyl-tRNA editing protein, YbaK/EbsC | YbaK/prolyl-tRNA synthetase associated region  (BLOCKS) |
|  | **P56507** | aspartate-semialdehyde dehydrogenase | tRNA pseudouridine synthase C | N-terminal nucleophile aminohydrolases (Ntn hydrolases) | Uncharacterised protein family UPF0181 | Aminotransferase class-III  (BLOCKS) |
|  | **P44197** | tRNA pseudouridine synthase C | thiamine biosynthesis protein ThiI | Ribosomal large subunit pseudouridine synthase C | Pseudouridine synthase, RsuA/RluB/C/D/E/F | Pseudouridine synthase, Rlu  (BLOCKS) |
|  | **Q57152** | RNA pseudouridine synthase C -like domain | penicillin-binding protein 1B | Dihydropteroate synthase-like | Protein of unknown function UCP006257 | Pseudouridine synthase C  (PRODOM) |
|  | **P44198** | dithiobiotin synthetase | thiamin transporter membrane protein | YjgF-like | YcgL domain | YcgL domain profile  (PROSITE PROFILE) |
|  | **P44201** | Membrane protein | potassium transporter peripheral membrane component | Adenylylcyclase toxin (the edema factor) | Protein of unknown function DUF340, prokaryotic membrane | Predicted permease YjgP/YjgQ family  (BLOCKS) |
|  | **P44202** | cytochrome c-type biogenesis protein | phosphoribosylformylglycinamidine synthase | Cytochrome C biogenesis protein transmembrane region | Cytochrome c assembly protein, transmembrane domain | Cytochrome c biogenesis protein, transmembrane reg  (BLOCKS) |
|  | **P44203** | Lipoprotein | opacity protein | No result | Uncharacterised conserved protein UCP012335 | Lipoprotein  (PRODOM) |
|  | **P45217** | Outer membrane efflux porin TdeA | acriflavine resistance protein | Multidrug resistance protein/ Outer membrane efflux proteins (OEP) | RND efflux system, outer membrane lipoprotein, NodT | Prokaryotic membrane lipoprotein lipid attachment site profile  (PROSITE PROFILE) |
|  | **Same as 293** | - | - | - | - | - |
|  | **Same as 292** | - | - | - | - | - |
|  | **P44205** | molybdenum ABC transporter substrate-binding protein | iron chelatin ABC transporter ATP-binding protein | L30e-like ribosomal protein | Molybdate-binding periplasmic protein | Prokaryotic extracellular metal-binding protein  (BLOCKS) |
|  | **Q57380** | molybdate ABC transporter, permease | molybdate-binding periplasmic protein | Molybdate/tungstate abc transporter | Molybdenum transport system permease protein MODB | ABC transporter integral membrane type-1 domain profile  (PROSITE PROFILE) |
|  | **P44208** | Transposase | DNA-binding protein | Transposase | Homeodomain-like | Transposase (probable), IS891/IS1136/IS1341 family  (BLOCKS) |
|  | **P44209** | ATP-dependent RNA helicase RhlE | S-adenosyl-L-methionine-dependent methyltransferase | DNA/RNA-binding 3-helical bundle | Hypothetical protein HI1480 | DNA-binding protein Dps  (BLOCKS) |
|  | **P44210** | adenine glycosylase | host-nuclease inhibitor protein | Immunoglobulin-like beta-sandwich fold | No result | Influenza matrix protein (M1)  (BLOCKS) |
|  | **P44212** | putative bacteriophage protein | HI1488,E16 protein | Phosphoglycerate kinase | Uncharacterised protein family HI1485 | MU-LIKE protein  (PRODOM) |
|  | **P44213** | No result | HI1488, E16 protein | Coiled coil region | No result | MU-LIKE protein  (PRODOM) |
|  | **P44214** | Uracil phosphoribosyltransferase | HI1488, E16 protein | Ferritin-like fold | No result | Transferase family  (BLOCKS) |
|  | **P44215** | No result | HI1488, E16 protein | RING/U-box  Superfamily | No result | Zn-finger, ZPR1 type  (BLOCKS) |
|  | **P44217** | No result | N-acetylmuramoyl-L-alanine amidase | Carbohydrate-binding domain | No result | Glucagon receptor signature  (BLOCKS) |
|  | **P44218** | N-acetylmuramoyl-L-alanine amidase | N-acetylmuramoyl-L-alanine amidase | N-acetylmuramoyl-L-alanine amidase | No result | N-acetylmuramoyl-L-alanine amidase  (PRODOM) |
|  | **P44219** | C4-dicarboxylate ABC transporter | N-acetylmuramoyl-L-alanine amidase | Aquaporin-like | Protein of unknown function DUF2644 | Porin, opacity type  (BLOCKS) |
|  | **P44220** | aTPase histidine kinase-DNA gyrase B-and HSP90-like domain protein | N-acetylmuramoyl-L-alanine amidase | Class I glutamine amidotransferase-like | Uncharacterised protein family HI1496 | Vacuolar ATP synthase 16kD subunit signature  (BLOCKS) |
|  | **P44221** | DNA-binding protein | -acetylmuramoyl-L-alanine amidase | Prokaryotic dksA/traR C4-type zinc finger | Zinc finger, DksA/TraR C4-type | Zn-finger, prokaryotic DksA/TraR C4 type  (BLOCKS) |
|  | **P44222** | Mu-like phage gp25 | N-acetylmuramoyl-L-alanine amidase | Frizzled cysteine-rich domain | Bacteriophage Mu, Gp25 | Bacteriophage lysis protein  (BLOCKS) |
|  | **O86242** | Ribonuclease R winged-helix domain protein | HI1568,G protein | Ribonuclease R winged-helix domain | Ribonuclease R winged-helix domain | GntR bacterial regulatory protein HTH signature  (PRINTS) |
|  | **P44223** | Mu-like phage gp27 | HI1568,G protein | Bacterial muramidases | Bacteriophage Mu, Gp27 | Bacteriophage Mu, Gp27  (PRODOM) |
|  | **P44224** | Mu-like prophage FluMu protein gp28 | HI1568,G protein | Terminase-like | Terminase, large subunit/ Bacteriophage Mu, Gp28 | Bacteriophage Mu, Gp28  (PRODOM) |
|  | **P44225** | Mu-like prophage FluMu protein gp29 | HI1568,G protein | NAD(P)-binding Rossmann-fold domains | Protein of unknown function DUF935 | Bacteriophage Mu, Gp29  (PRODOM) |
|  | **P44226** | F protein, phage head morphogenesis , SPP1 gp7 family domain protein | HI1568,G protein | ADP-ribosylation | Phage head morphogenesis domain | Phage head morphogenesis protein MU like  ,GP30 |
|  | **P44227** | Mu-like prophage FluMu major head subunit | HI1504,I protein | Protease propeptides/inhibitors | Bacteriophage Mu, GpT | Bacteriophage Mu, GpT  (PRODOM) |
|  | **P44228** | Rho termination factor domain protein | HI1504,I protein | Recombination endonuclease VII, C-terminal and dimerization domains | helix-extended loop-helix (HeH)/ LEM domain | Mu-like prophage FluMu protein gp35  (PRODOM) |
|  | **Same as 356** |  | - | - | - | - |
|  | **P44230** | Mu-like prophage protein GP36 | HI1511,sheath protein gpL | Protein of unknown function (DUF1320) | Bacteriophage Mu, Gp36 | Bacteriophage Mu, Gp36  (PRODOM) |
|  | **P44231** | Mu-like prophage FluMu protein gp37 | HI1511,sheath protein gpL | Outer membrane efflux proteins (OEP) | Bacteriophage Mu, Gp37 | Bacteriophage Mu, Gp37  (PRODOM) |
|  | **P44232** | Mu-like prophage FluMu protein gp38 | HI1511,sheath protein gpL | Acid proteases | Protein of unknown function DUF2635 | Phosphotransferase system PTS, lactose/cellobiose-  (BLOCKS) |
|  | **P44234** | Mu-like prophage FluMu tail tube protein | HI1511,sheath protein gpL | Phage tail tube protein | Bacteriophage Mu, GpM, tail tube | Phage tail tube protein  (PRODOM) |
|  | **P44235** | Mu-like prophage FluMu protein gp41 | HI1511,sheath protein gpL | Enolase C-terminal domain-like | Bacteriophage tail protein Gp41, putative | Bacteriophage tail protein Gp41  (PRODOM) |
|  | **P44238** | Mu-like prophage FluMu protein gp45 | HI1511,sheath protein gpL | Bacteriophage Mu Gp45 protein | Bacteriophage Mu/P2, baseplate assembly | Phage baseplate assembly protein V  (BLOCKS) |
|  | **P44239** | Mu-like prophage FluMu protein gp46 | HI1515,64 kDa virion protein | Ubiquitin-like | Bacteriophage Mu, Gp46 | Bacteriophage Mu, Gp46  (PRODOM) |
|  | **P44240** | Mu-like prophage FluMu protein gp47 | HI1511,sheath protein gpL | Baseplate J-like protein | Baseplate assemblt J-like protein ,predicted | Baseplate J-like protein GP47  (PRODOM) |
|  | **P44241** | Mu-like prophage FluMu protein gp48 | HI1515, 64 kDa virion protein | Uncharacterized protein conserved in bacteria (DUF2313) | Bacteriophage Mu, Gp48 | Bacteriophage Mu, Gp48  (PRODOM) |
|  | **P44242** | Mu-like prophage FluMu defective tail fiber protein | host-nuclease inhibitor protein | Pectin lyase-like | No result | Mu-like prophage FluMu defective tail fiber protein (PRODOM) |
|  | **P71390** | Mu-like prophage protein Com | selenocysteine-specific elongation factor | Mu-like prophage protein Com | Translational regulator Com | Translational regulator Com  (PRODOM) |
|  | **P44243** | D12 class N6 adenine-specific DNA methyltransferase | cell division FtsH-related protein | S-adenosyl-L-methionine-dependent methyltransferases | D12 class N6 adenine-specific DNA methyltransferase | D12 class N6 adenine-specific DNA methyltransferase  (BLOCKS) |
|  | **P44246** | FAD-dependent cmnm(5)s(2)U34 oxidoreductase | tRNA modification GTPase TrmE | tRNA 5-methylaminomethyl-2-thiouridine biosynthesis bifunctional protein MnmC | tRNA 5-methylaminomethyl-2-thiouridine biosynthesis bifunctional protein MnmC | Pyridine nucleotide-disulphide oxidoreductase (BLOCKS) |
|  | **Same as 370** | - | - | - | - | - |
|  | **P44247** | FAD-dependent cmnm(5)s(2)U34 oxidoreductase | lic-1 operon protein | Carbonic anhydrase | No result | Oxidoreductase  (PRODOM) |
|  | **P45244** | NAD(P)H nitroreductase | sppA HI1541,protease IV | NAD(P)H nitroreductase YdjA | NAD(P)H nitroreductase YdjA | Nitroreductase family  (BLOCKS) |
|  | **P44251** | dethiobiotin synthetase | biotin synthesis protein | Protein of unknown function (DUF452) | Protein of unknown function (DUF452) | Glycoside hydrolase, family 18  (BLOCKS) |
|  | **P44252** | Outer membrane-specific lipoprotein ABC transporter, permease component LolE | lipoprotein releasing system ATP-binding protein LolD | FtsX-like permease family | Lipoprotein releasing system, transmembrane protein, LolC/E family | Cytochrome c biogenesis protein, transmembrane region  (BLOCKS) |
|  | **P45252** | transcriptional regulator SirB1 | 2-dehydro-3-deoxyphosphooctonate aldolase | Tetratricopeptide repeat (TPR) like | Tetratricopeptide-like helical | Tetratricopeptide repeat (TPR)  (PRODOM) |
|  | **P45253** | N5-glutamine S-adenosyl-L-methionine-dependent methyltransferase | peptide chain release factor 1 | Release factor glutamine methyltransferase | Protein-(glutamine-N5) methyltransferase, release factor-specific | N12 class N6 adenine-specific DNA methyltransferase  (BLOCKS) |
|  | **P44253** | RDD domain-containing protein | 2-dehydro-3-deoxyphosphooctonate aldolase | C-terminal domain of adenylylcyclase associated protein | RDD domian | RDD domain  (BLOCKS) |
|  | **P44254** | pyruvate kinase/TPR repeat, Sel1 subfamily protein | peptide chain release factor 1 | Sel1-like repeats  (TPR (tetratricopeptide repeat) sequences) | No result | DM DNA-binding  (BLOCKS) |
|  | **P44255** | transporting ATPase | penicillin-insensitive murein endopeptidase | NAD(P)-binding Rossmann-fold domains | Protein of unknown function DUF462 | Transporting ATPase YFCM cytoplasmic protein like  (PRODOM) |
|  | **P44256** | DNA polymerase IV | HP HI1563 | Aminoacid dehydrogenase-like, N-terminal domain | DNA polymerase, Y-family, little finger domain | DNA polymerase IV  (PRODOM) |
|  | **P44260** | mu-like prophage protein gp29 | HI1504, I protein | Protein of unknown function (DUF935) | Protein of unknown function DUF935 | GP29 Mu-like  (PRODOM) |
|  | **Q4QKT3** | bacteriophage replication protein A | HP HI1571 | Immunoglobulin-like beta-sandwich | No result | RepA protein (BLOCKS) |
|  | **P44262** | Glyoxalase | peptide chain release factor 1 | Glyoxalase/Bleomycin resistance protein/Dihydroxybiphenyl dioxygenase | Glyoxalase/Bleomycin resistance protein/Dihydroxybiphenyl dioxygenase | Yecm like protein  (PRODOM) |
|  | **Same as 384** | - | - | - | - | - |
|  | **P45267** | adenylate cyclase | Hsf-like protein | CYTH-like adenylate cyclase | CYTH-like phosphatases | Adenylate cyclase  (BLOCKS) |
|  | **P44267** | RNA polymerase sigma factor | RNA polymerase sigma factor | beta and beta-prime subunits of DNA dependent RNA-polymerase | Protein of unknown function DUF2063 | RNA polymerase Rpb1, domain 6  (BLOCKS) |
|  | **P44268** | No result | RNA polymerase sigma factor | Xylose isomerase-like / metal binding proteins | Xylose isomerase-like, TIM barrel domain | 56kDa selenium binding  (BLOCKS) |
|  | **P44269** | outer membrane lipoprotein LolB | putative heme iron utilization protein | FucI/AraA N-terminal and middle domains superfamily | No result | Fusion glycoprotein F0  (BLOCKS) |
|  | **P44270** | TQO small subunit DoxD family protein | RNA polymerase sigma factor | TQO small subunit DoxD | DoxX protein | DoxX protein  (BLOCKS) |
|  | **P44272** | SH3 domain-containing protein | multifunctional tRNA nucleotidyl transferase/2'3'-cyclic phosphodiesterase/2'nucleotidase/phosphatase | Bacterial SH3 domain homologues | SH3-like domain, bacterial | Outer membrane protein (OmpH-like)  (BLOCKS) |
|  | **P44275** | 50S ribosomal protein L25 | cobalt transport protein CbiM | N-terminal domain of cbl (N-cbl) | No result | Transmembrane cytoplasmic CBL protein  (PRODOM) |
|  | **P44277** | protein, Sel1 repeat domain | UDP-N-acetylmuramoyl-L-alanyl-D-glutamate synthetase | Sel1-like repeats  (TPR (tetratricopeptide repeat) sequences) | Sel1-like | Sel1-like repeat  (BLOCKS) |
|  | **P44278** | membrane protein | glycosyl transferase | PEP carboxykinase-like | YwiC-like protein | YwiC-like protein  (PRODOM) |
|  | **P71394** | aminoacrylate peracid reductase | phosphoserine aminotransferase | Endoribonuclease L-PSP | Endoribonuclease L-PSP/chorismate mutase-like | Endoribonuclease L-PSP (BLOCKS) |
|  | **P45279** | Dithiobiotin synthetase | periplasmic serine protease | Proteasome activator | Protein of unknown function DUF1043 | Yhcb like periplasmic protein  (PRODOM) |
|  | **P45280** | alpha-amylase | tRNA pseudouridine synthase A | SNARE associated Golgi protein | SNARE associated Golgi protein | DedA family integral membrane protein  (PRODOM) |
|  | **P44279** | adenylosuccinate synthetase | DNA processing chain A | alpha/beta-Hydrolases | Domain of unknown function DUF2726 | NUDIX hydrolase  (BLOCKS) |
|  | **Q57525** | Aspartokinase | adenylosuccinate synthetase | Lysine-sensitive aspartokinase 3 | Aspartate/glutamate/uridylate kinase | Aspartate kinase  (BLOCKS) |
|  | **P44280** | protein with nucleoside triphosphate hydrolase domain | anti-peptide resistance ABC transporter periplasmic protein | P-loop containing nucleoside triphosphate hydrolases | Protein of unknown function DUF463, YcjX-like protein | ATP-binding ycjx atpase enzyme  (PRODOM) |
|  | **P45290** | phosphoenolpyruvate carboxylase | anti peptide resistance ABC transporter periplasmic protein | Restriction endonuclease-like | Uncharacterised protein family UPF0324, bacteria | Inner membrane protein yeih-related (BLOCKS) |
|  | **P44281** | DEAD/DEAH box helicase/  type I restriction endonuclease subunit R | D-lactate dehydrogenase | Type I restriction and modification enzyme - subunit R C terminal | Type I restriction and modification enzyme, subunit R, C-terminal | DNA-polymerase family A (pol I) signature  (BLOCKS) |
|  | **P44282** | ATPase | No predicted partners | Histidine Phosphotransfer domain  (Two-component signal transduction systems) | Signal transduction histidine kinase, phosphotransfer (Hpt) domain | Ion transport N-terminal  (BLOCKS) |
|  | **No result** | - | - | - | - | - |
|  | **P45298** | 16S rRNA C1402 ribose 2-O-methyltransferase, SAM-dependent | thymidylate kinase | S-adenosylmethionine-dependent methytransferase/ Tetrapyrrole (Corrin/Porphyrin) Methylases | S-adenosylmethionine-dependent methyltransferase/ Tetrapyrrole methylase | Tetrapyrrole family methylase  (PRODOM) |
|  | **P45300** | Endonuclease | HI1655,antigen | Thioredoxin-like | Restriction endonuclease type II-like | Protein of unknown function UPF0102  (BLOCKS) |
|  | **P52606** | DnaA initiator-associating factor for replication initiation | bifunctional heptose 7-phosphate kinase/heptose 1-phosphate adenyltransferase | DnaA initiator-associating protein DiaA like | Sedoheptulose 7-phosphate isomerase / dnaa initiator-associating factor for replication initiation | SIS domain profile(Sugar ISomerase)  (PROSITE PROFILE) |
|  | **P45301** | outer membrane lipoprotein | methyltransferase | bacterial OsmY and nodulation domain  (BON domain) | Transport-associated and nodulation domain, bacteria  (BON domain) | BON domain profile  (PROSITE PROFILE) |
|  | **Q57544** | Zn-dependent hydrolase-like protein, including glyoxylases | D-tyrosyl-tRNA(Tyr) deacylase | Hydroxyacylglutathione hydrolase 2/Metallo-beta-lactamase | Beta-lactamase-like | RNA-metabolising metallo-beta-lactamase  (BLOCKS) |
|  | **P45305** | TatD-related deoxyribonuclease | excinuclease ABC subunit C | TatD related DNase | Deoxyribonuclease, TatD-related | Deoxyribonuclease, TatD-related  (PRODOM) |
|  | **P44283** | putative hedgehog/DD-peptidase-like protein | carboxy-terminal protease | Peptidase_M15_2 | Hedgehog signalling/DD-peptidase zinc-binding domain/ Peptidase_M15_2 | Peptidase M15A  (BLOCKS) |
|  | **P44284** | putative hedgehog/DD-peptidase-like protein | condesin subunit E | Peptidase_M15_2 | Hedgehog signalling/DD-peptidase zinc-binding domain/ Peptidase_M15_2 | Peptidase M15A  (BLOCKS) |
|  | **P44285** | L,D-transpeptidase | carboxy-terminal protease | L,D-transpeptidase catalytic domain | L,D-transpeptidase catalytic domain/ Prokar_lipoprotein | N-6 Adenine-specific DNA methylases signature  (PROSITE PATTERN)/  Prokaryotic membrane lipoprotein lipid attachment site profile  (PROSITE PROFILE) |
|  | **P44287** | paraquat-inducible protein A-like protein | ribonuclease HII | Paraquat-inducible protein A/  Multihaem cytochrome | Paraquat-inducible protein A | Paraquat-inducible protein A  (BLOCKS) |
|  | **P44288** | mce-like protein | carboxy-terminal protease | MCE related protein | Mammalian cell entry (MCE) related protein | Paraquat-inducible protein B  (PRODOM) |
|  | **P44289** | YccS/YhfK family integral membrane protein | methylglyoxal synthase | ARM repeat | Integral membrane protein, YccS/YhfK | Integral membrane protein, YccS/YhfK  (PRODOM) |
|  | **P44290** | No result | methylglyoxal synthase | Uncharacterized protein conserved in bacteria (DUF2057) | Uncharacterized protein conserved in bacteria (DUF2057) | Conserved hypothetical protein 156  (BLOCKS) |
|  | **P44292** | molybdate ABC transporter permease | hemoglobin-binding protein | Inosine monophosphate dehydrogenase (IMPDH) | Rossmann-like alpha/beta/alpha sandwich fold | Protein ydcF like transmembrane  (PRODOM) |
|  | **P45332** | lipopolysaccharide ABC transporter permease | ABC transporter ATP-binding protein | Predicted permease YjgP/YjgQ family | Permease YjgP/YjgQ, predicted | Predicted permease YjgP/YjgQ family  (BLOCKS) |
|  | **P45333** | lipopolysaccharide ABC transporter permease LptF | ABC transporter ATP-binding protein | Predicted permease YjgP/YjgQ family | Permease YjgP/YjgQ, predicted | Predicted permease YjgP/YjgQ family |
|  | **P44293** | protein YgiW | transcriptional regulatory protein | Cadherin-like /  Bacterial OB-(BOF) fold (oligonucleotide/oligosaccharide binding motif) | Viral OB-fold, YgiW | Shiga-like toxin, beta subunit  (BLOCKS) |
|  | **P44294** | mannose-6-phosphate isomerase | glucose-specific PTS system component | RmlC-like cupins | Mannose-6-phosphate isomerase /  Cupin, RmlC-type | FAD-dependent pyridine nucleotide reductase signature  (PRINTS) |
|  | **P44296** | trimeric autotransporter adhesin | PII uridylyl-transferase | RmlC-like cupins | Adhesin YadA, collagen-binding domain | Cleaved adhesion  (BLOCKS) |
|  | **Q57066** | Transposase | PII uridylyl-transferase | Tetracyclin repressor-like, C-terminal domain | Homeodomain-like | Putative transposase, YhgA-like  (BLOCKS) |
|  | **P44297** | No result | iron-sulfur cluster insertion protein ErpA | Cystatin/monellin | YacL Protein like | YacL Protein like  (PRODOM) |
|  | **O05087** | Mn2+ and Fe2+ transporter of the NRAMP family | LamB/YcsF family protein | Natural resistance-associated macrophage protein | Natural resistance-associated macrophage protein like | Mn2+ and Fe2+ transporter of the NRAMP family  (PRODOM) |
|  | **P44298** | allophanate hydrolase subunit 2 | LamB/YcsF family protein | Allophanate hydrolase subunit 2 | Allophanate hydrolase subunit 2 | Urea amidolyase-related  (BLOCKS) |
|  | **P44299** | allophanate hydrolase subunit 1 | LamB/YcsF family protein | Allophanate hydrolase subunit 1 | Allophanate hydrolase subunit 1 | Allophanate hydrolase subunit 1  (PRODOM) |
|  | **P44300** | L-lactate dehydrogenase | putative branched-chain amino acid permease | Bacterial inner membrane protein  (Imp-YgjV) | Uncharacterised protein family HI1736/YgjV | ATPase proteolipid  (BLOCKS) |
