# Supplementary material for: Functional Annotation of Conserved Hypothetical Proteins from Haemophilus influenzae Rd KW20
Source: PLoS One. 2013 Dec 31;8(12):e84263. doi: 10.1371/journal.pone.0084263 (PMC3877243; doi:10.1371/journal.pone.0084263)
Supplement: Table S4 — List of functionally annotated domains of 429 HPs from H. influenzae by CATH, SUPERFAMILY, PANTHER, Pfam, SYSTERS, CDART SVMProt and ProtoNet. (DOCX) [file pone.0084263.s004.docx]

| **S.NO**  Table S2 List of functionally annotated domain of 429 HPs from *H. influenzae* by CATH, SUPERFAMILY, PANTHER, Pfam, SYSTERS, CDART SVMProt and ProtoNet | **UNIPROT ID** | **CATH** | **SUPERFAMILY** | **PANTHER families**  **(subfamily/family /superfamily )** | **Pfam**  **(family/Domain)** | **SYSTERS**  **(cluster)** | **CDART** | **SVMProt (family)** | **ProtoNet**  **Cluster**  **(cluster name)** |
| --- | --- | --- | --- | --- | --- | --- | --- | --- | --- |
| 1 | **Q57048** | No result | No result | 2-oxoglutarate/malate translocator subfamily | Sodium: sulfate symporter transmembrane family | Cluster-146746  (2-oxoglutarate/malate translocator) | Arsenic efflux pump | TC 2.A. Electrochemical Potential-driven transporters - Porters (uniporters, symporters, antiporters) | Cluster 3631830  Sodium/sulfate symporter |
| 2 | **P44465** | UPF0250 protein MCA0107 -like domain family | YbeD-like family | No result | DUF493 family | Cluster - 156264  (hypothetical protein) | DUF493 superfamily | All lipid binding proteins | Cluster 3959135  Protein of unknown function DUF493 |
| 3 | **P44471** | ATPase synthesis protein 25, mitochondrial -like domain family | Iojap/YbeB-like family  (Nucleotidyl transferase) | Iojap superfamily ortholog family | Oligomerization family | Cluster -153016  (Iojap related protein) | Oligomerization superfamily | Iron binding | Cluster 4170010  Iojap related protein |
| 4 | **P44472** | Putative transport protein YidE -like domain ½ family | TrkA C-terminal domain-like family | Uncharacterized family | TrkA_c family  (Regulator of K+ conductance, C-terminal) | Cluster-151662  (Aspartate: alanine antiporter) | TrkA_c superfamily | Transmembrane | YidE/YbjL duplication  (cation transport) |
| 5 | **P43929** | Regulator of ribonuclease activity B -like domain family | Hypothetical protein VC0424 family | No result | RraB family  (Regulator of ribonuclease activity B  Domain) | Cluster-155970  (DNA for Glycosyltransferases, lytic transglycosylase, dTDP-4-rhamnose reductase) | DUF695 superfamily | mRNA slicing | Cluster 3251533  Conserved hypothetical protein CHP01619  (catalytic activity) |
| 6 | **P43931** | No result | Calcium ATPase, transmembrane domain M family | No result | DUF697 family | Cluster – 149616  (GTP-binding protein) | DUF697 superfamily | EC [4.1.-.-](http://jing.cz3.nus.edu.sg/cgi-bin/desfam.pl?FN=4.1): Lyases - Carbon-Carbon Lyases | Cluster 418116  [Conserved hypothetical protein CHP01620](http://www.ebi.ac.uk/interpro/IEntry?ac=IPR006507) |
| 7 | **P44477** | No result | No result | Bax inhibitor-related family | Bax1-I family | cluster -141641  (Putative carrier/transport protein) | B-I-1 like superfamily | Transmembrane | Cluster 3845992  Uncharacterized protein family UPF0005 |
| 8 | **P44478** | No result | No result | No result | YtfJ_HI0045 family | cluster 144963  (Protein ytfJ precursor  ) | YTFJ-HI0045  superfamily | No result | Cluster 3999593  Conserved hypothetical protein, YtfJ |
| 9 | **P44484** | No result | No result | Family not named | DctQ family | Cluster -140419  (Serpentine receptor class alpha 3) | Dct-Q superfamily | Transmembrane | Tripartite ATP-independent periplasmic transporter, DctQ component |
| 10 | **P71336** | No result | Phosphate binding protein-like family | No result | SBP_bac_7 family  (Bacterial extracellular solute-binding protein) | Cluster-147378  (Extracellular solute-binding protein, family 7) | SBP_bac_7 superfamily | EC 2.4.-.-: Transferases - Glycosyltransferases | Cluster 4180508  Extracellular solute-binding protein, family 7, bacteria |
| 11 | **P43932** | No result | Calcium ATPase, transmembrane domain M family | Inner membrane protein  family | Integral membrane protein TerC family | Cluster-144352  (TerC family protein ) | TerC superfamily | Transmembrane | Cluster 4019065  Integral membrane protein TerC |
| 12 | **P44492** | Replication-associated protein -like domain family | YjeE-like family  (P-loop containing nucleoside triphosphate hydrolases superfamily) | No result | Uncharacterized P-loop hydrolase UPF0079 family | Cluster- 149840  (ATPase OR kinase) | AAA superfamily | Iron-binding | Cluster 4166012  Uncharacterized protein family UPF0079, ATPase bacteria |
| 13 | **P43935** | No result | MiaE-like family  (Ferritin-like superfamily ) | Ferredoxin-related family | Protein of unknown function (DUF455) family | Cluster-148957  (3-oxoacyl-[acyl-carrier-protein] synthase-like protein) | Ferritin like superfamily | EC 2.7.-.-: Transferases - Transferring Phosphorus-Containing Groups | Cluster 3942395  Protein of unknown function DUF455 |
| 14 | **P43936** | mRNA interferase MazF -like domain family | Kid/PemK family  (Cell growth inhibitor/plasmid maintenance toxic component superfamily) | No result | PemK-like protein family | Cluster- 133513  (Hypothetical protein yifN) | PemK superfamily  (toxin of the ChpB-ChpS toxin-antitoxin system) | Zinc binding | Cluster 3597088  Plasmid maintenance toxin/Cell growth inhibitor |
| 15 | **P44500** | Deoxyribonuclease TatD -like domain family | TatD Mg-dependent DNase-like family | TATD family deoxyribonuclease | TatD_DNase family | Cluster- 149137  (Hydrolase, TatD family) | Metallo_dependent_hydrolases superfamily | Zinc-binding | Cluster 4150200  Deoxyribonuclease, TatD Mg-dependent |
| 16 | **P43937** | Acyl-CoA dehydrogenase -like domain family | Medium chain acyl-CoA dehydrogenase, NM (N-terminal and middle) domains family | No result | Acyl-CoA_dh_N family  (Acyl-COA dehydrogenase ,N-terminal domain) | Cluster- 109390  (Putative acyl-CoA dehydrogenase) | ACDA superfamily | Metal-binding | Cluster 4673817  Eukaryota |
| 17 | **P43938** | No result | No result | No result | No result | Cluster -18480  (hypothetical protein) | No result | All DNA binding | Cluster 651650  Haemophilus influenzae |
| 18 | **P44506** | Alanine racemase -like domain family | "Hypothetical" protein ybl036c family  (PLP-binding barrel superfamily) | Proline synthetase co-transcribed bacterial homolog protein family | Ala_racemase_N family  (Alanine racemase, N-terminal domain) | Cluster-140661  (Putative Proline synthetase associated protein) | PLPDE_III superfamily | Metal binding | Cluster 4106519  Predicted pyridoxal phosphate-dependent enzyme, YBL036C type |
| 19 | **P44507** | Glycerate kinase -like domain family | Glycerate kinase I family | Glycerate kinase family | Gly_kinase family | Cluster-155132  (glycerate kinase) | Gly_kinase superfamily | EC 2.7.-.-: Transferases - Transferring Phosphorus-Containing Groups | Cluster 4144170  Glycerate kinase, domain 2 |
| 20 | **Q57493** | No result | No result | gnt family gluconate transporter family | GntP permease family | Cluster- 145230  (gluconate permease) | PRK10034 superfamily | TC 2.A. Electrochemical Potential-driven transporters - Porters | Cluster 3750408  Divalent ion symporter |
| 21 | **P44509** | No result | No result | No result | Diacid_rec family  (Putative sugar diacid recognition ) | Cluster – 147588  (carbohydrate diacid regulator) | Diacid_rec superfamily | All DNA binding | Cluster 4157002  Putative sugar diacid recognition |
| 22 | **P43939** | No result | No result | NAD dependent epimerase/dehydratase  Family | GntP permease family | Cluster- 142884  (nucleoside diphosphate sugar epimerase) | NADB_Rossman superfamily | EC 2.4 Transferases - glycosyltransferases | Cluster 4107863  NAD dependent epimerase/dehydratase |
| 23 | **Q57060** | Ribosomal protein L11 methyltransferase -like domain family | UbiE/COQ5-like family  (S-adenosyl-L-methionine-dependent methyltransferases superfamily ) | Methyltransferase family | Methyltransf_11 family | Cluster – 141931  (SAM dependent methyltransferase) | AdoMet_MTases superfamily | Zinc binding | Cluster 3952797  Methyltransferase type 11 |
| 24 | **P43940** | No result | No result | No result | No result | Cluster – 113245  (lipoprotein) | No result | All lipid binding proteins | Cluster 3845090  Haemophilus influenzae |
| 25 | **P44515** | Regulatory protein spx -like domain family | ArsC-like family  (Thioredoxin-like suprerfamily) | No result | Arsc family | Cluster – 148069  (Arsenate reductase) | GST_C_family  Superfamily | All lipid binding protein | Cluster 4139373  Conserved hypothetical protein ,ArsC related |
| 26 | **Q57354** | UPF0135 protein ybgI -like domain 2 family | NIF3 (NGG1p interacting factor 3)-like family | NGG1 interacting factor 3 superfamily | NIF3 family  (NGG1p interacting factor 3 ) | Cluster – 144114  (NGG1- interacting factor 3) | NIF3 superfamily | Ec 4.1 layases-carbon-carbon lyases | Cluster 3959512  NIF3 (NGG1p interacting factor 3 like |
| 27 | **P44520** | No result | No result | No result | DUF1212 family | Cluster – 145492  (Putative integral membrane protein ) | Nucleotidyl_cyc_III superfamily | Transmembrane | Protein of unknown function DUF 1212 |
| 28 | **P43943** | Lipopolysaccharide biosynthesis protein -like domain family | FepE-like family  (Bacterial polysaccharide co-polymerase-like superfamily ) | No result | GTP_EFTU family  (Elongation factor Tu GTP binding domain ) | Cluster- 104440  (transcription elongation factor ) | No result | Ec-2.7 Transferases Phosphorus containing group | Cluster 3393659  Haemophilus |
| 29 | **P71339** | Myb-related protein -like domain ½ family | Homeodomain-like/TrpR-like superfamily | No result | HTH_28 family  (Helix-turn-helix domain) | Cluster – 142986  (putative transposase) | HTH_23 superfamily | RNA binding protein | Cluster 360664  Acid phosphatase |
| 30 | **Q57097** | SUMO-activating enzyme subunit 1 -like domain ½ family | Molybdenum cofactor biosynthesis protein MoeB family | Ubiquitin activating enzyme E1 family | ThiF family | Cluster – 141827  (Molybdenum biosynthesis protein) | E1_enzyme_family superfamily | Transmembrane | Cluster 4090928  UBA/THIF-type NAD/FAD binding fold |
| 31 | **P43947** | No result | No result | No result | DUF2301 family | Cluster – 112930  (putative integral membrane protein) | DUF2301 superfamily ,  zf-ribbon_3 superfamily | Transmembrane | Cluster 3939039  Gammaproteo bacteria |
| 32 | **P44530** | No result | No result | Xanthin/uracil/vit C permease family member | Permease family | Cluster – 147664  (xanthin/uracil permease) | No result | Transmembrane | Cluster 4145036  Xanthin/uracil/vit C permease |
| 33 | **P43952** | No result | No result | No result | AsmA_2 Family  (Asm-A like C-terminal region ) | Cluster – 140134  (Penicillin binding protein ) | No result | Transmembrane | Cluster 3742698  pasteurellaceae |
| 34 | **P44540** | Bifunctional protein glk -like domain family/ Glucose-6-phosphate isomerase -like domain ½ family | mono-SIS domain family /Homeodomain-like superfamily | Glucokinase family | HTH_6 family  (Helix turn helix ,rpiR family ) | Cluster – 144388  (transcriptional regulator , RpiR family ) | SIS superfamily | Transferases – transferring alkyl or aryl group , other than methy group | Cluster 4192755  Helix turn helix protein RpiR |
| 35 | **P44542** | No result | Phosphate binding protein-like family | No result | SBP_bac_7 family  (Bacteria extracellular solute binding protein ) | Cluster – 147377  (C-4 dicarboxylate binding protein ) | SBP_bac_7 superfamily | All lipid binding protein | Cluster 4180508  Extracellular solute binding protein , family 7 , bacteria |
| 36 | **P44543** | No result | No result | Family not named | DctQ family  (Tripartite ATP-independent periplasmic transporter) | Cluster – 147373  (permease component of C-4 dicarboxylate transporter ) | DctQ superfamily | Transmembrane | Cluster 4101809  TRAP dicarboxylate transporter , DctM subunit |
| 37 | **O86220** | No result | No result | No result | Got1/Sft2-like family | Cluster -18576  (hypothetical protein) | No result | Transmembrane | No result |
| 38 | **P43953** | No result | No result | Protein-S Isoprenylcysteine O-methyltransferase family | PEMT family  (Phospholipid methyl transferase ) | Cluster – 146297  (putative protein-S-isoprenyl cysteine methyltransferase ) | PEMT superfamily | Photosynthesis | Cluster 4548116  C-terminal protein amino acid methylation |
| 39 | **P44545** | No result | Band 7/SPFH domain family | Band 7 protein related family | SPFH domain , Band 7 family | Cluster – 143375  (HFLC protein ) | Band_7 superfamily | EC-3.4 hydrolases – acting on peptide bonds | Cluster 4131544  HFLC |
| 40 | **P43954** | Holo-[acyl-carrier-protein] synthase -like domain family | 4'-Phosphopantetheinyl transferase SFP family | Phosphopantetheinyl transferase family | ACPS family  (4'-phosphopantetheinyl transferase superfamily) | Cluster – 115344  (putative 4’-phosphopantetheinyl transferase) | ACPS superfamily | Metal binding family | Cluster 4098210  4’-phosphopantetheinyl transferase |
| 41 | **P43790** | No result | No result | No result | DUF 177 family | Cluster – 156287  (predicted metal binding ) | DUF 177 superfamily | No match | Cluster 4143789  Protein of unknown function DUF177 |
| 42 | **P43960** | No result | No result | No result | DUF539 family | Cluster – 133282  (putative periplasmic protein ) | DUF539 superfamily | Transmembrane | Cluster 4129373  Protein of unknown function DUF539 |
| 43 | **P44552** | Putative uncharacterized protein -like domain family | CNF1/YfiH-like putative cysteine hydrolases superfamily | Uncharacterized family YFIH | Cu-oxidase_4 family  (multi-copper polyphenol oxidoreductase laccase ) | Cluster – 154635  (putative inner membrane protein ) | Cu_oxidase_4 superfamily | All lipid binding protein | Cluster 4170210  multi-copper polyphenol oxidoreductase laccase |
| 44 | **P44553** | Mitochondria fission 1 protein -like domain family | Tetratricopeptide repeat (TPR) family | No result | YfiO family  (Outermembrane protein lipoprotein domain) | Cluster – 154926  (putative competence protein ComL) | No result | All lipid binding protein | Cluster 4133061  Outer membrane assembly lipoprotein YfiO |
| 45 | **P43961** | No result | NE1680-likefamily | No result | LPAM_1 family  (Prokaryotic membrane lipoprotein lipid attachment site motif) | Cluster – 121456  (P36 like protein homolog) | No result | All lipid binding protein | Cluster 3750388  Periplasmic protein HI0178 |
| 46 | **P46490** | No result | No result | Transmembrane protein YfcA family | Sulfite exporter TauE/SafE family | Cluster – 150623  (transporter protein ) | PRK10621 super family | Transmembrane | Cluster 4629881  Protein of unknown function DUF81 |
| 47 | **P43963** | No result | No result | No result | RecX family | Cluster – 65234  (predicted transcriptional regulator , Fis family ) | No result | Transferases – transferring phosphorus containing group | Cluster 638968  Haemophilus influenzae |
| 48 | **P43965** | Transposase for insertion sequence element IS200 -like domain family | Transposase IS200-like family | No result | Transposases IS200 like family | Cluster – 156161  (YAFM protein) | Y1_Tnp superfamily | All lipid binding protein | Cluster 4081513  Proteobacteria |
| 49 | **P44577** | No result | No result | No result | DUF417 family | Cluster – 156419  (putative membrane protein ) | DUF417 superfamily | Transmembrane | Cluster 4017619  Protein of unknown function DUF417 |
| 50 | **O86222** | racil-DNA glycosylase -like domainfamily | Mug-like family  (Uracil-DNA glycosylase-likesuperfamily) | No result | UDG family  (Uracil DNA glycosylasesuperfamily ) | Cluster – 149074  (Uracil DNA glycosylase) | UDG like superfamily | Transmembrane | Cluster 4097728  Uracil DNA glycosylase |
| 51 | **P44579** | No result | Multidrug resistance efflux transporter EmrE family | Putative chloramphenicol resistance transporter family | Multidrug resistance efflux transporter family (EmrE family ) | Cluster-  145543  (RarD protein) | EmrE superfamily | Transmembrane | Cluster 4593188  RarD protein |
| 52 | **P44583** | uncharacterised family 185 -like domain | YhcH-like family (Clavaminate synthase-like superfamily) | No result | Domain of unknown function (DUF386) family | Cluster- 156379  (Evolved beta-galactosidase beta-subunit) | DUF386 superfamily | EC 2.4.-.-: Transferases - Glycosyltransferases | Cluster 3982124Conserved hypothetical protein CHP00022 |
| 53 | **P43966** | General stress protein A -like domain family | Glycogenin family/Galactosyltransferase LgtC family  (Nucleotide-diphospho-sugar transferases superfamily ) | No result | No result | Cluster-48523  (hypothetical membrane lipoprotein) | Glyco_tranf_GTA_type Superfamily | EC 1.9.-.-: Oxidoreductases - Acting on a heme group of donors | Cluster 3612942  Biotin--[acetyl-CoA-carboxylase] ligase |
| 54 | **P43968** | No result | No result | No result | No result | Cluster- 120053  (hypothetical protein ) | No result | No result | Cluster 801874  Haemophilus influenzae |
| 55 | **P44588** | No result | No result | No result | DUF331 family | Cluster- 133502  (putative regulator) | DUF 3331 superfamily | Zinc binding | Cluster 4325097  Protein of unknown function DUF331 |
| 56 | **P44593** | Sulfurtransferase TusA -like domain family | SirA-like family | No result | Sulfurtransferase TusA family | Cluster- 145561  (Hypothetical protein yeeD) | SirA_YedF_YeeD Superfamily | Metal-binding | Cluster 4048585  SirA-like |
| 57 | **P43971** | Iron-sulfur cluster repair protein YtfE -like domain family | Formin homology 2 domain (FH2 domain) family | No result | Hemerythrin family  (Hemerythrin HHE cation binding domain) | Cluster-125947  (Methyl-accepting chemotaxis protein mcpB) | Hemerythrin-like Superfamily | All DNA-binding | Cluster 4146242  Haemerythrin/HHE cation-binding motif |
| 58 | **P43972** | No result | No result | No result | LAM_1 family  (Prokaryotic membrane lipoprotein lipid attachment site motif) | Cluster- 120058  (Protein HI0246 precursor) | No result | No result | Cluster 3854887  Uncharacterised conserved protein UCP020772 |
| 59 | **P71346** | Ribosomal subunit interface protein -like domain family | Ribosome binding protein Y (YfiA homologue) family | No result | Ribosomal_S30AE family | Cluster- 150719  (Sigma-54 modulation protein) | RaiA superfamily | Sodium-binding | Cluster 3863398  Keyword 9863 |
| 60 | **P44606** | 1-(5-phosphoribosyl)-5-[(5-phosphoribosylamino)methylideneamino] -like domain family | FMN-linked oxidoreductases family | tRNA-Dihydrouridine synthase family | Dus family | Cluster- 147656  (TIM barrel proteins ) | TIM_phosphate_binding Superfamily | EC 3.5.-.-: Hydrolases - Acting on Carbon-Nitrogen Bonds, other than Peptide Bonds | Cluster 4154178  TRNA-dihydrouridine synthase |
| 61 | **P43975** | Phosphoglycerol transferase I -like domain family | Arylsulfatase family | No result | Sulfatase family | Cluster- 151986  (putative integral membrane protein ) | Sulfatase Superfamily | Transmembrane | Cluster 4276704  Sulfatase |
| 62 | **P44609** | UPF0225 protein MCA2786 -like domain family | SEC-C associated NTF2-like domain family/Sec-C motif family | No result | SEC-C family | Cluster- 147600  (Hypothetical protein HI0277) | SEC-C superfamily | Zinc-binding | Cluster 4127137  SEC-C motif |
| 63 | **P43980** | UPF0301 protein MCA2336 2 -like domain family | VC0467-like family | Uncharacterized protein YQGE family | DUF179 family | Cluster- 151233  (transcriptional regulator ) | DUF179 superfamily | TC 2.A.1 Major facilitator family (MFS) | Cluster 4164891  Protein of unknown function DUF179 |
| 64 | **P43982** | No result | No result | No result | MarB protein family | Cluster- 97759  (Merozoite surface protein-3) | No result | Transmembrane | Cluster 4596145  Proteobacteria |
| 65 | **P44634** | UPF0082 protein At2g25830 -like domain family | YebC-like family | Uncharacterized family | Transcrip_reg family | Cluster- 152835  (Glucose-1-phosphate adenylyltransferase) | Transcrip_reg Superfamily | All lipid-binding proteins | Cluster 4120159  Protein of unknown function DUF28 |
| 66 | **P43984** | No result | No result | No result | Isoprenylcysteine carboxyl methyltransferase  (ICMT) family | Cluster- 146292  (Farnesyl cysteine carboxyl-methyltransferase) | PEMT superfamily | Transmembrane | Cluster 3831400  Protein-S-isoprenylcysteine O-methyltransferase activity |
| 67 | **P44640** | No result | No result | No result | Na_H_antiport_2 family | Cluster- 149200  (Putative integral membrane protein) | Na_H_antiport_2 Superfamily | TC 2.A. Electrochemical Potential-driven transporters - Porters (uniporters, symporters, antiporters) | Cluster 3390635  Na+/H+ antiporter NhaC-like |
| 68 | **P43987** | No result | MW0975(SA0943)-like family | No result | priB_priC family | Cluster- 156082  (Primosomal replication protein N) | priB_priC superfamily | No result | Cluster 4045251  Pasteurellales |
| 69 | **P44641** | L-lysine 2,3-aminomutase -like domain family | MoCo biosynthesis proteins family | lysine 2,3-aminomutase like family | Fer4_14 family | Cluster- 133235  (L-lysine 2,3-aminomutase) | Radical_SAM Superfamily | Iron-binding | Cluster 4031921  Protein of unknown function DUF160 |
| 70 | **P44646** | No result | No result | Transmembrane protein family | Lig_chan family  (Ligand-gated ion channel) | Cluster- 140871  (Putative transport protein) | yhhT Superfamily  (Predicted permease) | Transmembrane | Cluster 4138321  Uncharacterised protein family UPF0118 |
| 71 | **P44649** | No result | No result | No result | Dehydratase_hem family | Cluster- 128738  (splicing factor like protein ) | DUF469 Superfamily ,Sulfotransfer_3 Superfamily | Zinc-binding | Cluster 4074320  Protein of unknown function DUF469 |
| 72 | **P24324** | uncharacterised family 154 -like domain ½ family | Alpha-2,3/8-sialyltransferase CstII family | No result | CST-I family | Cluster- 142738  (Alpha-2,3-sialyltransferase) | CST-I Superfamily | Lipoprotein | Cluster 4094607  Alpha-2,3/8-sialyltransferase CstII |
| 73 | **Q57065** | No result | SinR domain-like family  (lambda repressor-like DNA-binding domains superfamily) | No result | HTH_25 family  (Helix-turn-helix domain) | Cluster- 150265  (Putative DNA-binding protein) | HTH_XRE Superfamily | All lipid-binding proteins | Cluster 4174733  Helix-turn-helix type 3 |
| 74 | **P43989** | Receptor-associated protein of the synapse -like domain ½ family | Tetratricopeptide repeat (TPR) family | No result | TPR_21 family | Cluster- 144927  (Colicin protein) | TPR Superfamily | All lipid-binding proteins | Cluster 4156687  Tetratricopeptide-like helical |
| 75 | **P44668** | uncharacterised family 163 -like domain | IscX-like family | No result | Fe-S_assembly family | Cluster- 156071  (FeS cluster assembly) | Fe-S_assembly superfamily | Zinc-binding | Cluster 4639845  Hypothetical protein yfhj |
| 76 | **P44670** | No result | SMI1/KNR4-like family | No result | DUF2625 family | Cluster-133422  (Hypothetical protein) | DUF2625 Superfamily | All lipid-binding proteins | Cluster 3836505  Bacteria |
| 77 | **P44672** | Iron-sulfur cluster insertion protein erpA -like domain family | HesB-like domain family | FeS cluster assembly protein family | Fe-S_biosyn family | Cluster- 145453  (Iron-sulfur cluster assembly protein) | Fe-S_biosyn superfamily | Iron-binding | Cluster 4138459  FeS cluster insertion |
| 78 | **P44675** | HTH-type transcriptional regulator NsrR -like domain family | Z-DNA binding domain family | No result | Rrf2 family | Cluster- 139965  (transcriptional regulator) | Rrf2 Superfamily | Zinc-binding | Cluster 4164851  Transcriptional regulator, Rrf2 |
| 79 | **P44676** | RNA (cytidine/uridine-2'-O-)-methyltransferase -like domain family | SpoU-like RNA 2'-O ribose methyltransferase family | RNA methyltransferase family | SpoU rRNA Methylase family | Cluster- 149834  (tRNA/rRNA methyltransferase) | SpoU_methylase Superfamily | EC 2.1.-.-: Transferases - Transferring One-Carbon Groups | Cluster 4156967  RNA methyltransferase TrmH, group 1 |
| 80 | **P44679** | 1,4-dihydroxy-2-naphthoyl-CoA hydrolase -like domain family | 4HBT-like family  (Thioesterase/thiol ester dehydrase-isomerase superfamily) | Family not named | Thioesterase superfamily | Cluster- 149566  (4-hydroxybenzoyl-CoA thioesterase) | hot_dog Superfamily | Zinc-binding | Cluster 4145117  4-hydroxybenzoyl-CoA thioesterase |
| 81 | **P43990** | Acetate kinase -like domain ½ family | YeaZ-like family  (Actin-like ATPase domainsuperfamily) | o-sialoglycoprotein endopeptidase family | Glycoprotease family | Cluster- 143405  (Glycoprotein endopeptidase) | COG1214 Superfamily | EC 3.4.-.-: Hydrolases - Acting on peptide bonds (Peptidases) | Cluster 4162826  Peptidase M22, glycoprotease |
| 82 | **P43992** | Rhamnogalacturonan acetylesterase -like domain family | Rhamnogalacturonan acetylesterase family  (SGNH hydrolase superfamily) | No result | No result | Cluster- 93419  (Putative transcriptional regulatory protein) | No result | All lipid-binding proteins | Cluster 4167654  Acyltransferase 3 |
| 83 | **P43994** | uncharacterised family 256 -like domain | MoaD/ThiS superfamily | No result | RnfH family Ubiquitin | Cluster- 155495  (Protein rnfH) | Ub-RnfH Superfamily | Sodium-binding | Cluster 4150562  (Uncharacterised protein family UPF0125) |
| 84 | **P44683** | Lysine-specific demethylase NO66 -like domain ½ family/  Dachshund -like domain family | Asparaginyl hydroxylase-like family  (Clavaminate synthase-like superfamily)/  Dachshund-homology domain family (Putative DNA-binding domainsuperfamily) | mina53 (myc induced nuclear antigen) family | Cupin superfamily protein | Cluster- 150111  (Mina53) | Cupin_2 Superfamily | EC 3.1.-.-: Hydrolases - Acting on Ester Bonds | Cluster 3887799  Cupin 4 |
| 85 | **P44684** | RNA pyrophosphohydrolase -like domain family | MutT-like family (Nudix superfamily) | udp/adp-sugar pyrophosphatase family | NUDIX family | Cluster 147294  (Pyrophosphohydrolase) | Nudix_Hydrolase Superfamily | EC 3.6.-.-: Hydrolases - Acting on Acid Anhydrides | Cluster 4127686  Nucleoside diphosphate pyrophosphatase |
| 86 | **P44686** | UPF0234 protein Rv0566c/MT0592 -like domain family | No result | No result | DUF406 family | Cluster- 94128  (Similar to Ikaros/Helios (Fragment)) | DUF406 superfamily | EC 2.4.-.-: Transferases - Glycosyltransferases | Cluster 4482597  Conserved hypothetical protein CHP00743 |
| 87 | **P44691** | Vitamin B12 import system permease protein BtuC -like domain family | ABC transporter involved in vitamin B12 uptake, BtuC family | ABC-transporter metal-binding protein family | ABC 3 transport family | Cluster- 149307  (ABC transporter) | TM_ABC_iron-siderophores_like Superfamily | Transmembrane | Cluster 4098632  ABC 3 |
| 88 | **P44693** | Lipoprotein -like domain family | Peptidoglycan hydrolase LytM family (Duplicated hybrid motif superfamily) | Peptidase-related family | Peptidase family M23 | Cluster- 138457  (Peptidase, M23/M37 family) | Peptidase_M23 Superfamily | EC 3.4.-.-: Hydrolases - Acting on peptide bonds (Peptidases) | Cluster 4184571  Peptidase M23B |
| 89 | **Q57392** | Outer membrane protein -like domain family | Outer membrane protein family | No result | Opacity family | Cluster- 150900  Opacity protein | OMP_b-brl Superfamily | Photosynthesis | Cluster 3846753  Opacity |
| 90 | **P43995** | Sodium/potassium-transporting ATPase subunit gamma -like domain family | Ribbon-helix-helix superfamily | No result | DUF1778 family | Cluster- 152250  (Putative helix-turn-helix protein) | DUF1778 superfamily | EC 4.1.-.-: Lyases - Carbon-Carbon Lyases | Cluster 4095373  Protein of unknown function DUF1778 |
| 91 | **P44702** | tRNA (adenine-N(6)-)-methyltransferase -like domain family | N5-glutamine methyltransferase, HemKfamily | Methyltransferase family | Methyltransf_26 family | Cluster- 145157  (O-methyltransferase) | AdoMet_MTases Superfamily | EC 3.1.-.-: Hydrolases - Acting on Ester Bonds | Cluster 4119785  Methyltransferase small |
| 92 | **P44709** | uncharacterised family 27 -like domain family | No result | No result | DUF416 family | Cluster- 133516  (Hypothetical protein ) | DUF416 superfamily | All DNA-binding | Cluster 3848570  Protein of unknown function DUF416 |
| 93 | **P31777** | Ribosomal RNA small subunit methyltransferase G -like domain family | S-adenosyl-L-methionine-dependent methyltransferases superfamily | No result | Ribosomal RNA small subunit methyltransferase D, RsmJ family | Cluster- 152634  (Florfenicol resistance protein) | No result | DNA repair | Cluster 3854691  Protein of unknown function DUF519 |
| 94 | **P44711** | UPF0133 protein Rv3716c/MT3819 -like domain family | YbaB-like family | No result | YbaB/EbfC DNA-binding family | Cluster- 152197  (YbaB protein) | YbaB_DNA_bd Superfamily | All lipid-binding proteins | Cluster 4110991  Keyword 10025 |
| 95 | **P43997** | No result | No result | No result | LPAM_1 family | Cluster- 120106  (Hypothetical protein) | No result | All lipid-binding proteins | Cluster 3988855  Pasteurellales |
| 96 | **P43998** | No result | No result | No result | No result | Cluster- 145440  (fucosyltransferase) | No result | Lipoyl | Cluster 3948648  Proteobacteria |
| 97 | **P44717** | KpsF protein -like domain ½ family/  UDP-N-acetylenolpyruvoylglucosamine reductase -like domain family | CorC/HlyC domain-like family (FAD-binding/transporter-associated domain-like superfamily)/  CBS-domain pair family | Hemolysin-related family | CBS family  (Cystathionine beta synthase) | Cluster- 143846  (Hemolysin protein) | CBS_pair Superfamily | Transmembrane | Cluster 4144744  Transporter-associated region |
| 98 | **P43999** | No result | No result | No result | DUF1523 family | Cluster- 140145  (ABC transporter subunit) | DUF1523 superfamily | Transmembrane | Cluster 3869536  Protein of unknown function DUF1523 |
| 99 | **P44718** | Deoxyribonuclease TatD -like domain family | TatD Mg-dependent DNase-like family  (Metallo-dependent hydrolases superfamily) | Tatd family deoxyribonuclease | TatD_DNase family | Cluster- 149137  (Hydrolase, TatD family) | metallo-dependent_hydrolases Superfamily | No result | Cluster 4150200  Deoxyribonuclease, TatD Mg-dependent |
| 100 | **P44720** | Zinc finger protein 425 -like domain family | No result | Uncharacterized family | YceG-like family | Cluster- 152447  (Aminodeoxychorismate lyase (PABC)) | YceG-like family superfamily | Zinc-binding | Cluster 4125861  Aminodeoxychorismate lyase |
| 101 | **Q57144** | No result | No result | No result | UreF family | Cluster-155241  (VirK virulence protein) | DUF535 Superfamily/ANK Superfamily | Iron-binding | Cluster 4047417  Protein of unknown function DUF535 |
| 102 | **P44000** | RNA-modifying protein ygfZ -like domain family | Aminomethyltransferase folate-binding domain family | Uncharacterized family | GCV_T family | Cluster- 144469  (aminomethyltransferase) | ygfZ_signature Superfamily/GCV_T_C Superfamily | DNA repair | Cluster 4142011  Folate-binding, YgfZ |
| 103 | **P44726** | No result | No result | Uncharacterized family | YicC-like family | Cluster- 148892  (stress-induced protein) | YicC_N Superfamily | Structural protein (Matrix protein,Core protein,Viral occlusion body,Keratin) | Cluster 4144576  Region of unknown function DUF1732 |
| 104 | **P44003** | Beta-glucoside operon antiterminator -like domain ½ family | PTS-regulatory domain, PRD family | No result | PRD family | Cluster- 146476  (26S proteasome regulatory subunit) | No result | Nuclear Receptors | Cluster 3622930  PTS-regulatory domain, PRD |
| 105 | **P44005** | No result | No result | No result | SNARE_assoc family | Cluster- 141550  (Alkaline phosphatase) | SNARE_assoc Superfamily | Transmembrane | Cluster 4334463  SNARE associated Golgi protein |
| 106 | **O05023** | Nucleic acid binding protein -like domain family | Retroviral integrase, catalytic domain family  (Ribonuclease H-like superfamily) | Ferrichrome iron receptor-related family | rve_3 family | Cluster- 142960  (Transposase) | rve Superfamily | DNA recombination | Cluster 4067283  Integrase, catalytic core |
| 107 | **P44733** | No result | Myosin rod fragments family | DNA recombination protein rmuc family | RmuC family | Cluster-147263  (DNA recombination protein rmuC homolog) | RmuC Superfamily | All DNA-binding | Cluster 4170267  RmuC |
| 108 | **P44010** | No result | No result | No result | UPF0114 family | Cluster- 133284  (transmembrane ) | UPF0114 Superfamily/ AAA Superfamily | EC 2.4.-.-: Transferases - Glycosyltransferases | Cluster 4036464  Uncharacterised protein family UPF0114 |
| 109 | **P44740** | Nef-associated protein 1 -like domain family | YaeB-like family | Uncharacterized family | UPF0066 family | Cluster- 143774  (Formylmethanofuran dehydrogenase) | UPF0066 Superfamily | EC 2.5.-.-: Transferases - Transferring Alkyl or Aryl Groups, Other than Methyl Groups | Cluster 4130990  Uncharacterised protein family UPF0066 |
| 110 | **P44743** | ncharacterized protein HI_0520 -like domain family | MoCo biosynthesis proteins family  (Radical SAM enzymes superfamily) | Pyruvate formate-lyase-activating enzyme family | Fer4_12 family | Cluster- 146582  (Pyruvate formate-lyase 2 activating enzym) | Radical_SAM Superfamily | Iron-binding | Cluster 4152683 |
| 111 | **P44744** | Formate acetyltransferase -like domain family | PFL-like family  (PFL-like glycyl radical enzymes superfamily) | No result | Rad17 family | Cluster- 156410  (Yjji protein) | PFL Superfamily  (Pyruvate formate lyase) | Iron-binding | Cluster 4053911  Glycyl radical enzyme, HI0521, predicted |
| 112 | **Q57256** | No result | No result | No result | Uncharacterized BCR, YitT family | Cluster- 151901  (YCZE protein) | No result | Transmembrane | Cluster 4161004  Protein of unknown function DUF161 |
| 113 | **P44012** | Ribonuclease -like domain family | Ribonuclease Rh-like family | Ribonuclease T2 family | Ribonuclease T2 family | Cluster- 150158  (Ribonuclease T2 family) | RNase_T2 Superfamily | All lipid-binding proteins | Cluster 4158722  Ribonuclease Rh; Chain A |
| 114 | **P44013** | No result | No result | No result | G6PD_bact family | Cluster- 142546  (Sugar ABC transporter, sugar-binding protein) | G6PD_bact Superfamily | Zinc-binding | Cluster 3854686  Bacteria |
| 115 | **P44014** | Transposase for insertion sequence element IS200 -like domain family | Transposase IS200-like family | No result | Y1_Tnp family | Cluster-45444  (Putative glycosyl transferase) | Y1_Tnp Superfamily | EC 2.7.-.-: Transferases - Transferring Phosphorus-Containing Groups | Cluster 3969029  Complete proteome |
| 116 | **Q57409** | No result | No result | No result | DUF1304 family | Cluster- 156164  (transmembrane protein ) | DUF1304 superfamily | All lipid-binding proteins | Cluster 4069446  Protein of unknown function DUF1304 |
| 117 | **O86226** | No result | No result | No result | No result | Cluster- 95821  (Putative sugar transporter) | No result | EC 1.9.-.-: Oxidoreductases - Acting on a heme group of donors | Cluster 4046743  Haemophilus |
| 118 | **P44016** | No result | No result | Family not named | OPT family | Cluster- 142428  (Oligopeptide transporter, OPT family) | OPT Superfamily | Transmembrane | Cluster 4106511  Oligopeptide transporter OPT |
| 119 | **P44754** | 30S ribosomal protein S4 -like domain family | Heat shock protein 15 kD family  (Alpha-L RNA-binding motif superfamily) | RNA pseudouridylate synthase family protein | S4 family | Cluster- 148435  (Heat shock protein 15) | S4 Superfamily | RNA-binding Proteins | Cluster 4003495  RNA-binding S4 |
| 120 | **P44759** | Fusion glycoprotein F0 -like domain family | Fibrinogen coiled-coil and central regions family | No result | MerR-DNA-bind family | Cluster- 150246  (SlyX) | SlyX Superfamily | TC 1.C. Channels/Pores - Pore-forming toxins (proteins and peptides) | Cluster 4338006  SlyX |
| 121 | **P44761** | No result | No result | No result | HTH_22 family | Cluster- 138232  (Two-component sensor histidine kinase) | PAS_6 Superfamily | All lipid-binding proteins | Cluster 4245364  YheO-like |
| 122 | **P44017** | Sulfurtransferase TusD -like domain family | DsrH-like family | No result | DsrH family | Cluster- 156157  (DsrH protein ) | DsrH Superfamily | TC 3.A.1 ATP-binding cassette (ABC) family | Cluster 3815209  Sulphur relay, TusB/DsrH |
| 123 | **P44018** | No result | Amidase signature (AS) enzymes family | No result | DcuC family | Cluster- 149198  (C4-dicarboxylate transporter) | DcuC Superfamily | Transmembrane | Cluster 4107702  C4-dicarboxylate anaerobic carrier-like |
| 124 | **P44019** | No result | No result | No result | Ammonia monooxygenase  AMO family | Cluster- 149198  (C4-dicarboxylate transporter) | Dc-C Superfamily | Transmembrane | Cluster 4041962  Haemophilus influenzae |
| 125 | **P44023** | No result | No result | Solute carrier family 13 member family | DcuC family | Cluster- 147973  (Transporter) | COG1288 Superfamily | Transmembrane | Cluster 4117458  C4-dicarboxylate anaerobic carrier-like |
| 126 | **P44771** | Phosphoglycolate phosphatase -like domain family/  HMP-PP phosphatase -like domain family | Predicted hydrolases Cof family  (HAD-like superfamily) | Phosphoserine phosphatase family | Hydrolase_3 family | Cluster- 139306  (Hydrolase) | HAD_like Superfamily | All lipid-binding proteins | Cluster 4086008  HAD superfamily hydrolase-like, type 3 |
| 127 | **P44782** | No result | Pseudouridine synthase RsuA/RluD family | RNA pseudouridylate synthase family protein | PseudoU_synth_2 | Cluster- 137915  (Ribosomal large subunit pseudouridine synthase A) | PseudoU_synth Superfamily | Iron-binding | Cluster 4188164  Pseudouridine synthase, RsuA and RluB/C/D/E/F |
| 128 | **P44025** | Succinate dehydrogenase assembly factor 2, -like domain family | YgfY-like family | No result | Sdh5 family | Cluster- 150151  (Ygfy) | Sdh5 Superfamily | Sodium-binding | Cluster 4162414  Ygfy |
| 129 | **P44026** | Chloride channel protein -like domain family | Clc chloride channel family | Chloride channel family | Voltage_CLC family | Cluster-141500  (Chloride channel protein) | Voltage_gated_ClC Superfamily | Transmembrane | Cluster 3520476  Chloride transport |
| 130 | **P44027** | UPF0234 protein Rv0566c/MT0592 -like domain family | Integrin beta tail domain family | No result | Integrin_B_tail family | Cluster- 151454  (Capsid scaffolding protein) | PBPb Superfamily  (Bacterial periplasmic transport systems) | All lipid-binding proteins | Cluster 4141960  Conserved hypothetical protein CHP00743 |
| 131 | **P44796** | High frequency lysogenization protein HflD -like domain family | YcfC-like family | No result | Golgin subfamily A member 5 | Cluster- 156056  (ycfC protein) | DUF489 Superfamily | Transmembrane | Cluster 3916515  YcfC-like |
| 132 | **P44028** | No result | No result | No result | Prokaryotic membrane lipoprotein lipid attachment site  LPAM_1 family | Cluster- 156185  (lipoprotein) | DUF1375 Superfamily | Photosynthesis | Cluster 4094730  Protein of unknown function DUF1375 |
| 133 | **P44807** | tRNA threonylcarbamoyladenosine biosynthesis -like domain family | YrdC-like family | SUA5 family | Sua5_yciO_yrdC family | Cluster- 145750  (Sua5/YciO/YrdC family protein) | Sua5_yciO_yrdC Superfamily | RNA-binding Proteins | Cluster 4038495  Sua5/YciO/YrdC, N-terminal |
| 134 | **P46494** | DNA topoisomerase -like domain 1/2/3 family | PhnA zinc-binding domain family/  Prokaryotic DNA topoisomerase I, a C-terminal fragment family | Prokaryotic dna topoisomerase family | zf-C4_Topoisom family | Cluster- 144826  (DNA topoisomerase I) | TOPRIM Superfamily | Zinc-binding | Cluster 4554047  Prokaryotic type I DNA topoisomerase |
| 135 | **P44031** | No result | Glu-tRNAGln amidotransferase C subunit family | No result | Gp49 family | Cluster- 156920  (putative phage protein ) | Gp49 Superfamily | mRNA-binding Proteins | Cluster 4727452  Protein of unknown function DUF891 |
| 136 | **P44033** | No result | No result | No result | HipA_N family | Cluster- 156136  (Protein hipA) | HipA_N Superfamily/ HipA_C Superfamily | Zinc-binding | Cluster 4088874  HipA-like, N-terminal |
| 137 | **P44034** | No result | No result | No result | Couple_hipA family | Cluster- 154923  (ComK) | Couple_hipA Superfamily | Iron-binding | Cluster 4033498  HipA, N-terminal |
| 138 | **O86228** | DNA-binding protein -like domain family | ambda repressor-like DNA-binding domains superfamily | No result | HTH_3 family | Cluster- 145529  (Transcriptional regulator, Hth-3 family) | HTH_XRE Superfamily | TC 9.A. Incompletely Characterized Transport Systems - Recognized transporters of unknown biochemical mechanism | Cluster 3898899  Helix-turn-helix type 3 |
| 139 | **P44812** | No result | Tropomyosin family | No result | Nucleopolyhedrovirus P10 protein  NPV_P10 family | Cluster- 138169  (A kinase anchor protein) | DUF904 Superfamily | All lipid-binding proteins | Cluster 4106976  Protein of unknown function DUF904 |
| 140 | **P44036** | Mycothiol acetyltransferase -like domain 1/2/3 family | N-acetyl transferase, NAT family | N-terminal acetyltransferase family | Acetyltransf_1 family | Cluster- 146722  (Acetyltransferases) | No result | Iron-binding | Cluster 4032240  N-acetyltransferase activity |
| 141 | **P71356** | No result | Multidrug resistance efflux transporter EmrE family | Family not named | EamA-like transporter family | Cluster- 144807  (Integral membrane domain protein) | No result | Transmembrane | Cluster 4048630  Protein of unknown function DUF6, transmembrane |
| 142 | **P44037** | No result | Peptide methionine sulfoxide reductase family | No result | PAS_7 family | Cluster- 19036  (hypothetical protein ) | No result | EC 3.1.-.-: Hydrolases - Acting on Ester Bonds | Cluster 3826314  NAD(P)-binding Rossmann-like Domain |
| 143 | **P44827** | No result | Pseudouridine synthase RsuA/RluD family | Ribosomal large subunit pseudouridine synthase bfamily | PseudoU_synth_2 family | Cluster- 141200  (Ribosomal large subunit pseudouridine synthase B) | PseudoU_synth Superfamily | rRNA-binding Proteins | Cluster 4152309  Pseudouridine synthase, RsuA and RluB/E/F |
| 144 | **Q57523** | No result | No result | No result | Family of unknown function (DUF490) | Cluster- 143388  (Putative signal peptide protein) | DUF490 Superfamily | All lipid-binding proteins | Cluster 4141982  Protein of unknown function DUF490 |
| 145 | **P44038** | Outer membrane protein assembly factor yaeT -like domain family | No result | Sorting and assembly machinery (sam50) protein family | Bac_surface_Ag family | Cluster- 141007  (outer membrane protein) | Bac_surface_Ag Superfamily | Zinc-binding | Cluster 4169983  Bacterial surface antigen (D15) |
| 146 | **P44831** | Regulator of ribonuclease activity B -like domain family | Hypothetical protein VC0424 family | No result | RraB family | Cluster- 155971  (yjgD protein) | PRK11191 Superfamily | Metal-binding | Cluster 3797359  Uncharacterised conserved protein UCP018193 |
| 147 | **P44040** | No result | No result | No result | LPAM_1 family | Cluster- 152724  (utative lipoprotein) | No result | Nickel-binding | Cluster 3904479  Pasteurellales |
| 148 | **P71357** | No result | No result | No result | RelB family | Cluster- 150903  (Damage-inducible protein DinJ) | RelB Superfamily | All lipid-binding proteins | Cluster 4023654  RelB antitoxin |
| 149 | **P44041** | No result | RelE-like family | No result | Plasmid_stabil family | Cluster- 119867  (Capsid assembly protein) | COG3041 Superfamily | Repressor | Cluster 4099600  Addiction module toxin, RelE/StbE |
| 150 | **P44839** | RutC family protein yjgF -like domain family | YjgF/L-PSP family | Translation initiation inhibitor family | Ribonuc_L-PSP family | Cluster- 141110  (Probable translation initiation inhibitor) | YjgF_YER057c_UK114_family Superfamily | All lipid-binding proteins | Cluster 4195840  Endoribonuclease L-PSP |
| 151 | **P44842** | Elongation factor G -like domain family | YigZ N-terminal domain-like family  (Ribosomal protein S5 domain 2-like superfamily/  EF-G C-terminal domain-like superfamily) | Impact-related family | UPF0029 family | Cluster- 146637  (Proline dipeptidase) | UPF0029 superfamily | Zinc-binding | Cluster 4126851  Uncharacterised protein family UPF0029 |
| 152 | **P44844** | No result | No result | No result | YihI family | Cluster- 156394  (putative coproporphyrinogen III oxidase) | YihI Superfamily | Metal-binding | Cluster 4040724  Protein of unknown function DUF414 |
| 153 | **P44043** | No result | No result | No result | DUF2489 family | Cluster- 156197  (putative coproporphyrinogen III oxidase) | DUF2489 superfamily | Transmembrane | Cluster 4020603  Domain of unknown function DUF2489 |
| 154 | **P44045** | No result | No result | No result | No result | Cluster- 155912  (DNA-binding protein UL42) | No result | Photosynthesis | Cluster 4138486  Multicopper oxidase, type 2 |
| 155 | **P44047** | No result | No result | No result | No result | Cluster- 142291  (nucleoside-diphosphate-sugar epimerases) | NADB_Rossmann Superfamily | Zinc-binding | Cluster 3507333  Haemophilus influenzae |
| 156 | **P44854** | Centrosomal protein of 41 kDa -like domain family | Single-domain sulfurtransferase family | Family not named | Rhodanese family | Cluster- 143086  (Rhodanese-related sulfurtransferase) | RHOD Superfamily | All lipid-binding proteins | Cluster 4116016  Oxidized Rhodanese; domain 1 |
| 157 | **P44863** | Polysaccharide deacetylase -like domain family | Divergent polysaccharide deacetylase family | Uncharacterized yibq-related | Polysacc_deac_2 family | Cluster- 133511  (YibQ) | CE4_SF Superfamily | EC 3.4.-.-: Hydrolases - Acting on peptide bonds (Peptidases) | Cluster 4078228  Protein of unknown function DUF610, YibQ |
| 158 | **P44864** | Lipoprotein -like domain family | Peptidoglycan hydrolase LytM family | Peptidase-related family | Peptidase_M23 family | Cluster- 139239  (Peptidase, M23/M37 family) | Peptidase_M23 Superfamily | Zinc-binding | Cluster 4145059  Peptidase M23B |
| 159 | **P44048** | Probable Fe(2+)-trafficking protein -like domain family | YggX-like family | No result | Iron_traffic family | Cluster- 156159  (Protein yggX) | Bacterial Fe(2+) trafficking | Iron-binding | Cluster 4093460  YggX-like |
| 160 | **P44050** | Double-strand break repair protein MRE11 -like domain family | Metallo-dependent phosphatases superfamily | No result | Metallophos_2 family | Cluster- 87121  (Phosphoesterase) | MPP_superfamily Superfamily | Iron-binding | Cluster 4733582  Metallophosphoesterase |
| 161 | **P44869** | Ribosomal RNA small subunit methyltransferase D -like domain family | S-adenosyl-L-methionine-dependent methyltransferases superfamily | Methyltransferase family | Cons_hypoth95 family | Cluster- 142947  (Methyltransferase) | AdoMet_MTases Superfamily | EC 2.1.-.-: Transferases - Transferring One-Carbon Groups | Cluster 4136223  Conserved hypothetical protein CHP00095 |
| 162 | **P44052** | No result | DBL homology domain (DH-domain) family | No result | No result | Cluster- 112385  (Ser/Thr protein kinase) | No result | Lipoprotein | Cluster 3971366  Complete proteome |
| 163 | **P44053** | cAMP-dependent protein kinase regulatory subunit -like domain ½ family | cAMP-binding domain family | Family not named | cNMP_binding family | Cluster- 143194  (Transcriptional activator, CrP family) | CAP_ED Superfamily | Metal-binding | Cluster 3773754  Cyclic nucleotide-binding |
| 164 | **P44054** | No result | No result | Transmembrane protein YFCA family | TauE family | Cluster-150623  (Integral membrane protein) | No result | Metal-binding | Cluster 3598299  Protein of unknown function DUF81 |
| 165 | **P44882** | No result | YgfB-like family | No result | TerB-N family | Cluster- 156212  (ygfB) | UPF0149 Superfamily | All lipid-binding proteins | Cluster 3976330  Uncharacterised protein family UPF0149 |
| 166 | **P44056** | No result | No result | No result | Yip1 family  (Golgi vesicular transport protein) | Cluster- 80365  (Cytochrome oxidase II) | No result | Transmembrane | Cluster 3946111  Uncharacterised protein family UPF0259 |
| 167 | **P44886** | Acyl-coenzyme A thioesterase 11 -like domain ½ family | Thioesterase/thiol ester dehydrase-isomerase superfamily | Cytosolic acyl coenzyme a thioester hydrolase family | Thioesterase superfamily | Cluster- 147823  (Acyl-CoA hydrolase) | hot_dog Superfamily | EC 3.1.-.-: Hydrolases - Acting on Ester Bonds | Cluster 3929807  Thioesterase superfamily |
| 168 | **P44897** | uncharacterised family 67 -like domain | YejL-like family | No result | Na_H_antiport_2 family | Cluster- 152675  (yejL like ) | DUF1414 Superfamily | All DNA-binding | Cluster 4020600  Uncharacterised protein family UPF0352 |
| 169 | **P44898** | Inner membrane protein yejM -like domain family | Alkaline phosphatase family | Arylsulfatase family | Sulfatase family | Cluster- 151985  (hydrolase of alkaline phosphatase superfamily) | Sulfatase Superfamily/ DUF3413 Superfamily | Transmembrane | Cluster 3463167  Membrane sulfatase, HI0842-related |
| 170 | **P44058** | Cytosine deaminase -like domain family | Cytosine deaminase catalytic domain family | Family not named | CutC family | Cluster- 148111  (Ammelide aminohydrolase) | metallo-dependent_hydrolases Superfamily | Iron-binding | Cluster 3692882  Haemophilus |
| 171 | **P44059** | Dihydroorotase -like domain 2 family | No result | No result | No result | Cluster- 111725  (GTPase activator ) | No result | No result | Cluster 3667317  Nocardiopsaceae |
| 172 | **P44900** | No result | No result | No result | SEN1_N family  (helicase Sen1 is an RNA polymerase II termination factor) | Cluster- 135315  (YIHD) | DUF1040 Superfamily | Calcium-binding | Cluster 3673366  Protein of unknown function DUF1040 |
| 173 | **P31811** | No result | No result | No result | Nucleolar RNA-binding protein, Nop10p family | Cluster- 156393  (Putative LysR type transcriptional regulator with pssR) | DUF413 Superfamily | DNA repair | Cluster 4034123  Protein of unknown function DUF413 |
| 174 | **P44903** | Putative multidrug resistance protein MdtD -like domain family | LacY-like proton/sugar symporter family | Major facilitator superfamily domain-containing protein-related family | Major Facilitator Superfamily | Cluster- 140481  (Multidrug resistance protein) | MFS Superfamily | TC 2.A. Electrochemical Potential-driven transporters - Porters (uniporters, symporters, antiporters) | Cluster 4109456  Drug:hydrogen antiporter activity |
| 175 | **P44904** | No result | No result | Uncharacterized family | SDH_alpha family  (Serine dehydratase alpha chain) | Cluster- 156227  (inner membrane protein) | SDH_alpha Superfamily | Metal-binding | Cluster 4318831  Uncharacterised protein family UPF0597 |
| 176 | **P44062** | No result | Cell division protein ZapA-like family | No result | ZapA family | Cluster- 136610  (Kinectin (Fragment)) | ZapA Superfamily | Transmembrane | Cluster 4068282  Cell division protein ZapA-like |
| 177 | **P44905** | Lactate utilization protein B -like domain family | Methenyltetrahydrofolate synthetase family | ATPase inhibitor/5-formyltetrahydrofolate cyclo-ligase family | 5-formyltetrahydrofolate cyclo-ligase family | Cluster- 150145  (5-formyltetrahydrofolate cyclo-ligase) | COG0212 Superfamily | Repressor | Cluster 4172270  5-formyltetrahydrofolate cyclo-ligase activity |
| 178 | **P44908** | No result | No result | No result | YhhQ family | Cluster- 156383  (Transporter) | DUF165 Superfamily | TC 2.A. Electrochemical Potential-driven transporters - Porters (uniporters, symporters, antiporters) | Cluster 4290160  Protein of unknown function DUF165 |
| 179 | **P44063** | Lipopolysaccharide biosynthesis protein -like domain family | Bacterial polysaccharide co-polymerase-like family | No result | Baculo_E56 family | Cluster- 148453  (Chain length determinant protein (Polysaccharide antigen chain regulator)) | No result | Transmembrane | Cluster 4119936  Lipopolysaccharide biosynthesis |
| 180 | **Q57022** | Polypeptide N-acetylgalactosaminyltransferase 1 -like domain family | Nucleotide-diphospho-sugar transferases superfamily | Glycosyltransferase family | Glycosyl transferase family 2 | Cluster- 139894  (Glycosyl transferase) | Glyco_tranf_GTA_type Superfamily | EC 2.4.-.-: Transferases - Glycosyltransferases | Cluster 4204043  Glycosyl transferase, family 2 |
| 181 | **P44064** | Polypeptide N-acetylgalactosaminyltransferase 1 -like domain family | Nucleotide-diphospho-sugar transferases superfamily | No result | Glycosyl transferase family 2 | Cluster – 96009  (Glycosyl transferase ) | Glyco_tranf_GTA_type Superfamily | EC 3.1.-.-:Hydrolases- scting on ester bond | Cluster 3685481  Glycosyl transferase family 2 |
| 182 | **P44065** | No result | No result | No result | No result | Cluster – 100590  (Hypothetical protein) | No result | No result | Cluster 3685481  Glycosyl transferase family 2 |
| 183 | **P44067** | No result | No result | No result | WzyC family | Cluster – 155051  (O-Antigen ligase) | Wzy_C Superfamily  (O-antigen ligase) | Transmembrane | Cluster 4170778  O-Antigen ligase-realated |
| 184 | **P71360** | No result | Multidrug resistance efflux transporter EmrE family | Acyl-malonyl condensing enzyme-related family | EamA-like transporter family | Cluster-144807  (integral membrane protein ) | EamA Superfamily | Transmembrane | Cluster-3853107  Protein of unknown function DUF6, transmembrane |
| 185 | **P44068** | No result | N-type ATP pyrophosphatases family | No result | DUF208 family | Cluster – 15145  (Hypothetical protein ) | DUF208 Superfamily | Zinc binding | Cluster 4025260  Protein of unknown function DUF208 |
| 186 | **P44069** | No result | No result | No result | DoxX family | Cluster-149076  (Integral membrane protein) | DoxX Superfamily | Transmembrane | Cluster 3059231  DoxX |
| 187 | **P44070** | No result | No result | Transmembrane protein YFCA family | TauE family | Cluster – 150623  (Transporter) | No result | Transmembrane | Cluster 4117461  Protein of unknown function DUF81 |
| 188 | **P44931** | dCMP deaminase -like domain family | Deoxycytidylate deaminase-like family | Cytosine deaminase family | dCMP cyt deam 1 family | Cluster – 143335  (tRNA specific adenosine deaminase ) | cytidine_deaminase-like Superfamily | All lipid binding protein | Cluster 4151600  CMP/dCMP deaminase , zinc binding |
| 189 | **P44072** | No result | No result | No result | DUF721 family | Cluster – 132995  (RNA replicase (fragment )) | DUF721 Superfamily | Metal binding | Cluster 3748936  Protein of unknown function DUF721 |
| 190 | **P44073** | No result | No result | No result | DUF2547 family | Cluster – 132575  (WBS15 splice variant 3) | DUF2547 Superfamily | All lipid binding proteins | Cluster 4354641  Pasteurellales |
| 191 | **P44074** | tRNA (mo5U34)-methyltransferase -like domain family | S-adenosyl-L-methionine-dependent methyltransferases superfamily | Methyltransferase family | Methytransf 11 family | Cluster – 143210  (Mthyltransferase) | AdoMet_MTases Superfamily | Zinc binding | Cluster 3959076  Methytransferase type 11 |
| 192 | **P44936** | GTPase-activating protein -like domain 1/2/3/4/5/6/7/8/9/10/11/12/13 family | PDZ domain-like superfamily | Serine protease family s1c htra-related family | Peptidase M50 family | Cluster – 147367  (Zinc metalloprotease ) | S2P-M50 Superfamily/PDZ Superfamily | Transmembrane /Zinc binding | Cluster 4147222  Peptidase M50 , putativeassociated zinc metallopeptidase |
| 193 | **P44938** | Undecaprenyl pyrophosphate synthase -like domain family | Undecaprenyl diphosphate synthase family | Dehydrodolichyl diphosphate synthase family | Prenyltransf family | Cluster – 136904  (Undecaprenyl pyrophosphate synthase ) | CIS_IPPS Superfamily | EC 2.5.-.-: Transferases - Transferring Alkyl or Aryl Groups, Other than Methyl Groups | Cluster 4159268  Di-trans,poly-cis-decaprenylcistransferase |
| 194 | **P44075** | No result | Restriction endonuclease-like superfamily | No result | Domain of unknown function DUF559 family | Cluster – 144166  (DNA methylase) | Restriction_endonuclease_like Superfamily | DNA replication | Cluster 4054936  Domain of unknown function DUF559 |
| 195 | **P44076** | No result | Thioltransferase family | No result | Glutaredoxin family | Cluster – 142128  (Glutaredoxin) | COG4545 Superfamily | Outer membrane | Cluster4071603  Uncharacterised conserved protein UCP037291, glutaredoxin-related |
| 196 | **P44940** | No result | Glutathionylspermidine synthase ATP-binding domain-like family | Bifunctional glutathionylspermidine synthetase/amidase-related family | GSP_synth family | Cluster – 144944  (Glutathionylspermidine synthase) | GSP_synth Superfamily | Iron-binding | Cluster3943042  Glutathionylspermidine synthase |
| 197 | **P44077** | No result | No result | No result | No result | Cluster – 135599  (Lipoprotein ) | No result | Zinc-binding | Cluster4081953  Gammaproteobacteria |
| 198 | **P44078** | No result | Iron-dependent repressor protein, dimerization domain family | No result | DUF2251 family | Cluster – 151329  (Replication protein) | DUF2251 Superfamily | All lipid-binding proteins | Cluster4318060  Uncharacterised conserved protein UPC007050 |
| 199 | **P44941** | Phytoene dehydrogenase -like domain family/  Pyridine nucleotide-disulfide oxidoreductase -like domain family | FAD/NAD(P)-binding domain superfamily | Electron transfer flavoprotein-ubiquinone oxidoreductase family | HI0933_like family | Cluster – 148689  (flavoprotein) | NAD_binding_8 Superfamily | All lipid-binding proteins | Cluster 4145478  HI0933-like protein |
| 200 | **P44079** | No result | Pili subunits superfamily | No result | N_methyl_2 family | Cluster – 154385  (alpha-1,3-mannosyltransferase mnn1) | No result | Transmembrane | Cluster4057757  Prepilin-type cleavage/methylation, N-terminal |
| 201 | **P44080** | No result | No result | No result | No result | Cluster – 120178  (Hypothetical protein ) | No result | Transmembrane | Cluster4140983  Prepilin peptidase-dependent protein B, predicted |
| 202 | **P44081** | No result | No result | No result | 10 TM Acyl Transferase domain found in Cas1p (Cas1_AcylT family ) | Cluster – 137834  (Serine protease) | DUF2572 Superfamily | Transmembrane | Cluster3669568  Pasteurellaceae |
| 203 | **P44082** | No result | No result | No result | Type II secretory pathway pseudopilin  (PulG family) | Cluster – 120180  (Hypothetical protein) | No result | Transmembrane | Cluster4085812  Pasteurellales |
| 204 | **Q57120** | No result | Kis/PemI addiction antidote family | No result | Antitoxin-MazE family | Cluster – 155736  (Virulence-associated protein B) | VagC Superfamily | Iron-binding | Cluster4064538  Proteobacteria |
| 205 | **P44954** | uncharacterised family 128 -like domain | YehU-like | No result | Mei5 family | Cluster – 133503  (yheU like ) | UPF0270 Superfamily | Zinc-binding | Cluster4107858  Hypothetical upf0270 protein pa3463 |
| 206 | **P44084** | No result | No result | No result | LPAM_1 family | Cluster – 151115  (lipoprotein ) | DUF1425 Superfamily | EC 3.8.-.-: Hydrolases - Acting on Halide Bonds | Cluster3849717  Pasteurellales |
| 207 | **P44085** | No result | No result | No result | No result | Cluster – 120184  (Hypothetical protein ) | No result | All lipid-binding proteins | Cluster4160927  Complete proteome |
| 208 | **P44086** | No result | No result | No result | No result | Cluster – 19225  (Hypothetical protein) | No result | No result | No result |
| 209 | **Q57133** | No result | GNA1870 immunodominant domain-like family | No result | Lipoprotein_5 family | Cluster – 148872  (Transferrin binding protein 2) | Lipoprotein_5 Superfamily | All lipid-binding proteins | Cluster 4628469  Transferrin receptor activity |
| 210 | **P46455** | No result | No result | No result | Tic20-like protein family | Cluster – 156381  (Integral membrane protein) | DUF997 Superfamily | Transmembrane | Cluster3974290  Protein of unknown function DUF997 |
| 211 | **Q57147** | No result | Multidrug resistance efflux transporter EmrE family | Family not named | EamA-like transporter family | Cluster – 143722  (Tou6 like ) | EamA Superfamily | Transmembrane | Cluster3692408  Protein of unknown function DUF6, transmembrane |
| 212 | **O86230** | No result | Multidrug resistance efflux transporter EmrE family | Family not named | EamA-like transporter family | Cluster – 143722  (Tou6 like ) | EamA Superfamily | Transmembrane | Cluster 3692408  Protein of unknown function DUF6, transmembrane |
| 213 | **P44965** | Tryptophan synthase -like domain family/  tRNA-dihydrouridine synthase -like domain | FMN-linked oxidoreductases family | TRNA-dihydrouridine synthase family | Dihydrouridine synthase (Dus family ) | Cluster – 147656  (TIM-barrel protein) | TIM_phosphate_binding Superfamily | Zinc-binding | Cluster4154178  TRNA-dihydrouridine synthase |
| 214 | **P43907** | No result | No result | No result | LPAM_1 family | Cluster – 133395  (signal peptide protein ) | No result | EC 3.1.-.-: Hydrolases - Acting on Ester Bonds | Cluster3889417  Pasteurellales |
| 215 | **P43908** | No result | No result | Peroxide stress response protein YAAA family | Toxin_65 family | Cluster – 151722  (Protein yaaA) | DUF328 Superfamily | EC 2.5.-.-: Transferases - Transferring Alkyl or Aryl Groups, Other than Methyl Groups | Cluster4120731  Protein of unknown function DUF328 |
| 216 | **P44972** | No result | No result | No result | Haemolytic family | Cluster – 151228  (Hypothetical protein ) | Haemolytic Superfamily | Metal-binding | Cluster4114102  Protein of unknown function DUF37 |
| 217 | **P44974** | Phosphoglycerol transferase I -like domain | Arylsulfatase family  (Alkaline phosphatase-like superfamily) | Inner membrane protein family | Sulfatase family | Cluster – 151987  (Integral membrane protein) | Sulfatase Superfamily | Transmembrane | Cluster4200264  Sulfatase |
| 218 | **Q57134** | ranscription elongation factor, mitochondrial -like domain 1/2  family | RuvA domain 2-like superfamily | Family not named | HHH_3 family | Cluster – 144900  (ComE operon protein 1) | No result | No result | Cluster4161060  Competence protein ComEA, helix-hairpin-helix region |
| 219 | **P44093** | No result | YgbK-like family | D-tagatose-1,6-bisphosphate aldolase family | DUF1537 family | Cluster – 149258  (ygbK like ) | DUF1537 Superfamily | EC 4.1.-.-: Lyases - Carbon-Carbon Lyases | Cluster4160211  Protein of unknown function, DUF1537 |
| 220 | **Q57151** | Hydroxypyruvate isomerase -like domain family | Xylose isomerase-like superfamily | Hydroxypyruvate isomerase family | AP_endonuc_2 family | Cluster – 148356  (Hydroxypyruvate isomerase) | AP2Ec Superfamily | EC 2.4.-.-: Transferases - Glycosyltransferases | Cluster4151021  Hydroxypyruvate isomerase |
| 221 | **P44094** | CDP-abequose synthase -like domain family/  NAD dependent epimerase/dehydratase family protein -like domain | Tyrosine-dependent oxidoreductases family | NAD dependent epimerase/dehydratase family | Epimerase family | Cluster – 142884  (Nucleoside-diphosphate-sugar epimerase) | NADB_Rossmann Superfamily | EC 1.1.-.-: Oxidoreductases - Acting on the CH-OH group of donors | Cluster4107863  NAD-dependent epimerase/dehydratase |
| 222 | **P44095** | Kynurenine formamidase -like domain family | Putative cyclase family | No result | Cyclase family | Cluster – 153826  (Cyclase) | Cyclase Superfamily | Transmembrane | Cluster4102526  Putative cyclase |
| 223 | **P44992** | No result | Phosphate binding protein-like family | No result | SBP_bac_7 family | Cluster – 147377  (TRAP-type C4-dicarboxylate transport system, periplasmic component) | SBP_bac_7 Superfamily | EC 2.4.-.-: Transferases - Glycosyltransferases | Cluster4180508  Extracellular solute-binding protein, family 7, bacteria |
| 224 | **P44993** | No result | No result | Family not named | DctM family | Cluster – 147373  (TRAP dicarboxylate transporter, DctM subunit) | DctQ Superfamily | Transmembrane | Cluster4101809  TRAP dicarboxylate transporter, DctM subunit |
| 225 | **P44994** | No result | No result | Family not named | DctQ family | Cluster – 147376  (Permease component of C4 dicarboxylate transporter) | DctQ Superfamily | Transmembrane | Cluster 4101809  TRAP dicarboxylate transporter, DctM subunit |
| 226 | **P44097** | No result | No result | No result | YGGT family | Cluster – 154128  (YGGT family protein ) | YGGT superfamily | Transmembrane | Cluster4133866  Protein of unknown function YGGT |
| 227 | **P44098** | Asparagine synthetase [glutamine-hydrolyzing] -like domain ½ family | Class II glutamine amidotransferases family  (N-terminal nucleophile aminohydrolases (Ntn hydrolases) superfamily) | Putative glutamine amidotransferase yafj family | GATase_4 family | Cluster – 148742  (glutamine amidotransferase ) | Gn_AT_II Superfamily | All lipid-binding proteins | Cluster3979557  Amidophosphoribosyl transferase |
| 228 | **P44099** | Origin recognition complex protein 5 -like domain family | P-loop containing nucleoside triphosphate hydrolases superfamily | No result | AAA_14 family | Cluster – 151461  (ATPase) | AAA_14 Superfamily | EC 2.4.-.-: Transferases - Glycosyltransferases | Cluster4154065  Nucleotide binding |
| 229 | **P44103** | Transglutaminase family protein -like domain family | Transglutaminase core family | No result | Transglut_core family | Cluster – 148463  (Transglutaminase-like enzymes) | Transglut_core Superfamily | All lipid-binding proteins | Cluster3513118  Transglutaminase-like |
| 230 | **Q57498** | Carboxymuconolactone decarboxylase family protein -like domainfamily | AhpD-like superfamily | No result | CMD family | Cluster – 147110  (Carboxymuconolactone decarboxylase) | CMD Superfamily | All lipid-binding proteins | Cluster4134856  Alkylhydroperoxidase AhpD core |
| 231 | **P44104** | No result | No result | No result | No result | Cluster – 128052  (Type III restriction-modification system: methylase) | No result | Zinc-binding | Cluster3755017  Gammaproteobacteria |
| 232 | **P44106** | No result | Tail-associated lysozyme gp5, C-terminal domain family | No result | TypeIII_RM_methfamily | Cluster – 102844  (Hemolysin) | TypeIII_RM_meth Superfamily | All lipid-binding proteins | Cluster4024927  DNA methylase N-4/N-6 |
| 233 | **P44107** | No result | No result | No result | No result | Cluster – 19264  (Hypothetical protein ) | No result | All DNA-binding | Cluster4039085  Sulfuric ester hydrolase activity |
| 234 | **P71367** | Phosphoglycerol transferase I -like domain family | Arylsulfatase family | Inner membrane protein family | Sulfatase family | Cluster – 151987  (Membrane-associated metal-dependent hydrolase) | Sulfatase Superfamily | Transmembrane | Cluster4057510  Sulfatase |
| 235 | **P45019** | No result | Terpenoid cyclases/Protein prenyltransferases superfamily | Uncharacterized family | DUF423 family | Cluster – 143810  (Small membrane protein) | DUF423 Superfamily | Transmembrane | Cluster4653268  Protein of unknown function DUF423 |
| 236 | **P44110** | No result | No result | No result | DUF441 family | Cluster – 156337  (inner membrane protein) | DUF441 superfamily | Transmembrane | Cluster4066607  Protein of unknown function DUF441, transmembrame |
| 237 | **P45026** | BolA protein -like domain family | BolA-like family | BOLA-like protein-related family | BolA family | Cluster – 144957  (Transcriptional regulator (BolA family)) | BolA Superfamily | All DNA-binding | Cluster4143345  BolA-like |
| 238 | **P44111** | No result | No result | No result | DUF3884 family | Cluster – 19279  (Hypothetical protein ) | No result | No result | Cluster3780104  Haemophilus influenzae |
| 239 | **P44112** | No result | No result | No result | LPAM_1 family | Cluster – 120189  (Hypothetical protein ) | LPAM_1 Superfamily | Hormone | Cluster4092229  Pasteurellales |
| 240 | **P45071** | UPF0042 nucleotide-binding protein CMS1991 -like domain family | P-loop containing nucleoside triphosphate hydrolases superfamily | Uncharacterized family | ATP_bind_2 family | Cluster – 156331  (P-loop-containing kinase) | ATP_bind_2 Superfamily | Metal-binding | Cluster4121952  ATPase, P-loop-containing |
| 241 | **P45074** | LPS-assembly protein lptD -like domain family | No result | No result | OstA family | Cluster – 156101  (ABC superfamily (bind_prot) transport protein) | OstA Superfamily | All lipid-binding proteins | Cluster4049509  Cell envelope biogenesis YhbN |
| 242 | **P45075** | No result | No result | No result | LptC family | Cluster – 156487  (YrbK protein) | COG3117 Superfamily | Transmembrane | Cluster4193210  Protein of unknown function DUF1239 |
| 243 | **P45076** | UPF0307 protein yjgA -like domain 1 family | PSPTO4464-like family | No result | DUF615 family | Cluster – 148809  (yjgA protein) | DUF615 superfamily | All DNA-binding | Cluster4156047  Uncharacterised protein family UPF0307 |
| 244 | **P45077** | No result | Putative modulator of DNA gyrase, PmbA/TldD family | Uncharacterized protein TLDD AND PMBA family | PmbA_TldD family | Cluster – 155408  (PmbA protein) | No result | All lipid-binding proteins | Cluster4162426  Peptidase U62, modulator of DNA gyrase |
| 245 | **P45083** | 1,4-dihydroxy-2-naphthoyl-CoA hydrolase -like domain family | Thioesterase/thiol ester dehydrase-isomerase superfamily | Family not named | 4HBT family | Cluster – 147916  (Thioesterase) | hot_dog Superfamily | All lipid-binding proteins | Cluster4177136  Phenylacetic acid degradation-related protein |
| 246 | **P44116** | No result | Restriction endonuclease-like superfamily | No result | DUF559 family | Cluster –144166  (DNA methylase) | Restriction_endonuclease_like Superfamily | Zinc-binding | Cluster 4054936  Protein of unknown function DUF559 |
| 247 | **Q57252** | UDP-N-acetylenolpyruvoylglucosamine reductase -like domain family/  NADH-quinone oxidoreductase subunit I -like domain family | FAD-linked oxidases, C-terminal domain / FAD-binding/transporter-associated domain-like/ alpha-helical ferredoxin superfamily | D-lactate dehydrogenase family | FAD_binding_4 family | Cluster – 140820  (Oxidoreductase) | FAD_binding_4 Superfamily | Zinc-binding | Cluster4104308  FAD-linked oxidase, C-terminal |
| 248 | **P45085** | AT4G10000 protein -like domain ½ family | Thioltransferase family | Glutaredoxin-related protein family | Glutaredoxin family | Cluster – 141965  (Glutaredoxin-related protein) | Thioredoxin_like Superfamily | EC 2.4.-.-: Transferases - Glycosyltransferases | Cluster4112638  Glutaredoxin-related protein |
| 249 | **P44117** | AT4G10000 protein -like domain ½ family | Triger factor/SurA peptide-binding domain-like superfamily | No result | DUF496 family | Cluster – 152251  (Putative alpha helix protein) | DUF496 superfamily | TC 2.C. Electrochemical Potential-driven transporters - Ion-gradient-driven energizers | Cluster3591848  Protein of unknown function DUF496 |
| 250 | **P44119** | No result | No result | No result | SprT-like family | Cluster – 133019  (Protein sprT) | SprT Superfamily | Zinc-binding | Cluster4075447  Protein of unknown function DUF335, SprT |
| 251 | **P45097** | Predicted organic radical activating enzyme -like domain family | Radical SAM enzymes superfamily | Family not named | Fer4_14 family  (Radical SAM proteins) | Cluster – 148455  (Radical activating enzyme) | Radical_SAM Superfamily | Iron-binding | Cluster4145506  Radical SAM |
| 252 | **P44124** | 7-cyano-7-deazaguanine synthase -like domain family | N-type ATP pyrophosphatases family | No result | QueC family | Cluster – 147255  (PP-loop superfamily ATPase) | AANH_like Superfamily | EC 2.7.-.-: Transferases - Transferring Phosphorus-Containing Groups | Cluster3843800  Exoenzyme S synthesis protein B/queuosine synthesis |
| 253 | **P44125** | No result | No result | No result | No result | Cluster – 19329  (Hypothetical protein ) | No result | No result | Cluster3984753  Pasteurellales |
| 254 | **P45103** | tRNA threonylcarbamoyladenosine biosynthesis -like domain family | YrdC-like family | SUA5 family | Sua5_yciO_yrdC family | Cluster – 145750  (SUA5/yciO/yrdC family protein) | Sua5_yciO_yrdC Superfamily | Metal-binding | Cluster3913974  Sua5/YciO/YrdC/YwlC |
| 255 | **P45104** | 30S ribosomal protein S4 -like domain family | Pseudouridine synthase RsuA/RluD family | RIbosomal large subunit pseudouridine synthase b family | S4 family | Cluster – 141200  (Ribosomal large subunit pseudouridine synthase B) | PseudoU_synth Superfamily | RNA-binding Proteins | Cluster4152309  Pseudouridine synthase, RsuA and RluB/E/F |
| 256 | **P44126** | No result | SMR domain-like superfamily | No result | Smr family | Cluster – 147429  (Smr domain protein) | Smr Superfamily | Nuclear Receptors | Cluster3813237  Smr protein/MutS2 C-terminal |
| 257 | **P44127** | No result | No result | No result | DUF412 family | Cluster – 156191  (yfbV protein) | DUF412 Superfamily | Transmembrane | Cluster3935154  Uncharacterised protein family UPF0208 |
| 258 | **P71373** | Putative uncharacterized protein -like domain family | Tyrosine-dependent oxidoreductases family | Sugar nucleotide epimerase related family | Epimerase family | Cluster – 146743  (Cell-division inhibitor) | NADB_Rossmann Superfamily | EC 2.7.-.-: Transferases - Transferring Phosphorus-Containing Groups | Cluster3947300  NAD(P)-binding Rossmann-like Domain |
| 259 | **P44129** | No result | No result | No result | TctB family | Cluster – 156295  (yciS protein) | DUF1049 Superfamily | Transmembrane | Cluster4077230  Protein of unknown function DUF1049 |
| 260 | **P44131** | No result | No result | No result | DUF945 family | Cluster – 152146  (GTP-binding protein) | COG5339 Superfamily | EC 5.3.-.-: Isomerases - Intramolecular Oxidoreductases | Cluster3986091  Protein of unknown function DUF945, bacterial |
| 261 | **P44132** | No result | No result | No result | Connexin43 family | Cluster – 152146  (Putative GTP-binding protein) | COG5339 Superfamily | EC 2.7.-.-: Transferases - Transferring Phosphorus-Containing Groups | Cluster3986091  Protein of unknown function DUF945, bacterial |
| 262 | **P45122** | No result | No result | Inner membrane protein family | UPF0126 family | Cluster – 133357  (Transmembrane protein) | UPF0126 superfamily | Transmembrane | Cluster4137444  Uncharacterised protein family UPF0126 |
| 263 | **P44133** | No result | No result | No result | No result | Cluster – 148284  (Sterol O-acyltransferase 1) | No result | Transmembrane | Cluster4154441  Gammaproteobacteria |
| 264 | **P44134** | No result | CAC2185-like family | No result | PrpR_N family | Cluster – 129426  (Capsular polysaccharide biosynthesis protein) | DUF1919 Superfamily | Metal-binding | Cluster4118479  Protein of unknown function DUF1919 |
| 265 | **P44135** | Phosphoglycerol transferase I -like domain family | Arylsulfatase family | Arylsulfatase family | Sulfatase family | Cluster – 151985  (sulphatase) | Sulfatase Superfamily | Transmembrane | Cluster4133052  Membrane sulfatase, HI1246-related |
| 266 | **P44136** | No result | No result | No result | NicO family | Cluster – 127674  (ShiF protein) | NicO Superfamily | Nickel-binding | Cluster4041182  Nickel/cobalt transporter, high-affinity |
| 267 | **P44137** | No result | No result | No result | DUF1007 family | Cluster – 156273  (exported protein) | DUF1007 Superfamily | Zinc-binding | Cluster3835140  Protein of unknown function DUF1007 |
| 268 | **P44138** | No result | RelE-like superfamily | No result | Plasmid_killer family | Cluster – 156274  (Killer protein) | Plasmid_killer Superfamily | Zinc-binding | Cluster4056984  Plasmid maintenance system killer |
| 269 | **P44139** | No result | No result | No result | SirB family | Cluster – 155547  (Protein sirB2) | SirB Superfamily | Transmembrane | Cluster4252806  Invasion gene expression up-regulator, SirB |
| 270 | **P44140** | tRNA(Met) cytidine acetyltransferase TmcA -like domain family/  Mycothiol acetyltransferase -like domain 1/2/3 family | Acyl-CoA N-acyltransferases (Nat) superfamily/  P-loop containing nucleoside triphosphate hydrolases superfamily | N-acetyltransferase 10 family | GNAT_acetyltr_2 family | Cluster – 113588  (Acetyltransferase) | Helicase_RecD Superfamily/tRNA_bind_2 Superfamily | All DNA-binding | Cluster4159674  Protein of unknown function DUF699, ATPase putative |
| 271 | **Same as 270** | - | - | - | - | - | - | - | - |
| 272 | **Same as 270** | - | - | - | - | - | - | - | - |
| 273 | **P44144** | No result | No result | No result | YcaO-like family | Cluster – 156283  (YcaO protein) | YcaO Superfamily | No result | No result |
| 274 | **P44145** | No result | Sigma3 and sigma4 domains of RNA polymerase sigma factors superfamily | No result | Glucos_trans_II family | Cluster – 85251  (4-hydroxybenzoate octaprenyltranferase related protein) | No result | Transmembrane | Cluster4102994  Integral to membrane |
| 275 | **No result** | - | - | - | - | - | - | - | - |
| 276 | **P44148** | No result | No result | No result | No result | Cluster – 141467  (Iron ABC transporter, solute-binding protein) | No result | No result | No result |
| 277 | **P44150** | AT4G25080 protein -like domain ½ family | S-adenosyl-L-methionine-dependent methyltransferases superfamily | Methyltransferase family | Methyltransf_18 family | Cluster –146961  (Methyltransferase) | AdoMet_MTases Superfamily | All DNA-binding | Cluster4080031  Methyltransferase type 11 |
| 278 | **P45138** | Ribosome maturation factor rimP -like domain family | YhbC-like, N-terminal domain family | No result | DUF150 family | Cluster – 149464  (YhbC protein ) | Sm_like Superfamily  (protein associated with RNA) | Iron-binding | Cluster4189503  Uncharacterised protein family UPF0090 |
| 279 | **P44154** | No result | No result | No result | Zn_Tnp_IS1595 | Cluster – 156282  (Zn-ribbon-containing protein) | DUF2310 Superfamily | Zinc-binding | Cluster3771672  Uncharacterised conserved protein UCP029037 |
| 280 | **P44156** | Cysteine desulfuration protein sufE -like domain family | SufE/NifU superfamily | BOLA-like protein-related family | SufE family | Cluster – 142307  (SufE protein) | SufE Superfamily | Nuclear Receptors | Cluster3605417  Fe-S metabolism associated SufE |
| 281 | **P45145** | No result | No result | No result | LrgA family | Cluster – 151186  (Murein hydrolase exporter) | LrgA Superfamily | Transmembrane | Cluster 4142464  LrgA |
| 282 | **P45146** | No result | Indoleamine 2,3-dioxygenase-like family | Putative serotonin transporter family | LrgB-like family | Cluster – 151187  (Negative regulator of murein hydrolase) | LrgB Superfamily | Transmembrane | Cluster4139093  LrgB-like protein |
| 283 | **Q57320** | No result | ABC transporter transmembrane region family | No result | LysE family | Cluster – 152577  (Threonine efflux protein) | LysE Superfamily | Transmembrane | Cluster4184271  Lysine exporter protein (LYSE/YGGA) |
| 284 | **P45154** | NADH-quinone oxidoreductase -like domain family | 2Fe-2S ferredoxin-like family | Family not named | Fer2 family | Cluster – 145548  (Ferredoxin ) | fer2 Superfamily | Iron-binding | Cluster4087288  2Fe-2S ferredoxin-like |
| 285 | **P44158** | No result | No result | No result | Pox_A8 family | Cluster – 119769  (endospore development protein) | No result | Transmembrane | Cluster3259605  Proteobacteria |
| 286 | **P71375** | No result | No result | Sodium/solute symporter family | Sodium:solute symporter family | Cluster – 141147  (Na+/glucose cotransporter) | SLC5-6-like_sbd Superfamily | Transmembrane | Cluster4279952  Sodium/solute symporter |
| 287 | **P44160** | Aldose 1-epimerase family protein -like domain family | Galactose mutarotase-like superfamily | Apospory-associated protein c-related family | Aldose_epim family | Cluster – 112182  (kinase like protein) | Aldose_epim Superfamily | No result | Cluster4158146  Aldose 1-epimerase |
| 288 | **P44161** | No result | No result | No result | MatP family | Cluster –135338  (Dehydrogenase) | MatP Superfamily | Zinc-binding | Cluster3618064  Protein of unknown function DUF1047 |
| 289 | **P44162** | No result | Protein kinase-like (PK-like) superfamily | No result | RIO1 family | Cluster – 147713  (Aminoglycoside phosphotransferase) | PKc_like Superfamily | All lipid-binding proteins | Cluster4186044  Protein kinase-like |
| 290 | **P44163** | No result | No result | No result | LPAM_1 family | Cluster – 19499  (Hypothetical protein) | No result | No result | Cluster662407  Haemophilus influenzae |
| 291 | **P71376** | CRS2-associated factor 1, chloroplastic -like domain ½ family | YhbY-like family | No result | CRS1_YhbY family | Cluster – 149377  (RNA-binding protein) | CRS1_YhbY Superfamily | rRNA-binding Proteins | Cluster4159250  RNA-binding, CRM domain, prokaryote |
| 292 | **P44164** | Serine/threonine-protein phosphatase Pgam5, -like domain family | Phosphoglycerate mutase-like superfamily | No result | His_Phos_1 family | Cluster – 147721  (Phosphohistidine phosphatase sixA) | HP Superfamily | EC 3.1.-.-: Hydrolases - Acting on Ester Bonds | Cluster4158723  Phosphohistidine phosphatase activity |
| 293 | **P71378** | No result | Apolipoprotein A-I superfamily | Late embryogenesis abundant (plants) lea-related family | Lipase_bact_N family | Cluster – 141357  (Late embryogenesis abundant protein 1) | No result | Outer membrane | Cluster |
| 294 | **P44165** | NADPH-dependent 7-cyano-7-deazaguanine reductase -like domain ½ family/  Outer membrane protein oprJ -like domain family | Outer membrane efflux proteins (OEP) family | Outer membrane cation efflux protein family | QueF_N family | Cluster – 147254  (Outer membrane efflux protein) | TFold Superfamily | Outer membrane | Cluster3521305  Outer membrane efflux protein |
| 295 | **P71379** | Cysteine desulfurase -like domain family | Cystathionine synthase-like family | Cysteine desulfurylase family | Aminotran_5 family | Cluster – 139955  (Cysteine desulfurase) | AAT_I Superfamily | EC 4.1.-.-: Lyases - Carbon-Carbon Lyases | Cluster4120088  Cysteine desulfurase |
| 296 | **P45173** | Ferritin -like domain family | Ferritin-like superfamily | Bacterioferritin family | Ferritin family | Cluster – 147577  (bacterioferritin) | Ferritin_like Superfamily | All lipid-binding proteins | Cluster4174904  DNA-binding protein Dps |
| 297 | **P44167** | tRNA (mo5U34)-methyltransferase -like domain family | S-adenosyl-L-methionine-dependent methyltransferases superfamily | Methyltransferase family | Methyltransf_9 family | Cluster – 143210  (Methyltransferase) | AdoMet_MTases Superfamily | Zinc-binding | Cluster3904934  TRNA (mo5U34)-methyltransferase |
| 298 | **P44168** | No result | No result | No result | CxxCxxCCfamily  (Putative zinc- or iron-chelating domain) | Cluster – 156293  (ycgN protein) | FliB Superfamily  (Flagellin N-methylase) | All lipid-binding proteins | Cluster4332397  Uncharacterised conserved protein UCP006173 |
| 299 | **P45180** | Glycogen synthase -like domain ½ family | Oligosaccharide phosphorylase family | Glycogen phosphorylase family | Phosphorylasefamily | Cluster – 147282  (Glycogen phosphorylase 1) | Glycosyltransferase_GTB_type Superfamily | EC 2.4.-.-: Transferases - Glycosyltransferases | Cluster3976983  Glycogen/starch/alpha-glucan phosphorylase |
| 300 | **P45182** | Vitamin B12 transporter BtuB -like domain family | Ligand-gated protein channel family  (Porins superfamily) | No result | Plug family | Cluster – 149214  (Oar protein) | OM_channels Superfamily | No result | Cluster4258564  TonB-dependent receptor, plug |
| 301 | **P44169** | No result | Frataxin-like family | No result | HSDR_N_2family  (Type I restriction enzyme R protein N terminus) | Cluster – 137251  (Adenylate cyclase) | HSDR_N Superfamily | EC 3.1.-.-: Hydrolases - Acting on Ester Bonds | Cluster4106033  Complete proteome |
| 302 | **P44170** | No result | Multidrug resistance efflux transporter EmrE family | No result | EamA-like transporter family | Cluster – 143726  (Permease of the drug/metabolite transporter (DMT) | No result | Transmembrane | Cluster4008610  Protein of unknown function DUF6, transmembrane |
| 303 | **P44171** | No result | No result | No result | Transcription factor DP family | Cluster – 19520  (Hypothetical protein ) | No result | All lipid-binding proteins | Cluster332978  Haemophilus influenzae |
| 304 | **O86237** | Macrophage migration inhibitory factor -like domain family | Tautomerase/MIF superfamily | No result | Tautomerase_2 family | Cluster – 156324  (4-oxalocrotonate tautomerase) | No result | EC 3.1.-.-: Hydrolases - Acting on Ester Bonds | Cluster4016578  Macrophage Migration Inhibitory Factor |
| 305 | **P44172** | Activating signal cointegrator 1 -like domain family | yqfB-like famiy | No result | ASCH family | Cluster – 151905  (Tex protein) | ASCH Superfamily | EC 3.6.-.-: Hydrolases - Acting on Acid Anhydrides | Cluster3775188  Uncharacterised conserved protein UCP029143 |
| 306 | **P44173** | No result | ABC transporter ATPase domain-like family | No result | Zeta_toxin family | Cluster – 145346  (kinase) | COG4185 Superfamily | EC 3.5.-.-: Hydrolases - Acting on Carbon-Nitrogen Bonds, other than Peptide Bonds | Cluster3940252  Bacteria |
| 307 | **P44175** | No result | No result | No result | No result | Cluster – 108913  (Putative peptidoglycan bound protein) | No result | Transmembrane | Cluster4299950  Proteobacteria |
| 308 | **P44176** | Error-prone DNA polymerase -like domain family | PHP domain family | PHP domain protein family | PHP domain family | Cluster – 147364  (Protein trpH) | PHP Superfamily  (polymerase and Histidinol Phosphatase domain) | Zinc-binding | Cluster4132555  Polymerase and histidinol phosphatase, N-terminal |
| 309 | **P44177** | No result | No result | No result | RelB antitoxin family | Cluster – 133463  (Hypothetical protein) | No result | Structural protein (Matrix protein,Core protein,Viral occlusion body,Keratin) | Cluster4027900  Proteobacteria |
| 310 | **P44180** | No result | No result | No result | Ezrin/radixin/moesin family | Cluster – 133464  (Putative bacteriophage protein) | DUF2213 Superfamily | EC 2.7.-.-: Transferases - Transferring Phosphorus-Containing Groups | Cluster4008185  Uncharacterised conserved protein UCP029215 |
| 311 | **P44181** | No result | "Winged helix" DNA-binding domain superfamily | No result | HTH_20family | Cluster – 114345  (Putative ArsR-like transcriptional regulato) | DUF2513 Superfamily | EC 4.2.-.-: Lyases - Carbon-Oxygen Lyases | Cluster4150082  Bacteria |
| 312 | **P44183** | No result | No result | No result | DUF1073 family | Cluster – 128920  (Putative bacteriophage protein) | phge_rel_HI1409 Superfamily | Outer membrane | Cluster3804492  Phage-related protein HI1409 |
| 313 | **P45197** | No result | No result | No result | Phage_pRha family | Cluster – 130171  (Prophage protein ) | hage_pRha Superfamily | EC 2.7.-.-: Transferases - Transferring Phosphorus-Containing Groups | Cluster4242382  Phage regulatory protein, Rha |
| 314 | **P44185** | No result | No result | No result | No result | Cluster – 112330  (Mannosyl transferase) | No result | All lipid-binding proteins | Cluster3948635  Pasteurellaceae |
| 315 | **P44186** | No result | No result | No result | HAMP family | Cluster –95125  (Sec1 protein) | DUF2570 Superfamily | Transmembrane | Cluster3700737  Haemophilus influenzae |
| 316 | **P44187** | Endochitinase -like domain family | Lysozyme-like superfamily | Chitinase-related family | Glyco_hydro_19 family | Cluster – 139238  (prophage chitinase) | lysozyme_like Superfamily | All lipid-binding proteins | Cluster3954912  Glycoside hydrolase, family 19, catalytic |
| 317 | **P44188** | No result | No result | No result | Phage_holin_3 family | Cluster – 152656  (Holin (Lysis protein 13)) | Phage_holin_3 Superfamily | TC 1.E. Channels/Pores - Holins | Cluster3969424  Holin, phage lambda |
| 318 | **P44189** | No result | No result | No result | Bro-N family | Cluster – 146013  (Antirepressor protein) | Bro-N Superfamily/P22_AR_C Superfamily | Transmembrane | Cluster4081061  BRO, N-terminal |
| 319 | **P44190** | No result | No result | No result | Gp49 family | Cluster – 152258  (prophage protein) | Gp49 Superfamily | EC 3.1.-.-: Hydrolases - Acting on Ester Bonds | Cluster4157329  Addiction module killer protein, HI1419 |
| 320 | **P44191** | DNA-binding protein -like domain family | lambda repressor-like DNA-binding domains superfamily | No result | HTH_3 family | Cluster – 152259  (DNA-binding prophage protein) | COG3636 Superfamily | All DNA-binding | Cluster4108294  Addiction module antidote protein, HI1420 |
| 321 | **P44193** | No result | No result | No result | AntA family | Cluster – 24522  (Hypotheticalprotein) | AntA Superfamily/ P22_AR_C Superfamily | Iron-binding | Cluster4129441  AntA/AntB antirepressor |
| 322 | **P44194** | No result | DHS-like NAD/FAD-binding domain superfamily | No result | HTH_23 family | Cluster – 136774  (DNA binding protein) | No result | rRNA-binding Proteins | Cluster4147257  Proteobacteria |
| 323 | **P44196** | No result | No result | No result | Xol-1_N family  (ribosomal protein S5 domain 2-like) | Cluster – 120248  (Putative periplasmic binding protein CbiK precursor) | No result | All lipid-binding proteins | Cluster4123296  Proteobacteria |
| 324 | **P45202** | Prolyl-tRNA synthetase -like domain family | YbaK/ProRS associated domain family | Uncharacterized family | tRNA_edit family | Cluster – 146267  (Transcriptional regulator ybaK) | YbaK_like Superfamily | EC 3.4.-.-: Hydrolases - Acting on peptide bonds (Peptidases) | Cluster3947534  Conserved hypothetical protein CHP00011 |
| 325 | **P56507** | No result | Multidrug efflux transporter AcrB pore domain; PN1, PN2, PC1 and PC2 subdomains family | No result | Rsbr_N family | Cluster – 34950  (Major capsid protein) | UPF0181 Superfamily | All DNA-binding | Cluster4638118  Uncharacterised protein family UPF0181 |
| 326 | **P44197** | No result | Pseudouridine synthase RsuA/RluD family | RNA pseudouridylate synthase family protein | PseudoU_synth_2 family | Cluster – 126634  (Pseudouridylate synthase) | PseudoU_synth Superfamily | No result | Cluster4026323  Pseudouridine synthase, RsuA and RluB/C/D/E/F |
| 327 | **Q57152** | tRNA pseudouridine synthase C -like domain family | YqcC-like family | No result | DUF446 family  (tRNA pseudouridine synthase C) | Cluster – 111794  (yqcC protein) | DUF446 Superfamily | No result | Cluster4058700  Protein of unknown function DUF446 |
| 328 | **P44198** | No result | YcgL-like | No result | YcgL family | Cluster – 92219  (Transcriptional regulator) | YcgL Superfamily | Outer membrane | Cluster4083593  YcgL domain |
| 329 | **P44201** | No result | No result | No result | DUF340 family | Cluster – 147632  (Permease protein of ABC transporter) | DUF340 Superfamily | Transmembrane | Cluster4082786  Protein of unknown function DUF340, prokaryotic membrane |
| 330 | **P44202** | No result | No result | Family not named | DsbD family | Cluster – 140799  (Cytochrome c-type biogenesis protein ccdA) | DsbD Superfamily | Transmembrane | Cluster4063148  Cytochrome c assembly protein, transmembrane region |
| 331 | **P44203** | No result | No result | No result | DUF2846 family | Cluster – 133467  (lipoprotein) | DUF2846 Superfamily | Transmembrane | Cluster4151583  Uncharacterised conserved protein UCP012335 |
| 332 | **P45217** | Outer membrane protein oprJ -like domain family | Outer membrane efflux proteins (OEP) family | Outer membrane cation efflux protein family | OEP family | Cluster – 147254  (Efflux system protein) | No result | All lipid-binding proteins | Cluster3521305  Outer membrane efflux protein |
| 333 | **Same as 293** | - | - | - | - | -- | - | - | - |
| 334 | **Same as 292** | - | - | - | - | - | - | - | - |
| 335 | **P44205** | Maltose/maltodextrin-binding protein -like domain family | Periplasmic binding protein-like II superfamily | Molybdate-binding periplasmic protein family | SBP_bac_11 family | Cluster – 148792  (Molybdate ABC transporter, periplasmic-binding protein) | SBP_bac_1 Superfamily | EC 3.1.-.-: Hydrolases - Acting on Ester Bonds | Cluster3920215  Molybdenum ABC transporter, periplasmic binding protein |
| 336 | **Q57380** | Sulfate transport system permease protein -like domain family | MetI-like family | Molybdenum transport system permease protein MODB family | BPD_transp_1 family | Cluster – 106173  (ABC transporter, permease protein) | TM_PBP2 Superfamily | Transmembrane | Cluster4111433  Binding-protein-dependent transport systems inner membrane component |
| 337 | **P44208** | Transposase -like domain family | Recombinase DNA-binding domain family | No result | HTH_Tnp_Mu_2 family | Cluster – 147020  (Mu-like prophage FluMu transposase A) | HTH_Tnp_Mu_2 Superfamily | RNA-binding Proteins | Cluster4501988  Mu DNA binding, I gamma subdomain |
| 338 | **P44209** | No result | Hypothetical protein HI1480 family | No result | No result | Cluster – 64926  (DNA for 5S rRNA (Fragment)) | No result | All DNA-binding | Cluster651846  1MW5 |
| 339 | **P44210** | No result | No result | No result | No result | Cluster – 136630  (Transmembrane receptor Roundabout1) | No result | Zinc-binding | Cluster4590269  Proteobacteria |
| 340 | **P44212** | No result | MIT domain family | No result | TPR_11 family | Cluster – 19581  (Mu like) | No result | No result | Cluster4002200  Bacteriophage Mu Gam like |
| 341 | **P44213** | No result | No result | No result | FlaC_arch family  (Flagella accessory protein C) | Cluster – 19581  (Mu like) | No result | Nickel-binding | Cluster4372814  Pasteurellales |
| 342 | **P44214** | No result | Bacterial exopeptidase dimerisation domain family | No result | No result | Cluster – 141573  (Membrane associated lipoprotein) | No result | Outer membrane | Cluster4687471  Bacteria |
| 343 | **P44215** | No result | No result | No result | No result | Cluster –136913  (Golgi complex-associated protein) | No result | All lipid-binding proteins | Cluster3746515  Proteobacteria |
| 344 | **P44217** | No result | No result | No result | HMG_box family | Cluster – 143933  (ABC-type oligopeptide transport system, periplasmic component) | No result | All lipid-binding proteins | Cluster3833866  Gammaproteobacteria |
| 345 | **P44218** | No result | N-acetylmuramoyl-L-alanine amidase-like | No result | No result | Cluster – 112438  (N-acetylmuramoyl-L-alanine amidase) | PGRP Superfamily | Metal-binding | Cluster528019  Haemophilus influenzae |
| 346 | **P44219** | No result | No result | No result | DUF2644 family | Cluster – 19593  (Hypothetical protein) | DUF2644 superfamily | TC 1.E. Channels/Pores - Holins | Cluster3941109  Uncharacterised protein family HI1495 |
| 347 | **P44220** | No result | No result | No result | SHR3_chaperone family  (ER membrane protein SH3) | Cluster – 19594  (Hypothetical protein) | DUF2681 Superfamily | EC 3.6.-.-: Hydrolases - Acting on Acid Anhydrides | Cluster3903620  Haemophilus influenzae |
| 348 | **P44221** | No result | Glucocorticoid receptor-like (DNA-binding domain) superfamily | No result | zf-dskA_traR family | Cluster – 151030  (DnaK deletion suppressor protein) | zf-dskA_traR Superfamily | Zinc-binding | Cluster4365291  Zinc finger, C4 DksA/TraR-type |
| 349 | **P44222** | No result | No result | No result | DUF2730 family | Cluster – 126210  (Sensor histidine kinase) | DUF2730 Superfamily | All lipid-binding proteins | Cluster4141003  Gammaproteobacteria |
| 350 | **O86242** | No result | "Winged helix" DNA-binding domain superfamily | No result | HTH_12 family | Cluster – 133279  (DNA-binding domain) | HTH_12 Superfamily | Zinc-binding | Cluster4184246  Proteobacteria |
| 351 | **P44223** | No result | No result | No result | DUF3486 family | Cluster – 133128  (Mu-like prophage FluMu protein gp27) | No result | RNA-binding Proteins | Cluster4084663  Proteobacteria |
| 352 | **P44224** | No result | No result | No result | Terminase_6 family | Cluster – 133130  (Mu-like prophage FluMu protein gp28) | COG4373 Superfamily  (Mu-like prophage FluMu protein gp28) | All lipid-binding proteins | Cluster3788315  Uncharacterised conserved protein UCP007056 |
| 353 | **P44225** | No result | No result | No result | DUF935 family | Cluster – 155792  (Mu-like prophage FluMu protein gp29) | DUF935 Superfamily | Zinc-binding | Cluster4151326  Protein of unknown function DUF935 |
| 354 | **P44226** | No result | No result | No result | Phage_Mu_F family | Cluster – 155625  (Head morphogenesis protein) | Phage_Mu_F Superfamily | All lipid-binding proteins | Cluster4118925  Phage putative head morphogenesis protein, SPP1 gp7 |
| 355 | **P44227** | No result | No result | No result | Mu-like_gpT family | Cluster – 133198  (Major head subunit (gpT)) | Mu-like_gpT Superfamily | EC 2.7.-.-: Transferases - Transferring Phosphorus-Containing Groups | Cluster4053807  Bacteria |
| 356 | **P44228** | No result | YqbF N-terminal domain-like family | No result | HeH family | Cluster – 155801  (Mu-like prophage FluMu protein gp35) | LEM_like Superfamily | Outer membrane | Cluster3947763  2OUT |
| 357 | **Same as 356** | - | - | - | - | - | - | - | - |
| 358 | **P44230** | No result | No result | No result | DUF1320 family | Cluster –155802  (Mu-like prophage FluMu protein gp36) | DUF1320 Superfamily | EC 2.4.-.-: Transferases - Glycosyltransferases | Cluster4141019  Protein of unknown function DUF1320 |
| 359 | **P44231** | No result | No result | No result | DUF1834 family | Cluster – 155803  (Mu-like prophage FluMu protein gp37) | DUF1834 Superfamily | Zinc-binding | Cluster3721770  Protein of unknown function DUF1834 |
| 360 | **P44232** | No result | No result | No result | PTS_IIA family | Cluster – 133134  (Mu-like prophage FluMu protein gp38) | DUF2635 Superfamily | Outer membrane | Cluster4127856  Proteobacteria |
| 361 | **P44234** | No result | No result | No result | Tail_tube family | Cluster – 155921  (Tail tube protein (gpM)) | Tail_tube Superfamily | Zinc-binding | Cluster4097488  Proteobacteria |
| 362 | **P44235** | No result | No result | No result | FluMu_gp41family | Cluster – 155808  (Mu-like prophage FluMu protein gp41) | COG4518 Superfamily | All DNA-binding | Cluster3952333  Gammaproteobacteria |
| 363 | **P44238** | No result | No result | No result | Phage_Mu_Gp45 family | Cluster – 155809  (Bacteriophage (Baseplate assembly) protein) | Phage_Mu_Gp45 Superfamily | Transmembrane | Cluster4098758  Mu-like prophage protein gp45 |
| 364 | **P44239** | No result | No result | No result | GP46 family | Cluster – 155812  (Protein gp46) | GP46 Superfamily | EC 2.7.-.-: Transferases - Transferring Phosphorus-Containing Groups | Cluster4107343  Phage GP46 |
| 365 | **P44240** | No result | No result | No result | Baseplate_J family | Cluster – 155814  (Protein gp47) | Baseplate_J Superfamily | All lipid-binding proteins | Cluster3961356  Baseplate assembly protein J-like, predicted |
| 366 | **P44241** | No result | No result | No result | DUF2313 family | Cluster – 155819  (Mu-like prophage FluMu protein gp48) | DUF2313 Superfamily | All lipid-binding proteins | Cluster3967784  Gammaproteobacteria |
| 367 | **P44242** | No result | Pectin lyase-like superfamily | No result | No result | Cluster – 100889  (Mu-like prophage FluMu defective tail fiber protein) | No result | EC 4.2.-.-: Lyases - Carbon-Oxygen Lyases | Cluster4052667  Pectin lyase fold/virulence factor |
| 368 | **P71390** | No result | No result | No result | Mu-like_Com family | Cluster – 133104  (Cryptic Mu-phage protein Com) | Mu-like_Com Superfamily | No Result | Cluster4755074  Bacteria |
| 369 | **P44243** | eRF1 methyltransferase catalytic subunit MTQ2 -like domain family | S-adenosyl-L-methionine-dependent methyltransferases superfamily | No result | MethyltransfD12 family | Cluster – 133481  (Hypothetical protein ) | No result | EC 3.1.-.-: Hydrolases - Acting on Ester Bonds | Cluster3638648  D12 class N6 adenine-specific DNA methyltransferase |
| 370 | **P44246** | tRNA 5-methylaminomethyl-2-thiouridine -like domain family | FAD/NAD(P)-binding domain superfamily | FAD NAD binding oxidoreductases family | Methyltransf_30 family | Cluster – 150558  (FAD dependent oxidoreductase) | Methyltransf_30 Superfamily/Strep_67kDa_ant Superfamily | EC 1.3.-.-: Oxidoreductases - Acting on the CH-CH group of donors | Cluster4146363  TRNA U-34 5-methylaminomethyl-2-thiouridine biosynthesis protein MnmC, C-terminal |
| 371 | **Same as 370** | - | - | - | - | - | - | - | - |
| 372 | **P44247** | No result | FAD/NAD-linked reductases, N-terminal and central domains | FAD NAD binding oxidoreductases family | DAO family  (FAD dependent oxidoreductase) | Cluster – 150558  (FAD dependent oxidoreductase) | No result | EC 2.4.-.-: Transferases - Glycosyltransferases | Cluster4146363  TRNA U-34 5-methylaminomethyl-2-thiouridine biosynthesis protein MnmC, C-terminal |
| 373 | **P45244** | Oxygen-insensitive NAD(P)H nitroreductase -like domain family | FMN-dependent nitroreductase-like superfamily | NADPH nitroreductase family | Nitroreductase family | Cluster – 143710  (Nitroreductase family protein) | Nitro_FMN_reductase Superfamily | EC 2.3.-.-: Transferases - Acyltransferases | Cluster4157972  Nitroreductase-like |
| 374 | **P44251** | No result | alpha/beta-Hydrolases superfamily | No result | DUF452 family | Cluster – 135245  (Hypothetical protein) | DUF452 Superfamily | All lipid-binding proteins | Cluster3980451  Protein of unknown function DUF452 |
| 375 | **P44252** | No result | No result | Lipoprotein-releasing system transmembrane protein lole family | MacB_PCDfamily | Cluster – 141137  (Lipoprotein releasing system transmembrane protein LOLE) | MacB_PCD Superfamily/FtsX Superfamily | TC 3.A. Primary Active Transporters - P-P-bond-hydrolysis-driven transporters | Cluster4163048  Lipoprotein releasing system, transmembrane protein, LolC/E family |
| 376 | **P45252** | Superkiller protein 3 -like domain family | Tetratricopeptide repeat (TPR) family | Family not named | Transglut_core2family | Cluster – 141615  (TPR domain protein) | Transglut_core2 Superfamily | EC 2.4.-.-: Transferases - Glycosyltransferases | Cluster3880357  Gammaproteo bacteria |
| 377 | **P45253** | Release factor glutamine methyltransferase -like domain family | N5-glutamine methyltransferase, HemK family | HEMK methyltransferase family member | Methyltransf_31 family | Cluster – 145158  (Ribosomal RNA small subunit methyltransferase C (EC 2.1.1.52) | AdoMet_MTases Superfamily | EC 2.1.-.-: Transferases - Transferring One-Carbon Groups | Cluster4151922  Modification methylase HemK |
| 378 | **P44253** | No result | No result | No result | RDD family | Cluster – 123426  (Putative integral membrane protein) | RDD Superfamily | Transmembrane | Cluster3651740  RDD |
| 379 | **P44254** | No result | No result | No result | No result | Cluster – 83894  (Protein HI1562) | No result | All DNA-binding | Cluster4060045  Haemophilus  (Serine Threonine Protein Phosphatase 5, Tetratricopeptide repeat) |
| 380 | **P44255** | No result | No result | No result | DUF462 family | Cluster – 156356  (Putative transporting ATPase) | DUF462 Superfamily | Copper-binding | Cluster4791876  Protein of unknown function DUF462 |
| 381 | **P44256** | DNA polymerase IV -like domain family | Lesion bypass DNA polymerase (Y-family), little finger domain superfamily | No result | UPF0242 family | Cluster – 14066  (no result ) | No result | Nickel-binding | No result |
| 382 | **P44260** | No result | No result | No result | DUF935family | Cluster – 155792  (Mu-like prophage FluMu protein gp29) | DUF935 Superfamily | Metal-binding | Cluster3884590  Protein of unknown function DUF935 |
| 383 | **Q4QKT3** | No result | No result | No result | Band_7_1family | Cluster – 155914  (Replication protein) | No result | rRNA-binding Proteins | Cluster3837166  Replication endonuclease, probable, phage |
| 384 | **P44262** | 4-hydroxyphenylpyruvate dioxygenase -like domain ½ family | Glyoxalase/Bleomycin resistance protein/Dihydroxybiphenyl dioxygenase superfamily | No result | YecMfamily | Cluster – 156340  (Protein yecM) | Glo_EDI_BRP_like Superfamily | No result | Cluster4011886  Protein of unknown function DUF991 |
| 385 | **Same as 384** | - | - | - | - | - | - | - | - |
| 386 | **P45267** | Adenylate cyclase -like domain family | CYTH-like phosphatases superfamily | No result | CYTH family  (CyaB-like adenlyl cyclases) | Cluster – 148636  (ygiF protein) | CYTH-like_Pase Superfamily | All DNA-binding | Cluster2691408  CAMP biosynthetic process |
| 387 | **P44267** | No result | No result | No result | DUF2063 family | Cluster – 156353  (Hypothetical protein) | DUF2063 Superfamily | Zinc-binding | Cluster3451596  Pasteurellales |
| 388 | **P44268** | UPF0276 protein MCA3108 -like domain family | Xylose isomerase-like superfamily | No result | DUF692 family | Cluster – 133486  (Hypothetical protein) | DUF692 Superfamily | Manganese-binding | Cluster41293114  Protein of unknown function DUF692  (Xylose isomerase-like) |
| 389 | **P44269** | No result | No result | No result | Cytomega_UL20A family | Cluster – 148891  (Putative periplasmic protein) | COG3767 Superfamily | Iron-binding | Cluster4103162  Complete proteome |
| 390 | **P44270** | No result | Aquaporin-like family | No result | DoxX family | Cluster – 149079  (Putative integral membrane protein) | DoxX Superfamily | Transmembrane | Cluster3919138  DoxX |
| 391 | **P44272** | No result | No result | No result | SH3_3 family | Cluster – 130233  (SH3 domain protein) | SH3_3 Superfamily | Transmembrane | Cluster4089004  Uncharacterised conserved protein UCP006158, SH3, YgiM |
| 392 | **P44275** | No result | No result | No result | No result | Cluster – 120247  (Putative cytoplasmic membrane protein CbiL precursor) | No result | Transmembrane | Cluster3859326  Integral to membrane |
| 393 | **P44277** | LRP2-binding protein -like domain ½ family | HCP-like superfamily | SEL-1-like protein family | Sel1 family | Cluster – 139872  (Sel-1-like protein) | Sel1 Superfamily | All lipid-binding proteins | Cluster4215175  Keyword 9172  (Serine Threonine Protein Phosphatase 5, Tetratricopeptide repeat) |
| 394 | **P44278** | No result | No result | No result | YwiC family | Cluster – 156361  (Ywic protein) | YwiC Superfamily | Transmembrane | Cluster4118246  Bacteria |
| 395 | **P71394** | RutC family protein yjgF -like domain famiy | YjgF-like superfamily | Translation initiation inhibitor family | Ribonuc_L-PSP family | Cluster – 141938  (Translation initiation inhibitor) | YjgF_YER057c_UK114_family Superfamily | EC 2.5.-.-: Transferases - Transferring Alkyl or Aryl Groups, Other than Methyl Groups | Cluster4054994  Keyword 10030  (Endoribonuclease L-PSP/chorismate mutase-like) |
| 396 | **P45279** | No result | Gam-like family | No result | Phosphate transporter family  (PHO4 family ) | Cluster – 133501  (YhcB protein) | DUF1043 Superfamily | Transmembrane | Cluster4107460  Protein of unknown function DUF1043 |
| 397 | **P45280** | No result | No result | Inner membrane protein deda-related family | SNARE_assoc family | Cluster – 142554  (DedA-family integral membrane protein) | SNARE_assoc Superfamily | Transmembrane | Cluster3971317  SNARE associated Golgi protein |
| 398 | **P44279** | No result | No result | No result | CLPTM1family  (Cleft lip and palate transmembrane protein 1 ) | Cluster – 113288  (DNA helicase) | DUF2726 Superfamily | EC 3.1.-.-: Hydrolases - Acting on Ester Bonds | Cluster3987398  Alphaproteobacteria |
| 399 | **Q57525** | Aspartokinase -like domain family | Carbamate kinase-like superfamily | Aspartate kinase family | AA family  (Amino acid kinase family) | Cluster – 146114  (Aspartokinase (EC 2.7.2.4) (Aspartate kinase)) | AAK Superfamily/ACT Superfamily | EC 2.4.-.-: Transferases - Glycosyltransferases | Cluster3858451  Aspartate kinase activity |
| 400 | **P44280** | No result | P-loop containing nucleoside triphosphate hydrolases superfamily | No result | DUF463 family | Cluster – 156298  (Putative ATPase) | DUF463 Superfamily | Zinc-binding | Cluster4153076  Protein of unknown function DUF463, YcjX-like protein |
| 401 | **P45290** | No result | Ribokinase-like superfamily | Inner membrane protein yeih-related family | Cons_hypoth698 family | Cluster – 156575  (Membrane protein Yeih) | Cons_hypoth698 Superfamily | Transmembrane | Cluster4165087  Uncharacterised protein family UPF0324 |
| 402 | **P44281** | No result | No result | No result | EcoR124_C family | Cluster – 133000  (Class III chitinase) | No result | No result | Cluster4149146  Restriction endonuclease, type I, EcoRI, R subunit |
| 403 | **P44282** | Chemotaxis protein CheA -like domain family | Histidine-containing phosphotransfer domain, HPT domain superfamily | No result | No result | Cluster – 101158  (Sensor histidine kinase-related protein) | HPT Superfamily | Zinc-binding | Cluster4397702  Fungi |
| 404 | **No result** | - | - | - | - | - | - | - | - |
| 405 | **P45298** | Ribosomal RNA small subunit methyltransferase I -like domain family | Tetrapyrrole methylase family | S-adenosylmethionine-dependent methytransferase subfamily | TP_methylase family | Cluster – 144435  (Tetrapyrrole methylase family protein) | TP_methylase Superfamily | EC 2.7.-.-: Transferases - Transferring Phosphorus-Containing Groups | Cluster4162143  Uncharacterised protein family UPF0011  (Cobalt-precorrin-4 Transmethylase; Domain 1) |
| 406 | **P45300** | UPF0102 protein MCA0184 -like domain family | Restriction endonuclease-like superfamily | No result | UPF0102 family | Cluster – 155972  (Endonuclease (EC 3.1.-.-)) | UPF0102 Superfamily | All lipid-binding proteins | Cluster4124296  (Restriction endonuclease-like) |
| 407 | **P52606** | Glucose-6-phosphate isomerase -like domain ½ family | mono-SIS domain family | Sedoheptulose 7-phosphate isomerase / dnaa initiator-associating factor for replication initiation family | SIS_2 family  (Sugar ISomerase) | Cluster – 147050  (Phosphoheptose isomerase (EC 5.-.-.-)) | SIS Superfamily  (Sugar ISomerase) | Manganese-binding | Cluster3887230  Interconverting aldoses and ketoses, and related compounds |
| 408 | **P45301** | No result | No result | No result | BON family | Cluster – 84123  (Homolog of osmotically induced OSMY protein of E.coli) | BON Superfamily | All lipid-binding proteins | Cluster4138967  Transport-associated |
| 409 | **Q57544** | Hydroxyacylglutathione hydrolase -like domain family | Metallo-hydrolase/oxidoreductase superfamily | Beta lactamase domain family | Lactamase_Bfamily | Cluster – 142344  (Metallo-beta-lactamase superfamily protein) | Lactamase_B Superfamily | Iron-binding | Cluster4170543  Beta-lactamase-like |
| 410 | **P45305** | Deoxyribonuclease TatD -like domain family | TatD Mg-dependent DNase-like family | TATD family deoxyribonuclease | TatD_DNasefamily | Cluster – 149137  (Deoxyribonuclease tatD) | metallo-dependent_hydrolases Superfamily | EC 3.1.-.-: Hydrolases - Acting on Ester Bonds | Cluster3957754  Deoxyribonuclease, TatD Mg-dependent |
| 411 | **P44283** | Sonic hedgehog protein -like domain ½ family | No result | No result | Peptidase_M15_2family | Cluster – 147735  (Putative exported protein) | Peptidase_M15_3 Superfamily | No result | Cluster3991562  Protein of unknown function DUF882, bacterial  (Hedgehog/DD-peptidase, zinc-binding motif) |
| 412 | **P44284** | Sonic hedgehog protein -like domain ½ family | Hedgehog/DD-peptidase superfamily | No result | Peptidase_M15_2family | Cluster – 147735  (Putative exported protein) | Peptidase_M15_3 Superfamily | EC 3.1.-.-: Hydrolases - Acting on Ester Bonds | Cluster3991562  Protein of unknown function DUF882, bacterial  (Hedgehog/DD-peptidase, zinc-binding motif) |
| 413 | **P44285** | ErfK/YbiS/YcfS/YnhG family protein -like domain family | L,D-transpeptidase catalytic domain-like family | No result | YkuD family | Cluster – 130284  (Peptidoglycan-binding protein) | YkuD Superfamily | Transmembrane | Cluster2817677  YkuD domain |
| 414 | **P44287** | No result | No result | Uncharacterized family | PqiA family | Cluster – 133477  (Paraquat-inducible protein A) | PqiA Superfamily | Transmembrane | Cluster3861391  Paraquat-inducible protein A  (Multihaem cytochrome) |
| 415 | **P44288** | No result | No result | Uncharacterized family | MCE family | Cluster – 130652  (Paraquat-inducible protein B) | MCE Superfamily | Transmembrane | Cluster3973964  Mammalian cell entry related |
| 416 | **P44289** | No result | MFS general substrate transporter superfamily | P-Hydroxybenzoic acid efflux pump subunit-related family | FUSC-like inner membrane protein yccS family | Cluster – 151054  (Putative efflux (PET) family transporter) | FUSC-like Superfamily/ FUSC_2 Superfamily | TC 9.B. Incompletely Characterized Transport Systems - Putative uncharacterized transport proteins | Cluster4158714  Protein of unknown function DUF893, YccS/YhfK |
| 417 | **P44290** | No result | No result | No result | DUF2057 family | Cluster – 126294  (Exopolygalacturonase precursor) | DUF2057 Superfamily | All lipid-binding proteins | Cluster3464715  Pasteurellales |
| 418 | **P44292** | Putative uncharacterized protein -like domain family | No result | No result | DUF218 family | Cluster – 115958  (Protein ydcF) | YdcF-like Superfamily | Transmembrane | Cluster3970067  Protein of unknown function DUF218 |
| 419 | **P45332** | No result | No result | No result | YjgP_YjgQ family | Cluster – 152692  (YjgP/YjgQ protein) | COG0795 Superfamily | TC 2.A. Electrochemical Potential-driven transporters - Porters (uniporters, symporters, antiporters) | Cluster4271706  Predicted permease YjgP/YjgQ |
| 420 | **P45333** | No result | No result | No result | YjgP_YjgQ family | Cluster – 152692  (YjgP/ YjgQ protein) | COG0795 Superfamily | Transmembrane | Cluster4271706  Predicted permease YjgP/YjgQ |
| 421 | **P44293** | Protein ygiW -like domain family | Hypothetical protein YgiW family | No result | BOF family | Cluster – 156370  (Protein ygiW precursor) | BOF Superfamily | TC 3.A.5 Type II (general) secretory pathway (IISP) family | Cluster4102476  Hypothetical protein YgiW  (OB fold (Dihydrolipoamide Acetyltransferase, E2P)) |
| 422 | **P44294** | No result | No result | No result | HgmA Family  (homogentisate 1,2-dioxygenase) | No result | No result | No result | Cluster4773061  Homogentisate 1,2-dioxygenase |
| 423 | **P44296** | Erythrocyte membrane protein 1, PfEMP1 -like domain | Adhesin YadA, collagen-binding domain family | TSBP protein (fragment)  Subfamily | YadA_head family  (Head domain of trimeric autotransporter adhesin) | Cluster – 108737  (MapA protein (Hsf)) | No result | EC 3.4.-.-: Hydrolases - Acting on peptide bonds (Peptidases) | Cluster295602  Haemophilus influenzae |
| 424 | **Q57066** | Transposase -like domain family | Homeodomain-like superfamily | No result | HTH_28family | Cluster – 142986  (Putative transposase) | HTH_23 Superfamily | All lipid-binding proteins | Cluster4243269  Transposase IS3/IS911 |
| 425 | **P44297** | No result | No result | No result | UPF0231 family | Cluster – 156244  (Pasteurella haemolytica putative coproporphyrinogen III oxidase) | UPF0231 Superfamily | TC 1.C. Channels/Pores - Pore-forming toxins (proteins and peptides) | Cluster4041461  Uncharacterised conserved protein UCP006287 |
| 426 | **O05087** | No result | No result | Manganese transporter family | Nramp family | Cluster – 149528  (Mn2+ and Fe2+ transporter of the NRAMP family) | MntH Superfamily | TC 2.A. Electrochemical Potential-driven transporters - Porters (uniporters, symporters, antiporters) | Cluster2999810  Natural resistance-associated macrophage protein |
| 427 | **P44298** | Urea amidolyase -like domain 1 family | Cyclophilin-like superfamily | Urea amidolyase subfamily | AHS2 family | Cluster – 140465  (Acetyl-CoA carboxylase) | AHS2 Superfamily | EC 3.1.-.-: Hydrolases - Acting on Ester Bonds | Cluster4167582  Allophanate hydrolase subunit 2 |
| 428 | **P44299** | Arginine repressor -like domain family/  Allophanate hydrolase/urea amidolyase-related -like domain family | Cyclophilin-like superfamily | No result | AHS1 family | Cluster – 140465  (Acetyl-CoA carboxylase) | DUR1 Superfamily | EC 4.1.-.-: Lyases - Carbon-Carbon Lyases | Cluster4100812  Conserved hypothetical protein CHP00370 |
| 429 | **P44300** | No result | No result | No result | E1-E2_ATPase family | Cluster – 122532  (ATP synthase subunit I) | No result | EC 3.1.-.-: Hydrolases - Acting on Ester Bonds | Cluster4077986  Bacteria |
