# Supplementary material for: Functional Annotation of Conserved Hypothetical Proteins from Haemophilus influenzae Rd KW20
Source: PLoS One. 2013 Dec 31;8(12):e84263. doi: 10.1371/journal.pone.0084263 (PMC3877243; doi:10.1371/journal.pone.0084263)
Supplement: Table S5 — List of annotated functions of 100 proteins with known function from H. influenzae using BLASTp, SMART, INTERPROSCAN and MOTIF for ROC analysis. (DOCX) [file pone.0084263.s005.docx]

| **S.No**  Table S5: List of annotated function of 100 proteins with known function from *H. influenzae* using BLASTp, SMART, INTERPROSCAN and MOTIF for ROC analysis. | **Accession number** | **UNIPROT ID** | **Protein name** | **BLAST*** | **SMART*** | **INTERPROSCAN*** | **MOTIF*** |
| --- | --- | --- | --- | --- | --- | --- | --- |
|  |  |  |  |  |  | **MOTIF FOUND** | |
|  | [NP_438183.1](http://www.ncbi.nlm.nih.gov/protein/16271985) | P44305 | ribosomal-protein-alanine N-acetyltransferase | ribosomal-protein-alanine N-acetyltransferase  1(5) | ribosomal-n-alpha-acetyltransferase  1(5) | ribosomal-protein-alanine N-acetyltransferase 1(5) | Gcn5-related N-acetyltransferase (GNAT) domain profile 1(5) |
|  | [NP_438185.1](http://www.ncbi.nlm.nih.gov/protein/16271987) | P44453 | 16S ribosomal RNA m2G1207 methyltransferase | 16S ribosomal RNA m2G1207 methyltransferase  1(5) | rRNA (guanine-N2-)-methyltransferase activity  1(4) | Ribosomal RNA small subunit methyltransferase C  1(5) | N-6 Adenine-specific DNA methylases signature  1(4) |
|  | [NP_438192.1](http://www.ncbi.nlm.nih.gov/protein/16271994) | Q57163 | (dimethylallyl)adenosine tRNA methylthiotransferase | (dimethylallyl)adenosine tRNA methylthiotransferase  1(5) | uridine methylases of the TRM2 family  1(3) | Methylthiotransferase/radical SAM-type protein  1(4) | Methylthiotransferase radical SAM domain signature  1(3) |
|  | [NP_438206.1](http://www.ncbi.nlm.nih.gov/protein/16272008) | P44470 | rRNA large subunit methyltransferase | rRNA large subunit methyltransferase  1(5) | Predicted SPOUT methyltransferase  1(3) | Ribosomal RNA large subunit methyltransferase H  1(5) | Coenzyme A transferase 2  1(3) |
|  | [NP_438231.1](http://www.ncbi.nlm.nih.gov/protein/16272032) | P44490 | 3-deoxy-manno-octulosonate cytidylyltransferase | 3-deoxy-manno-octulosonate cytidylyltransferase  1(5) | Cytidylyltransferase  1(4) | 3-deoxy-D-manno-octulosonate cytidylyltransferase  1(5) | Acylneuraminate cytidylyltransferase  1(4) |
|  | [NP_438241.1](http://www.ncbi.nlm.nih.gov/protein/16272042) | P44495 | tRNA delta1(2)-isopentenylpyrophosphate transferase | tRNA delta1(2)-isopentenylpyrophosphate transferase  1(5) | Isopentenyl transferase  1(4) | tRNA delta1(2)-isopentenylpyrophosphate transferase  1(5) | tRNA isopentenyltransferase  1(4) |
|  | [NP_438242.1](http://www.ncbi.nlm.nih.gov/protein/16272043) | P44419 | bifunctional glutamine-synthetase adenylyltransferase/deadenyltransferase | bifunctional glutamine-synthetase adenylyltransferase/deadenyltransferase  1(5) | Glutamate-ammonia ligase adenylyltransferase  1(5) | Glutamate-ammonia-ligase adenylyltransferase  1(5) | Glutamate-ammonia ligase adenylyltransferase  1(5) |
|  | [NP_438246.1](http://www.ncbi.nlm.nih.gov/protein/16272047) | P43933 | nucleotidyltransferase | Nucleotidyltransferase  1(5) | Nucleotidyltransferase domain  1(4) | Nucleotidyl transferase domain  1(4) | GlnD PII-uridylyltransferase  1(3) |
|  | [NP_438247.1](http://www.ncbi.nlm.nih.gov/protein/16272048) | P43934 | nucleotidyltransferase | Nucleotidyltransferase  1(5) | Nucleotidyltransferase substrate binding protein like  1(4) | Nucleotidyltransferase substrate binding protein, HI0074  1(4) | Glycosyl transferase, family 17  0(2) |
|  | [NP_438285.1](http://www.ncbi.nlm.nih.gov/protein/16272085) | P44521 | glutathione transferase | glutathione transferase  1(5) | Glutathione S-transferase  1(5) | Glutathione S-transferase  1(5) | Soluble glutathione S-transferase C-terminal domain profil  1(5) |
|  | [NP_438326.1](http://www.ncbi.nlm.nih.gov/protein/16272123) | P43712 | acyl carrier protein S-malonyltransferase | acyl carrier protein S-malonyltransferase  1(5) | Acyl transferase domain  1(4) | Malonyl CoA-acyl carrier protein transacylase, FabD-type  1(5) | Acyl transferase domain  1(4) |
|  | [NP_438348.1](http://www.ncbi.nlm.nih.gov/protein/16272145) | P43753 | formate acetyltransferase | formate acetyltransferase 1(5) | Pyruvate formate lyase  1(3) | Formate acetyltransferase 1(5) | Pyruvate formate-lyase domain profile  1(3) |
|  | [NP_438368.1](http://www.ncbi.nlm.nih.gov/protein/16272163) | P44567 | lipid A biosynthesis (KDO)2-(lauroyl)-lipid IVA acyltransferase | lipid A biosynthesis (KDO)2-(lauroyl)-lipid IVA acyltransferase  1(5) | Bacterial lipid A biosynthesis acyltransferase  1(4) | Lipid A biosynthesis (KDO)2-(lauroyl)-lipid IVA acyltransferase  1(5) | Bacterial lipid A biosynthesis acyltransferase  1(4) |
|  | [NP_438371.1](http://www.ncbi.nlm.nih.gov/protein/16272165) | P43912 | tRNA (guanine-N(1)-)-methyltransferase | tRNA (guanine-N(1)-)-methyltransferase  1(5) | tRNA (Guanine-1)-methyltransferase  1(5) | tRNA (guanine-N1-)-methyltransferase, bacteria  1(5) | tRNA (guanine-N1-)-methyltransferase  1(5) |
|  | [NP_438414.1](http://www.ncbi.nlm.nih.gov/protein/16272204) | P44594 | queuine tRNA-ribosyltransferase | queuine tRNA-ribosyltransferase  1(5) | Queuine tRNA-ribosyltransferase  1(5) | tRNA-guanine(15) transglycosylase-like  1(5) | Queuine/other tRNA-ribosyltransferase  1(5) |
|  | [NP_438415.1](http://www.ncbi.nlm.nih.gov/protein/16272205) | P44595 | S-adenosylmethionine:tRNA ribosyltransferase-isomerase | S-adenosylmethionine:tRNA ribosyltransferase-isomerase  1(5) | Queuosine biosynthesis protein  1(4) | S-adenosylmethionine:tRNA ribosyltransferase-isomerase  1(5) | Queuosine biosynthesis protein  1(4) |
|  | [NP_438427.1](http://www.ncbi.nlm.nih.gov/protein/16272216) | P43974 | glycosyl transferase | glycosyl transferase  1(5) | Glycosyl transferase family 8  1(5) | Glycosyl transferase, family 8  1(5) | Glycosyl transferase, family 8, C-terminal  1(5) |
|  | [NP_438435.1](http://www.ncbi.nlm.nih.gov/protein/16272224) | P44603 | glycerol-3-phosphate acyltransferase PlsY | glycerol-3-phosphate acyltransferase PlsY  1(5) | Glycerol-3-phosphate acyltransferase  1(5) | Glycerol-3-phosphate acyltransferase, PlsY  1(5) | Oligosaccharyl transferase, STT3 subunit  1(4) |
|  | [NP_438441.1](http://www.ncbi.nlm.nih.gov/protein/16272230) | P43855 | orotate phosphoribosyltransferase | orotate phosphoribosyltransferase 1(5) | Phosphoribosyl transferase domain  1(4) | Orotate phosphoribosyl transferase domain 1(5) | Purine/pyrimidine phosphoribosyl transferases signature.  1(4) |
|  | [NP_438453.1](http://www.ncbi.nlm.nih.gov/protein/16272241) | P71348 | aminotransferase AlaT | aminotransferase AlaT  1(5) | Aminotransferase class I and II  1(4) | Aminotransferase, class I/classII  1(5) | Tyrosine aminotransferase ubiquitination region  1(4) |
|  | [NP_438469.2](http://www.ncbi.nlm.nih.gov/protein/30995366) | P44626 | apolipoprotein N-acyltransferase | apolipoprotein N-acyltransferase  1(5) | Carbon-nitrogen hydrolase  0(2) | Apolipoprotein N-acyltransferase  1(5) | Carbon-nitrogen hydrolase domain profile  0(2) |
|  | [NP_438470.1](http://www.ncbi.nlm.nih.gov/protein/16272258) | P44627 | 16S ribosomal RNA methyltransferase RsmE | 16S ribosomal RNA methyltransferase RsmE  1(5) | RNA methyltransferase  1(4) | Ribosomal RNA small subunit methyltransferase E  1(5) | Protein of unknown function DUF558  0(2) |
|  | [NP_438485.1](http://www.ncbi.nlm.nih.gov/protein/16272273) | P43985 | S-adenosyl-L-methionine-dependent methyltransferase | S-adenosyl-L-methionine-dependent methyltransferase  1(5) | Methyltransferase domain  1(4) | tRNA (cmo5U34)-methyltransferase  1(4) | Glutamate methyltransferase family signature  1(3) |
|  | [NP_438497.1](http://www.ncbi.nlm.nih.gov/protein/16272285) | P44643 | 23S rRNA 5-methyluridine methyltransferase | 23S rRNA 5-methyluridine methyltransferase  1(5) | tRNA (Uracil-5-)-methyltransferase  1(5) | 23S rRNA (uracil(1939)-C(5))-methyltransferase RlmD  1(5) | RNA methyltransferase trmA family signature 2  1(5) |
|  | [NP_438504.1](http://www.ncbi.nlm.nih.gov/protein/16272292) | P44648 | tRNA (guanine-N(7)-)-methyltransferase | tRNA (guanine-N(7)-)-methyltransferase  1(5) | tRNA (guanine-N-7) methyltransferase  1(3) | tRNA (guanine-N-7) methyltransferase  1(5) | Putative methyltransferase  1(3) |
|  | [NP_438526.1](http://www.ncbi.nlm.nih.gov/protein/16272313) | P44665 | ribosomal RNA large subunit methyltransferase N | ribosomal RNA large subunit methyltransferase N  1(5) | Elongator protein 3, MiaB family, Radical SAM  1(3) | Ribosomal RNA large subunit methyltransferase RlmN/Cfr  1(5) | Alpha-class glutathione S-transferase signature  1(3) |
|  | [NP_438564.1](http://www.ncbi.nlm.nih.gov/protein/16272351) | P44687 | methylated-DNA-protein-cysteine methyltransferase | methylated-DNA-protein-cysteine methyltransferase  1(5) | 6-O-methylguanine DNA methyltransferase, ribonuclease-like domain  1(5) | Methylguanine DNA methyltransferase, ribonuclease-like  1(5) | Methylated-DNA--protein-cysteine methyltransferase active site  1(5) |
|  | [NP_438568.1](http://www.ncbi.nlm.nih.gov/protein/16272355) | P43872 | acetyl-CoA carboxylase carboxyltransferase subunit alpha | acetyl-CoA carboxylase carboxyltransferase subunit alpha  1(5) | Acetyl co-enzyme A carboxylase carboxyltransferase alpha subunit  1(5) | Acetyl-coenzyme A carboxyltransferase, C-terminal  1(5) | Acetyl-coenzyme A carboxyltransferase domain C-terminal region profile.  1(5) |
|  | [NP_438590.1](http://www.ncbi.nlm.nih.gov/protein/16272377) | P44708 | glucosamine--fructose-6-phosphate aminotransferase | glucosamine--fructose-6-phosphate aminotransferase  1(5) | Glutamine amidotransferases class-II  1(5) | Glucosamine-fructose-6-phosphate aminotransferase, isomerising  1(5) | Glutamine amidotransferase type 2 domain profile 1(5) |
|  | [NP_438620.1](http://www.ncbi.nlm.nih.gov/protein/16272407) | P44722 | bifunctional pyrimidine regulatory protein PyrR uracil phosphoribosyltransferase | bifunctional pyrimidine regulatory protein PyrR uracil phosphoribosyltransferase  1(5) | Phosphoribosyl transferase domain  1(4) | Bifunctional protein PyrR  1(5) | Purine/pyrimidine phosphoribosyl transferase 1(4) |
|  | [NP_438629.1](http://www.ncbi.nlm.nih.gov/protein/16272416) | P43853 | ATP phosphoribosyltransferase | ATP phosphoribosyltransferase  1(5) | ATP phosphoribosyltransferase  1(5) | ATP phosphoribosyltransferase HisG  1(5) | ATP phosphoribosyltransferase signature  1(5) |
|  | [NP_438631.1](http://www.ncbi.nlm.nih.gov/protein/16272418) | P44423 | histidinol-phosphate aminotransferase | histidinol-phosphate aminotransferase  1(5) | Aminotransferase class I and II  1(4) | Aminotransferase, class I/classII  1(5) | Aminotransferases class-II pyridoxal-phosphate attachment site  1(4) |
|  | [NP_438647.1](http://www.ncbi.nlm.nih.gov/protein/16272434) | P44728 | 16S rRNA methyltransferase GidB | 16S rRNA methyltransferase GidB  1(5) | rRNA small subunit methyltransferase G  1(5) | rRNA small subunit methyltransferase G  1(5) | Glucose inhibited division protein  1(4) |
|  | [NP_438653.1](http://www.ncbi.nlm.nih.gov/protein/16273634) | P44009 | acid phosphatase/phosphotransferase | acid phosphatase/phosphotransferase  1(5) | HAD superfamily, subfamily IIIB (Acid phosphatase) 1(4) | HAD-superfamily phosphatase, subfamily IIIB, AphA  1(4) | Acid phosphatase (Class B)  1(3) |
|  | [NP_438667.2](http://www.ncbi.nlm.nih.gov/protein/30995381) | P44739 | 1,4-dihydroxy-2-naphthoate octaprenyltransferase | 1,4-dihydroxy-2-naphthoate prenyltransferase  1(5) | UbiA prenyltransferase family  1(4) | 1,4-dihydroxy-2-naphthoate octaprenyltransferase  1(5) | UbiA prenyltransferase  1(4) |
|  | [NP_438681.2](http://www.ncbi.nlm.nih.gov/protein/30995382) | P44011 | heptosyltransferase-like protein | Hypothetical protein /  heptosyltransferase-like protein  1(3) | Glycosyltransferase family 9 (heptosyltransferase)  1(5) | Glycosyl transferase, family 9  1(4) | Glycosyl transferase, family 9  1(5) |
|  | [NP_438707.1](http://www.ncbi.nlm.nih.gov/protein/16272493) | **P44749** | dimethyladenosine transferase | dimethyladenosine transferase  1(5) | Ribosomal RNA adenine dimethylases  1(4) | Ribosomal RNA adenine methylase transferase  1(5) | Ribosomal RNA adenine dimethylases signature  1(4) |
|  | [NP_438753.1](http://www.ncbi.nlm.nih.gov/protein/16272539) | **P44770** | ornithine carbamoyltransferase | ornithine carbamoyltransferase  1(5) | Aspartate/ornithine carbamoyltransferase, carbamoyl-P binding domain  1(5) | Aspartate/ornithine carbamoyltransferase  1(5) | Ornithine carbamoyltransferase signature  1(5) |
|  | [NP_438764.1](http://www.ncbi.nlm.nih.gov/protein/16272548) | **P43886** | serine acetyltransferase | serine acetyltransferase  1(5) | Serine acetyltransferase, N-terminal  1(5) | Serine O-acetyltransferase  1(5) | Serine acetyltransferase, N-terminal  1(5) |
|  | [NP_438783.1](http://www.ncbi.nlm.nih.gov/protein/16272566) | **P44787** | methionyl-tRNA formyltransferase | methionyl-tRNA formyltransferase  1(5) | Methionyl-tRNA formyltransferase  1(4) | Methionyl-tRNA formyltransferase  1(5) | Phosphoribosylglycinamide formyltransferase active site  1(4) |
|  | [NP_438802.1](http://www.ncbi.nlm.nih.gov/protein/16272585) | **P43889** | bifunctional N-acetylglucosamine-1-phosphate uridyltransferase/glucosamine-1-phosphate acetyltransfe... | bifunctional N-acetylglucosamine-1-phosphate uridyltransferase/glucosamine-1-phosphate acetyltransferase  1(5) | Nucleotidyl transferase  1(3) | Bifunctional UDP-N-acetylglucosamine pyrophosphorylase/glucosamine-1-phosphate N-acetyltransferase  1(5) | Hexapeptide-repeat containing-transferases signature  1(3) |
|  | [NP_438811.1](http://www.ncbi.nlm.nih.gov/protein/16272594) | **P44805** | phosphopantetheine adenylyltransferase | phosphopantetheine adenylyltransferase  1(5) | Cytidylyltransferase  1(4) | Cytidyltransferase-like domain  1(4) | Cytidylyltransferase  1(4) |
|  | [NP_438812.1](http://www.ncbi.nlm.nih.gov/protein/16272595) | **P44806** | 3-deoxy-D-manno-octulosonic-acid transferase | 3-deoxy-D-manno-octulosonic-acid transferase  1(5) | 3-Deoxy-D-manno-octulosonic-acid transferase (kdotransferase)1(5) | 3-deoxy-D-manno-octulosonic-acid transferase, N-terminal  1(5) | Three-deoxy-D-manno-octulosonic-acid transferase,  1(5) |
|  | [NP_438832.1](http://www.ncbi.nlm.nih.gov/protein/16272614) | **O05029** | 2-C-methyl-D-erythritol 4-phosphate cytidylyltransferase | 2-C-methyl-D-erythritol 4-phosphate cytidylyltransferase  1(5) | 4-diphosphocytidyl-2C-methyl-D-erythritol synthase  1(5) | 2-C-methyl-D-erythritol 4-phosphate cytidylyltransferase  1(5) | 4-diphosphocytidyl-2C-methyl-D-erythritol synthase signature  1(5) |
|  | [NP_438834.1](http://www.ncbi.nlm.nih.gov/protein/16272616) | **P43859** | xanthine-guanine phosphoribosyltransferase | xanthine-guanine phosphoribosyltransferase  1(5) | Phosphoribosyl transferase domain  1(4) | Xanthine-guanine phosphoribosyltransferase  1(5) | Purine/pyrimidine phosphoribosyl transferases signature.  1(4) |
|  | [NP_438839.1](http://www.ncbi.nlm.nih.gov/protein/16272621) | **P44819** | thiosulfate sulfurtransferase GlpE | thiosulfate sulfurtransferase GlpE  1(5) | Rhodanese Homology Domain  1(4) | Thiosulfate sulfurtransferase, bacterial  1(5) | Rhodanese domain profile  1(4) |
|  | [NP_438852.1](http://www.ncbi.nlm.nih.gov/protein/16272634) | **P43859** | xanthine-guanine phosphoribosyltransferase | xanthine-guanine phosphoribosyltransferase  1(5) | Phosphoribosyl transferase domain  1(4) | Xanthine-guanine phosphoribosyltransferase  1(5) | Purine/pyrimidine phosphoribosyl transferases signature  1(4) |
|  | [NP_438893.1](http://www.ncbi.nlm.nih.gov/protein/16272675) | **P44848** | 1-acylglycerol-3-phosphate O-acyltransferase | 1-acylglycerol-3-phosphate O-acyltransferase  1(5) | Phosphate acyltransferases  1(4) | 1-acyl-sn-glycerol-3-phosphate acyltransferase  1(5) | Phospholipid/glycerol acyltransferase  1(4) |
|  | [NP_438907.1](http://www.ncbi.nlm.nih.gov/protein/16272689) | **P44857** | glycerol-3-phosphate acyltransferase | glycerol-3-phosphate acyltransferase  1(5) | Phosphate acyltransferases  1(4) | Glycerol-3-phosphate O-acyltransferase/Dihydroxyacetone phosphate acyltransferase  1(5) | Phospholipid/glycerol acyltransferase  1(4) |
|  | [NP_438922.1](http://www.ncbi.nlm.nih.gov/protein/16272704) | P44308 | nicotinamide-nucleotide adenylyltransferase | nicotinamide-nucleotide adenylyltransferase  1(5) | Nucleotidylyl transferase  1(3) | Nicotinamide-nucleotide adenylyltransferase  1(5) | Aldehyde dehydrogenases glutamic acid active site.  0(2) |
|  | [NP_438925.1](http://www.ncbi.nlm.nih.gov/protein/16272707) | P44868 | RNA methyltransferase | RNA methyltransferase  1(5) | SpoU rRNA Methylase family  1(5) | tRNA (cytidine/uridine-2'-O-)-methyltransferase  1(5) | tRNA/rRNA methyltransferase (SpoU)  1(5) |
|  | [NP_438930.1](http://www.ncbi.nlm.nih.gov/protein/16272712) | P44873 | acetyl-CoA acetyltransferase | acetyl-CoA acetyltransferase  1(5) | Thiolase, N-terminal domain  1(5) | acetyl-CoA C-acetyltransferase  1(5) | Thiolases acyl-enzyme intermediate signature  1(5) |
|  | [NP_438932.1](http://www.ncbi.nlm.nih.gov/protein/16272714) | P44874 | acetate CoA-transferase subunit beta | acetate CoA-transferase subunit beta  1(5) | Coenzyme A transferase  1(5) | Coenzyme A transferase family I  1(5) | Coenzyme A transferases signature 2  1(5) |
|  | [NP_438933.1](http://www.ncbi.nlm.nih.gov/protein/16272715) | P44875 | acetyl-CoA:acetoacetyl-CoA transferase subunit alpha | acetyl-CoA:acetoacetyl-CoA transferase subunit alpha  1(5) | Coenzyme A transferase  1(5) | Coenzyme A transferase family I  1(5) | Coenzyme A transferases signature 1  1(5) |
|  | [NP_438972.1](http://www.ncbi.nlm.nih.gov/protein/16272753) | P44878 | glucosephosphate uridylyltransferase | glucosephosphate uridylyltransferase  1(5) | Nucleotidyl transferase  1(4) | UTP--glucose-1-phosphate uridylyltransferase, bacterial/archaeal-type  1(5) | Nucleotidyl transferase  1(4) |
|  | [NP_438980.1](http://www.ncbi.nlm.nih.gov/protein/16272761) | P31764 | galactose-1-phosphate uridylyltransferase | galactose-1-phosphate uridylyltransferase  1(5) | Galactose-1-phosphate uridyl transferase, N-terminal domain  1(5) | Galactose-1-phosphate uridyl transferase, class I  1(5) | Galactose-1-phosphate uridyl transferase family 1 active site signature  1(5) |
|  | [NP_439008.1](http://www.ncbi.nlm.nih.gov/protein/16272789) | P31812 | tRNA (uracil-5-)-methyltransferase | tRNA (uracil-5-)-methyltransferase  1(5) | tRNA (Uracil-5-)-methyltransferase  1(5) | tRNA (uracil(54)-C(5))-methyltransferase,TrmA, proteobacteria  1(5) | tRNA (uracil-5-)-methyltransferase/TrmA  1(5) |
|  | [NP_439009.1](http://www.ncbi.nlm.nih.gov/protein/16273637) | P44901 | SAM-dependent methyltransferase | SAM-dependent methyltransferase  1(5) | S-adenosyl-L-methionine-dependent methyltransferases  1(4) | Ribosomal RNA small subunit methyltransferase J  1(5) | S-adenosyl-L-methionine-dependent methyltransferases  1(4) |
|  | [NP_439020.1](http://www.ncbi.nlm.nih.gov/protein/16272800) | P44906 | 23S rRNA (guanosine-2\'-O-)-methyltransferase | 23S rRNA (guanosine-2'-O-)-methyltransferase  1(5) | RNA 2'-O ribose methyltransferase substrate binding  1(5) | 23S rRNA (guanosine-2-O-)-methyltransferase RlmB  1(5) | tRNA/rRNA methyltransferase (SpoU)  1(5) |
|  | [NP_439033.1](http://www.ncbi.nlm.nih.gov/protein/16272813) | Q57491 | undecaprenyl-phosphate galactosephosphotransferase | undecaprenyl-phosphate galactosephosphotransferase  1(5) | Bacterial sugar transferase  1(3) | Undecaprenyl-phosphate galactose phosphotransferase, WbaP  1(5) | Bacterial sugar transferase  1(3) |
|  | [NP_439048.1](http://www.ncbi.nlm.nih.gov/protein/16272827) | P43852 | bifunctional phosphoribosylaminoimidazolecarboxamide formyltransferase/IMP cyclohydrolase | bifunctional phosphoribosylaminoimidazolecarboxamide formyltransferase/IMP cyclohydrolase  1(5) | AICARFT/IMPCHase bienzyme  1(5) | AICARFT/IMPCHase bienzyme  1(5) | AICARFT/IMPCHase bienzyme  1(5) |
|  | [NP_439050.1](http://www.ncbi.nlm.nih.gov/protein/16272829) | P43844 | serine hydroxymethyltransferase | serine hydroxymethyltransferase  1(5) | Serine hydroxymethyltransferase  1(5) | Serine hydroxymethyltransferase  1(5) | Serine hydroxymethyltransferase pyridoxal-phosphate attachment site  1(5) |
|  | [NP_439064.1](http://www.ncbi.nlm.nih.gov/protein/16272841) | P44930 | prolipoprotein diacylglyceryl transferase | prolipoprotein diacylglyceryl transferase  1(5) | Prolipoprotein diacylglyceryl transferase  1(5) | Prolipoprotein diacylglyceryl transferase  1(5) | Prolipoprotein diacylglyceryl transferase  1(5) |
|  | [NP_439075.1](http://www.ncbi.nlm.nih.gov/protein/16272852) | P43888 | UDP-3-O-[3-hydroxymyristoyl] glucosamine N-acyltransferase | UDP-3-O-[3-hydroxymyristoyl] glucosamine N-acyltransferase  1(5) | UDP-3-O-[3-hydroxymyristoyl] glucosamine N-acyltransferase, LpxD  1(5) | UDP-3-O-[3-hydroxymyristoyl] glucosamine N-acyltransferase, LpxD  1(5) | UDP-3-O-[3-hydroxymyristoyl] glucosamine N-acyltransferase, LpxD  1(5) |
|  | [NP_439110.1](http://www.ncbi.nlm.nih.gov/protein/16272887) | P44951 | diaminobutyrate--2-oxoglutarate aminotransferase | diaminobutyrate--2-oxoglutarate aminotransferase  1(5) | Aminotransferase class-III  1(4) | 2,4-diaminobutyrate 4-transaminase  1(5) | Aminotransferases class-III pyridoxal-phosphate attachment site  1(4) |
|  | [NP_439119.1](http://www.ncbi.nlm.nih.gov/protein/16272896) | P44083 | 23S rRNA methyluridine methyltransferase | 23S rRNA methyluridine methyltransferase  1(5) | tRNA (Uracil-5-)-methyltransferase  1(5) | 23S rRNA (uracil(747)-C(5))-methyltransferase RlmC  1(5) | tRNA (uracil-5-)-methyltransferase/TrmA  1(5) |
|  | [NP_439124.1](http://www.ncbi.nlm.nih.gov/protein/16272900) | P44957 | bifunctional riboflavin kinase/FMN adenylyltransferase | bifunctional riboflavin kinase/FMN adenylyltransferase  1(5) | Riboflavin kinase  1(5) | Riboflavin kinase, bacterial  1(5) | Riboflavin kinase / FAD synthetase  1(5) |
|  | [NP_439141.1](http://www.ncbi.nlm.nih.gov/protein/16272916) | P44402 | ribosomal protein L11 methyltransferase | ribosomal protein L11 methyltransferase  1(5) | Ribosomal protein L11 methyltransferase (PrmA)  1(5) | Ribosomal protein L11 methyltransferase  1(5) | Ribosomal L11 methyltransferase  1(5) |
|  | [NP_439215.1](http://www.ncbi.nlm.nih.gov/protein/16272988) | P71366 | type III restriction-modification system methyltransferase-like protein | type III restriction-modification system methyltransferase-like protein  1(5) | DNA methylase  1(4) | N6 adenine-specific DNA methyltransferase, D21 class  1(4) | D21 class N6 adenine-specific DNA methyltransferase  1(4) |
|  | [NP_439219.1](http://www.ncbi.nlm.nih.gov/protein/16272992) | P43887 | UDP-N-acetylglucosamine acyltransferase | UDP-N-acetylglucosamine acyltransferase  1(5) | Bacterial transferase hexapeptide (six repeats)  1(5) | Acyl-[acyl-carrier-protein]--UDP-N-acetylglucosamine O-acyltransferase 1(5) | UDP-N-acetylglucosamine acyltransferase  1(5) |
|  | [NP_439238.1](http://www.ncbi.nlm.nih.gov/protein/16273009) | P45025 | UDP-N-acetylglucosamine 1-carboxyvinyltransferase | UDP-N-acetylglucosamine 1-carboxyvinyltransferase  1(5) | EPSP synthase (3-phosphoshikimate 1-carboxyvinyltransferase)  1(5) | UDP-N-acetylglucosamine 1-carboxyvinyltransferase  1(5) | EPSP synthase (3-phosphoshikimate 1-carboxyvinyltransferase)  1(5) |
|  | [NP_439262.1](http://www.ncbi.nlm.nih.gov/protein/16273031) | P45042 | ADP-heptose-LPS heptosyltransferase II | ADP-heptose-LPS heptosyltransferase II  1(5) | Glycosyltransferase family 9 (heptosyltransferase)  1(5) | Lipopolysaccharide heptosyltransferase II  1(5) | Glycosyl transferase, family 9  1(5) |
|  | [NP_439288.1](http://www.ncbi.nlm.nih.gov/protein/16273056) | P45057 | S-adenosyl-methyltransferase MraW | S-adenosyl-methyltransferase MraW  1(5) | MraW methylase family  1(5) | Ribosomal RNA small subunit methyltransferase H  1(5) | Bacterial methyltransferase  1(5) |
|  | [NP_439293.1](http://www.ncbi.nlm.nih.gov/protein/16273061) | P45062 | phospho-N-acetylmuramoyl-pentapeptide-transferase | phospho-N-acetylmuramoyl-pentapeptide-transferase  1(5) | Phospho-N-acetylmuramoyl-pentapeptide-transferase signature 1  1(5) | Phospho-N-acetylmuramoyl-pentapeptide transferase  1(5) | Phospho-N-acetylmuramoyl-pentapeptide transferase  1(5) |
|  | [NP_439296.1](http://www.ncbi.nlm.nih.gov/protein/16273064) | P45065 | undecaprenyldiphospho-muramoylpentapeptide beta-N- acetylglucosaminyltransferase | undecaprenyldiphospho-muramoylpentapeptide beta-N- acetylglucosaminyltransferase  1(5) | Glycosyltransferase family 28 N-terminal domain  1(4) | N-acetylglucosaminyltransferase, MurG  1(5) | Glycosyl transferase, family 28  1(4) |
|  | [NP_439311.1](http://www.ncbi.nlm.nih.gov/protein/16273078) | P45078 | hypoxanthine-guanine phosphoribosyltransferase | hypoxanthine-guanine phosphoribosyltransferase  1(5) | Phosphoribosyl transferase domain  1(5) | Hypoxanthine phosphoribosyl transferase  1(5) | Purine/pyrimidine phosphoribosyl transferases signature  1(5) |
|  | [NP_439324.1](http://www.ncbi.nlm.nih.gov/protein/16273090) | Q57004 | histidinol-phosphate aminotransferase | histidinol-phosphate aminotransferase  1(5) | Aminotransferase class I and II  1(4) | Histidinol-phosphate aminotransferase family  1(5) | Aminotransferases class-I pyridoxal-phosphate attachment site  1(4) |
|  | [NP_439325.1](http://www.ncbi.nlm.nih.gov/protein/16273091) | P44336 | phosphoserine aminotransferase | phosphoserine aminotransferase  1(5) | Aminotransferase class-V  1(5) | Phosphoserine aminotransferase  1(5) | Phosphoserine aminotransferase  1(5) |
|  | [NP_439349.1](http://www.ncbi.nlm.nih.gov/protein/16273115) | P54689 | branched-chain amino acid aminotransferase | branched-chain amino acid aminotransferase  1(5) | Aminotransferase class IV  1(5) | Branched-chain amino acid aminotransferase II  1(5) | Aminotransferase class IV  1(5) |
|  | [NP_439351.1](http://www.ncbi.nlm.nih.gov/protein/16273647) | P45100 | RNA 2\'-O-ribose methyltransferase | RNA 2'-O-ribose methyltransferase  1(5) | FtsJ-like methyltransferase  1(4) | Ribosomal RNA large subunit methyltransferase M  1(5) | Cell division protein FtsJ  1(4) |
|  | [NP_439357.1](http://www.ncbi.nlm.nih.gov/protein/16273121) | P45106 | N5-glutamine S-adenosyl-L-methionine-dependent methyltransferase | N5-glutamine S-adenosyl-L-methionine-dependent methyltransferase  1(5) | Ribosomal RNA large subunit methyltransferase F  1(4) | Ribosomal protein L3-specific, glutamine-N5-methyltransferase  1(5) | N-6 Adenine-specific DNA methylases signature.  1(4) |
|  | [NP_439359.1](http://www.ncbi.nlm.nih.gov/protein/16273123) | P45107 | phosphate acetyltransferase | phosphate acetyltransferase 1(5) | Phosphate acetyl/butaryl transferase  1(5) | Phosphate acetyltransferase, bacteria  1(5) | Phosphate acetyl/butaryl transferase  1(5) |
|  | [NP_439363.1](http://www.ncbi.nlm.nih.gov/protein/16273127) | P43854 | amidophosphoribosyltransferase | Amidophosphoribosyltransferase  1(5) | Phosphoribosyl transferase domain  1(5) | Amidophosphoribosyl transferase  1(5) | Glutamine amidotransferase, class-II  1(5) |
|  | [NP_439384.1](http://www.ncbi.nlm.nih.gov/protein/16273147) | P43857 | uracil phosphoribosyltransferase | uracil phosphoribosyltransferase  1(5) | Phosphoribosyl transferase domain  1(5) | Uracil phosphoribosyl transferase  1(5) | Purine/pyrimidine phosphoribosyl transferase  1(4) |
|  | [NP_439386.1](http://www.ncbi.nlm.nih.gov/protein/16273149) | P43856 | adenine phosphoribosyltransferase | adenine phosphoribosyltransferase  1(5) | Phosphoribosyl transferase domain  1(5) | Adenine phosphoribosyl transferase  1(5) | Purine/pyrimidine phosphoribosyl transferases signature  1(5) |
|  | [NP_439388.1](http://www.ncbi.nlm.nih.gov/protein/16273151) | **P45118** | dihydrolipoamide acetyltransferase | dihydrolipoamide acetyltransferase  1(5) | 2-oxoacid dehydrogenases acyltransferase (catalytic domain)  1(5) | Dihydrolipoamide acetyltransferase pyruvate dehydrogenase complex  1(5) | 2-oxo acid dehydrogenases acyltransferase component lipoyl binding site  1(5) |
|  | [NP_439418.1](http://www.ncbi.nlm.nih.gov/protein/16273181) | **P45131** | homoserine O-acetyltransferase | homoserine O-acetyltransferase  1(5) | alpha/beta hydrolase fold  1(3) | Homoserine acetyltransferase  1(5) | Homoserine acetyltransferase  1(4) |
|  | [NP_439432.1](http://www.ncbi.nlm.nih.gov/protein/16273194) | **Q57140** | acylneuraminate cytidylyltransferase | acylneuraminate cytidylyltransferase  1(5) | CMP-N-acetylneuraminic acid synthetase  1(5) | Acylneuraminate cytidylyltransferase  1(5) | Acylneuraminate cytidylyltransferase  1(5) |
|  | [NP_439485.1](http://www.ncbi.nlm.nih.gov/protein/16273244) | **P45162** | 23S rRNA methyltransferase J | 23S rRNA methyltransferase J  1(5) | FtsJ-like methyltransferase  1(5) | Ribosomal RNA large subunit methyltransferase E  1(5) | Cell division protein FtsJ  1(5) |
|  | [NP_439507.1](http://www.ncbi.nlm.nih.gov/protein/16273266) | **P45176** | 4-alpha-glucanotransferase | 4-alpha-glucanotransferase  1(5) | 4-alpha-glucanotransferase  1(5) | Glycoside hydrolase, catalytic domain  1(5) | Glycoside hydrolase, family 77  1(5) |
|  | [NP_439510.1](http://www.ncbi.nlm.nih.gov/protein/16273269) | **P43796** | glucose-1-phosphate adenylyltransferase | glucose-1-phosphate adenylyltransferase  1(5) | Nucleotidyl transferase  1(4) | Glucose-1-phosphate adenylyltransferase  1(5) | ADP-glucose pyrophosphorylase  1(4) |
|  | [NP_439542.1](http://www.ncbi.nlm.nih.gov/protein/16273299) | **P43858** | anthranilate phosphoribosyltransferase | anthranilate phosphoribosyltransferase  1(5) | Glycosyl transferase family, a/b domain  1(5) | Anthranilate phosphoribosyl transferase  1(5) | Thymidine/pyrimidine-nucleoside phosphorylase  1(4) |
|  | [NP_439577.1](http://www.ncbi.nlm.nih.gov/protein/16273333) | **P43846** | phosphoribosylglycinamide formyltransferase | phosphoribosylglycinamide formyltransferase  1(5) | Formyl transferase  1(4) | Phosphoribosylglycinamide formyltransferase  1(5) | Phosphoribosylglycinamide formyltransferase active site  1(5) |
|  | [NP_439590.1](http://www.ncbi.nlm.nih.gov/protein/16273345) | **P45204** | geranyltranstransferase | Geranyltranstransferase  1(5) | Polyprenyl synthetase  1(5) | Polyprenyl synthetase  1(5) | Polyprenyl synthetase  1(5) |
|  | [NP_439675.1](http://www.ncbi.nlm.nih.gov/protein/16273426) | **O05074** | bifunctional heptose 7-phosphate kinase/heptose 1-phosphate adenyltransferase | bifunctional heptose 7-phosphate kinase/heptose 1-phosphate adenyltransferase  1(5) | Phosphomethylpyrimidine kinase  1(5) | RfaE bifunctional protein, domain I  1(5) | Cytidylyltransferase  1(4) |
|  | [NP_439676.2](http://www.ncbi.nlm.nih.gov/protein/30995456) | **P45239** | lipid A biosynthesis lauroyl acyltransferase | lipid A biosynthesis lauroyl acyltransferase (htrB)  1(5) | Bacterial lipid A biosynthesis acyltransferase  1(5) | Lipid A biosynthesis lauroyl (or palmitoleoyl) acyltransferase  1(5) | Bacterial lipid A biosynthesis acyltransferase  1(5) |
|  | [NP_439724.1](http://www.ncbi.nlm.nih.gov/protein/16273473) | **Q57287** | glycosyl transferase | glycosyl transferase  1(5) | Glycosyl transferase family 2  1(5) | Glycosyl transferase, family 2  1(5) | Glycosyl transferase, family 2  1(5) |
|  | [NP_439734.1](http://www.ncbi.nlm.nih.gov/protein/16273483) | **Q03421** | 3-phosphoshikimate 1-carboxyvinyltransferase | 3-phosphoshikimate 1-carboxyvinyltransferase  1(5) | EPSP synthase (3-phosphoshikimate 1-carboxyvinyltransferase)  1(5) | 3-phosphoshikimate 1-carboxyvinyltransferase  1(5) | EPSP synthase (3-phosphoshikimate 1-carboxyvinyltransferase)  1(5) |
|  | [NP_439748.1](http://www.ncbi.nlm.nih.gov/protein/16273496) | **P45269** | Multifunctional tRNA nucleotidyl transferase/2\'3\'-cyclic phosphodiesterase/2\'nucleotidase/phospha... | multifunctional tRNA nucleotidyl transferase/2'3'-cyclic phosphodiesterase/2'nucleotidase/phosphatase  1(5) | Poly A polymerase head domain  1(5) | tRNA nucleotidyltransferase, proteobacteria  1(5) | Polynucleotide adenylyltransferase  1(5) |
|  | [NP_439759.1](http://www.ncbi.nlm.nih.gov/protein/16273507) | **P44425** | aromatic amino acid aminotransferase | aromatic amino acid aminotransferase  1(5) | Aminotransferase class I and II  1(5) | minotransferase, class I/classII  1(5) | Aminotransferases class-I pyridoxal-phosphate attachment site  1(5) |

***True positive and true negative are denoted by “1” and “0”**

**Integers in () denotes the confidence level**
