# Supplementary material for: Functional Annotation of Conserved Hypothetical Proteins from Haemophilus influenzae Rd KW20
Source: PLoS One. 2013 Dec 31;8(12):e84263. doi: 10.1371/journal.pone.0084263 (PMC3877243; doi:10.1371/journal.pone.0084263)
Supplement: Table S6 — List of functionally annotated domains of 100 proteins with known function from H. influenzae by CATH, SUPERFAMILY, PANTHER, Pfam, SYSTERS, CDART SVMProt and ProtoNet for ROC analysis. (DOCX) [file pone.0084263.s006.docx]

| **S.NO**  **Table S6:** List of functionally annotated domain of 100 proteins with known function from *H. influenzae* by CATH, SUPERFAMILY, PANTHER, Pfam, SYSTERS, CDART SVMProt and ProtoNet for ROC analysis. | **UNIPROT ID** | **SUPERFAMILY*** | **CATH*** | **PANTHER* families**  **(subfamily/family /superfamily )** | **Pfam***  **(family/Domain)** | **SYSTERS***  **(cluster)** | **CDART***  **@NCBI** | **SVMProt* (family)** | **ProtoNet***  **Cluster**  **(cluster name)** |
| --- | --- | --- | --- | --- | --- | --- | --- | --- | --- |
|  | P44305 | Mycothiol acetyltransferase -like domain 1/2/3  1(5) | Acyl-CoA N-acyltransferases (Nat)  1(5) | N-terminal acetyltransferase  1(5) | Acetyltransferase (GNAT) family  1(5) | Cluster- 143401  (Ribosomal-protein-alanine acetyltransferase)  1(5) | N-Acyltransferase superfamily  1(4) | Zinc-binding  0(3) | Cluster 4140879  (Ribosomal-protein-alanine acetyltransferase)  1(5) |
|  | P44453 | S-adenosyl-L-methionine-dependent methyltransferases  1(4) | Ribosomal RNA small subunit methyltransferase C -like domain  1(5) | RNA methyltransferase  1(4) | Methyltransferase small domain N-terminal  1(4) | Cluster- 145158  (Ribosomal RNA small subunit methyltransferase C)  1(5) | MTS_N Superfamily  1(4) | EC 2.1.-.-: Transferases - Transferring One-Carbon Groups  1(3) | Cluster 4070293  (RRNA (guanine-N1(2)-)-methyltransferase)  1(5) |
|  | Q57163 | Radical SAM enzymes  1(3) | (Dimethylallyl)adenosine tRNA -like domain  1(5) | Radical SAM proteins  1(3) | Radical SAM superfamily/TRAM domain  1(3) | Cluster- 145170  (methyltransferase)  1(3) | Radical_SAM Superfamily  1(3) | EC 2.7.-.-: Transferases - Transferring Phosphorus-Containing Groups  1(3) | Cluster 4160521  (Uncharacterised protein family UPF0004, N-terminal)  0(2) |
|  | P44470 | alpha/beta knot  0(2) | Ribosomal RNA large subunit methyltransferase H -like domain  1(5) | 0(5) | Predicted SPOUT methyltransferase family  1(3) | Cluster-154116  (Hypothetical protein HI0033)  0(2) | SPOUT_MTase Superfamily  1(3) | EC 2.4.-.-: Transferases – Glycosyltransferases  1(3) | Cluster 4127703  RRNA (pseudouridine) methyltransferase activity  1(4) |
|  | P44490 | Cytidylytransferase family  1(4) | 3-deoxy-manno-octulosonate cytidylyltransferase -like domain  1(5) | CMP-2-keto-3-deoctulosonate (CMP-kdo) cytidyltransferase  1(5) | Cytidylyltransferase family  1(4) | Cluster- 146577  (3-deoxy-manno-octulosonate cytidylyltransferase)  1(5) | Glyco_tranf_GTA_type Superfamily  1(4) | EC 2.5.-.-: Transferases - Transferring Alkyl or Aryl Groups, Other than Methyl Groups  1(3) | Cluster 4134380  (3-deoxy-manno-octulosonate cytidylyltransferase activity)  1(5) |
|  | P44495 | P-loop containing nucleoside triphosphate hydrolases  0(2) | tRNA dimethylallyltransferase -like domain  1(4) | tRNA delta1(2)-isopentenylpyrophosphate transferase-related  1(5) | IPP transferase family  1(4) | Cluster-151684  (tRNA delta1(2)-isopentenylpyrophosphate transferase)  1(5) | MiaA Superfamily  (tRNA delta1(2)-isopentenylpyrophosphate transferase)  1(5) | EC 2.5.-.-: Transferases - Transferring Alkyl or Aryl Groups, Other than Methyl Groups  1(5) | Cluster 4147525  (TRNA isopentenyltransferase)  1(5) |
|  | P44419 | Glutamine synthase adenylyltransferase GlnE, domain 2  1(4) | Glutamate-ammonia-ligase adenylyltransferase -like domain ½  1(5) | Glutamine synthetase adenylyltransferase  1(4) | Glutamate-ammonia ligase adenylyltransferase family  1(5) | Cluster- 155118  (Glutamate-ammonia-ligase adenylyltransferase)  1(5) | Rel-Spo_like Superfamily  1(4) | EC 2.7.-.-: Transferases - Transferring Phosphorus-Containing Groups  1(5) | Cluster 3797527  ([Glutamate--ammonia-ligase] adenylyltransferase)  1(5) |
|  | P43933 | Nucleotidyltransferase  1(4) | Poly(A) polymerase -like domain  0(2) | 0(5) | Nucleotidyltransferase domain  1(4) | Cluster- 130134  (Hypothetical protein HI0073)  0(2) | Rel-Spo_like Superfamily  1(4) | All lipid-binding proteins  0(3) | Cluster 4139445  (Nucleotidyltransferase)  1(4) |
|  | P43934 | Family 1 bi-partite nucleotidyltransferase subunit  1(5) | Focal adhesion kinase -like domain  0(2) | 0(5) | Nucleotidyltransferase substrate binding protein like  1(4) | Cluster- 133255  (Hypothetical protein HI0074)  0(2) | NTase_sub_bind Superfamily  1(4) | All DNA-binding  0(2) | Cluster 4014672  (Nucleotidyltransferase substrate binding protein, HI0074)  1(4) |
|  | P44521 | GST C-terminal domain-like  1(5) | Glutathione S-transferase -like domain  1(5) | Glutathione S-transferase, gst, superfamily, gst domain containing  1(5) | Glutathione S-transferase, N-terminal domain  1(5) | Cluster- 140916  (Glutathione S-transferase) 1(5) | GST_C_family Superfamily 1(5) | EC 2.5.-.-: Transferases - Transferring Alkyl or Aryl Groups, Other than Methyl Groups  1(5) | Cluster 4114414  (Glutathione transferase)  1(5) |
|  | P43712 | Probable ACP-binding domain of malonyl-CoA ACP transacylase  1(5) | Polyketide synthase -like domain  0(2) | Malonyl COA-acyl carrier protein transacylase 1(4) | Acyl transferase domain  1(4) | Cluster-136895  (Malonyl CoA-acyl carrier protein transacylase)  1(5) | Acyl_transf_1 Superfamily 1(4) | EC 2.3.-.-: Transferases – Acyltransferases  1(5) | Cluster 4149732  (Malonyl CoA-acyl carrier protein transacylase)  1(5) |
|  | P43753 | PFL-like glycyl radical enzymes  1(3) | Formate acetyltransferase -like domain  1(5) | Formate acetyltransferase  1(4) | Pyruvate formate lyase  1(3) | Cluster- 155397  (formate acetyltransferase)  1(4) | RNR_PFL Superfamily 1(3) | EC 2.3.-.-: Transferases - Acyltransferases  1(3) | Cluster 4455182  (Formate C-acetyltransferase)  1(5) |
|  | P44567 | 0(5) | 0(5) | Lipid a biosynthesis lauroyl acyltransferase  1(5) | Bacterial lipid A biosynthesis acyltransferase  1(4) | Cluster) 152309  (Lipid A biosynthesis lauroyl acyltransferase)  1(5) | LPLAT Superfamily 1(4) | EC 2.7.-.-: Transferases - Transferring Phosphorus-Containing Groups  1(3) | Cluster 4162169  (Bacterial lipid A biosynthesis acyltransferase)  1(4) |
|  | P43912 | tRNA(m1G37)-methyltransferase TrmD  1(5) | tRNA (guanine-N(1)-)-methyltransferase -like domain  1(5) | 0(5) | tRNA (Guanine-1)-methyltransferase  1(5) | Cluster- 155624  (tRNA (Guanine-N(1)-)-methyltransferase)  1 (4) | tRNA_m1G_MT Superfamily  1(5) | EC 2.7.-.-: Transferases - Transferring Phosphorus-Containing Groups  1(3) | Cluster 3995627  (TRNA (guanine-N1-)-methyltransferase, bacteria)  1(5) |
|  | P44594 | tRNA-guanine transglycosylase  1(5) | Queuine tRNA-ribosyltransferase -like domain  1(5) | Queuine tRNA-ribosyltransferase  1(4) | Queuine tRNA-ribosyltransferase 1(5) | Cluster-145418  (Queuine tRNA-ribosyltransferase)  1(4) | tgt Superfamily 1(5) | EC 2.4.-.-: Transferases - Glycosyltransferases  1(5) | Cluster 4083606  (TRNA-guanine transglycosylase)  1(5) |
|  | P44595 | QueA-like  1(4) | S-adenosylmethionine:tRNA -like domain  1(4) | S-adenosylmethionine:trna ribosyltransferase-isomerase  1(4) | Queuosine biosynthesis protein  1(4) | Cluster- 155460  (S-adenosylmethionine:tRNA ribosyltransferase-isomerase)  1(4) | Queuosine_synth Superfamily 1(4) | EC 2.4.-.-: Transferases – Glycosyltransferases  1(3) | Cluster 4090929  (QueA-like)  1(4) |
|  | P43974 | Galactosyltransferase LgtC  1(5) | General stress protein A -like domain  0(2) | Glycosyltransferase 8 domain-containing protein  1(4) | Glycosyl transferase family 8  1(5) | Cluster- 139937  (Glycosyl transferase)  1(3) | Glyco_tranf_GTA_type Superfamily 1(5) | All DNA-binding  0(3) | Cluster 4133558  (Glycosyl transferase, family 8) 1(5) |
|  | P44603 | Clc chloride channel  0(2) | 0(5) | Inner membrane protein ygih  0(2) | Glycerol-3-phosphate acyltransferase  1(5) | Cluster- 146414  (Hypothetical protein HI0266)  0(2) | G3P_acyltransf Superfamily 1(5) | Transmembrane  0(2) | Cluster 4182942 (Protein of unknown function DUF205)  0(2) |
|  | P43855 | Phosphoribosyltransferases (PRTases)  1(4) | Phosphoribosyltransferase -like domain  1(4) | Orotate phosphoribosyltransferase 1(4) | Phosphoribosyl transferase domain  1(4) | Cluster- 146492  (orotatephosphoribosyl transferase)  1(4) | PRTases_typeI Superfamily  1(4) | EC 2.7.-.-: Transferases - Transferring Phosphorus-Containing Groups  1(3) | Cluster 3955266  (Orotate phosphoribosyl transferase)  1(5) |
|  | P71348 | PLP-dependent transferases  1(4) | Aspartate aminotransferase -like domain  1(4) | Aminotransferase related  1(4) | Aminotransferase class I and II  1(4) | Cluster- 141580  (Aminotransferase, class I)  1(4) | AAT_I Superfamily  1(4) | EC 2.6.-.-: Transferases - Transferring Nitrogenous Groups  1(5) | Cluster 4215049  (1-aminocyclopropane-1-carboxylate synthase)  1(4) |
|  | P44626 | Carbon-nitrogen hydrolase  0(2) | Apolipoprotein N-acyltransferase -like domain  1(4) | 0(5) | Carbon-nitrogen hydrolase  0(2) | Cluster- 112809  (Apolipoprotein N-acyltransferase)  1(4) | nitrilase Superfamily  0(2) | EC 2.7.-.-: Transferases - Transferring Phosphorus-Containing Groups  1(3) | Cluster 4154704  (Apolipoprotein N-acyltransferase)  1(5) |
|  | P44627 | alpha/beta knot  1(3) | Ribosomal RNA small subunit methyltransferase E -like domain  1(5) | Ribosomal RNA small subunit methyltransferase E  1(5) | RNA methyltransferase1(4) | Cluster- 153854  (Hypothetical protein HI0303)  0(2) | Methyltrans_RNA Superfamily  1(4) | Zinc-binding  0(3) | Cluster 4150590  (Ribosomal RNA small subunit methyltransferase E)  1(5) |
|  | P43985 | S-adenosyl-L-methionine-dependent methyltransferases  1(4) | tRNA (cmo5U34)-methyltransferase -like domain  1(4) | Methyltransferase  1(4) | Methyltransferase domain  1(4) | Cluster-146193  (Hypothetical protein HI0319)  0(2) | AdoMet_MTases Superfamily  1(5) | EC 2.5.-.-: Transferases - Transferring Alkyl or Aryl Groups, Other than Methyl Groups  1(4) | Cluster 3913455  (TRNA (cmo5U34)-methyltransferase)  1(5) |
|  | P44643 | S-adenosyl-L-methionine-dependent methyltransferases  1(5) | 23S rRNA (Uracil-5-)-methyltransferase RumA -like domain  1(5) | 23S rRNA (Uracil-5-)-methyltransferase  1(5) | tRNA (Uracil-5-)-methyltransferase  1(5) | Cluster- 139477  (23S rRNA (Uracil-5-)-methyltransferase rumA)  1(5) | AdoMet_MTases Superfamily 1(4) | EC 2.1.-.-: Transferases - Transferring One-Carbon Groups  1(3) | Cluster 4144764  ((Uracil-5)-methyltransferase)  1(5) |
|  | P44648 | S-adenosyl-L-methionine-dependent methyltransferases  1(3) | tRNA (guanine-N(7)-)-methyltransferase -like domain  1(5) | 3-Deoxy-d-manno-octulosonic-acid transferase/tRNA (guanine-n(7)-)-methyltransferase  1(3) | Putative methyltransferase  1(3) | Cluster- 128660  (Hypothetical protein HI0096)  0(2) | Methyltransf_4 Superfamily 1(3) | All lipid-binding proteins  0(3) | Cluster 4167817  (TRNA (guanine-N(7)-)-methyltransferase)  1(5) |
|  | P44665 | Radical SAM enzymes  1(3) | Ribosomal RNA large subunit methyltransferase N -like domain  1(5) | 23S rRNA methyltransferase  1(4) | Radical SAM superfamily  1(3) | Cluster- 143558  (Hypothetical protein)  0(2) | Radical_SAM Superfamily 1(3) | EC 2.7.-.-: Transferases - Transferring Phosphorus-Containing Groups  1(3) | Cluster 4146805  (Conserved hypothetical protein CHP00048)  0(2) |
|  | P44687 | Methylated DNA-protein cysteine methyltransferase  1(5) | O-6-alkylguanine-DNA/cysteine-protein -like domain  1(5) | Methylated DNA-protein cysteine methyltransferase domain  1(5) | 6-O-methylguanine DNA methyltransferase, ribonuclease-like domain  1(5) | Cluster- 141371  (Methylated-DNA--protein-cysteine methyltransferase)  1(5) | Methyltransf_1N Superfamily  1(5) | EC 2.1.-.-: Transferases - Transferring One-Carbon Groups  1(3) | Cluster 4122286  (Methylated-DNA-[protein]-cysteine S-methyltransferase activity)  1(5) |
|  | P43872 | Biotin dependent carboxylase carboxyltransferase domain  1(5) | Acetyl-coenzyme A carboxylase carboxyl transferase -like domain  1(5) | Acetyl-coenzyme A carboxylase carboxyl transferase alpha  1(5) | Acetyl co-enzyme A carboxylase carboxyltransferase alpha subunit  1(5) | Cluster-2613  0(5) | AccA Superfamily 1(4) | EC 2.1.-.-: Transferases - Transferring One-Carbon Groups  1(3) | Cluster 3893816  (Acetyl-CoA carboxylase, alpha subunit)  1(5) |
|  | P44708 | Class II glutamine amidotransferases  1(5) | Glucose-6-phosphate isomerase -like domain 1/2  1(5) | Glucosamine-fructose-6-phosphate aminotransferase, isomerising 1(5) | Glutamine amidotransferase domain  1(5) | Cluster- 143975  (Glucosamine--fructose-6-phosphate aminotransferase [)  1(5) | Gn_AT_II Superfamily  1(5) | EC 2.6.-.-: Transferases - Transferring Nitrogenous Groups  1(5) | Cluster 4138889  (Glucosamine-fructose-6-phosphate aminotransferase, isomerising)  1(5) |
|  | P44722 | Phosphoribosyltransferases (PRTases)  1(4) | Phosphoribosyltransferase -like domain 1(4) | 0(5) | Phosphoribosyl transferase domain  1(4) | Cluster- 128626  (PyrR bifunctional protein)  1(5) | PRTases_typeI Superfamily  1(4) | EC 2.4.-.-: Transferases - Glycosyltransferases  1(5) | Cluster 4150183  (Uracil phosphoribosyltransferase)  1(4) |
|  | P43853 | ATP phosphoribosyltransferase (ATP-PRTase, HisG), regulatory C-terminal domain  1(5) | ATP phosphoribosyltransferase -like domain  1(5) | ATP phosphoribosyltransferase (ATP-PRTase)  1(5) | ATP phosphoribosyltransferase  1(5) | Cluster- 154358  (ATP phosphoribosyltransferase)  1(5) | HisG Superfamily  1(5) | EC 2.4.-.-: Transferases – Glycosyltransferases  1(4) | Cluster 4066400  (ATP phosphoribosyltransferase, catalytic region)  1(5) |
|  | P44423 | PLP-dependent transferases  1(4) | Histidinol-phosphate aminotransferase -like domain  1(4) | Histidinol-phosphate aminotransferase  1(4) | Aminotransferase class I and II  1(4) | Cluster 1415801  (Histidinol-phosphate aminotransferase)  1(4) | AAT_I Superfamily 1(4) | EC 2.6.-.-: Transferases - Transferring Nitrogenous Groups  1(4) | Cluster 4113738  (Histidinol-phosphate aminotransferase)  1(5) |
|  | P44728 | Glucose-inhibited division protein B (GidB)  1(4) | Ribosomal RNA small subunit methyltransferase G -like domain  1(5) | 0(5) | rRNA small subunit methyltransferase G  1(5) | Cluster-150716  (Methyltransferase gidB)  1(5) | gidB Superfamily  1(5) | EC 2.7.-.-: Transferases - Transferring Phosphorus-Containing Groups  1(3) | Cluster 4144153  (Glucose inhibited division protein)  1(4) |
|  | P44009 | Class B acid phosphatase, AphA  1(4) | Class B acid phosphatase -like domain  1(4) | 0(5) | HAD superfamily, subfamily IIIB (Acid phosphatase  1(4) | Cluster- 154818  (Acid phosphatase, class B)  1(4) | Acid_phosphat_B Superfamily  1(4) | EC 3.1.-.-: Hydrolases - Acting on Ester Bonds  1(3) | Cluster 4031869  (HAD-superfamily phosphatase, subfamily IIIB, AphA)  1(4) |
|  | P44739 | 0(5) | 0(5) | 1,4-Dihydroxy-2-naphthoate octaprenyltransferase 1(5) | UbiA prenyltransferase family  1(4) | Cluster- 143752  (1,4-dihydroxy-2-naphthoate octaprenyltransferase)  1(5) | MenA Superfamily  1(5) | EC 2.5.-.-: Transferases - Transferring Alkyl or Aryl Groups, Other than Methyl Groups  1(5) | Cluster 4097268  (1,4-dihydroxy-2-naphthoate octaprenyltransferase)  1(5) |
|  | P44011 | UDP-Glycosyltransferase/glycogen phosphorylase  1(5) | Glycogen synthase -like domain  1(3) | ADP-heptose--lps heptosyltransferase 2  1(5) | Glycosyltransferase family 9 (heptosyltransferase)  1(5) | Cluster- 153768  (Lipopolysaccharide heptosyltransferas)  1(4) | Glycosyltransferase_GTB_type Superfamily  1(4) | EC 2.4.-.-: Transferases - Glycosyltransferases  1(5) | Cluster 4113634  (Glycosyl transferase, family 9)  1(4) |
|  | **P44749** | rRNA adenine dimethylase-like  1(4) | Ribosomal RNA methyltransferase -like domain  1(5) | Dimethyladenosine transferase  1(4) | Ribosomal RNA adenine dimethylase  1(4) | Cluster- 143213  (Dimethyladenosine transferase)  1(4) | AdoMet_MTases Superfamily  1(4) | EC 2.1.-.-: Transferases - Transferring One-Carbon Groups  1(4) | Cluster 4191386  (RRNA adenine dimethylase)  1(5) |
|  | **P44770** | Aspartate/ornithine carbamoyltransferase  1(5) | Aspartate carbamoyltransferase -like domain ½  1(5) | Carbamoyltransferase related  1(4) | Aspartate/ornithine carbamoyltransferase, carbamoyl-P binding domain  1(5) | Cluster- 138524  (Ornithine carbamoyltransferase, catabolic)  1(5) | OTCace Superfamily  1(5) | EC 2.1.-.-: Transferases - Transferring One-Carbon Groups  1(4) | Cluster 4126810  (Ornithine carbamoyltransferase complex)  1(5) |
|  | **P43886** | Serine acetyltransferase  1(5) | Serine acetyltransferase -like domain  1(5) | Sialic acid synthase-related  1(3) | Serine acetyltransferase, N-terminal  1(5) | Cluster- 141733  (Serine acetyltransferase)  1(5) | SATase_N Superfamily  1(5) | EC 2.3.-.-: Transferases - Acyltransferases  1(5) | Cluster 4052820  (Serine O-acetyltransferase)  1(5) |
|  | **P44787** | Formyltransferase  1(4) | Methionyl-tRNA formyltransferase -like domain  1(5) | Methionyl-tRNA formyltransferase  1(5) | Formyl transferase  1(4) | Cluster-139515  (Methionyl-tRNA formyltransferase)  1(3) | FMT_core Superfamily  1(4) | EC 2.1.-.-: Transferases - Transferring One-Carbon Groups  1(4) | Cluster 4122367  (Methionyl-tRNA formyltransferase)  1(5) |
|  | **P43889** | Nucleotide-diphospho-sugar transferases  1(4) | UDP-3-O-acylglucosamine N-acyltransferase -like domain 1/2/3  1(3) | Sugar-1-phosphate guanyl transferase  1(4) | MobA-like NTP transferase domain  1(3) | Cluster-141240  (Sugar phosphate nucleotydyl transferase)  1(3) | Glyco_tranf_GTA_type Superfamily  1(4) | EC 2.7.-.-: Transferases - Transferring Phosphorus-Containing Groups  1(5) | Cluster 4157074  (UDP-N-acetylglucosamine pyrophosphorylase)  1(3) |
|  | **P44805** | Nucleotidylyl transferase  1(4) | Phosphopantetheine adenylyltransferase -like domain  1(5) | Phosphopantetheine adenylyltransferase  1(5) | Cytidylyltransferase  1(4) | Cluster- 147015  (Phosphopantetheine adenylyltransferase)  1(5) | nt_trans Superfamily  1(4) | EC 2.7.-.-: Transferases - Transferring Phosphorus-Containing Groups  1(3) | Cluster 4143710  (Coenzyme A biosynthesis protein)  1(3) |
|  | **P44806** | UDP-Glycosyltransferase/glycogen phosphorylase  1(4) | 0(5) | 3-Deoxy-D-manno-octulosonic-acid transferase (kdotransferase)  1(5) | 3-Deoxy-D-manno-octulosonic-acid transferase (kdotransferase)  1(5) | Cluster- 152019  0(5) | Glycos_transf_N Superfamily  1(5) | EC 2.4.-.-: Transferases – Glycosyltransferases  1(3) | Cluster 4165664  (Three-deoxy-D-manno-octulosonic-acid transferase, N-terminal)  1(5) |
|  | **O05029** | Cytidylytransferase  1(5) | 2-C-methyl-D-erythritol 4-phosphate -like domain  1(5) | 0(5) | 2-C-methyl-D-erythritol 4-phosphate cytidylyltransferase  1(5) | Cluster- 142896  (Hypothetical 4-diphosphocytidyl-2C-methyl-D-erythritol synthase containing protein)  1(3) | Glyco_tranf_GTA_type Superfamily  1(4) | EC 2.7.-.-: Transferases - Transferring Phosphorus-Containing Groups  1(3) | Cluster 4161259  (2-C-methyl-D-erythritol 4-phosphate cytidylyltransferase)  1(5) |
|  | **P43859** | Phosphoribosyltransferases (PRTases)  1(4) | Phosphoribosyltransferase -like domain  1(4) | 0(5) | Phosphoribosyl transferase domain  1(4) | Cluster-147566  (Xanthine-guanine phosphoribosyltransferase)  1(5) | PRTases_typeI Superfamily  1(5) | EC 2.4.-.-: Transferases – Glycosyltransferases  1(3) | Cluster 4046091  (Xanthine phosphoribosyltransferase activity)  1(5) |
|  | **P44819** | Rhodanese/Cell cycle control phosphatase  1(4) | tRNA sulfurtransferase -like domain  1(5) | 0(5) | Rhodanese-like domain  1(4) | Cluster- 143968  (Thiosulfate sulfurtransferase glpE)  1(4) | RHOD Superfamily  1(4) | EC 2.8.-.-: Transferases - Transferring Sulfur-Containing Groups  1(5) | Cluster 3999068  (Thiosulfate sulfurtransferase)  1(5) |
|  | **P43859** | Phosphoribosyltransferases (PRTases)  1(4) | Phosphoribosyltransferase -like domain  1(4) | 0(5) | Phosphoribosyl transferase domain  1(4) | Cluster-147566  (Xanthine-guanine phosphoribosyltransferase)  1(5) | PRTases_typeI Superfamily  1(4) | EC 2.4.-.-: Transferases – Glycosyltransferases  1(3) | Cluster 4046091  (Xanthine phosphoribosyltransferase activity)  1(5) |
|  | **P44848** | Glycerol-3-phosphate (1)-acyltransferase  1(5) | 1-acyl-sn-glycerol-3-phosphate acyltransferase -like domain  1(5) | 1-acyl-sn-glycerol-3-phosphate acyltransferase  1(5) | Acyltransferase  1(4) | Cluster- 139770  (1-acyl-sn-glycerol-3-phosphate acyltransferase)  1(5) | LPLAT Superfamily  1(4) | C 2.3.-.-: Transferases – Acyltransferases  1(3) | Cluster 4179158  (1-acylglycerol-3-phosphate O-acyltransferase)  1(5) |
|  | **P44857** | Glycerol-3-phosphate (1)-acyltransferase  1(4) | 0(5) | Dihydroxyacetone phosphate acyltransferase  1(4) | Acyltransferase  1(4) | Cluster- 143942  (Glycerol-3-phosphate acyltransferase)  1(5) | LPLAT Superfamily 1(4) | EC 2.3.-.-: Transferases – Acyltransferases  1(5) | Cluster 3977038  (Glycerol-3-phosphate O-acyltransferase)  1(5) |
|  | P44308 | Adenylyltransferase  1(4) | Phosphopantetheine adenylyltransferase -like domain  1(3) | 0(5) | AAA domain  0(3) | Cluster- 150175  (Transcriptional regulator nadR)  0(3) | nt_trans Superfamily  1(4) | EC 2.3.-.-: Transferases – Acyltransferases  1(3) | Cluster 3970536  (Bifunctional transcriptional regulator NadR)  0(3) |
|  | P44868 | SpoU-like RNA 2'-O ribose methyltransferase  1(5) | tRNA (cytidine(34)-2'-O)-methyltransferase -like domain  1(5) | RNA methyltransferase  1(5) | SpoU rRNA Methylase family  1(5) | Cluster- 144145  (Hypothetical tRNA/rRNA methyltransferase)  1(3) | SpoU_methylase Superfamily  1(5) | Iron-binding  0(3) | Cluster 3995126  (S-adenosyl-L-methionine dependent tRNA/rRNA methyltransferase, SpoU, predicted)  1(5) |
|  | P44873 | Thiolase-like  1(5) | Thiolase -like domain  1(5) | acetyl-CoA acetyltransferase  1(5) | Thiolase, N-terminal domain  1(5) | Cluster-145889  (Acetyl-CoA acetyltransferase)  1(4) | cond_enzymes Superfamily  1(4) | EC 2.3.-.-: Transferases – Acyltransferases  1(4) | Cluster 4161050  (Thiolase)  1(5) |
|  | P44874 | NagB/RpiA/CoA transferase-like  1(5) | Acetate CoA-transferase subunit beta -like domain  1(5) | Ketoacid-coenzyme a transferase  1(5) | Coenzyme A transferase  1(5) | Cluster- 153562  (Glutaconate CoA-transferase, subunit B)  1(3) | CoA_trans Superfamily  1(5) | EC 2.8.-.-: Transferases - Transferring Sulfur-Containing Groups  1(5) | Cluster 3958844  (3-oxoacid CoA-transferase, subunit B)  1(5) |
|  | P44875 | CoA transferase alpha subunit-like  1(5) | Acetate CoA-transferase subunit alpha -like domain  1(5) | Ketoacid-coenzyme a transferase  1(5) | Coenzyme A transferase  1(5) | Cluster- 153560  (Acetate CoA-transferase beta subunit)  1(5) | CoA_trans Superfamily  1(5) | EC 2.4.-.-: Transferases – Glycosyltransferases  1(5) | Cluster 4027485  (3-oxoacid CoA-transferase, subunit A)  1(5) |
|  | P44878 | Nucleotide-diphospho-sugar transferases  1(4) | UTP-glucose-1-phosphate uridylyltransferase -like domain  1(5) | glucose-1-phosphate uridylyltransferase  1(5) | Nucleotidyl transferase  1(4) | Cluster) 141582  (Glucose-1-phosphate cytidylyltransferase)  1(3) | Glyco_tranf_GTA_type Superfamily  1(4) | EC 2.7.-.-: Transferases - Transferring Phosphorus-Containing Groups  1(5) | Cluster 3991628  (UTP--glucose-1-phosphate uridylyltransferase, bacterial and archaeal type)  1(5) |
|  | P31764 | Hexose-1-phosphate uridylyltransferase  1(5) | Galactose-1-phosphate uridylyltransferase -like domain  1(5) | Galactose-1-phosphate uridylyltransferase  1(5) | Galactose-1-phosphate uridyl transferase  1(5) | Cluster- 147838  (Galactose-1-phosphate uridylyltransferase)  1(5) | HIT_like Superfamily  1(3) | EC 2.7.-.-: Transferases - Transferring Phosphorus-Containing Groups  1(4) | Cluster 3987055  (Galactose-1-phosphate uridyl transferase, N-terminal)  1(5) |
|  | P31812 | S-adenosyl-L-methionine-dependent methyltransferases  1(4) | tRNA (uracil-5-)-methyltransferase -like domain  1(5) | tRNA (uracil-5-)-methyltransferase  1(5) | tRNA (Uracil-5-)-methyltransferase  1(5) | Cluster- 127030  (tRNA (Uracil-5-)-methyltransferase)  1(5) | AdoMet_MTases Superfamily  1(4) | EC 2.1.-.-: Transferases - Transferring One-Carbon Groups  1(5) | Cluster 3880425  (tRNA (Uracil-5-)-methyltransferase)  1(5) |
|  | P44901 | S-adenosyl-L-methionine-dependent methyltransferases  1(5) | Cap-specific mRNA -like domain  0(2) | 0(5) | Putative SAM-dependent methyltransferase  1(4) | Cluster- 156165  (Hypothetical protein HI0849)  0(2) | SAM_MT Superfamily  1(4) | All lipid-binding proteins  0(2) | Cluster 4020318  (Protein of unknown function DUF548)  0(2) |
|  | P44906 | SpoU-like RNA 2'-O ribose methyltransferase  1(5) | 23S rRNA (guanosine-2'-O-)-methyltransferase RlmB -like domain  1(5) | RNA methyltransferase  1(3) | RNA 2'-O ribose methyltransferase substrate binding  1(5) | Cluster- 143099  (Probable tRNA/rRNA methyltransferase)  1(3) | SpoU_methylase Superfamily  1(5) | EC 2.7.-.-: Transferases - Transferring Phosphorus-Containing Groups  1(3) | Cluster 4163001  (RNA methyltransferase TrmH, group 3)  1(4) |
|  | Q57491 | 0(5) | 0(5) | Exopolysaccharide biosynthesis polyprenyl glycosylphosphotransferase  1(5) | Bacterial sugar transferase  1(3) | Cluster-141559  (Undecaprenyl-phosphate galactosephosphotransferase)  1(5) | Bac_transf Superfamily  1(3) | EC 2.7.-.-: Transferases - Transferring Phosphorus-Containing Groups  1(3) | Cluster 4179062  (Exopolysaccharide biosynthesis polyprenyl glycosylphosphotransferase)  1(5) |
|  | P43852 | AICAR transformylase domain of bifunctional purine biosynthesis enzyme ATIC  1(5) | Phosphoribosylaminoimidazolecarboxamide -like domain  1(5) | Bifunctional purine biosynthesis protein  1(4) | AICARFT/IMPCHase bienzyme  1(5) | Cluster- 141405  (Bifunctional purine biosynthesis protein purH)  1(5) | AICARFT_IMPCHas Superfamily  1(5) | Iron-binding  0(2) | Cluster 4133184  (AICARFT/IMPCHase bienzyme, formylation region)  1(5) |
|  | P43844 | GABA-aminotransferase-like  1(4) | Serine hydroxymethyltransferase -like domain  1(5) | Serine hydroxymethyltransferase  1(5) | Serine hydroxymethyltransferase  1(5) | Cluster- 149068  (Serine hydroxymethyltransferase)  1(5) | AAT_I Superfamily  1(4) | EC 2.4.-.-: Transferases – Glycosyltransferases  1(5) | Cluster 3677077 (Serine hydroxymethyltransferase)  1(5) |
|  | P44930 | 0(5) | 0(5) | Prolipoprotein diacylglyceryl transferase  1(5) | Prolipoprotein diacylglyceryl transferase  1(5) | Cluster- 155260  (Prolipoprotein diacylglyceryl transferase)  1(5) | LGT Superfamily  1(5) | EC 2.4.-.-: Transferases – Glycosyltransferases  1(3) | Cluster 4325121 (Prolipoprotein diacylglyceryl transferase)  1(5) |
|  | P43888 | Trimeric LpxA-like enzymes  1(4) | 0(5) | GDP-mannose pyrophosphorylase  1(3) | UDP-3-O-[3-hydroxymyristoyl] glucosamine N-acyltransferase, LpxD  1(5) | Cluster- 142661  (UDP-3-O-[3-hydroxymyristoyl] glucosamine N-acyltransferase)  1(5) | LpxD Superfamily  1(5) | EC 2.7.-.-: Transferases - Transferring Phosphorus-Containing Groups  1(3) | Cluster 4094292 (UDP-3-O-[3-hydroxymyristoyl] glucosamine N-acyltransferase, LpxD)  1(5) |
|  | P44951 | GABA-aminotransferase-like  1(4) | Aminotransferase -like domain  1(4) | 2,4-diaminobutyrate 4-transaminase  1(5) | Aminotransferase class-III  1(4) | Cluster- 137279  (Diaminobutyrate--2-oxoglutarate aminotransferase)  1(5) | AAT_I Superfamily  1(4) | EC 2.6.-.-: Transferases - Transferring Nitrogenous Groups  1(3) | Cluster 4184290  (Aminotransferase class-III)  1(4) |
|  | P44083 | (Uracil-5-)-methyltransferase  1(5) | tRNA (uracil-5-)-methyltransferase -like domain  1(5) | 23S rRNA (uracil(747)-C(5))-methyltransferase  1(5) | 23S rRNA (uracil(747)-C(5))-methyltransferase  1(5) | Cluster- 139477  (23S rRNA (Uracil-5-)-methyltransferase rumA)  1(5) | 0(5) | EC 2.1.-.-: Transferases - Transferring One-Carbon Groups  1(5) | Cluster 4144764  ((Uracil-5)-methyltransferase)  1(5) |
|  | P44957 | Nucleotidylyl transferase  1(5) | Riboflavin biosynthesis protein ribF -like domain  1(5) | riboflavin kinase/FMN adenylyltransferase  1(5) | FAD synthetase  1(5) | Cluster- 142825  (Riboflavin biosynthesis protein ribF)  1(5) | nt_trans Superfamily  1(5) | EC 2.7.-.-: Transferases - Transferring Phosphorus-Containing Groups  1(5) | Cluster 4162927  (Riboflavin kinase/FAD synthetase)  1(5) |
|  | P44402 | Ribosomal protein L11 methyltransferase PrmA  1(5) | Ribosomal protein L11 methyltransferase -like domain  1(5) | Ribosomal protein L11 methyltransferase (L11 Mtase)  1(5) | Ribosomal protein L11 methyltransferase (PrmA)  1(5) | Cluster- 145162  (Ribosomal protein L11 methyltransferase)  1(5) | AdoMet_MTases Superfamily  1(5) | EC 4.3.-.-: Lyases - Carbon-Nitrogen Lyases  0(2) | Cluster 4117128 (Ribosomal protein L11 methyltransferase)  1(5) |
|  | P71366 | S-adenosyl-L-methionine-dependent methyltransferases  1(4) | Type III restriction-modification system EcoP15I -like domain  1(5) | 0(5) | DNA methylase  1(4) | Cluster- 144165  (Putative type III restriction-modification system HindVIP enzyme mod)  1(5) | N6_N4_Mtase Superfamily  1(4) | EC 2.1.-.-: Transferases - Transferring One-Carbon Groups  1(5) | Cluster 4024927  (DNA methylase N-4/N-6)  1(4) |
|  | P43887 | UDP N-acetylglucosamine acyltransferase  1(5) | UDP-3-O-acylglucosamine N-acyltransferase -like domain 1/2/3  1(5) | Serine acetyl transferase  1(4) | UDP-3-O-acylglucosamine N-acyltransferase -like domain 2  1(5) | Cluster- 142660  (Acyl-[acyl-carrier-protein]--UDP-N-acetylglucosamine O-acyltransferase)  1(5) | LbetaH Superfamily  1(4) | EC 1.1.-.-: Oxidoreductases - Acting on the CH-OH group of donors  1(3) | Cluster 4023454 (Acyl-[acyl-carrier-protein]--UDP-N-acetylglucosamine O-acyltransferase)  1(5) |
|  | P45025 | Enolpyruvate transferase, EPT  1(5) | 3-phosphoshikimate 1-carboxyvinyltransferase -like domain ½  1(5) | UDP-N-acetylglucosamine 1-carboxyvinyltransferase  1(5) | EPSP synthase (3-phosphoshikimate 1-carboxyvinyltransferase)  1(5) | Cluster- 144179  (UDP-N-acetylglucosamine 1-carboxyvinyltransferase)  1(5) | EPT_RTPC-like Superfamily  1(4) | EC 2.5.-.-: Transferases - Transferring Alkyl or Aryl Groups, Other than Methyl Groups  1(5) | Cluster 4645261  (Alpha-beta prism)  1(5) |
|  | P45042 | ADP-heptose LPS heptosyltransferase II  1(5) | Glycogen synthase -like domain 1/2  1(3) | Tetraacyldisaccharide 4'-kinase-related 1(3) | Glycosyltransferase family 9 (heptosyltransferase)  1(5) | Cluster- 153766  (Putative glycosyl transferase)  1(4) | Glycosyltransferase_GTB_type Superfamily 1(5) | EC 2.4.-.-: Transferases - Glycosyltransferases  1(3) | Cluster 4159581  (Glycosyl transferase, family 9)  1(5) |
|  | P45057 | MraW-like putative methyltransferases  1(5) | Ribosomal RNA small subunit methyltransferase H -like domain  1(5) | S-adenosyl-methyltransferase MraW  1(5) | MraW methylase family  1(5) | Cluster- 129839  (S-adenosyl-methyltransferase mraW)  1(5) | Methyltransf_5 Superfamily  1(5) | All DNA-binding  0(2) | Cluster 3996220  (Bacterial methyltransferase)  1(4) |
|  | P45062 | 0(5) | 0(5) | phospho-N-acetylmuramoyl-pentapeptide-transferase  1(5) | Phospho-N-acetylmuramoyl-pentapeptide-transferase signature 1  1(5) | Cluster- 150951  (Phospho-N-acetylmuramoyl-pentapeptide-transferase)  1(5) | GT_MraY-like Superfamily  1(4) | EC 2.7.-.-: Transferases - Transferring Phosphorus-Containing Groups  1(3) | Cluster 4156974 (Phospho-N-acetylmuramoyl-pentapeptide-transferase)  1(5) |
|  | P45065 | Peptidoglycan biosynthesis glycosyltransferase MurG  1(5) | Glycogen synthase -like domain 1/2  1(3) | Glycosyltransferase  1(4) | Glycosyltransferase family 28 N-terminal domain  1(4) | Cluster- 137053  (UDP-N-acetylglucosamine--N-acetylmuramyl-(pentapeptide) pyrophosphoryl-undecaprenol N-acetylglucosamine transferase)  1(5) | Glycosyltransferase_GTB_type Superfamily  1(4) | EC 2.4.-.-: Transferases - Glycosyltransferases  1(5) | Cluster 4093555  (N-acetylglucosaminyltransferase, MurG)  1(5) |
|  | P45078 | Phosphoribosyltransferases (PRTases)  1(5) | Phosphoribosyltransferase -like domain  1(5) | hypoxanthine-guanine phosphoribosyltransferase  1(5) | Phosphoribosyl transferase domain  1(5) | Cluster-128626  (PyrR bifunctional protein)  1(5) | PRTases_typeI Superfamily  1(5) | EC 2.4.-.-: Transferases – Glycosyltransferases  1(5) | Cluster 4138458  (Hypoxanthine phosphoribosyl transferase)  1(5) |
|  | Q57004 | PLP-dependent transferases  1(4) | Histidinol-phosphate aminotransferase -like domain  1(5) | Putative gntr-family regulatory protein  1(4) | Aminotransferase class I and II  1(4) | Cluster- 141580  (Histidinol-phosphate aminotransferase)  1(5) | AAT_I Superfamily  1(4) | EC 2.6.-.-: Transferases - Transferring Nitrogenous Groups  1(5) | Cluster 4113738 (Histidinol-phosphate aminotransferase)  1(5) |
|  | P44336 | GABA-aminotransferase-like  1(5) | Phosphoserine aminotransferase -like domain  1(5) | phosphoserine aminotransferase  1(5) | Aminotransferase class-V  1(5) | Cluster-145092  (Phosphoserine aminotransferase)  1(5) | AAT_I Superfamily  1(4) | EC 2.6.-.-: Transferases - Transferring Nitrogenous Groups  1(5) | Cluster 4108879  (Phosphoserine transaminase)  1(5) |
|  | P54689 | D-aminoacid aminotransferase-like PLP-dependent enzymes  1(5) | Branched-chain-amino-acid aminotransferase -like domain  1(5) | Subgroup IIII aminotransferase  1(4) | Aminotransferase class IV  1(5) | Cluster-140118  (Branched-chain amino acid aminotransferase)  1(5) | PLPDE_IV Superfamily  1(5) | EC 2.6.-.-: Transferases - Transferring Nitrogenous Groups  1(5) | Cluster 4031416 (Branched-chain amino acid aminotransferase)  1(5) |
|  | P45100 | S-adenosyl-L-methionine-dependent methyltransferases  1(4) | Ribosomal RNA large subunit methyltransferase M -like domain  1(5) | 0(5) | FtsJ-like methyltransferase  1(4) | Cluster- 151185  (Hypothetical protein HI1195)  0(2) | FtsJ Superfamily  1(4) | EC 5.3.-.-: Isomerases - Intramolecular Oxidoreductases  1(3) | Cluster 3995456  (YdgE, putative RNA 2'-O-ribose methyltranserase)  1(4) |
|  | P45106 | S-adenosyl-L-methionine-dependent methyltransferases  1(4) | Release factor glutamine methyltransferase -like domain  1(4) | Hemk methyltransferase family member  1(4) | Methyltransferase small domain  1(4) | Cluster- 147017  (DNA adenine methyltransferase)  1(4) | AdoMet_MTases Superfamily  1(4) | EC 2.1.-.-: Transferases - Transferring One-Carbon Groups  1(5) | Cluster 3845716  (Ribosomal protein L3-specific, glutamine-N5-methyltransferase)  1(5) |
|  | P45107 | Phosphotransacetylase  1(5) | Phosphate acetyltransferase -like domain  1(5) | phosphate acetyltransferase  1(5) | Phosphate acetyl/butaryl transferase  1(5) | Cluster- 146989  (Phosphate butyryltransferase)  1(5) | PTA_PTB Superfamily  1(5) | EC 1.1.-.-: Oxidoreductases - Acting on the CH-OH group of donors  1(5) | Cluster 4077505 (Phosphate acetyltransferase)  1(5) |
|  | P43854 | Phosphoribosyltransferases (PRTases)  1(5) | Phosphoribosyltransferase -like domain  1(5) | Amidophosphoribosyltransferase  1(5) | Glutamine amidotransferase, class-II  1(5) | Cluster- 143975  (Amidophosphoribosyltransferase)  1(5) | Gn_AT_II Superfamily  1(5) | EC 2.4.-.-: Transferases – Glycosyltransferases  1(5) | Cluster 4002446 (Amidophosphoribosyltransferase)  1(5) |
|  | P43857 | Phosphoribosyltransferases (PRTases)  1(5) | Phosphoribosyltransferase -like domain  1(5) | uracil phosphoribosyltransferase  1(5) | uracil phosphoribosyltransferase  1(5) | Cluster-95084  (Putative uracil phosphoribosyltransferase)  1(3) | PRTases_typeI Superfamily  1(5) | EC 2.4.-.-: Transferases – Glycosyltransferases  1(5) | Cluster 4082806 (uracil phosphoribosyltransferase)  1(3) |
|  | P43856 | Phosphoribosyltransferases (PRTases)  1(5) | Phosphoribosyltransferase -like domain  1(5) | adenine phosphoribosyltransferase  1(5) | Phosphoribosyl transferase domain  1(5) | Cluster-146490  (Xanthine phosphoribosyltransferase)  1(4) | PRTases_typeI Superfamily  1(5) | EC 2.4.-.-: Transferases – Glycosyltransferases  1(5) | Cluster 4136691  (Adenine phosphoribosyl transferase)  1(5) |
|  | **P45118** | CoA-dependent acyltransferases  1(5) | Dihydrolipoyllysine-residue acetyltransferase -like domain  1(5) | Dihydrolipoamide acetyl/succinyl-transferase-related  1(5) | 2-oxoacid dehydrogenases acyltransferase (catalytic domain)  1(5) | Cluster- 101152  (Putative acyltransferase)  1(3) | 2-oxoacid_dh Superfamily  1(5) | EC 2.3.-.-: Transferases - Acyltransferases1(5) | Cluster 4176860  (Dihydrolipoamide Transferase)  1(5) |
|  | **P45131** | O-acetyltransferase  1(4) | Homoserine O-acetyltransferase -like domain  1(5) | 0(5) | alpha/beta hydrolase fold  1(3) | Cluster- 141075  (Homoserine O-acetyltransferase)  1(5) | metX Superfamily  1(5) | EC 2.3.-.-: Transferases – Acyltransferases  1(5) | Cluster 4272343 (Homoserine O-acetyltransferase)  1(5) |
|  | **Q57140** | Cytidylytransferase  1(5) | N-acylneuraminate cytidylyltransferase -like domain  1(5) | CMP-N-acetylneuraminic acid synthase  1(5) | Cytidylytransferase  1(5) | Cluster- 146577  (3-deoxy-manno-octulosonate cytidylyltransferase)  1(5) | Glyco_tranf_GTA_type Superfamily  1(4) | EC 2.7.-.-: Transferases - Transferring Phosphorus-Containing Groups  1(3) | Cluster 4161073  (N-acylneuraminate cytidylyltransferase)  1(5) |
|  | **P45162** | RNA methyltransferase FtsJ  1(5) | Ribosomal RNA large subunit methyltransferase E -like domain  1(5) | Ribosomal RNA methyltransferase 2  1(5) | FtsJ-like methyltransferase  1(5) | Cluster- 145000  (Ribosomal RNA large subunit methyltransferase J)  1(5) | AdoMet_MTases Superfamily  1(4) | Magnesium-binding  0(2) | Cluster 4154030 (Ribosomal RNA large subunit methyltransferase J)  1(5) |
|  | **P45176** | (Trans)glycosidases  1(5) | 4-alpha-glucanotransferase -like domain  1(5) | 0(5) | 4-alpha-glucanotransferase  1(5) | Cluster-151695  (4-alpha-glucanotransferase)  1(5) | Glyco_hydro_77 Superfamily  1(5) | EC 2.4.-.-: Transferases – Glycosyltransferases  1(5) | Cluster 4125122  (Glycoside hydrolase, family 77)  1(5) |
|  | **P43796** | glucose-1-phosphate thymidylyltransferase  1(5) | Glucose-1-phosphate adenylyltransferase -like domain  1(5) | Glucose-1-phosphate adenylyltransferase  1(5) | Nucleotidyl transferase  1(4) | Cluster- 141241  (Glucose-1-phosphate thymidylyltransferase)  1(4) | Glyco_tranf_GTA_type Superfamily  1(5) | EC 2.7.-.-: Transferases - Transferring Phosphorus-Containing Groups  1(5) | Cluster 4122216  (Glucose-1-phosphate adenylyltransferase)  1(5) |
|  | **P43858** | Nucleoside phosphorylase/phosphoribosyltransferase catalytic domain  1(5) | Anthranilate phosphoribosyltransferase -like domain  1(5) | GMP synthase-related  1(3) | Glycosyl transferase family, a/b domain  1(5) | Cluster- 145503  (Pyrimidine-nucleoside phosphorylase)  1(4) | Glycos_transf_3 Superfamily  1(5) | EC 2.4.-.-: Transferases – Glycosyltransferases  1(5) | Cluster 4122390  (Anthranilate phosphoribosyl transferase)  1(5) |
|  | **P43846** | Formyltransferase  1(4) | Methionyl-tRNA formyltransferase -like domain  1(5) | Trifunctional purine biosynthetic protein adenosine-3-related  1(5) | Formyl transferase  1(4) | Cluster- 141404  (Phosphoribosylglycinamide formyltransferase)  1(5) | FMT_core Superfamily  1(4) | EC 2.1.-.-: Transferases - Transferring One-Carbon Groups  1(4) | Cluster 4123119  (Keyword 10133)  0(2) |
|  | **P45204** | Isoprenyl diphosphate synthases  1(5) | Geranylgeranyl pyrophosphate synthase -like domain  1(5) | Geranylgeranyl pyrophosphate synthase  1(5) | Polyprenyl synthetase  1(5) | Cluster- 152573  (Geranyltranstransferase)  1(5) | Isoprenoid_Biosyn_C1 Superfamily  1(5) | EC 2.5.-.-: Transferases - Transferring Alkyl or Aryl Groups, Other than Methyl Groups  1(5) | Cluster 4153444  (Polyprenyl synthetase)  1(5) |
|  | **O05074** | Nucleotidylyl transferase  1(5) | Phosphopantetheine adenylyltransferase -like domain  1(5) | Sugar kinase  1(3) | pfkB family carbohydrate kinase  1(5) | Cluster- 144233  (ADP-heptose synthase)  1(4) | ribokinase_pfkB_like Superfamily  1(5) | EC 2.7.-.-: Transferases - Transferring Phosphorus-Containing Groups  1(5) | Cluster 4096733  (Carbohydrate/purine kinase)  1(4) |
|  | **P45239** | Glycerol-3-phosphate (1)-acyltransferase  1(3) | 0(5) | Lipid a biosynthesis lauroyl acyltransferase  1(5) | Bacterial lipid A biosynthesis acyltransferase  1(5) | Cluster- 152309  (Lipid A biosynthesis lauroyl acyltransferase)  1(5) | LPLAT Superfamily  1(5) | Lipopolysaccharide biosynthesis  1(5) | Cluster 4162169  (Bacterial lipid A biosynthesis acyltransferase)  1(5) |
|  | **Q57287** | Nucleotide-diphospho-sugar transferases  1(5) | Polypeptide N-acetylgalactosaminyltransferase 1 -like domain  1(5) | Glycosyl transferase  1(5) | Glycosyl transferase, family 2  1(5) | Cluster- 139894  (Glycosyltransferase)  1(5) | Glyco_tranf_GTA_type Superfamily  1(5) | TC 9.B. Incompletely Characterized Transport Systems - Putative uncharacterized transport proteins  0(2) | Cluster 4143258  (Glycosyl transferase, family 2)  1(5) |
|  | **Q03421** | Enolpyruvate transferase, EPT  1(5) | 3-phosphoshikimate 1-carboxyvinyltransferase -like domain ½  1(5) | 3-phosphoshikimate 1-carboxyvinyltransferase  1(5) | EPSP synthase (3-phosphoshikimate 1-carboxyvinyltransferase)  1(5) | Cluster- 107580  (3-phosphoshikimate 1-carboxyvinyltransferase)  1(5) | EPT_RTPC-like Superfamily  1(5) | EC 2.5.-.-: Transferases - Transferring Alkyl or Aryl Groups, Other than Methyl Groups  1(5) | Cluster 4137915  (3-phosphoshikimate 1-carboxyvinyltransferase)  1(5) |
|  | **P45269** | Poly A polymerase C-terminal region-like  1(5) | Multifunctional CCA protein -like domain  1(5) | tRNA nucleotidyl transferase-related  1(5) | Poly A polymerase head domain  1(5) | Cluster- 142140  (tRNA nucleotidyltransferase)  1(5) | PolyA_pol_RNAbd Superfamily  1(5) | EC 2.7.-.-: Transferases - Transferring Phosphorus-Containing Groups  1(5) | Cluster 4142723  (Polynucleotide adenylyltransferase region)  1(5) |
|  | **P44425** | PLP-dependent transferases  1(5) | Aspartate aminotransferase -like domain  1(5) | Aspartate aminotransferase  1(5) | Aminotransferase class I and II  1(5) | Cluster- 138319  (Aspartate aminotransferase)  1(5) | AAT_I Superfamily  1(5) | EC 2.6.-.-: Transferases - Transferring Nitrogenous Groups  1(5) | Cluster 3902822  (Aspartate/other aminotransferase)  1(5) |

***True positive and true negative are denoted by “1” and “0”**

**Integers in () denotes the confidence level**
