# Supplementary material for: Functional Annotation of Conserved Hypothetical Proteins from Haemophilus influenzae Rd KW20
Source: PLoS One. 2013 Dec 31;8(12):e84263. doi: 10.1371/journal.pone.0084263 (PMC3877243; doi:10.1371/journal.pone.0084263)
Supplement: Table S7 — List of accuracy, sensitivity, specificity and ROC area of various bioinformatics tools used for predicting function of HPs from H. influenzae obtained after ROC analysis. (DOCX) [file pone.0084263.s007.docx]

| **S. No.** | **Software name** | **Accuracy of prediction** | **Sensitivity** | **Specificity** | **ROC Area** |
| --- | --- | --- | --- | --- | --- |
|  | BLAST | 100% | 100% | n/a | n/a |
|  | SMART | 100% | 100% | 100% | 1 |
|  | INTERPROSCAN | 100% | 100% | n/a | n/a |
|  | MOTIF | 100% | 100% | 100% | 1 |
|  | SUPERFAMILY | 95% | 100% | 44.4% | 0.603 |
|  | CATH | 90% | 100% | 33.3% | 0.596 |
|  | PANTHER | 82% | 100% | 5.3% | 0.351 |
|  | Pfam | 99% | 100% | 50% | 0.98 |
|  | SYSTERS | 97% | 100% | 76.9% | 0.893 |
|  | CDART | 99% | 100% | 50% | 0.615 |
|  | SVMProt | 94% | 100% | 57.1% | 0.918 |
|  | ProtoNet | 99% | 100% | 83.3%% | 0.997 |
|  | Average | 96.25% | 100% | 50.02% | 0.6627 |

**Table S7:** List of accuracy, sensitivity, specificity and ROC area of various bioinformatics tools used for predicting function of HPs from H. influenzae obtained after ROC analysis.
