# Supplementary material for: Functional Annotation of Conserved Hypothetical Proteins from Haemophilus influenzae Rd KW20
Source: PLoS One. 2013 Dec 31;8(12):e84263. doi: 10.1371/journal.pone.0084263 (PMC3877243; doi:10.1371/journal.pone.0084263)
Supplement: Table S8 — List of clusters formed by CLUSS online tool and predicted motif sequence site and sequence by MEME Suite in 429 HPs from H. influenzae. (DOCX) [file pone.0084263.s008.docx]

| **S.NO**  Table S8: List of clusters formed by CLUSS online tool and predicted motif sequence site and sequence by MEME Suite in 429 HPs from *H. influenzae* | **Cluster** | **UNIPROT ID** | **MEME results** | | | | | | |
| --- | --- | --- | --- | --- | --- | --- | --- | --- | --- |
|  |  |  | **Motif 1** | | **Motif 2** | | **Motif 3** | | **MAST function prediction** |
|  |  |  | **Start** | **Site** | **Start** | **Site** | **Start** | **Site** |  |
| 1 | **Cluster 1** | P71356 | 122 | DERDRIRQKE | 28 | HFETLN | 107 | LAMPLA | **Uncharacterized transporter** |
| 2 |  | P44300 | 65 | YQLWRLKQEN | 28 | HLRIVN | **48** | **TAYPVA** | No result |
| 3 | **Cluster 2** | **P43966** | 84 | SENACVN | 17 | LMHNEN | 70 | FHFTGPHK | No result |
| 4 |  | **P44299** | 7 | SESAVVC | 114 | QMHSEP | 104 | FHQTTPER | No result |
| 5 | **Cluster 3** | **P44297** | 99 | FCGTQD | 16 | CSMEHIALANW | 5 | FTHYQGNVSVK | No result |
| 6 |  | **P44116** | 55 | FCGIRF | 37 | RNMTDAEYALW | 138 | PPHFGSSPHKW | No result |
| 7 | **Cluster 4** | **P44112** | 17 | LAACSSQPE | 96 | YNYHYF | 33 | DMKTVQEY | No result |
| 8 |  | **P44283** | 3 | LIKVKSSAE | 21 | YPTSNF | 30 | DTGPVRTW | No result |
| 9 | **Cluster 5** | **Q57409** | 54 | QPNFGD | 33 | VRQSRV | 73 | WGSYDE | No result |
| 10 |  | **P45300** | 114 | IPNFLD | 54 | VRQRSH | 62 | YGSAIE | No result |
| 11 | **Cluster 6** | **P44465** | 16 | FPAKMT | 70 | ENFDQV | 1 | MTIEND | RutC family protein |
| 12 |  | **P71394** | 68 | FLADMK | 84 | EWVDHV | 1 | MTIQRI | No result |
| 13 | **Cluster 7** | P44260 | 66 | WVWDKLG | 56 | TGIGVQ | 29 | PHRIPYFEF | No result |
| 14 |  | P43994 | 47 | LSTNKIG | 25 | EGITVQ | 13 | PERYYLKSF | UPF0125 |
| 15 | **Cluster 8** | P44156 | 21 | AGKNLPRPSDNELAQMQPITGCEAQMWFQIMPKND | - | No motif | 59 | QFSGFS | Uncharacterized SufE-like |
| 16 |  | P44185 | 26 | ACTNKITTKPEYIYPPQAYTAPCVKTAFTGETYGD | 71 | ERDKCASQVDH | 19 | CLSLFL | No result |
| 17 |  | P43943 | - | No motif | 5 | ERKATDSAYHE | 102 | WKSLFE | No result |
| 18 |  | P44213 | - | No motif | - | No motif | 25 | QKSLFK | No result |
| 19 | **Cluster 9** | P44111 | 2 | PVYSITDKDLSKRIQ | 30 | YVSEGR | 22 | TLKEKT | No result |
| 20 |  | P43937 | 40 | GSYQLKQKIFSDIVQ | 77 | YINMAW | 4 | TLKEHA | No result |
| 21 | **Cluster 10** | P71346 | 30 | WQTQLISPHFV | 45 | PNGFSV | 70 | MYKAIN | Ribosome-associated inhibitor A |
| 22 |  | P44076 | 9 | AETCPDTAPFV | 71 | PNGDVV | 61 | GYLGIP |  |
| 23 | **Cluster 11** | P44649 | 43 | DRFINEVIQP | 106 | WWEYPT | 75 | GKCDESHR | No result |
| 24 |  | P44040 | 26 | DALPNGIMQP | 53 | KNTIPT | 16 | GCASESVK | No result |
| 25 | **Cluster 12** | P45085 | 65 | ANWPTF | 41 | HCKVPFGYVDILQHPD | 88 | EMYQAG | Glutaredoxin-4 |
| 26 |  | P43987 | 79 | ANVPFF | 42 | GQNVSFYLKEINQTLD | 16 | QLYQAH | No result |
| 27 | **Cluster 13** | **P44515** | 5 | YGIKNCDTVKK | 51 | WDVLVNKRS | 23 | HNIEHKLHDYR | No result |
| 28 |  | **P44191** | 70 | TGNPTFATVMK | 8 | FDVAEHLTS | 47 | RNMSQIARDAG | No result |
| 29 | **Cluster 14** | O86242 | 67 | DGYMVA | 34 | GLDLYGH | 91 | RKTPPPEN | No result |
| 30 |  | P44188 | 54 | DAAMCA | 69 | CTHLFGH | 43 | RKKPAIRY | No result |
| 31 | **Cluster 15** | **P31811** | 33 | QVLEQYG | 12 | FDDKNYPRG | 78 | WNKYRTRI | No result |
| 32 |  | **P71357** | 22 | QVIEQYG | 49 | PVDLNYLRP | 35 | FNMFLAQI | Putative antitoxin RelB |
| 33 | **Cluster 16** | P43938 | 29 | YNNPEHKEMM | 45 | RWIARR | 15 | NSANAFTET | No result |
| 34 |  | P44672 | 90 | YNNPNVKESC | 7 | EKAAQR | 49 | NSEDQVFEQ | Iron-binding protein IscA |
| 35 | **Cluster 17** | **P44075** | 91 | TVMRFSN | 49 | YIVDFYC | 65 | DGSQHYKP | No result |
| 36 |  | **P44107** | 33 | TEDLFSN | 18 | GIANWIG | 61 | DPAIDIRP | No result |
| 37 | **Cluster 18** | **P44198** | 19 | ANREDFS | 63 | TIKRQGFYLQIAKQDD | 3 | CAIYKSK | YcgL domain-containing protein |
| 38 |  | P44053 | 106 | ANNPDFS | 74 | TIFSETSYHCSAKVLM | 15 | CIISESK | No result |
| 39 | Cluster 19 | P44025 | 11 | WDCRRG | 25 | MPFYLTH | - | No motif | No result |
| 40 |  | P45026 | 75 | WKRDRA | 54 | MPYFSTG | 23 | GENAHF | No result |
| 41 |  | P44759 | - | No motif | - | No motif | 68 | TPPPHY | Protein SlyX homolog |
| 42 |  | P56507 | - | No motif | - | No motif | - | No motif | No result |
| 43 | Cluster 20 | P44186 | 17 | FGALRY | 71 | IKTQPCAH | 57 | ERIATENA | Mu-like prophageFluMu protein |
| 44 |  | P71390 | 19 | VGTVGY | 4 | IKTIRCTF | 30 | PRCKVINF | No result |
| 45 | **Cluster 21** | **P44027** | 46 | LAFFTQKARDVESEPCEIQSEITKVDDGYLLKADFTFCCQAELVIF | 24 | DNRDCSQVIEYIY | 11 | CVGCNT | No result |
| 46 |  | **P44686** | 45 | LERLTKKARNTESDPCEIKSEIVAVENGVQLNASFTFSCQAEAMIF | 23 | DGSDCSVEVHQFY | 15 | CVDVGT | UPF0381 |
| 47 | **Cluster 22** | **P43968** | 11 | QRSFTE | 26 | DYFDPV | 40 | IGNLGI | No result |
| 48 |  | **P44017** | 61 | ARNLTA | 43 | DYFAKC | 49 | KGNCMI | No result |
| 49 | **Cluster 23** | **P44228** | 16 | GYRRAGFSFHLGDN | 5 | FCVVVQNRIK | 58 | QEGGEG | Mu-like prophageFluMu protein gp35 |
| 50 |  | **P44208** | 18 | YYRPEMPTFKACYF | 55 | FKAAVPEIIW | 38 | QEQGWG | No result |
| 51 | **Cluster 24** | **P44234** | 52 | MVECTVFNC | 85 | VYLLPG | 10 | IIRLNGKEW | Mu-like prophageFluMu tail tube protein |
| 52 |  | **P44219** | 46 | MFSTFLFAC | 36 | VYLDKP | 69 | QRREQGKEQ | No result |
| 53 | **Cluster 25** | **P44205** | 34 | FCGPAG | 23 | FRQIHH | 6 | VFSAGSFCY | No result |
| 54 |  | **P44733** | 297 | MCSPNF | 51 | DEQIHH | 305 | MFIAVEPAY | DNA recombination protein RmuC homolog |
| 55 | **Cluster 26** | **O05023** | 21 | VARTQPTKKDLKTQNPILHSDQGW | 81 | GRLKTECYYD | 49 | VGYQAIL | No result |
| 56 |  | **P44172** | 55 | VLSVSPITLDELTQPHAKQENMGL | 42 | GRFEDNQYFC | 96 | WVIQFSL | UPF026 |
| 57 | **Cluster 27** | **P45083** | 114 | WQIDIR | 40 | MPVDHRTMQPF | 14 | QLCSNS | Putative esterase |
| 58 |  | **P44972** | 19 | YQIMIS | 73 | DPVPPKTNNND | 35 | PTCSCY | Putative membrane protein insertion efficiency |
| 59 | **Cluster 28** | **Q57525** | 104 | QSEHSD | 1 | MPYLSV | 25 | KIVIADPNTR | Putative inactive aspartokinase 3 |
| 60 |  | **P44218** | 69 | QSEPVA | 52 | NPHLSS | 7 | KIVVHCSATR | No result |
| 61 | **Cluster 29** | **P44220** | 74 | ERMHND | 8 | FSGVIALY | 63 | NLISRS | No result |
| 62 |  | **P71376** | 86 | YRPSEE | 11 | FLKGLAHH | 65 | NAIVRE | RNA-binding protein |
| 63 | **Cluster 30** | **P45138** | 24 | WGIECQ | 85 | PLFTLP | 109 | MERRKWQGK | Ribosome maturation factor RimP |
| 64 |  | **P44194** | 43 | FGRHYQ | 58 | PNFPKP | 1 | MESIKLSQK | No result |
| 65 | **Cluster 31** | **P44711** | 43 | KITINGAHNCR | 57 | IDPSLMEDD | 82 | RRAEEL | Nucleoid-associated protein |
| 66 |  | **P44026** | 68 | KPFYHFAAGCF | 3 | WDLSGGMVD | 85 | RQAEEL | No result |
| 67 | Cluster 32 | Q57120 | 35 | SGSDRILSPLNQSWD | 58 | VSDDFM | 7 | FMTNRSQAVRLPAEV | Putative antitoxin VapB2 |
| 68 |  | **P44864** | 19 | SCGLLIFSPVSQSSD | 340 | NGYGYM | 394 | FGISRKGTPVNPAGW | No result |
| 69 | **Cluster 33** | **P44173** | 15 | FYCGTN | 7 | WVMQNKA | 78 | FPMEST | No result |
| 70 |  | **P44034** | 51 | FHSDTL | 68 | WLKHKYA | 44 | FPIEQS | No result |
| 71 | **Cluster 34** | **P44048** | 35 | SKQAWGEW | 3 | RTVFCEY | 18 | DFQLYP | Probable Fe(2+)-trafficking protein |
| 72 |  | **P44583** | 59 | SKKAELHH | 121 | FAVFYPY | 101 | DYQLCA | No result |
| 73 | **Cluster 35** | **P44119** | 83 | GKEWQLVMNEIFKLPA | 39 | YQMFGRV | 33 | YRCACQ | Protein SprT |
| 74 |  | **P45154** | 50 | GKVSYKEMPLAFIQPD | 73 | YCGSCRV | 116 | YQCRSG | Uncharacterized ferredoxin-like protein |
| 75 | **Cluster 36** | **P44471** | 20 | VHFDVR | 37 | CTGTSS | 91 | AREMYQ | Ribosomal silencing factor RsfS |
| 76 |  | **P44492** | 82 | YHFDLY | 110 | CLIEWS | 50 | TRGMLQ | UPF0079 ATP-binding protein |
| 77 | **Cluster 37** | **P44190** | 13 | SWLSKL | 35 | QFGNFG | 58 | QGYRVY | No result |
| 78 |  | **P43998** | 51 | YRLSHL | 14 | QFGQFG | 20 | VPYVVG | No result |
| 79 | Cluster 38 | O86237 | 104 | QPAHCW | 48 | DFYYPF | 34 | KGKHAIRFLC | No result |
| 80 |  | **P44160** | 86 | QPAHGT | 75 | PICYPW | 29 | CGWNTKNFPC | Putative glucose-6-phosphate 1-epimerase |
| 81 | **Cluster 39** | **P44243** | 197 | RYQNQDKV | 107 | NCLHGIIP | 117 | GRLSKE | No result |
| 82 |  | **P44065** | 21 | RYRNQLIV | 1 | MTYHKVSP | 10 | GRMSKE | No result |
| 83 | **Cluster 40** | **P44074** | 187 | RNRNWF | 159 | HKEGERW | 125 | HYIENFPT | No result |
| 84 |  | **P45075** | 20 | CALAWF | 4 | IKMNIRW | 74 | HYTVNEQT | Lipopolysaccharide export system protein LptC |
| 85 | **Cluster 41** | **P44082** | 89 | VRYPTS | 60 | RQFLGLACE | 4 | MFRMNKG | No result |
| 86 |  | **P43963** | 244 | IRYKTF | 217 | RQFKGVEVD | 151 | MERGAKA | No result |
| 87 | **Cluster 42** | **P44154** | 224 | RCCPSCGANWALKDAIFDTFHFKCDTCR | 6 | TYFRLT | 154 | KWQENW | No result |
| 88 |  | **P44003** | 30 | RLAHYWQADVNTPQVDFMLLHIACSLGR | 105 | TYFLAN | 5 | KQLERW | No result |
| 89 | **Cluster 43** | **P44041** | 63 | GYRDCH | 10 | SYSKQF | 27 | PNVLIGSKYITAIHC | Putative toxin RelE |
| 90 |  | **P44241** | 96 | CLRDCT | 69 | SNSKRF | 108 | ANHWRIFMYTPAVSS | Mu-like prophageFluMu protein gp48 |
| 91 | **Cluster 44** | **P44210** | 33 | ECEALG | 46 | WFDIGK | 10 | GEMMSPRN | No result |
| 92 |  | **P43999** | 101 | EAQALG | 169 | WFDSDK | 73 | GKIMVYRN | No result |
| 93 | **Cluster 45** | **P44093** | 68 | WLKENGCTQFYFKYCST | 281 | IHNENYIE | 151 | NLMRLM | No result |
| 94 |  | **P44886** | 100 | WVKKVASEPIGERYCVT | 134 | IPRENNQE | 38 | WIMSQM | Uncharacterized acyl-CoA thioester hydrolase |
| 95 | **Cluster 46** | **P43995** | 71 | KPFEPNPSM | 50 | SEHQVLRLAVNDQ | 31 | RTLTDFV | No result |
| 96 |  | **P45298** | 54 | KPFFALHDH | 165 | STHRILDTLEDMQ | 217 | RTKGEMV | Ribosomal RNA small subunit methyltransferase |
| 97 | **Cluster 47** | **P44230** | 69 | HCCYIARYFLEKNRAT | 21 | QIAWLP | 124 | MMESAGSVWG | Mu-like prophageFluMu protein gp36 |
| 98 |  | **P45071** | 76 | KHHQIKIIFLEADRAT | 188 | NPHWDP | 163 | IVESFGFKYG | UPF0042 nucleotide-binding protein |
| 99 | **Cluster 48** | **P45077** | 221 | WVGENC | 286 | PDWFSISERPH | 343 | GHAGGIHNW | Protein PmbA homolog |
| 100 |  | P44047 | 20 | WYTENY | 97 | RVWYLVIKNLH | 74 | GFAGLIAQW | No result |
| 101 | **Cluster 49** | **P44900** | 62 | YEPIPG | 43 | NELTDEVIIYQLKM | 36 | SGFQKP | No result |
| 102 |  | **P44221** | 47 | GLPIPT | 58 | NPFAHRCVSCQQDW | 39 | AGRQCS | No result |
| 103 | **Cluster 50** | **P44072** | 7 | RYQKAV | 55 | GNMTDNS | 93 | VTGFEI | No result |
| 104 |  | **P46455** | 6 | RYQQAG | 27 | GWCLCAY | 61 | VIGHWI | No result |
| 105 | **Cluster 51** | **P44284** | 75 | DKRTNQ | 105 | EIEVIC | 82 | HKMDPN | No result |
| 106 |  | **P43947** | 162 | DKTKYQ | 115 | PIFVAC | 5 | HIKSPM | No result |
| 107 | **Cluster 52** | **P44974** | 352 | IVIHLIGSHPAFCER | 218 | GESMRKDYMSLYGFPLKTTPFLERVKGTVFENYYSAAPNTQPSLQLT | 286 | AGVKTYWISNQGKIGEFDT | Putative phosphoethanolamine transferase |
| 108 |  | **P71367** | 363 | IVLHIYGSHPMACDR | 226 | GESARKDYHHAYGYPIENTPFMSNAKGTLIDGFRSAGTNTVASLRLM | 296 | AGIKTYWLSNHGMIGKFDT | Putative phosphoethanolamine transferase |
| 109 | **Cluster 53** | **P44281** | 17 | IRDWLGY | 9 | NYHSTY | 26 | AGEEKA | No result |
| 110 |  | **P44232** | 14 | IRDPETF | 34 | SYWLNH | 26 | SGEDKP | Mu-like prophageFluMu protein gp38 |
| 111 | **Cluster 54** | **P44282** | 21 | FFVDERE | 66 | SNYLTQ | 41 | FDLALIETH | No result |
| 112 |  | **P44676** | 182 | YFFDYTE | 172 | KNYPTT | 65 | FDEAVDDCS | Uncharacterized tRNA/rRNAmethyltransferase |
| 113 | **Cluster 55** | **P44277** | 4 | KLFFHI | 123 | EQAIKLF | 13 | CFSLPI | No result |
| 114 |  | **P44148** | 19 | KLFYHI | 29 | EQALKLF | 9 | DFVPEM | No result |
| 115 | **Cluster 56** | **P44842** | 47 | PNARHHCW | 142 | DCDYGQ | 95 | ISAVVVRYY | IMPACT family member |
| 116 |  | **P44180** | 149 | PMIGNHVA | 128 | SCGYDC | 28 | ISKVGVFDY | No result |
| 117 | **Cluster 57** | **P44212** | 64 | RMHQRP | 39 | HWDLAM | 9 | KSVENF | No result |
| 118 |  | **O86228** | 75 | RLFQDP | 56 | GIELSM | 20 | KSVITY | Uncharacterized HTH-type transcriptional regulator |
| 119 | **Cluster 58** | **Q57144** | 206 | AEKWQCELVG | 132 | HIDPFEGYF | 76 | FCDKRF | No result |
| 120 |  | **P44285** | 19 | MMMSGCVLVG | 384 | HDTPNRGLF | 252 | FHNGIF | Putative L,D-transpeptidase |
| 121 | **Cluster 59** | **P44031** | 59 | HLDGEIW | 87 | LHQFMK | 80 | MDGRFV | No result |
| 122 |  | **P44280** | 238 | HLSGEHW | 203 | LHQCKA | 215 | QPGRFV | No result |
| 123 | **Cluster 60** | **P44132** | 326 | CLYLKHWC | 62 | FERGLF | 93 | YHGPLP | No result |
| 124 |  | **Q57134** | 77 | VQYREKHG | 107 | RDRIIF | 10 | LCGALV | No result |
| 125 | **Cluster 61** | **P44992** | 187 | NPVPMPLAELYTALETRAVDAQEHPIGIFWSSKLYEVQKYLSLTNHGYTP | 142 | AFWDVGFR | 111 | FKDREHVY | No result |
| 126 |  | **P71336** | 185 | NPQSMPLSEVFTALEQKVIDGQENPYMLIKDSGLYEVQKYIIQSNHIFSP | 141 | AYGMNGFR | 101 | FGVWEWPY | No result |
| 127 | **Cluster 62** | **P44744** | 51 | QNQMICDLC | 19 | YHQKLM | 189 | KSKTSR | No result |
| 128 |  | **P45173** | 150 | MFQAACQTC | 37 | YHWNIK | 1 | MSKTSI | No result |
| 129 | **Cluster 63** | **P44270** | 32 | EKWNGQNW | 41 | AEIQDR | 139 | KKVYPTKS | No result |
| 130 |  | **P44139** | 37 | MQLNGKNW | 54 | PHLSDT | 100 | KKVSQPKS | No result |
| 131 | **Cluster 64** | **P44133** | 111 | WKQPKTEK | 149 | DFAEYNP | 237 | HSFQKWT | No result |
| 132 |  | **P45146** | 49 | GKIPYDDY | 193 | SCMETNP | 22 | QISKRWK | No result |
| 133 | **Cluster 65** | **P44484** | 89 | EQYRKI | 53 | EEFSRI | 122 | YTQQMP | Putative TRAP transporter small permease protein |
| 134 |  | **P71360** | 67 | RQYAWI | 235 | EALNRW | 56 | YKKQLP | Uncharacterized transporter |
| 135 | **Cluster 66** | **P43932** | 112 | HQEHHESESKNKVSY | 42 | RQRQSGR | 4 | WIADPEAW | UPF0053 protein |
| 136 |  | **P43953** | 38 | AREETYLESKFGDEY | 54 | RYKQKVR | 15 | WLGNSLAW | No result |
| 137 | **Cluster 67** | **P45145** | 113 | HYLYQMQSFTHKRKK | 133 | ENQVKQ | 95 | VPNIVSTC | UPF0299 membrane protein |
| 138 |  | **P44097** | 33 | DYYNPVSTFAVKMTD | 135 | EPLLKP | 60 | VKNIDTSA | No result |
| 139 | **Cluster 68** | **P46490** | 247 | VYDQGWFH | 126 | EDRKQR | 64 | YFLRKR | UPF0721 transmembrane protein |
| 140 |  | **P44579** | 147 | VAHELWHT | 295 | NKKKVR | 172 | YLSRKW | Uncharacterized transporter |
| 141 | **Cluster 69** | **P44994** | 93 | DMMMLICC | 67 | ENQHVS | 101 | YLIIEGSWIQFQ | Putative TRAP transporter small permease protein |
| 142 |  | **O86230** | 20 | DNISLFGC | 77 | ENWQIS | 93 | YLAIGCSWLAYW | Uncharacterized transporter |
| 143 | **Cluster 70** | **P44252** | 257 | RMEKNM | 81 | ENLPHF | 244 | DWRVQKGEF | Lipoprotein-releasing system transmembrane protein LolC |
| 144 |  | P44272 | 147 | EMTKNK | 30 | ENLSTF | 113 | DWQQRTVEM | No result |
| 145 | **Cluster 71** | **P43952** | 188 | VTYIKPQCDNDCLA | 333 | DLNRMEC | 1 | MKKFAL | No result |
| 146 |  | **P44293** | 55 | VGNITQQIDDDEFW | 113 | DVLRIEK | 1 | MKKFAL | No result |
| 147 | **Cluster 72** | **P44201** | 18 | CKDWLH | 137 | MQHYRST | - | No motif | No result |
| 148 |  | **P44145** | 31 | KKKWIK | - | No motif | 47 | HNRMPSYCNLDRLMM | No result |
| 149 |  | **P44129** | - | No motif | 77 | RQVKRQT | - | No motif | No result |
| 150 |  | **P44170** | - | No motif | - | No motif | 231 | HQSFAGNPTLVFAGM | No result |
| 151 | **Cluster 73** | **Q57147** | 52 | KIDKPMR | 84 | SASSAVTMIGLEP | 120 | QWHLLA | No result |
| 152 |  | **P44125** | 35 | KFDKDSR | 17 | CANKDVYFNGAEG | 42 | QWGLNQ | No result |
| 153 | **Cluster 74** | **P44936** | 21 | HEYGHFW | 31 | KCGIKVHR | 419 | ICYRIG | Putative zinc metalloprotease HI_0918 |
| 154 |  | **P44073** | 5 | TVKPNFW | 53 | KVAREVQR | 91 | APIRAG | No result |
| 155 | **Cluster 75** | **P44064** | 34 | CVNKYH | 145 | SHEIPH | 73 | SYFHTGE | No result |
| 156 |  | **P44224** | 247 | CVPKAS | 299 | TQLLPH | 474 | SYMTGGE | Mu-like prophageFluMu protein gp28 |
| 157 | **Cluster 76** | **Q57022** | 115 | VMTHGNYAFCD | 10 | MPVYNAEC | 193 | SHQASSFVRIG | Uncharacterized glycosyltransferase |
| 158 |  | **P44169** | 268 | VSNTGHWGNGD | 89 | MLEYKADF | 72 | RQQNSSVVDQG | No result |
| 159 | **Cluster 77** | **P44225** | 28 | MPLQYHYSDH | 156 | NVHWRD | 260 | MSIGHNAGGIIPRGM | Mu-like prophageFluMu protein gp29 |
| 160 |  | **P45202** | 13 | IPFILHTYDH | 26 | NQHFGD | 94 | KSTGYLVGGISPLGQ | Cys-tRNA(Pro)/Cys-tRNA(Cys) deacylaseYbaK |
| 161 | **Cluster 78** | **P44043** | 95 | RDMPTH | 77 | FGKNLSSYP | 66 | GVMRLT | No result |
| 162 |  | **P43931** | 164 | NDSPTV | 244 | YGIELGYFP | 298 | GVGLLT | UPF0283 membrane protein |
| 163 | **Cluster 79** | **P44222** | 5 | HSHYTLACESIRSPLCK | 51 | AIFIGAFW | 121 | QVDAMSHQ | No result |
| 164 |  | **P44898** | 190 | HLIYAWADAYLYRPITM | 36 | AIFIGARY | 538 | QPDGTQYH | No result |
| 165 | **Cluster 80** | **P44827** | 84 | VYAAGRLDRDSEGLLILTNNGELQHRLADPKFKTEKTYWVQVEGI | 51 | TKVVLFNKPFDVLTQFTDEQGRATLKD | 178 | WLEIKISEGRNRQVRRMTAHIGFP | Ribosomal large subunit pseudouridine synthase E |
| 166 |  | **P45104** | 176 | WIAVGRLDINTSGLLLFTTDGELANRLMHPSREVEREYSVRVFGQ | 140 | CRVLMYYKPEGELCTRSDPEGRATVFD | 256 | WYDVTLMEGRNREVRRLWESQGIQ | Ribosomal large subunit pseudouridine synthase B |
| 167 | **Cluster 81** | **P44941** | 260 | DLLPNH | 326 | HHWEFTPN | 65 | SQNPHF | No result |
| 168 |  | **P44223** | 14 | DLLPPN | 109 | MAFDLTLN | 122 | ANDPKF | Mu-like prophageFluMu protein gp27 |
| 169 | **Cluster 82** | **P44287** | 34 | QAECPRCHHLLASGTRWSLHR | 89 | WDGIWKMAVGGYEY | 344 | QMRLLH | No result |
| 170 |  | **P44080** | 49 | QAELQRTLQLIGKDLRRLGFR | 181 | AGGGWADLLDNNEY | 40 | QHMLLH | No result |
| 171 | **Cluster 83** | **P44477** | 7 | VDAQKE | 98 | VARGMED | 70 | ADRPAG | No result |
| 172 |  | **P44136** | 30 | ASWQKE | 190 | CSCGHQH | 220 | GMRPCS | Putative nickel/cobalt efflux system HI_1248 |
| 173 | **Cluster 84** | **P44127** | 103 | YDICERLKQLH | 85 | WLGKRANT | 9 | QGQIYLNTWP | UPF0208 membrane protein |
| 174 |  | **P44542** | 70 | GDDRAMLKQLK | 146 | YNGTRQTT | 56 | QGKIEISLYP | Sialic acid-binding periplasmic protein SiaP |
| 175 | **Cluster 85** | **P44165** | 241 | FPHIMNVKTAGVNLNVPVSVIANRPDVKAAQFRLSSAFKNAKATQKSWFP | 304 | TVGTALHNPVAAGTVGISLPFLNWNTVKWNVKISEADYETARLNYEQRIT | 359 | VDTNYFAFTQAQSTLSNLQQTHSYNQRITQYYRNRYNAGVSELREWLV | No result |
| 176 |  | **P45217** | 254 | FPHIMNVKTAGVNLNVPVSVIANRPDVKAAQFRLSSAFKNAKATQKSWFP | 317 | TVGTALHNPVAAGTVGISLPFLNWNTVKWNVKISEADYETARLNYEQRIT | 372 | VDTNYFAFTQAQSTLSNLQQTHSYNQRITQYYRNRYNAGVSELREWLV | No result |
| 177 | **Cluster 86** | **P44077** | 47 | DDCLADW | 58 | LCEADK | 148 | YHPGNRAVTTP | No result |
| 178 |  | **P44138** | 11 | DDYLYRF | 78 | YCLIFK | 18 | YQYGDTHSKIP | No result |
| 179 | **Cluster 87** | **P44507** | 192 | CDVNNP | 325 | CLREDYD | 99 | VPPEKR | Glycerate kinase |
| 180 |  | **P45074** | 26 | DDVNQP | 106 | ANKVHYD | 72 | RPAEKS | Lipopolysaccharide export system protein LptA |
| 181 | **Cluster 88** | **P44167** | 4 | FRPFYQQ | 77 | RIIHHL | 134 | YHMWRM | tRNA (mo5U34)-methyltransferase |
| 182 |  | **P44478** | 89 | FNPVKYQ | 62 | RVVHHL | 49 | FQPWGS | No result |
| 183 | **Cluster 89** | **P44720** | 315 | DGSGGH | 123 | WRKDLENAPH | 338 | RWYRSQ | UPF0755 protein |
| 184 |  | **P44028** | 15 | SGCGSV | 36 | VAYDLEMAQQ | 65 | PYAWAQ | No result |
| 185 | **Cluster 90** | **P44217** | 44 | CQWQKDE | 66 | TGAYFYFIDDK | 16 | SSHSFA | No result |
| 186 |  | **P44509** | 286 | HSWQGNE | 347 | TGLFFNKIDDK | 124 | SWHRRY | No result |
| 187 | **Cluster 91** | **P44709** | 28 | MAPNFKLFCQM | 82 | VNDYDS | 191 | GIEFHQ | No result |
| 188 |  | **P44176** | 1 | MTKKYDLHCHS | 117 | PNAYDG | 245 | GSDFHF | Protein TrpH |
| 189 | **Cluster 92** | **P44897** | 50 | PQTQCE | 26 | HKAPVD | - | No motif | UPF0352 protein |
| 190 |  | **P44000** | 157 | PQTQNE | 45 | HCDPKG | 140 | EKIWEV | No result |
| 191 |  | **P44056** | - | No motif | - | No motif | 175 | RKIWMR | No result |
| 192 |  | **P44045** | - | No motif | - | No motif | - | No motif | No result |
| 193 | **Cluster 93** | **P44668** | 31 | HKWICE | 39 | NFDDDP | 1 | MKWTDA | Protein IscX |
| 194 |  | **P44726** | 112 | YPGVVE | 34 | NFFRLP | 15 | KDWGDA | UPF0701 protein |
| 195 | **Cluster 94** | **P44606** | 92 | IDLNCGCPSKTVN | 166 | TVHGRT | 195 | IPVIANGEIWHWQDGQDCLSQTGCQDLMVGRGALNIPNLSH | tRNA-dihydrouridine synthase |
| 196 |  | **P44965** | 94 | IDINMGCPAKKVN | 167 | TVHGRT | 195 | IPVIANGDIDSARKAKFVLNYTGADAIMIGRAALGNPWLFQ | tRNA-dihydrouridine synthase B |
| 197 | **Cluster 95** | **P44905** | 139 | GMGGGFYDR | 175 | EHWDVP | 1 | MNTQKR | No result |
| 198 |  | **P44177** | 36 | GQRQAFEDR | 62 | EQWQFP | 1 | MLTQIA | No result |
| 199 | **Cluster 96** | **P44540** | 168 | SGNNHFMY | 59 | CRTIGFKGFSEF | 91 | TEIMPS | Uncharacterized HTH-type transcriptional regulator |
| 200 |  | **P44844** | 30 | SGKKLTCY | 174 | YRTFERMDINQF | 11 | TDIMPI | Der GTPase-activating protein YihI |
| 201 | **Cluster 97** | **Q57065** | 118 | MIVIGMTGLWWW | 10 | SPGEIF | 103 | SSHNRW | No result |
| 202 |  | **P44588** | 28 | MALLGDKLFRQR | 56 | HPGKIF | 15 | YLHTRG | Alternative ribosome-rescue factor A |
| 203 | **Cluster 98** | **P71339** | 31 | VIEFYIQNGKNYSLISKHFQLDSRTLRHWINQFNHSRINGLAVLGKTRNY | 87 | NVIQTVKNG | 1 | MIKARYFYLYKLIVRCRFFIAKYNTLFKQ | No result |
| 204 |  | **Q57066** | 31 | VIEFYLQNDKNSSLTRRHFQLAETTLERWINQFNHSGINGLALLGKKRNY | 87 | NVIQAVKNG | 1 | MICTPKVGLNNQLTKVQFFMTKYNFLFKQ | No result |
| 205 | **Cluster 99** | **P43907** | 98 | FHFDAIPENSTNPNE | 128 | WQVNVMK | 42 | QDFETI | No result |
| 206 |  | **P44693** | 52 | FDSGEYHELNTSPNE | 252 | WQVSLRK | 170 | QQFYWI | Uncharacterized metalloprotease HI_0409 |
| 207 | **Cluster 100** | **P44904** | 19 | HDVMPA | 101 | HQKVCSISTDR | 1 | MNLERLN | UPF0597 protein |
| 208 |  | **P44292** | 199 | FDVLPA | 144 | FFNVPTQWIEN | 102 | PQLERLR | No result |
| 209 | **Cluster 101** | **P45301** | 124 | ISKDSW | 1 | MTLSPL | 138 | MFVDGR | No result |
| 210 |  | **P44033** | 68 | IIKDEH | 1 | MNFCRI | 131 | AFVITR | Putative kinase HI_0665 |
| 211 | **Cluster 102** | **P45252** | 207 | ENPYHIRDRGLV | 226 | PSALKDLEFFV | 64 | DWGFHC | UPF0162 protein |
| 212 |  | **P44506** | 151 | ENLPHLCLRGLM | 209 | PSAIKCGSTMV | 76 | EWHFIG | UPF0001 protein |
| 213 | **Cluster 103** | **P45332** | 176 | YTFDQQHN | 36 | QFRSVGKGTYDIWQ | 121 | WGIPQTEQ | Lipopolysaccharide export system permease protein LptG |
| 214 |  | **P44235** | 113 | DTADQETG | 64 | QIASVGKINGPISM | 98 | WESAKAEQ | Mu-like prophageFluMu protein gp41 |
| 215 | **Cluster 104** | **P44010** | 103 | RNHPDQPE | 9 | PYAKYNEQ | 27 | SRWLQV | UPF0114 protein |
| 216 |  | **P45290** | 53 | PQFSSQVE | 115 | RYLKMDKQ | 175 | YTWSQH | UPF0324 membrane protein |
| 217 | **Cluster 105** | **P43908** | 123 | LMQPYR | 216 | CRYIIQHH | 38 | CRKLSP | UPF0246 protein |
| 218 |  | **P44203** | 3 | LMWALR | 129 | NYFIRQYT | 39 | CATVPM | No result |
| 219 | **Cluster 106** | **P44278** | 168 | NPRYFW | 106 | KKDERNLWND | 133 | SDRTFD | No result |
| 220 |  | **P44796** | 149 | SPDYLR | 112 | KEQEIHYARD | 47 | PQRIED | High frequency lysogenization protein HflD homolog |
| 221 | **Cluster 107** | **P45253** | 253 | HGWQQG | 261 | VRSIFQE | 86 | NVSKGT | Release factor glutamine methyltransferase |
| 222 |  | **P45076** | 104 | HNQQQA | 164 | YREIYQM | 22 | WVSKSE | UPF0307 protein |
| 223 | **Cluster 108** | **P45122** | 203 | NYQELTSEEQDKQPN | 58 | WVKHPE | 193 | IHFEWG | UPF0126 membrane protein |
| 224 |  | **P43975** | 347 | NLWDISSEEMMKYTR | 88 | WRNWET | 21 | SHYRWT | No result |
| 225 | **Cluster 109** | **P44940** | 133 | QWQWLEQ | 245 | ARHITT | 93 | WRNQEPY | No result |
| 226 |  | **P44164** | 34 | QGQWLKQ | 20 | ARHLTV | 83 | WEGITPY | Phosphohistidine phosphatase SixA homolog |
| 227 | **Cluster 110** | **P44209** | 146 | YVRVSFEYWH | 49 | ENWHKQ | 10 | MVRQPVK | No result |
| 228 |  | **P44099** | 347 | YVQVAFEIPE | 101 | EGWQKI | 184 | MADEPLK | No result |
| 229 | **Cluster 111** | P44740 | 207 | RIYGMS | 53 | QFSHLW | 68 | QGKWQP | UPF0066 protein |
| 230 |  | P44863 | 139 | MTYLMT | 237 | GMGNLW |  | 82 | HMPMQP |
| 231 | **Cluster 112** | **Q57060** | 58 | KQFGCH | 202 | GMIYDEG | 23 | GKKATDW | No result |
| 232 |  | **P43936** | 21 | FSHTCH | 106 | YHIYDKG | 85 | YDKRTRW | No result |
| 233 | **Cluster 113** | **P71373** | 209 | DHSECRGAFNFAAPKSIKQH | 284 | DCENYL | 268 | VVPEKLLNAGFQFQY | Epimerase family protein HI_1208 |
| 234 |  | **P43961** | 111 | FYDEFWGQGLRAAPKKQKKH | 98 | NCANYH | 80 | VYPEPKRYARSVRQY | No result |
| 235 | **Cluster 114** | **Q57152** | 51 | WVFIPRM | 72 | AISPYI | 38 | FSIDTM | No result |
| 236 |  | **P45267** | 335 | RVYFERM | 14 | AISPQI | 253 | FSQDFM | No result |
| 237 | **Cluster 115** | **P44104** | 305 | NDWYVM | 62 | FYYTHDEQSR | 249 | AGCLKI | No result |
| 238 |  | **P43997** | 80 | NDWYLG | 131 | CWEKVDSQSM | 17 | AGCSSW | No result |
| 239 | **Cluster 116** | **P43960** | 45 | CDCDKPCDTH | 78 | DDSQFYQV | 59 | DAGDEQ | No result |
| 240 |  | **P44067** | 135 | YEKPFPETMH | 294 | DEFRHKQV | 271 | DMWENA | No result |
| 241 | **Cluster 117** | **P44103** | 174 | LIHHWF | 189 | VLGCGDG | 263 | GGQHCRAEFYLAGFGWV | No result |
| 242 |  | **P44553** | 93 | SFLHQF | 16 | VIGCSSG | 175 | RHELEIAKFYAKRKAWV | Outer membrane protein assembly factor BamD |
| 243 | **Cluster 118** | **P44717** | 262 | GYIESH | 199 | TQEHYL | 405 | YGKYKF | UPF0053 protein |
| 244 |  | **P44854** | 91 | GKLESH | 12 | AQKHTL | 31 | YTFYKG | No result |
| 245 | **Cluster 119** | **P44023** | 99 | VEQHHK | 278 | REEFRQ | 257 | YLYWYC | No result |
| 246 |  | **P45280** | 185 | HTQIQK | 116 | REKFSQ | 28 | YGYWAV | No result |
| 247 | **Cluster 120** | **P44240** | 211 | WCFERY | 47 | HMHLDW | 228 | CDEREDILPTPEDIARVRAYIEGHK | Mu-like prophageFluMu protein gp47 |
| 248 |  | **P44275** | 25 | YVFAQY | 94 | EGHRAS | 109 | NQSGADLMLLREDIAHLKDKIYLHD | No result |
| 249 | **Cluster 121** | **P44226** | 148 | PNDWGCRC | 76 | QLGSPR | 373 | PNKHSIKPKEKVDAIINAYKVDM | Mu-like prophageFluMu F protein |
| 250 |  | **P44013** | 105 | CLDWHCYR | 54 | QLGTPF | 15 | PFYQFAQMKKFCPEDIPAIKADY | No result |
| 251 | **Cluster 122** | **P44214** | 49 | WLGDETSIFGIVCS | 18 | IHECLT | 63 | YDNPARDEAV | No result |
| 252 |  | **P44158** | 32 | MLGFITALFITACS | 168 | QWICLN | 118 | YDFGLRDEAV | No result |
| 253 | **Cluster 123** | **Q57380** | 40 | PMCWKP | 74 | KTETLPM | 15 | FESGYRF | No result |
| 254 |  | **P44545** | 277 | IMILKP | 175 | KQINLPD | 48 | YEPGLHF | Protein HflC |
| 255 | **Cluster 124** | **P44683** | 12 | PEHITPEIFLRDYW | 148 | GHRRWQ | 339 | IYANGE | Probable ribosomal oxygenase HI_0396 |
| 256 |  | **P44239** | 120 | PDGSTEQRTFRANW | 44 | GERGWW | 7 | TWHNGE | Mu-like prophageFluMu protein gp46 |
| 257 | **Cluster 125** | **P44144** | 53 | DCPQCF | 382 | YACRIIVPGM | 223 | SVMDRY | No result |
| 258 |  | **P44150** | 95 | DCLAQF | 12 | YQQHLIACNH | 159 | SVTQRH | No result |
| 259 | **Cluster 126** | **Q57544** | 13 | QNCSLIWDDEKN | 104 | FLPDRWF | 60 | HVGAAM | No result |
| 260 |  | **P44743** | 32 | QGCKLNCLYCHN | 112 | FEFDRVC | 65 | QVMEAV | No result |
| 261 | **Cluster 127** | **P44081** | 123 | AYFRPH | 43 | HYVERT | 90 | YFLWCER | No result |
| 262 |  | **P43972** | 48 | VYSCNK | 22 | DAPEMT | 99 | DYVWNVD | No result |
| 263 | **Cluster 128** | Q57151 | 99 | CPNVHIM | 71 | WGGSAI | 178 | DYFHAQ | Putative hydroxypyruvate isomerase |
| 264 |  | P44131 | 92 | QDLVYRM | 13 | QAGMAI | 54 | DLMHSG | No result |
| 265 | **Cluster 129** | **P44247** | 1 | MDTSDNLARVGIRCSVRDLAPMVGNVPHFEQQQADYYNLFNLRRRKQPIQ | 66 | SRGLTSAPLLGETLASIIYGEPLPISEGILHNLSANRAWVKKWLKGSKVE | 51 | SAANFQNLFLIAALG | No result |
| 266 |  | **P44246** | 556 | VDTSDNLARVGIRCSVRDLAPMVGNVPHFEQQQADYYNLFNLRRRKQPIQ | 621 | SRGLTSAPLLGETLASIIYGEPLPISEGILHNLSANRAWVKKWLKGSKVE | 606 | SAANFQNLFLIAALG | tRNA 5-methylaminomethyl-2-thiouridine biosynthesis bifunctional protein MnmC |
| 267 | **Cluster 130** | **P44168** | 87 | NLPDFHW | 137 | RDVIDWF | 71 | SERFNIETDCTK | UPF0260 protein |
| 268 |  | **P44014** | 64 | NIPMFFP | 127 | AGVTSWA | 24 | SEGFYFITICCK | No result |
| 269 | Cluster 131 | P43965 | 106 | GIWQRRFW | 57 | PDHIHLLM | 146 | WPYSSF | No result |
| 270 |  | P44095 | 128 | GIWFPCTW | 21 | PNHCGTHM | 11 | TPFSSF | No result |
| 271 | Cluster 132 | P44068 | 109 | YDRQNWFDRAKGMEW | 208 | FYQQEYCGCVYSLRDS | 168 | QINGCGHR | No result |
| 272 |  | P44552 | 228 | YRRDNQTGRMASVIW | 65 | FLTQTHSTRVIQLPYS | 210 | QIYGGNHC | Laccase domain protein HI_0175 |
| 273 | **Cluster 133** | **P44675** | 96 | ENCKNGVECLTH | 1 | MKLTSK | 65 | YQLGLP | Putative HTH-type transcriptional regulator HI_0379 |
| 274 |  | **P44908** | 108 | ESKFQGFESLTH | 1 | MKLTSP | 180 | YQSADP | No result |
| 275 | **Cluster 134** | **P43940** | 80 | PIYSFED | 88 | KTYIPK | 126 | LHLYIMSQM | No result |
| 276 |  | **P44296** | 231 | QLYTFED | 56 | KYTHPK | 252 | LDKETIKQM | No result |
| 277 | **Cluster 135** | **Q4QKT3** | 5 | CDQPIC | 71 | RIEPLG | 13 | YEECEP | No result |
| 278 |  | **Q57133** | 140 | RDLKVC | 176 | RNMPVG | 31 | QSAHTP | No result |
| 279 | **Cluster 136** | **P45103** | 25 | QKGGVIVYPTDSGYALGCMMGDKHAMDRI | 4 | FFYIHPEN | 174 | HGGYLG | No result |
| 280 |  | **P44215** | 98 | QTGQKIEPLTNHNYLKSVYETQKPHFAVI | 164 | FVKNSPEY | 42 | MVGYLG | No result |
| 281 | **Cluster 137** | **P44500** | 114 | WAKQCHFFESQLYLAKQFNLPVNIHSRKTHDQIFTFLKRIPLSKLGVVHG | 1 | MHFFDTHTH | 210 | ETDSPDMPVFGFQGQPNRPERIVESFKALCTLRNEPAELIKKLTWEN | Uncharacterized deoxyribonuclease HI_0081 |
| 282 |  | **P44718** | 107 | KAAQQAVFGSQIDIANQLDKPVIIHTRSAGDDTIAMLRQHRAEKCGGVIH | 1 | MFIVDSHCH | 204 | ETDSPYLAPVPYRGKENQPAYTREVCEYVATLKGVSAEAFAQITTQN | Uncharacterized deoxyribonuclease HI_0454 |
| 283 | **Cluster 138** | **P44869** | 52 | IHQSEC | 9 | AKGEVRIIAGLWRGR | 148 | CENNWL | Ribosomal RNA small subunit methyltransferase D |
| 284 |  | **P43790** | 136 | MHSSEH | 61 | MKGKVQIDVDLECQR | 86 | CEFTYS | No result |
| 285 | **Cluster 140** | **P44670** | 19 | SQWIDNARNHCEVIKKDQSSAER | 10 | PEHSAW | 82 | PRGLMDW | No result |
| 286 |  | **Q57354** | 54 | SQNADAILVHHGYFWKSETPCIR | 203 | TIHSAR | 140 | PMTGKDF | UPF0135 protein |
| 287 | **Cluster 140** | **P44702** | 79 | NINNSVWK | 138 | HLNWLEW | 10 | HINQNSCAMKVGT | tRNA1(Val) (adenine(37)-N6)-methyltransferase |
| 288 |  | **P44126** | 59 | NDNDGVVK | 149 | HQAPREW | 116 | HVDCASIMTGYGT | UPF0115 protein |
| 289 | **Cluster 141** | **P52606** | 97 | ELYCHQ | 32 | QMVMQC | 1 | MLQKVK | No result |
| 290 |  | **Q57256** | 139 | YLTCHL | 152 | LMVGIC | 1 | MKKKVR | No result |
| 291 | **Cluster 142** | **P45097** | 30 | GMPSVFVR | 3 | MEKYHD | 45 | CPWCDT | 7-carboxy-7-deazaguanine synthase |
| 292 |  | **P43929** | 15 | GMPAVFTA | 27 | VEKYHG | 244 | GWECDS | No result |
| 293 | **Cluster 143** | **P43954** | 150 | CWCLREAV | 90 | HSGDWV | 1 | MTTYIAY | Putative 4'-phosphopantetheinyl transferase HI_0152 |
| 294 |  | **P44140** | 335 | FPCLRGKV | 584 | DDFDWM | 256 | STTIHSY | tRNA(Met) cytidineacetyltransferaseTmcA |
| 295 | Cluster 144 | P44069 | 125 | CALDNK | 33 | EFPISM | 78 | SVEMAYAY | No result |
| 296 |  | P71379 | 64 | PILDNW | 99 | EQPIKR | 49 | WHETAKKC | Putative csd-like protein |
| 297 | **Cluster 145** | **P44882** | 135 | CQLGYD | 158 | YVRTIAMLFYSHFNEGEIESKP | 62 | PVTELY | UPF0149 protein |
| 298 |  | **P44646** | 221 | CRVQYA | 167 | MKHKFAMVISSTPHDVAKEERH | 332 | PNTAKY | UPF0118 membrane protein |
| 299 | **Cluster 146** | **P44520** | 113 | KYNRWL | 137 | DWIICG | 1 | MEHEYQ | No result |
| 300 |  | **O86226** | 63 | RYTRYN | 104 | GWLTEG | 96 | GEQDTS | No result |
| 301 | **Cluster 147** | **P43984** | 165 | MSHLFP | 137 | CQAKYTALVGLP | 38 | GKRNST | No result |
| 302 |  | **P44255** | 1 | MEHKLE | 137 | EQVKIYAEKGLP | 123 | GNPGDT | Elongation factor P hydroxylase |
| 303 | **Cluster 148** | **P44070** | 101 | GRLDRE | 70 | RHHKLGNIVW | 204 | NPLMPEY | UPF0721 transmembrane protein |
| 304 |  | **P44054** | 124 | SRIQIE | 72 | KIDYINMILW | 241 | NPVLTRK | UPF0721 transmembrane protein |
| 305 | Cluster 149 | Q57097 | 46 | SWVVEA | 20 | GRLYTP | 215 | FPQMTEGC | No result |
| 306 |  | P44807 | 87 | TWIVPA | 163 | GRAHNP | 155 | FPVLNEMV | tRNAthreonylcarbamoyladenosine biosynthesis protein RimN |
| 307 | **Cluster 150** | **P43980** | 12 | MPHLDD | 153 | QILFDMP | 26 | MCEHNEQ | UPF0301 protein |
| 308 |  | **P44242** | 1 | MYHLDN | 547 | QWQFRLP | 501 | VCEFSSQ | Mu-like prophageFluMu defective tail fiber protein |
| 309 | Cluster 151 | P44754 | 13 | WAARFYKT | 103 | SLSMPH | 47 | EVGAML | Heat shock protein 15 homolog |
| 310 |  | P44931 | 108 | FGASDYKT | 127 | DYKMNH | 89 | EPCTMC | tRNA-specific adenosine deaminase |
| 311 | Cluster 152 | P46494 | 76 | FGMFIGCSHYPECDFVV | 1 | MNQSLFHH | 115 | RRGRQGKIFY | No result |
| 312 |  | P45279 | 18 | VGLFIGYIIVRLTKGSV | 75 | DYQKLYRH | 1 | MENWTNEIWV | No result |
| 313 | Cluster 153 | P44256 | 21 | FMRAKG | 31 | LIGLHV | 42 | NKQEQMSLW | No result |
| 314 |  | P44761 | 87 | FTRAKG | 30 | LIGEHC | 176 | NRQIVLSLY | No result |
| 315 | **Cluster 154** | **P44251** | 186 | YWALRCAVQEIEGEHYVFSRFTHW | 31 | ILPENH | 40 | ICYDYQ | No result |
| 316 |  | **P44038** | 354 | YWNNAHGWQYFGGLRMRYDSFTQA | 172 | ISPETH | 504 | LEYQYQ | Translocation and assembly module TamA |
| 317 | **Cluster 155** | **P43982** | 49 | GTHIQVN | 1 | MKKITL | 30 | CEQLFK | No result |
| 318 |  | **P44063** | 197 | GSVIQTN | 1 | MDKIAL | 136 | DRQLFK | No result |
| 319 | Cluster 156 | P44938 | 130 | TLNIAANYGGCWD | 159 | MSVSDINNSTFQHHLATQNAPPVDL | 20 | NGRWAKQKNKMRIFGHTNG | Ditrans,polycis-undecaprenyl-diphosphate synthase ((2E,6E)-farnesyl-diphosphate specific) |
| 320 |  | P44085 | 6 | TLGMAALLSGCSM | 116 | TRVSNVEPLTKQSCLKLRNEARDDL | 149 | NGMVEVQKNEDGTPKNSDG | No result |
| 321 | Cluster 157 | P44262 | 48 | EQSAKNW | 126 | EWINRVNMYF | 126 | ERWHPM | No result |
| 322 |  | O86222 | 69 | EQSRLYW | 85 | EWLGVDYDYF | 107 | EGWEHI | No result |
| 323 | Cluster 158 | P44593 | 1 | MKYQLNLTALRC | 64 | EKEFIV | 55 | ALVEQY | UPF0033 protein |
| 324 |  | P44294 | 1 | MKGQLNLRSETT | 20 | EVAFIT | 34 | GLIEGY | No result |
| 325 | **Cluster 159** | **P31777** | 142 | CDNGFQQV | 5 | HHSFHA | 231 | SDQRGMTASGM | Ribosomal RNA large subunit methyltransferase J |
| 326 |  | **P44117** | 5 | NKQSFQDV | 101 | QKKAHA | 88 | SKQRREASTKM | UPF0265 protein |
| 327 | Cluster 160 | P44036 | 75 | QRYWQI | 103 | KDFVHF | 21 | YRQAYG | No result |
| 328 |  | P45244 | 43 | HPYHFI | 178 | KDFVTY | 113 | GCATYG | Putative NAD(P)H nitroreductase HI_1542 |
| 329 | **Cluster 161** | **P44679** | 96 | RLMRNT | 31 | FFERAR | 19 | AGGVVYHARY |  |
| 330 |  | **P45305** | 196 | RLMIET | 209 | GFERAG | 87 | AIGEVGLPHY | Uncharacterized deoxyribonuclease HI_1664 |
| 331 | Cluster 162 | P44094 | 149 | MCELLINDYSRKGFVDGIVVRLPTICIRPGKPNKAASSFVSSIMREPLHG | 55 | CPVSEE | 291 | QALALGFKV | No result |
| 332 |  | P43939 | 1 | MSELLINDYTRKGFVDGLCLRLPTICIRPGKPNKATSSFVSSIIREPLHG | 203 | CPVAEK | 76 | WALAITGYV | No result |
| 333 | Cluster 163 | P44079 | 92 | PINCPKEVYAH | 117 | HHIYPKE | 57 | NSSEVW | No result |
| 334 |  | P44135 | 618 | PDAMKKEALAH | 124 | YLIYPKE | 79 | HSSKVW | No result |
| 335 | **Cluster 164** | **P44839** | 101 | HPNFPARSC | 84 | DFAAVN | 78 | FVKDLN | RutC family protein HI_0719 |
| 336 |  | **P43935** | 218 | HVYIGNHWY | 201 | DFAAVN | 121 | FVKDWL | No result |
| 337 | **Cluster 165** | **P43989** | 36 | MFGWRYW | 194 | QQMAKM | 171 | AKGDKV | UPF0070 protein |
| 338 |  | **P45197** | 31 | VFGKRHD | 73 | QPMYQM | 148 | WKGDKP | No result |
| 339 | **Cluster 166** | **P44609** | 12 | NCPCQSSHHYAD | 124 | KIEDRW | 42 | MRSRYTAY | UPF0225 protein |
| 340 |  | **P44078** | 83 | NVDSTSNHHEAR | 95 | KLEICW | 1 | MKSHRTLY | No result |
| 341 | **Cluster 167** | **P44684** | 128 | KNLTHC | 82 | RIGAAY | 191 | IDNGIA | ADP-ribose pyrophosphatase |
| 342 |  | **P44954** | 65 | KNKTEF | 23 | REGTDY | 46 | IRNGIA | UPF0270 protein |
| 343 | **Cluster 168** | **Q57523** | 103 | DFGCLFSREVC | 22 | HWVRKAVCI | 734 | HCWQNT | Translocation and assembly module TamB |
| 344 |  | **Q57392** | 41 | DLGYRYNDWGR | 60 | HEASFGVRY | 1 | KADQET | No result |
| 345 | **Cluster 169** | **P44290** | 200 | YWFQQADK | 100 | IRNLDS | 111 | QMPNIT | UPF0319 protein |
| 346 |  | **P44062** | 81 | TGIQQLDH | 31 | ARNLDI | 40 | EMKEKT | Cell division protein ZapA |
| 347 | **Cluster 170** | **P44641** | 120 | GCAVNC | 236 | IFAHAM | 88 | GFSTDP | L-lysine 2,3-aminomutase |
| 348 |  | **P44254** | 37 | GCDKYR | 25 | YFGDAC | 1 | MLSKDP | No result |
| 349 | **Cluster 171** | **P44298** | 188 | SQYYWW | 222 | EMLSHAIQFGSVQVPPS | 254 | YPKIAN | No result |
| 350 |  | **P44634** | 171 | EIYTAW | 100 | ECLSDNANRTISQVRPS | 4 | HSKWAN | Probable transcriptional regulatory protein HI_0315 |
| 351 | **Cluster 172** | **P44231** | 63 | WLGQQP | 165 | IYDHTFNQDKDEH | 116 | PSGMFE | Mu-like prophageFluMu protein gp37 |
| 352 |  | **P44227** | 49 | WLGQMP | 130 | CYDGQYFFDTDHP | 28 | PSQYTE | Mu-like prophageFluMu major head subunit |
| 353 | **Cluster 173** | **P45182** | 770 | SYHYGRHTQWDSSIRW | 431 | IQDNLHF | 46 | LMLLFC | No result |
| 354 |  | **P44171** | 51 | CYAVPREIAQKTSYSW | 32 | IQQQLHF | 4 | LMAIFS | No result |
| 355 | **Cluster 174** | **P44098** | 79 | CIKQYN | 196 | WMIAHC | 14 | IKVMCQ | Putative glutamine amidotransferase HI_1037 |
| 356 |  | **P44771** | 106 | CMNSYQ | 228 | WAGKGC | 121 | IPAMRQ | No result |
| 357 | Cluster 175 | P44183 | 20 | QTYYAR | 45 | IANKVC | 133 | WKISPTGQR | No result |
| 358 |  | **P44106** | 86 | YTKYAN | 112 | FPFKDC | 183 | HQINPKKTR | No result |
| 359 | **Cluster 176** | **P44037** | 45 | CREDNDY | 66 | CMTNNHQ | 22 | YELYQY | No result |
| 360 |  | **P44196** | 207 | HREPQGF | 60 | PMEIIHQ | 41 | YGDYPY | No result |
| 361 | **Cluster 177** | **P44084** | 71 | HLGVTQIW | 9 | ASFMLVAC | 64 | YHLYWY | No result |
| 362 |  | **P24324** | 142 | HYGVNIYC | 71 | GVYMCAVA | 208 | FRLIWD | No result |
| 363 | Cluster 178 | P44289 | 517 | WFAVSYLWPDW | 619 | YRDRMKN | 492 | DVMGFD | No result |
| 364 |  | P44137 | 116 | YFDVPLAHPQK | 97 | YSPRPKN | 73 | DVMGNI | No result |
| 365 | **Cluster 179** | **P44124** | 202 | GCRKCPS | 132 | TDFSGYPDCR | 186 | DYVQKHTHT | 7-cyano-7-deazaguanine synthase |
| 366 |  | **P43971** | 147 | MGRECLS | 22 | CHSKVKRFCR | 38 | DYLEKHGYT | No result |
| 367 | **Cluster 180** | **P44019** | 74 | MKYTQCDTH | 114 | PSAAGC | 20 | LIKKAE | Putative uncharacterized transporter HI_0586 |
| 368 |  | **P44691** | 71 | MVWLESNTQ | 241 | PSVVIC | 164 | NIKKMR | High-affinity zinc uptake system membrane protein ZnuB |
| 369 | Cluster 181 | P44018 | 49 | FKDYGEQHRQAY | 176 | ESNEFVR | 140 | MGNSYAN | Putative uncharacterized transporter HI_0585 |
| 370 |  | **P45019** | 38 | WIDTGLEYQMFH | 62 | RDNKFAR | 1 | MKNKYLT | UPF0382 membrane protein |
| 371 | Cluster 182 | P44253 | 98 | YHTNQP | 80 | KTYGQTMAKRW | 14 | ENQKDAE | No result |
| 372 |  | O05087 | 172 | YHSFIP | 300 | CPTIERNRNRW | 252 | NPKNPAE | Uncharacterized membrane protein HI_1728 |
| 373 | **Cluster 183** | **P44279** | 156 | ACRKAGVIFLEFQPNY | 3 | VSMGEF | 32 | NKNDHL | No result |
| 374 |  | **P71375** | 85 | AWTVPTVIPLCFLALY | 97 | VDMGEQ | 50 | TKGDDL | Putative uncharacterized symporter HI_1315 |
| 375 | **Cluster 184** | **P45333** | 226 | HFDEYHAY | 265 | ELHWRI | 177 | MTPQKQ | Lipopolysaccharide export system permease protein LptF |
| 376 |  | **P44110** | 45 | HIPLLEKY | 82 | FLSWKM | 1 | MTLQLN | UPF0756 membrane protein |
| 377 | **Cluster 185** | **P44577** | 102 | EWHKENGTY | 17 | RIVAPMQR | 203 | ECANRILARKK | No result |
| 378 |  | **P44238** | 50 | ERFQNYGHY | 6 | RIIAPLKR | 112 | ENGEAILSCKK | Mu-like prophageFluMu protein gp45 |
| 379 | **Cluster 186** | **P44012** | 236 | WMKQHNP | 112 | SWSPGFCD | 196 | WEKHGSC | Probable ribonuclease HI_0526 |
| 380 |  | **P44005** | 81 | WMPKFDP | 110 | SWLPVVGD | 12 | WQANSLC | No result |
| 381 | **Cluster 187** | **P44782** | 152 | VKLTPVTGRSHQLRLHMLALGHPILGDKFY | 56 | FCEPAHRLDMATSGIIVFALSKAADRELKRQFREREPKKHYQAIVWGH | 18 | YQDNHLCVVNKPSG | Ribosomal large subunit pseudouridine synthase A |
| 382 |  | **P44197** | 152 | VKLIPHTGRKHXLRXHMKHVFHPIXGDTQY | 46 | HVFPIHRLDRPTSGVLLFALSSEIANLMCEQFEQKYVQKSYLAVVRGY | 6 | YQDGFLVAVNKPAG | tRNApseudouridine synthase C |
| 383 | Cluster 188 | Blast search sequence |  |  |  |  |  |  | No result |
| 384 |  | **P44267** | 46 | FGFIDRCFVEAPLHIEPEYWKNAKENFVQNGNAHSPYFQDIAGEFLLFCQ | 222 | QVWMKW | 33 | RLAVYTRLVRN | No result |
| 385 | Cluster 189 | P44181 | 27 | PDGFTG | 81 | RNDTVW | 37 | TVSYHF | No result |
| 386 |  | P44161 | 79 | KRFFNG | 96 | LEYAVW | 118 | TITYMI | MacrodomainTer protein |
| 387 | Cluster 190 | P44059 | 20 | CRFPHLDEM | 1 | MVPLCEGDLWQE | 30 | NIASINGRK | No result |
| 388 |  | P44202 | 23 | CIFPIIPIY | 156 | MMFVYVLGLATP | 68 | NILFSNTTR | Putative cytochrome c-type biogenesis protein HI_1454 |
| 389 | **Cluster 191** | **P43990** | 194 | PNALYM | 174 | GWAAYS | 145 | WREIIS | No result |
| 390 |  | **P44831** | 25 | PNALYI | 107 | GWGTYF | 73 | CFDIIS | Regulator of ribonuclease activity B |
| 391 | **Cluster 192** | **Q57320** | 92 | YAKFESHSDTEFNQQT | 32 | RRNTVC | 141 | ITEMWQ | Uncharacterized membrane protein HI_1307 |
| 392 |  | **O86220** | 53 | YVKPPLILVTLKAQQN | 5 | PPPLLC | 45 | LSSVMQ | No result |
| 393 | **Cluster 193** | **P44189** | 139 | KFSFEFTEYELQQLVWLWFAFMRGIVTFQHIEKAFKALGSNMSGDIYGQA | 46 | DVCHILGYTNSRKALQDHC | 127 | QPQQLALPEPE | No result |
| 394 |  | **P44193** | 120 | KFTHEFTEFEIETLVWLLIGHHQMNTLLGQLEKPLDAIGSNLHPAVYSYW | 55 | DYLVITERTNGRPRKEYHI | 109 | QPQQLALPEPE | No result |
| 395 | **Cluster 194** | **P44187** | 157 | WYWKCY | 34 | CVNKMQ | 46 | QCGHES | No result |
| 396 |  | **P45180** | 496 | SEWRCD | 363 | CHTLMS | 136 | ACFMDS | Glycogen phosphorylase |
| 397 | **Cluster 195** | **P44134** | 153 | KVIFTHKPYPAFKSAYYIKGFEKQNQVGDIFEFSGWNGKKYYDQFDYVKW | 88 | KSYPVGKLADLEIHFMHYHSEQEANEKWQLRTSRMKLDNLFIMMTDRD | 9 | SAVNKRQRFFINRTLQRKLTNQGMTVISANCVGAFILHDLHQPFNSPFVN | No result |
| 398 |  | **Blast sequence** |  |  |  |  |  |  | No result |
| 399 | **Cluster 196** | **Q57498** | 1 | MFTDWK | 59 | TRCESC | 21 | KQYPKM | No result |
| 400 |  | **P44268** | 249 | KGTVWD | 99 | CECEGH | 35 | ENWSKM | No result |
| 401 | **Cluster 197** | **P44050** | 175 | MKMGVKLV | 69 | WGSEWQ | 101 | IWMPPNR | No result |
| 402 |  | **P44175** | 1 | MKMKSLFV | 92 | WLKEMQ | 142 | IWLAPNS | No result |
| 403 | **Cluster 198** | **P44812** | 57 | NWQEHI | 14 | KQAVET | 34 | NAESQR | Cell division protein ZapB |
| 404 |  | **P43992** | 148 | GDQDHL | 56 | TRFVET | 8 | TPECQG | No result |
| 405 | **Cluster 199** | **Q57252** | 923 | YLFPHC | 796 | PYTSYY | 657 | WRGNYDFSHEVKAAMDTCLACKACASQC | No result |
| 406 |  | P44162 | 189 | YYQTHC | 74 | PKVSYY | 1 | MMVNDDFQEYVKQLVTKHRDERIYPFQY | No result |
| 407 | **Cluster 200** | **P44086** | 1 | MQNSSNIFTTDKAA | 18 | SFPDLKNFRYNDRTF | 1 | MQNSSN | No result |
| 408 |  | **P44052** | 70 | NVYSTNIFAIHYVA | 121 | SFATKYCSHHNPIAF | 31 | ADIYDR | No result |
| 409 | **Cluster 201** | **P44058** | 62 | SSVDDYYARFCQ | 255 | LMPFHNALTP | 47 | NLQQKW | No result |
| 410 |  | **P44269** | 28 | SSSTDNTATPCV | 7 | LTALAGALTM | 65 | SAEGKC | No result |
| 411 | **Cluster 202** | **Q57493** | 370 | DHMPHGSF | 387 | MDIKERLKLIPYES | 260 | CMGKLRNINSY | Uncharacterized transporter HI_0092 |
| 412 |  | **P44640** | - | No motif | 219 | TYRKPREYNINVEE | 417 | MDGKHDHIWDS | Uncharacterized membrane protein HI_0325 |
| 413 | **Cluster 203** | **P44016** | 254 | MVHSFRMLKGGQEASEHRID | 64 | YWQDFPFWQTMLIC | 28 | FKDSSILENN | Putative oligopeptide transporter HI_0561 |
| 414 |  | **P44530** | 1 | MPTLEKTFELKQRGSTVRQE | 370 | DFDDFVGAMSGLIC | 222 | FKMPTFGENS | Putative permease HI_0125 |
| 415 | **Cluster 204** | **Blast sequence** |  |  |  |  |  |  | No result |
| 416 |  | **P44288** | 26 | RISPFWLLPFIALCIGAILFFQIVKERGTSITITFTNGSGIVADKTQIRY | 789 | DRVLVHIAIMPKYSHLVRQNTEFWIASGYDFSLGWKGAVFNTGSVQQLLK | 110 | RENTKFWLVQPNVSLAGISGLDSLVSGNYITLQPGDGDREDEFIAEEQGP | No result |
| 417 | **Cluster 205** | **P44903** | 449 | MKLHKHDGDHLRK | 358 | CNLMWY | 3 | TESNAYR | Probable transport protein HsrA |
| 418 |  | **Q57048** | 171 | EKSPKKAGRYLMM | 327 | NTLVWY | 144 | TPSNTAR | Uncharacterized transporter HI_0020 |
| 419 | **Cluster 206** | **P44543** | 379 | NYRIAKKRGYPRTPKATREQLCSSFKQSFW | 235 | EKLVYSLDSFPLLAVPFYILTGILMNTGGITERIFNFAKALLGHYTGG | 446 | YKELTLKS | Sialic acid TRAP transporter permease protein SiaT |
| 420 |  | **P44993** | 189 | WRWQAKRLNLMTFSKATKQDLCFSFKNSVW | 45 | QQLVSGADSFSLMAIPFFILAGEIMNEGGLSKRIIDLPMKLVGHKRGG | 256 | YHELPLKH | Putative TRAP transporter large permease protein HI_1029 |
| 421 | **Cluster 207** | **P44472** | 53 | KLDAHT | 369 | QMLPVF | 187 | DEAARF | Putative transport protein HI_0035 |
| 422 |  | **P44163** | 14 | ILAACT | 1 | MMKKIF | 40 | GSGIRF | No result |
| 423 | **Cluster 208** | **Blast sequence** |  |  |  |  |  |  | No result |
| 424 |  | **P71378** | 22 | GCFDKQEAKQKVEDTKQTVASVASETKDAAANTMTEVKEKAQQLSTDVKN | 5 | MKKLTLALVLGSAL | 77 | VEDAKEVIKSAT | No result |
