# Supplementary material for: Functional Annotation of Conserved Hypothetical Proteins from Haemophilus influenzae Rd KW20
Source: PLoS One. 2013 Dec 31;8(12):e84263. doi: 10.1371/journal.pone.0084263 (PMC3877243; doi:10.1371/journal.pone.0084263)
Supplement: Table S9 — List of annotated HPs at low confidence from H. influenzae. (DOCX) [file pone.0084263.s009.docx]

| **S.NO**  Table S9: List of less precisely functionally annotated HPs from *H. influenzae* | **PROTEIN NAME** | **GENE ID** | **UNIPROT ID** | **Protein Function** |
| --- | --- | --- | --- | --- |
|  | HP HI0028 | [950924](http://www.ncbi.nlm.nih.gov/gene/950924) | P44465 | Metallo-hydrolase/oxidoreductase |
|  | HP HI0040 | 950937 | P43929 | Regulator of ribonuclease activity B -like domain family protein |
|  | HP HI0043 | [950940](http://www.ncbi.nlm.nih.gov/gene/950940) | P43931 | Calcium ATPase, transmembrane domain M family |
|  | HP HI0045 | [950943](http://www.ncbi.nlm.nih.gov/gene/950943) | P44478 | YtfJ transcriptional regulator protein |
|  | HP HI0083 | [950981](http://www.ncbi.nlm.nih.gov/gene/950981) | P43938 | pentatricopeptide repeat-containing protein |
|  | HP HI0096 | [951000](http://www.ncbi.nlm.nih.gov/gene/951000) | P43940 | Methyltransferase |
|  | HP HI0108 | [951010](http://www.ncbi.nlm.nih.gov/gene/951010) | P44520 | Phosphopantetheine attachment site |
|  | HP HI0109 | [951013](http://www.ncbi.nlm.nih.gov/gene/951013) | P43943 | Lipopolysaccharide biosynthesis protein -like domain family |
|  | HP HI0120 | [951028](http://www.ncbi.nlm.nih.gov/gene/951028) | **P43947** | Arabinose efflux permease |
|  | HP HI0148.1 | [951051](http://www.ncbi.nlm.nih.gov/gene/951051) | O86220 | protein-S-isoprenylcysteinemethyltransferase |
|  | HP HI0159 | 951069 | P43790 | putative metal/nucleic acid-binding protein |
|  | HP HI0173 | [951082](http://www.ncbi.nlm.nih.gov/gene/951082) | P43960 | Na(+)-translocating NADH-quinone reductase subunit E |
|  | HP HI0198 | [951110](http://www.ncbi.nlm.nih.gov/gene/951110) | P46490 | Sulfite exporter TauE/SafE |
|  | HP HI0205 | [951114](http://www.ncbi.nlm.nih.gov/gene/951114) | P43963 | Shikimate kinase |
|  | HP HI0219 | [951126](http://www.ncbi.nlm.nih.gov/gene/951126) | P44577 | Vascular endothelial growth factor receptor 3 like |
|  | HP HI0227 | [951142](http://www.ncbi.nlm.nih.gov/gene/951142) | P44583 | Clavaminate synthase-like |
|  | HP HI0234 | [951146](http://www.ncbi.nlm.nih.gov/gene/951146) | P43968 | dithiobiotin synthetase |
|  | HP  HI0235 | [951152](http://www.ncbi.nlm.nih.gov/gene/951152) | P44588 | Alternative ribosome-rescue factor A |
|  | HP HI0304 | [949387](http://www.ncbi.nlm.nih.gov/gene/949387) | P43980 | transcriptional regulator |
|  | HP HI0310 | [949975](http://www.ncbi.nlm.nih.gov/gene/949975) | P43982 | Holliday junction DNA helicase RuvB |
|  | HP HI0338 | [949451](http://www.ncbi.nlm.nih.gov/gene/949451) | P44646 | PerM family permease |
|  | HP HI0341 | [949447](http://www.ncbi.nlm.nih.gov/gene/949447) | P44649 | Acyl-CoA dehydrogenase NM domain-like |
|  | HP HI0400 | [950654](http://www.ncbi.nlm.nih.gov/gene/950654) | P44686 | DNA mismatch repair protein |
|  | HP HI0431 | [950606](http://www.ncbi.nlm.nih.gov/gene/950606) | P44709 | D-fructose-6-phosphate amidotransferase |
|  | HP  HI0451 | [950826](http://www.ncbi.nlm.nih.gov/gene/950826) | P43998 | RNA polymerase sigma factor |
|  | HP HI0453 | [49624](http://www.ncbi.nlm.nih.gov/gene/949624) | P43999 | twin-arginine translocation protein TatA/E |
|  | HP  HI0461 | [949548](http://www.ncbi.nlm.nih.gov/gene/949548) | Q57144 | coproporphyrinogen III oxidase |
|  | HP HI0507 | [949655](http://www.ncbi.nlm.nih.gov/gene/949655) | P44010 | EC 2.4.-.-: Transferases - Glycosyltransferases |
|  | HP HI0522 | [950631](http://www.ncbi.nlm.nih.gov/gene/950631) | Q57256 | GGY family of carbohydrate kinases signature 2 |
|  | HP HI0555 | [950248](http://www.ncbi.nlm.nih.gov/gene/950248) | Q57409 | integral membrane protein/LysR family transcriptional regulator |
|  | HP HI0559.1 | 9[49585](http://www.ncbi.nlm.nih.gov/gene/949585) | O86226 | Clathrin heavy-chain terminal domain protein |
|  | HP HI0636 | [949687](http://www.ncbi.nlm.nih.gov/gene/949687) | P44027 | Integrin beta tail domain family protein |
|  | HP HI0688 | [949721](http://www.ncbi.nlm.nih.gov/gene/949721) | P44037 | sn-glycerol-3-phosphate dehydrogenase subunit A |
|  | HP HI0696 | [950032](http://www.ncbi.nlm.nih.gov/gene/950032) | Q57523 | Lipocalins superfamily protein |
|  | HP HI0724 | [949751](http://www.ncbi.nlm.nih.gov/gene/949751) | P44844 | PLP-dependent transferases |
|  | HP HI0732 | [949763](http://www.ncbi.nlm.nih.gov/gene/949763) | P44045 | Multicopper oxidase, type 2 |
|  | HP HI0742 | [949769](http://www.ncbi.nlm.nih.gov/gene/949769) | P44047 | N-terminal nucleophile aminohydrolases (Ntn hydrolases) |
|  | HP HI0787 | [949801](http://www.ncbi.nlm.nih.gov/gene/949801) | P44052 | 30S ribosomal protein S17 |
|  | HP HI0817 | [949830](http://www.ncbi.nlm.nih.gov/gene/949830) | P44882 | Polypeptide chain release factor 2 (RF2) |
|  | HP HI0825 | [949928](http://www.ncbi.nlm.nih.gov/gene/949928) | P44056 | beta-methylgalactoside transporter inner membrane component |
|  | HP HI0840 | [949854](http://www.ncbi.nlm.nih.gov/gene/949854) | P44897 | ATPase domain of HSP90 chaperone /DNA topoisomerase II/histidine kinase |
|  | HP HI0843 | [949859](http://www.ncbi.nlm.nih.gov/gene/949859) | P44059 | Cytosine deaminase |
|  | HP HI0845 | [950597](http://www.ncbi.nlm.nih.gov/gene/950597) | P44900 | Chelatase |
|  | HP HI0847 | [950632](http://www.ncbi.nlm.nih.gov/gene/950632) | P31811 | transcriptional regulator with pssR |
|  | HP HI0855 | [949868](http://www.ncbi.nlm.nih.gov/gene/949868) | P44904 | SDH_alpha family  (Serine dehydratase alpha chain) |
|  | HP HI0862 | [949874](http://www.ncbi.nlm.nih.gov/gene/949874) | P44908 | preQ0 transporter |
|  | HP HI0870 | [949880](http://www.ncbi.nlm.nih.gov/gene/949880) | P44065 | Glycosyltransferase |
|  | HP HI0882 | [949886](http://www.ncbi.nlm.nih.gov/gene/949886) | P44068 | PLP-dependent transferases |
|  | HP HI0886 | [949888](http://www.ncbi.nlm.nih.gov/gene/949888) | P44069 | Ribosomal protein S6e signature |
|  | HP HI0907 | [949909](http://www.ncbi.nlm.nih.gov/gene/949909) | P44072 | Transcription factor CBF/NF-Y/archeal histone like |
|  | HP HI0908 | [949910](http://www.ncbi.nlm.nih.gov/gene/949910) | P44073 | RuBisCo,Cterminal domain |
|  | HP HI0931 | [949933](http://www.ncbi.nlm.nih.gov/gene/949933) | P44078 | glycyl-tRNA synthetase subunit alpha |
|  | HP HI0939 | [949939](http://www.ncbi.nlm.nih.gov/gene/949939) | P44080 | Type II secretory pathway, pseudopilin |
|  | HP HI0940 | [949941](http://www.ncbi.nlm.nih.gov/gene/949941) | P44081 | Putative type II secretory pathway, pseudopilin |
|  | HP HI0941 | [950292](http://www.ncbi.nlm.nih.gov/gene/950292) | P44082 | Putative type II secretory pathway, pseudopilin |
|  | HP HI0956 | [949959](http://www.ncbi.nlm.nih.gov/gene/949959) | P44954 | NAD(P)-linked oxidoreductase |
|  | HP HI0967 | [949914](http://www.ncbi.nlm.nih.gov/gene/949914) | P44086 | Lantibiotic regulatory protein signature |
|  | HP HI0974.1 | [949407](http://www.ncbi.nlm.nih.gov/gene/949407) | P46455 | sodium/panthothenate symporter |
|  | HP HI1000 | [949902](http://www.ncbi.nlm.nih.gov/gene/949902) | P44972 | ribonuclease P |
|  | HP HI1036 | [950018](http://www.ncbi.nlm.nih.gov/gene/950018) | P44097 | Carbamoyl phosphate synthetase, large subunit connection domain |
|  | HP HI1063 | [950635](http://www.ncbi.nlm.nih.gov/gene/950635) | P44107 | PE--lipooligosaccharidephosphorylethanolamine transferase |
|  | HP HI1073 | [950051](http://www.ncbi.nlm.nih.gov/gene/950051) | P45019 | CTP synthetase |
|  | HP HI1074 | [950048](http://www.ncbi.nlm.nih.gov/gene/950048) | P44110 | Cytochrome c biogenesis protein, transmembrane region |
|  | HP HI1098 | [949461](http://www.ncbi.nlm.nih.gov/gene/949461) | P44111 | Ribokinase-like |
|  | HP HI1149m | [950113](http://www.ncbi.nlm.nih.gov/gene/950113) | P45074 | Lipopolysaccharide assembly, LptA |
|  | HP HI1150 | [950694](http://www.ncbi.nlm.nih.gov/gene/950694) | P45075 | Lipopolysaccharide assembly, LptC-related |
|  | HP HI1151 | [949961](http://www.ncbi.nlm.nih.gov/gene/949961) | P45076 | ribosome-associated, YjgA family protein |
|  | HP HI1168 | [950127](http://www.ncbi.nlm.nih.gov/gene/950127) | P44117 | NagB/RpiA/CoA transferase-like |
|  | HP  HI1205 | [950166](http://www.ncbi.nlm.nih.gov/gene/950166) | P44127 | Agglutinin |
|  | HP HI1222 | [950617](http://www.ncbi.nlm.nih.gov/gene/950617) | P44129 | C4-dicarboxylate transporter/malic acid transport |
|  | HP HI1235 | [950185](http://www.ncbi.nlm.nih.gov/gene/950185) | P44131 | gamma-glutamyl phosphate reductase |
|  | HP HI1236 | [950175](http://www.ncbi.nlm.nih.gov/gene/950175) | P44132 | gamma-glutamyl phosphate reductase |
|  | HP HI1240 | [950105](http://www.ncbi.nlm.nih.gov/gene/950105) | P45122 | bicyclomycin/multidrug efflux system protein |
|  | HP HI1241 | [950179](http://www.ncbi.nlm.nih.gov/gene/950179) | P44133 | gamma-glutamyl phosphate reductase |
|  | HP HI1244 | [950183](http://www.ncbi.nlm.nih.gov/gene/950183) | P44134 | polysaccharide biosynthesis protein |
|  | HP HI1249 | [950126](http://www.ncbi.nlm.nih.gov/gene/950126) | P44137 | ABC-type transport system, periplasmic component |
|  | HP HI1266 | [950198](http://www.ncbi.nlm.nih.gov/gene/950198) | P44145 | chemotaxis protein methyltransferase |
|  | HP HI1269 | [950202](http://www.ncbi.nlm.nih.gov/gene/950202) | P44148 | ABC transporter substrate-binding protein |
|  | HP HI1310 | [949509](http://www.ncbi.nlm.nih.gov/gene/949509) | P44158 | (Trans)glycosidases |
|  | HP HI1326 | [950251](http://www.ncbi.nlm.nih.gov/gene/950251) | P44162 | Protein kinase-like (PK-like) superfamily protein |
|  | HP HI1355 | [950283](http://www.ncbi.nlm.nih.gov/gene/950283) | P44168 | flagellin N-methylase family protein |
|  | HP HI1375 | [950817](http://www.ncbi.nlm.nih.gov/gene/950817) | P44169 | Type I restriction enzyme R protein N terminus |
|  | HP HI1386 | [950299](http://www.ncbi.nlm.nih.gov/gene/950299) | P44171 | Transcription factor DP family |
|  | HP HI1399 | [950317](http://www.ncbi.nlm.nih.gov/gene/950317) | P44175 | dihydroorotate dehydrogenase 2 |
|  | HP HI1402 | [950318](http://www.ncbi.nlm.nih.gov/gene/950318) | P44177 | tail assembly protein |
|  | HP HI1405 | [950333](http://www.ncbi.nlm.nih.gov/gene/950333) | P44180 | gp27 putative head protein (Bacteriophage protein) |
|  | HP HI1406 | [950830](http://www.ncbi.nlm.nih.gov/gene/950830) | P44181 | "Winged helix" DNA-binding domain superfamily |
|  | HP HI1409 | [950321](http://www.ncbi.nlm.nih.gov/gene/950321) | P44183 | HI1409 family phage-associated protein |
|  | HP HI1412 | [950722](http://www.ncbi.nlm.nih.gov/gene/950722) | P45197 | Bacteriophage regulatory protein, Rha family |
|  | HP HI1414 | [950322](http://www.ncbi.nlm.nih.gov/gene/950322) | P44186 | putative lytic protein Rz |
|  | HP HI1423 | [950324](http://www.ncbi.nlm.nih.gov/gene/950324) | P44194 | "Winged helix" DNA-binding domain |
|  | HP HI1427 | [950330](http://www.ncbi.nlm.nih.gov/gene/950330) | P44196 | ABC-type transport system protein, periplasmic component |
|  | HP HI1434.2 | [949916](http://www.ncbi.nlm.nih.gov/gene/949916) | P56507 | aspartate-semialdehyde dehydrogenase |
|  | HP HI1446 | [950564](http://www.ncbi.nlm.nih.gov/gene/950564) | P44198 | dithiobiotin synthetase |
|  | HP HI1452 | [950785](http://www.ncbi.nlm.nih.gov/gene/950785) | P44201 | Predicted permease YjgP/YjgQ family |
|  | HP HI1456 | [950348](http://www.ncbi.nlm.nih.gov/gene/950348) | P44203 | Lipoprotein |
|  | HP HI1480 | [950577](http://www.ncbi.nlm.nih.gov/gene/950577) | P44209 | DNA-binding protein Dps |
|  | HP HI1482 | [950568](http://www.ncbi.nlm.nih.gov/gene/950568) | P44210 | adenine glycosylase |
|  | HP HI1485 | [950356](http://www.ncbi.nlm.nih.gov/gene/950356) | P44212 | Bacteriophage Mu Gam like |
|  | HP HI1486 | [950578](http://www.ncbi.nlm.nih.gov/gene/950578) | P44213 | MU-LIKE protein |
|  | HP HI1487 | [950580](http://www.ncbi.nlm.nih.gov/gene/950580) | P44214 | Uracil phosphoribosyltransferase |
|  | HP HI1489 | [950579](http://www.ncbi.nlm.nih.gov/gene/950579) | P44215 | RING/U-box  Superfamily |
|  | HP HI1492 | [950359](http://www.ncbi.nlm.nih.gov/gene/950359) | P44217 | ABC-type oligopeptide transport system, periplasmic component |
|  | HP HI1495 | [950744](http://www.ncbi.nlm.nih.gov/gene/950744) | P44219 | C4-dicarboxylate ABC transporter |
|  | HP HI1496 | [950362](http://www.ncbi.nlm.nih.gov/gene/950362) | P44220 | aTPase histidine kinase-DNA gyrase B-and HSP90-like domain protein |
|  | HP HI1498 | [950364](http://www.ncbi.nlm.nih.gov/gene/950364) | P44222 | Mu-like phage gp25 |
|  | HP HI1506 | [950374](http://www.ncbi.nlm.nih.gov/gene/950374) | P44228 | helix-extended loop-helix (HeH)/ LEM domain |
|  | HP HI1552 | [950563](http://www.ncbi.nlm.nih.gov/gene/950563) | P44251 | dethiobiotin synthetase |
|  | HP HI1563 | [950423](http://www.ncbi.nlm.nih.gov/gene/950423) | P44255 | transporting ATPase |
|  | HP HI1571 | [950428](http://www.ncbi.nlm.nih.gov/gene/950428) | P44260 | mu-like prophage protein gp29 |
|  | HP HI1599 | [950442](http://www.ncbi.nlm.nih.gov/gene/950442) | P44267 | beta and beta-prime subunits of DNA dependent RNA-polymerase |
|  | HP HI1601 | [950456](http://www.ncbi.nlm.nih.gov/gene/950456) | P44269 | outer membrane lipoprotein LolB |
|  | HP HI1622 | [950474](http://www.ncbi.nlm.nih.gov/gene/950474) | P44275 | N-terminal domain of cbl (N-cbl) |
|  | HP HI1626 | [950846](http://www.ncbi.nlm.nih.gov/gene/950846) | P44278 | PEP carboxykinase-like |
|  | HP HI1628 | [950472](http://www.ncbi.nlm.nih.gov/gene/950472) | P45279 | Dithiobiotin synthetase |
|  | HP HI1631 | [950845](http://www.ncbi.nlm.nih.gov/gene/950845) | P44279 | alpha/beta-Hydrolases |
|  | HP HI1643 | [950484](http://www.ncbi.nlm.nih.gov/gene/950484) | P45290 | Restriction endonuclease-like |
|  | HP HI1681 | [950865](http://www.ncbi.nlm.nih.gov/gene/950865) | P44290 | Exopolygalacturonase precursor |
|  | HP HI1701 | [949784](http://www.ncbi.nlm.nih.gov/gene/949784) | P44292 | molybdate ABC transporter permease |
|  | HP HI1703 | [950522](http://www.ncbi.nlm.nih.gov/gene/950522) | P45332 | lipopolysaccharide ABC transporter permease |
|  | HP HI1704 | [949709](http://www.ncbi.nlm.nih.gov/gene/949709) | P45333 | lipopolysaccharide ABC transporter permease LptF |
|  | HP HI1710 | [950870](http://www.ncbi.nlm.nih.gov/gene/950870) | P44294 | mannose-6-phosphate isomerase |
|  | HP HI1724 | [950875](http://www.ncbi.nlm.nih.gov/gene/950875) | P44297 | Cystatin/monellin |
|  | HP HI1736 | [950544](http://www.ncbi.nlm.nih.gov/gene/950544) | P44300 | E1-E2_ATPase family protein |
